# Trace and major elements in food supplements of different origin: Implications for daily intake levels and health risks

**DOI:** 10.1016/j.toxrep.2021.04.012

**Published:** 2021-05-02

**Authors:** A. Augustsson, A. Qvarforth, E. Engström, C. Paulukat, I. Rodushkin

**Affiliations:** a Department of Biology and Environmental Science, Linnaeus University, Kalmar, Sweden; b Division of Geosciences and Environmental Engineering, Luleå University of Technology, Luleå, Sweden; c ALS Laboratory Group, ALS Scandinavia AB, Luleå, Sweden

**Keywords:** Dietary supplements, Metals, Trace elements, Arsenic, Lead, Cadmium

## Abstract

As the use of food supplements increases, voices are being raised questioning the safety of these products. As a contribution to understanding the trace and major elemental composition of food supplements and their potential health risks, this study presents concentrations of 71 elements in 138 supplements, categorised into synthetic products and three groups of products with natural ingredients. Concentrations were converted into average daily doses (ADDs) and compared to tolerable daily intakes (TDIs). For elements where we found significant ADDs relative to the TDI a comparison was also made to the normal dietary intake. Our main findings are that: 1) Most elements display highly variable concentrations in food supplements; more so than in normal foodstuff; 2) For ten of the analysed elements some products rendered ADDs > 50 % of the TDI. Half of the elements were essential (Fe, Mn, Se, Mo, Zn), and as such motivated in food supplements. The other half (As, Pb, Cd, Al, Ni) represent non-essential and highly toxic elements, where the occurrence in food supplements ought to be viewed as contamination. Although none of these toxic metals were declared on any product’s table of content, several products gave high ADDs - in several cases even exceeding the TDIs; 3) The risk of reaching high ADDs for the toxic elements is strongly associated with products that contain marine ingredients (e.g. algae, mussels etc), and to some degree products of terrestrial plant-based origin. The health of consumers would benefit if food regulatory frameworks were updated to better address the risks of food supplements occasionally being contaminated with different toxic metals, for example by setting maximum permissible concentrations for a longer list of elements.

## Highlights

• Most elements display highly variable concentrations in food supplements.
• Some supplements with “natural ingredients” are contaminated by toxic metals.
• Food supplements can add significantly to the normal dietary metal intake.
• Especially products with marine ingredients (eg algae) can contain much arseni.
• Measures are needed to keep “high-risk-products” off the food supplement market.

## Graphical abstract

**Figure:**
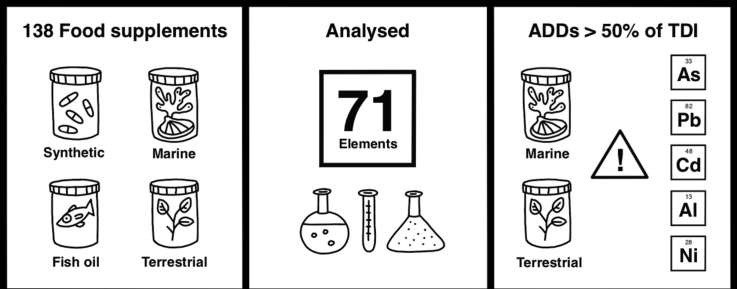

## 1 Introduction

Food supplements, sometimes referred to as “nutritional supplements” or “dietary supplements”, are according to the European Food Safety Authority [1] *“concentrated sources of nutrients (or other substances) with a nutritional or physiological effect”.* They are typically provided in dose forms (e.g. tablets or capsules), like medicines, but are not intended to treat or prevent specific diseases. Rather, the intention is to ensure sufficient intake of nutrients or other substances which support physiological functions. Sometimes the use of food supplements is justified, for example to remedy certain deficiency conditions, but in most cases a well-balanced diet is enough to meet the body’s demands for essential elements [2]. Nevertheless, the consumption of food supplements is steadily increasing among both adults [3,4,2,[5], [6], [7], [8], [9], [10]] and children [11]. Today approximately 50 % of the US population consume food supplements of some sort [7,10]. The consumption is high in Europe and in many other parts of the world as well, but reliable data on the extent of supplement use is scarce [7,12]. According to a market research report conducted by [13], however, the European market for food supplements in 2019 was valued at $ 14.95 billion, and expected to increase to $ 33.80 billion by 2027.

The steadily increasing commercial market for food supplements is broad and diverse, with a wide variety of products of different origin. They can be synthetic, like many vitamin- and mineral products (i.e. produced from raw materials of known, often certified, composition to achieve a product of uniform quality). Or they can be produced from natural vegetable or animal raw materials, e.g. herbs, plants, fungi, algae, lichens, fish etc. [4]. Most *synthetic* supplements are intended to provide the consumer with a well-defined intake of various essential vitamins, minerals or trace elements, thus preventing potential deficiencies. The purposes for consuming “*natural”* supplements are more diverse. For example, cranberries are argued to prevent urinary tract infection [14], turmeric is, through the action of curcuminoids, relieving symptoms of arthritis [15] and ashwagandha products are said to counteract stress and restore mental balance [16]. What kind of active substances that (possibly) are responsible for the desired effects are, however, not always well known and often not stated on the product labels. Supplements with *"natural"* ingredients are also more often marketed as health-promoting in general. Although synthetic vitamin and mineral products currently make up the highest proportion of the food supplement market, a drastic increase in use can be seen for supplements of natural origin ([13] [12];). This especially applies to algae-based products, where spirulina and chlorella dominate [17,8,18]. The major selling arguments for the increasingly popular algae supplements are their high content of proteins, fatty acids, vitamins (e.g. B12, C and E) and minerals (e.g. calcium, magnesium, phosphorus, zinc, copper, and iron) [18,19].

Unsurprisingly, there is a widespread notion that food supplements, which are bought without a prescription, are inherently safe and that the positive effects outweigh the risks, if any risks are perceived at all [20]. However, there is a non-negligible risk of ingesting dangerously high amounts of various substances together with food supplements [5,[21], [22], [23]]. This applies not only to constituents that are listed on the supplements’ labels, but also to undeclared and sometimes toxic compounds. Such compounds may originate from contamination of the source materials, which is e.g. the case for many supplements with elevated concentrations of toxic metal(loid)s [3,24]. Or, they can be natural constituents, which is for example the case with plant toxins such as pyrrolizidine alkaloids [25]. In addition, there is a risk of adverse interactions between food supplements and regular, prescribed drugs [26,27].

The first aim of the present study was to conduct a broad screening of the elemental composition of different kinds of food supplements. More than 70 different elements of both essential (vital elements needed in small quantities) and non-essential (not required for any vital bodily functions) character were analysed in 138 different food supplements. For the study, products were selected that could be categorised into, firstly, synthetic supplements, and secondly, three types of “natural products”, namely marine fish oil supplements, marine non-fish oil supplements and terrestrial plant-based supplements. The results were used to evaluate i) the average daily doses (ADDs) of elements in relation to tolerable daily intakes (TDIs) and normal dietary intakes, and ii) whether the highest ADDs were associated with certain types of food supplements.

## 2 Materials and methods

### 2.1 Sample collection

A total of 138 food supplements, many of them available on an international market, were purchased from leading Swedish pharmacies or health food stores online. The selection was made to include products that the stores themselves promoted as particularly popular, and to also include products aimed at children. Finally, products were selected to represent four main product groups based on origin, namely: 1) synthetic supplements (S, N = 29), i.e. different mineral- and vitamin products, 2) marine fish oil supplements (MF, N = 9), 3) marine non-fish oil supplements (M, N = 38), where the main ingredients were algae, mussels etc., and 4) terrestrial plant-based supplements (TPB, N = 62), which consisted mainly of dried and ground up plant materials (berries, herbs etc.).

The following details were recorded for each sample: name of the product, declared ingredients, brand name/manufacturer, batch number, dosage form and recommended daily dosage in mg. We do not report any product names in the article. This choice is made because, after all, we have only analysed a small portion of the entire food supplement market, and we do not fulfil the aims of the study better by pointing out which products in our selection contained harmful levels of various substances. Only the main ingredients and dosage form (e.g. tablet, capsule, oil, powder) are presented in the article’s supplementary material (Table S1).

To allow uniform blind handling, all samples were given a laboratory code number before the analysis.

### 2.2 Analytical procedure

Total concentrations of 71 elements of both non-essential and essential character (Ag, Al, As, Au, B, Ba, Be, Bi, Br, Ca, Cd, Ce, Co, Cr, Cs, Cu, Dy, Er, Eu, Fe, Ga, Gd, Ge, Hf, Hg, Ho, I, Ir, K, La, Li, Lu, Mg, Mn, Mo, Na, Nb, Nd, Ni, Os, P, Pb, Pd, Pr, Pt, Rb, Re, Rh, Ru, S, Sb, Sc, Se, Si, Sm, Sn, Sr, Ta, Tb, Te, Th, Ti, Tl, Tm, U, V, W, Y, Yb, Zn and Zr) were determined in the investigated food supplements. Sample preparation and chemical analyses were carried out at the accredited laboratory ALS Scandinavia in Luleå, Sweden.

#### 2.2.1 Sample preparations

Sample preparation was performed in Class 10 000 clean laboratory areas by personnel wearing clean room attire. General precautions detailed by Rodushkin et al. [28] were taken to minimize contamination. Laboratory materials used during sample preparation were soaked in 0.7 M nitric acid for 24 h at room temperature and rinsed with de-ionized Milli-Q water prior to use. In order to limit contamination risks during sample homogenization, whole tablets/capsules were prepared, while for powders, oils, oral solutions, approximately 1 g of material was used. Samples were weighed into 50 mL polypropylene tubes, 10 mL of nitric acid and 0.05 mL of hydrofluoric acid (Suprapur grade) were added, and the tubes were then loosely cupped and left inside a fume hood at room temperature overnight. Sample digestion was performed using graphite heating blocks at 110 °C for 120 min. Digests were diluted to 30 mL with Milli-Q water, homogenized by agitation and further diluted with 1.4 M nitric acid in order to provide a total dilution factor of 1000 (v/m). Two preparation blanks, two duplicate samples and two reference materials were included in each preparation batch of 36 samples.

#### 2.2.2 Instrumental analysis

Concentrations of the 71 elements were determined by double-focusing, sector field inductively coupled plasma mass-spectrometry (ICP-SFMS, ELEMENT XR, Thermo Scientific, Bremen, Germany), operated with methane addition to the plasma [29] and equipped with solution nebulization sample introduction system. Matrix effect correction was accomplished by internal standardization (indium added to all measurement solutions at 2.5 μg/l concentration) and quantification was done by external calibration with synthetic, concentration-matched standards. Further details on the operation conditions and measured parameters as well as figures of merit of the method can be found elsewhere [30]. The limits of detection (LOD) were calculated as three times the standard deviation for element’s concentrations detected in preparation blanks (n>15). The LODs of all elements are presented in the supplementary material, Table S2.

#### 2.2.3 Quality control and quality assurance (QC/QA) procedures

The accuracy of the ICP-SFMS data was assessed by analyses of the two certified reference materials (CRMs), namely tea GBW 07605 (Institute of Geophysical and Geochemical Exploration. Langfang, China) and wheat flour NBS 1467a (NIST, Gaithersburg, USA). Those CRMs were selected on the basis of expected similarities in matrix and elemental concentrations. The ICP-SFMS results were within 10 % RSD range from certified, indicative or information values, where such were available. Method reproducibility was evaluated from replicate preparation/analysis of samples and CRMs, and as a rule was better than 10 % RSD for elements presented in tested matrixes at concentrations 10 times above respective LODs. In order to account for as many sources of deviation as possible, samples containing the highest concentrations of As, Cd and Pb when first tested underwent repeated testing.

### 2.3 Comparison between average daily intakes (ADDs) and tolerable daily intakes (TDIs) of individual elements

To assess the risk of a potentially detrimental intake of different elements, concentrations obtained from all supplements were translated into average daily doses, expressed per kg bodyweight (ADDs; μg/kg/day) and compared with tolerable daily intakes (TDIs). The applied assessment methodology is similar to the analysis for drugs recommended by the EMA ICH guideline Q3D (R1) on elemental impurities [31]. While so called PDE (Permitted Daily Exposure) values are used as estimated “safe levels” according to ICH guideline Q3D (R1), we used oral TDIs in our work.

The ADDs were calculated by multiplying concentrations in food supplements (μg of a certain element per kg food supplement) with the recommended maximum daily intake of the different supplements (kg of supplement per day), divided by body weight (kg). Concentrations < LOD were hereby set to half the detection limit. The expression of doses per kilogram body weight lead us to focus on adult women and children (aged 3–6 years and 9–11 years), who are particularly vulnerable. According to Figueiredo et al. [7] and Vargas-Murga et al. [12], women also use supplements to a greater extent than men. Calculations were then performed for normal-weight and underweight women and children, respectively, to consider both average and worst-case intakes. Normal weights were hereby characterised by arithmetic means and underweight was defined by a body weight corresponding to the 1^st^ percentile reported by Filipsson et al. [32]. For adult women, the body weights used were 67.7 and 47 kg for the normal weight and underweight scenarios, respectively. For children the corresponding figures were 18.4 and 13.6 kg (3–6 year olds) and 38.9 and 25.4 kg (9–11 year olds). Of the 138 dietary supplements included in the study, 42 were labelled as suitable for children (S = 5; M = 17; MF = 4; TPB = 16) and calculations that concerned children were performed only for these products.

Assessments were performed for all elements for which a TDI value, regarding oral intake specifically, could be found in the literature. This applied to 39 elements (23 individual elements + the sum of 16 rare earth elements, REEs). Guidance values available as tolerable weekly intakes (TWIs) were converted into, and presented as, daily doses in our assessment. The first choice when TDI values (or TWIs) were selected was reports published by the European Food Safety Authority (EFSA), which directly focus on dietary intake. EFSA has published TDI values ​​for Al [33], As [34], Cd [35], Fe [36], Hg [37], Ni [38], Pb [39] and U [40]. The second choice was to consult the Integrated Risk Information System (IRIS) database of the US Environmental Protection Agency. Here, oral TDI-values were found for Ag [41], B [42], Ba [43], Be [44], Mo [45], Cr III [46], Mn [47], Sb [48], Se [49] and Sr [50]. Further, four oral TDI values (for Co, Cu, Zn and Sn) were retrieved from the Dutch RIVM, National Institute of Public Health and the Environment [51,52], and a TDI value for Br was found in a report The Committee on Toxicity of Chemicals in Food, Consumer Products and the Environment [53]. For the 16 REEs (Ce, Dy, Er, Eu, Gd, Ho, La, Lu, Nd, Pr, Sc, Sm, Tb, Tm, Y, Yb) Rodríguez-Hernández et al. [54] suggest a total oral TDI of 61 μg/kg/day. Others [55] suggest a corresponding value for REEs of 70 μg/kg/day. The former, lower, value was used in the comparative analysis of this paper.

Elements for which we found ADDs > 50 % of TDI in at least two of the investigated food supplements (for either women or children) were examined in more detail.

## 3 Results and discussion

### 3.1 Element concentrations in food supplements

There are food regulations that state the maximum permissible concentrations (MPCs) of different elements in different types of food. For food supplements, however, MPCs are only given for Cd, Pb and Hg (EU Commission Regulation (EC) No. 629/2008 of 2 July 2008 amending Regulation (EC) No 1881/2006). These MPCs are 1000 or 3000 μg/kg for Cd (the latter for supplements containing dried seaweed or bivalve molluscs [56]), 3000 μg/kg for Pb, and 100 μg/kg for Hg. None of the supplements analysed in this study exceeded these limits (Table 1), although Cd and Pb were present in relatively high concentrations in some of the products. Maximum concentrations were found at 1500 μg/kg for Cd and 1700 μg/kg for Pb. Concentrations in all the analysed products are given in the supplementary material, Table S2. Others have also reported that Cd and Pb, but in most cases not Hg, may occasionally approach or even exceed the MPCs for food supplements [3,6,18,57]. Generalisations about the presence of these elements in food supplements are, however, difficult to make since concentrations differ considerably between products (and thus studies, depending on the selection of products). For example, considering four previous studies that have all investigated a large number of food supplements of mixed origin (Ćwieląg-Drabek et al. [6] Poniedziałek et al. [10] Korfali et al. [9] and Amariei et al. [3]), the reported maximum concentrations for Cd, Pb and Hg are in the range 940–500,000 μg/kg, 36–50,000 μg/kg and 0.4–550 μg/kg, respectively, thus varying by several orders of magnitude. The median ranges reported by the same studies are equally variable; 20–51,000 μg/kg for Cd, 2.9–15,000 μg/kg for Pb and 0.022–45 μg/kg for Hg. Thus ranges of Cd and Pb concentrations in food supplements are wider than in most other types of commercially available food, as reported by the EFSA [58,39]. EFSA has presented extensive summaries of the occurrence of different metals in different types of food across the EU member states. In their compilations of Cd and Pb concentrations in “fruit” samples, for example, we see that the P95 concentrations are about 80 times the P5 concentrations for both these metals [58,39]. “Fruit” is a category that is both highly heterogeneous and an important contributor to the dietary intake of Cd and Pb, suitable for a comparison of concentration ranges. The food supplements included in the present study have a larger difference between P5 and P95 concentrations; 240 times for Cd and 220 times for Pb. Arsenic is another toxic metal(loid) with MPCs in most commercial foodstuff. For this element, the most recent EFSA compilation shows a 34-fold difference between P5 and P95 concentrations in European “fruit” samples [34]. Again, the corresponding interval in food supplements from this study was strikingly wider, with the P95 concentration being 1220 times as high as the P5 concentration. And yet, the variability for food supplements would have been even higher if data from previous studies, e.g. from the above references, is included. The high variability in food supplement Cd, Pb and As content,

**Table 1:** Summary of element concentrations (mean, median, min and max) in the four different groups of food supplements analysed. Data for each individual product is presented in the supplementary material, **Table S2**. Note different concentration units.

		Synthetic, S (n = 29)				Marine non-fish-oil, M (n = 38)				Marine fish-oil, MF (n = 9)				Terrestrial Plant Based, TPB (n = 62)			
	
Element		Mean ± SD	Median	Min	Max	Mean ± SD	Median	Min	Max	Mean ± SD	Median	Min	Max	Mean ± SD	Median	Min	Max
**Ag**	μg/kg	2.0 **±** 1.9	1.5	0.50	7.6	12 **±** 21	5.3	0.50	110	1.3 **±** 1.4	0.50	0.50	4.8	4.2 **±** 6.3	2.0	0.50	33
**Al**	mg/kg	17 **±** 25	4.6	0.10	99	93 **±** 99	75	0.39	320	20 **±** 51	2.0	0.42	160	160 **±** 400	19	0.10	2200
**As**	μg/kg	57 **±** 120	10	1.0	470	2700 **±** 7000	280	2.2	40,000	19 **±** 35	6.4	1.0	110	63 **±** 91	27	1.0	430
**Au**	μg/kg	5.1 ± 23	0.50	0.50	130	0.66 **±** 0.39	0.50	0.50	2.1	0.50 **±** 0.00	0.50	0.50	0.50	0.56 **±** 0.31	0.50	0.50	2.8
**B**	mg/kg	23 **±** 69	2.2	0.25	320	13 **±** 23	7.5	0.25	140	1.3 **±** 1.9	0.60	0.25	6.1	11 **±** 14	5.1	0.25	88
**Ba**	mg/kg	1.9 **±** 6.3	0.48	0.10	35	6.4 **±** 8.6	2.3	0.10	33	0.46 **±** 1.0	0.10	0.10	3.2	6.1 **±** 11	1.8	0.10	64
**Be**	μg/kg	14 **±** 51	1.5	0.50	280	8.7 **±** 12	4.5	0.50	55	2.2 **±** 5.1	0.50	0.50	16	7.4 **±** 13	2.6	0.50	77
**Bi**	μg/kg	1.1 **±** 1.0	0.50	0.50	4.0	8.5 **±** 17	3.9	0.50	88	2.2 **±** 3.0	0.50	0.50	9.4	3.0 **±** 5.2	0.50	0.50	28
**Br**	mg/kg	23 **±** 78	1.0	0.25	390	48 **±** 100	4.2	0.25	470	4.2 **±** 3.7	2.3	0.25	10	12 **±** 35	3.2	0.25	270
**Ca**	g/kg	65 **±** 120	3.7	0.011	510	17 **±** 34	3.0	0.024	140	14 **±** 41	0.037	0.0036	120	16 **±** 39	2.0	0.0075	220
**Cd**	μg/kg	80 **±** 140	6.0	0.50	510	140 **±** 270	39	0.50	1500	22 **±** 64	0.50	0.50	190	49 **±** 98	14	0.50	530
**Ce**	μg/kg	200 **±** 640	14	0.50	3400	360 **±** 690	100	0.50	3100	41 **±** 120	1.7	0.50	350	170 **±** 420	53	0.50	3100
**Co**	μg/kg	430 **±** 920	45	0.50	4200	510 **±** 1000	280	1.0	5700	160 **±** 460	1.9	0.50	1400	260 **±** 610	57	0.50	3800
**Cr**	mg/kg	11 **±** 23	0.47	0.0062	88	1.0 **±** 2.8	0.49	0.010	17	0.55 **±** 1.3	0.058	0.010	4.0	2.0 **±** 7.8	0.28	0.0030	48
**Cs**	μg/kg	2.9 **±** 5.8	0.50	0.50	28	31 **±** 52	13	0.50	270	5.8 **±** 14	0.50	0.50	43	69 **±** 140	13	0.50	770
**Cu**	mg/kg	200 **±** 430	2.7	0.023	1900	22 **±** 80	1.9	0.0082	360	100 **±** 300	0.057	0.0025	910	55 **±** 200	2.4	0.0025	1000
**Dy**	μg/kg	30 **±** 55	1.9	0.50	190	36 **±** 56	9.2	0.50	200	6.4 **±** 18	0.50	0.50	53	20 **±** 48	3.5	0.50	340
**Er**	μg/kg	20 **±** 37	1.8	0.50	140	27 **±** 50	5.9	0.50	230	4.0 **±** 10	0.50	0.50	32	11 **±** 26	2.2	0.50	180
**Eu**	μg/kg	6.8 **±** 15	0.50	0.50	73	8.1 **±** 15	2.2	0.50	60	1.8 **±** 3.9	0.50	0.50	12	4.9 **±** 13	0.76	0.50	95
**Fe**	g/kg	3.8 **±** 6.8	0.090	0.00010	26	1.0 **±** 1.8	0.3	0.00034	8.3	3.6 **±** 11	0.0010	0.00010	32	0.61 **±** 2.6	0.029	0.00010	16
**Ga**	μg/kg	27 **±** 40	7.2	2.5	150	28 **±** 29	21	2.5	130	20 **±** 53	2.5	2.5	160	25 **±** 90	5.4	2.5	710
**Gd**	μg/kg	32 **±** 60	2.0	0.50	210	35 **±** 57	12	0.50	230	7.1 **±** 20	0.50	0.50	60	22 **±** 55	3.8	0.50	380
**Ge**	μg/kg	56 **±** 110	5.0	5.0	430	28 **±** 62	12	5.0	310	5.0 **±** 0.00	5.0	5.0	5.0	38 **±** 160	5.0	5.0	1100
**Hf**	μg/kg	6.6 **±** 11	1.1	0.50	42	7.7 **±** 10	3.5	0.50	44	1.3 **±** 1.4	0.50	0.50	4.6	6.8 **±** 13	2.3	0.50	89
**Hg**	μg/kg	1.0 **±** 0.98	0.50	0.50	4.3	5.3 **±** 6.9	3.1	0.50	38	1.1 **±** 0.95	0.50	0.50	2.8	2.1 **±** 3.4	0.50	0.50	16
**Ho**	μg/kg	7.1 **±** 13	0.50	0.50	47	8.4 **±** 14	1.9	0.50	60	1.7 **±** 3.5	0.50	0.50	11	4.2 **±** 10	0.50	0.50	67
**I**	mg/kg	62 **±** 78	15	0.28	290	200 **±** 650	4.8	0.13	3800	17 **±** 23	8.2	0.78	76	24 **±** 54	5.4	0.13	330
**Ir**	μg/kg	0.50 **±** 0.00	0.50	0.50	0.50	0.50 **±** 0.00	0.50	0.50	0.50	0.50 **±** 0.00	0.50	0.50	0.50	0.50 **±** 0.00	0.50	0.50	0.50
**K**	g/kg	4.5 **±** 16	0.10	0.0025	72	13 **±** 22	9.5	0.031	130	0.21 **±** 0.51	0.028	0.0025	1.6	10 **±** 12	5.3	0.0057	61
**La**	μg/kg	170 **±** 350	12	0.50	1700	190 **±** 350	54	0.50	1400	33 **±** 94	0.50	0.50	280	130 **±** 260	26	0.50	1400
**Li**	μg/kg	440 **±** 1700	88	15	9300	360 **±** 570	170	15	2500	95 **±** 130	63	15	430	180 **±** 300	100	15	1900
**Lu**	μg/kg	2.5 **±** 3.9	0.50	0.50	16	5.2 **±** 12	0.50	0.50	59	0.85 **±** 1.0	0.50	0.50	3.6	1.6 **±** 2.9	0.50	0.50	20
**Mg**	g/kg	45 **±** 87	2.9	0.0021	410	3.7 **±** 4.9	2.9	0.016	29	7.2 **±** 21	0.0075	0.00060	63	10 **±** 44	1.3	0.0024	260
**Mn**	mg/kg	350 **±** 800	6.5	0.047	3800	75 **±** 180	32	0.042	860	10 **±** 24	0.044	0.010	73	130 **±** 350	16	0.029	2300
**Mo**	mg/kg	13 **±** 30	0.075	0.0025	100	0.37 **±** 0.52	0.21	0.0025	2.7	0.057 **±** 0.10	0.010	0.0025	0.24	0.62 **±** 3.2	0.077	0.0025	25
**Na**	g/kg	17 **±** 36	0.53	0.091	150	8.0 **±** 14	4.8	0.064	81	0.93 **±** 1.3	0.68	0.0033	4.3	2.9 **±** 12	0.62	0.0033	91
**Nb**	μg/kg	99 **±** 210	5.2	0.50	760	15 **±** 18	7.3	0.50	65	7.5 **±** 20	0.50	0.50	62	32 **±** 93	4.2	0.50	650
**Nd**	μg/kg	120 **±** 260	23	0.50	1300	180 **±** 330	67	0.50	1300	31 **±** 84	1.8	0.50	250	99 **±** 230	28	0.50	1500
**Ni**	μg/kg	570 **±** 790	160	25	3600	930 **±** 1000	650	25	5300	250 **±** 680	25	25	2100	1200 **±** 1800	600	25	9300
**Os**	μg/kg	2.5 **±** 0.00	2.5	2.5	2.5	2.5 **±** 0.00	2.5	2.5	2.5	2.5 **±** 0.00	2.5	2.5	2.5	2.5 **±** 0.00	2.5	2.5	2.5
**P**	g/kg	4.1 **±** 18	0.11	0.0036	100	15 **±** 23	7.7	0.010	110	0.40 **±** 0.70	0.067	0.0081	2.2	11 **±** 29	1.4	0.011	180
**Pb**	μg/kg	54 **±** 65	28	1.1	260	250 **±** 290	190	2.2	1300	63 **±** 140	3.3	0.50	430	160 **±** 310	62	0.50	1700
**Pd**	μg/kg	8.9 **±** 14	2.5	2.5	55	5.6 **±** 7.5	2.5	2.5	37	6.6 **±** 10	2.5	2.5	32	11 **±** 49	2.5	2.5	390
**Pr**	μg/kg	30 **±** 71	2.2	0.50	370	43 **±** 80	12	0.50	340	7.1 **±** 20	0.50	0.50	60	24 **±** 54	6.6	0.50	360
**Pt**	μg/kg	0.50 **±** 0.00	0.50	0.50	0.50	0.63 **±** 0.39	0.50	0.50	2.2	0.56 **±** 0.17	0.50	0.50	1.0	0.56 **±** 0.36	0.50	0.50	3.2
**Rb**	mg/kg	0.64 **±** 1.6	0.064	0.0050	6.5	4.2 **±** 5.7	2.1	0.0050	27	0.18 **±** 0.31	0.031	0.0050	0.89	14 **±** 25	4.2	0.0050	110
**Re**	μg/kg	0.52 **±** 0.11	0.50	0.50	1.1	3.5 **±** 8.0	0.50	0.50	34	0.50 **±** 0.00	0.50	0.50	0.50	0.72 **±** 0.81	0.50	0.50	5.0
**Rh**	μg/kg	4.5 **±** 4.4	2.5	2.5	16	3.8 **±** 2.8	2.5	2.5	12	3.0 **±** 1.4	2.5	2.5	6.6	3.1 **±** 2.2	2.5	2.5	15
**Ru**	μg/kg	2.5 **±** 0.00	2.5	2.5	2.5	2.5 **±** 0.00	2.5	2.5	2.5	2.5 **±** 0.00	2.5	2.5	2.5	2.5 **±** 0.00	2.5	2.5	2.5
**S**	g/kg	3.6 **±** 7.1	0.61	0.011	35	8.7 **±** 10	6.4	0.35	51	0.59 **±** 0.63	0.49	0.0025	2.0	1.7 **±** 1.7	1.1	0.018	9.0
**Sb**	μg/kg	48 **±** 180	3.4	0.50	990	68 **±** 220	11	0.50	1300	4.5 **±** 6.0	1.5	0.50	17	51 **±** 180	4.7	0.50	1300
**Sc**	μg/kg	14 **±** 24	3.1	0.50	110	50 **±** 82	19	0.50	350	4.6 **±** 12	0.50	0.50	36	28 **±** 120	5.6	0.50	970
**Se**	mg/kg	32 **±** 71	0.78	0.025	320	3.9 **±** 16	0.080	0.025	94	5.6 **±** 17	0.050	0.025	50	4.3 **±** 16	0.025	0.025	92
**Si**	mg/kg	200 **±** 320	71	2.5	1200	520 **±** 1300	110	2.5	6700	40 **±** 36	34	5.8	110	390 **±** 420	200	2.5	1500
**Sm**	μg/kg	25 **±** 52	2.5	0.50	250	36 **±** 62	11	0.50	260	6.3 **±** 17	0.50	0.50	53	20 **±** 50	3.9	0.50	360
**Sn**	μg/kg	25 **±** 34	13	0.50	110	35 **±** 42	21	2.2	200	12 **±** 24	1.8	0.50	74	29 **±** 47	11	0.50	230
**Sr**	mg/kg	32 **±** 51	3.2	0.063	180	57 **±** 100	19	0.24	510	4.5 **±** 13	0.062	0.010	39	15 **±** 22	6.1	0.026	100
**Ta**	μg/kg	38 **±** 52	25	25	300	25 **±** 0.00	25	25	25	290 **±** 800	25	25	2400	45 **±** 88	25	25	660
**Tb**	μg/kg	4.8 **±** 8.2	0.50	0.50	26	5.6 **±** 8.4	1.8	0.50	32	1.4 **±** 2.8	0.50	0.50	9.0	3.3 **±** 7.8	0.50	0.50	56
**Te**	μg/kg	2.8 **±** 1.5	2.5	2.5	11	2.9 **±** 1.5	2.5	2.5	9.0	2.5 **±** 0.00	2.5	2.5	2.5	2.5 **±** 0.00	2.5	2.5	2.5
**Th**	μg/kg	6.1 **±** 7.5	2.6	0.50	35	110 **±** 300	21	0.50	1400	7.6 **±** 21	0.50	0.50	62	16 **±** 38	4.6	0.50	270
**Ti**	mg/kg	410 **±** 1000	1.5	0.010	3800	7.2 **±** 12	3.7	0.027	73	0.55 **±** 1.0	0.083	0.051	3.2	170 **±** 710	0.89	0.010	5200
**Tl**	μg/kg	5.3 **±** 9.3	1.7	0.50	37	22 **±** 39	5.8	0.50	200	5.9 **±** 16	0.50	0.50	49	18 **±** 36	4.1	0.50	190
**Tm**	μg/kg	2.8 **±** 4.6	0.50	0.50	18	4.5 **±** 9.2	0.50	0.50	46	0.88 **±** 1.1	0.50	0.50	3.9	1.7 **±** 3.2	0.50	0.50	23
**U**	μg/kg	100 **±** 230	5.9	0.50	950	75 **±** 99	39	0.50	450	170 **±** 510	2.2	0.50	1500	27 **±** 60	6.5	0.50	350
**W**	μg/kg	8.5 **±** 12	3.7	0.50	46	120 **±** 500	21	0.50	3100	3.4 **±** 5.1	1.2	0.50	15	14 **±** 20	6.9	0.50	120
**V**	μg/kg	580 **±** 950	47	2.5	3700	460 **±** 630	250	2.5	2900	450 **±** 1300	7.2	2.5	4000	440 **±** 1900	60	2.5	15,000
**Y**	μg/kg	310 **±** 640	22	0.50	2600	230 **±** 360	67	0.50	1300	53 **±** 150	1.1	0.50	460	150 **±** 360	22	0.50	2000
**Yb**	μg/kg	15 **±** 27	1.5	0.50	100	31 **±** 71	4.5	0.50	350	3.1 **±** 7.5	0.50	0.50	23	9.0 **±** 20	1.8	0.50	140
**Zn**	mg/kg	3300 **±** 5400	51	0.050	18,000	400 **±** 1600	16	0.050	9100	870 **±** 2600	0.24	0.050	7700	950 **±** 3400	14	0.21	22,000
**Zr**	μg/kg	200 **±** 290	30	2.1	940	370 **±** 530	180	2.5	2500	48 **±** 69	9.3	0.50	200	250 **±** 470	110	0.50	3100
**REE**	mg/kg	1.0 **±** 2.0	0.074	0.0080	8.5	1.2 **±** 2.0	0.40	0.0086	8.0	0.20 **±** 0.57	0.010	0.0080	1.7	0.70 **±** 1.6	0.17	0.0080	11

together with the fact that most food supplement consumers eat the same product every day, are strong arguments for an improved quality control of these products. In the case of most other food types, the effect of concentration differences for consumers is largely evened out by the fact that we buy different kinds of dairy products, meat, vegetables and so on, and from different producers. Mercury, with much lower concentrations relative to its MPC in food supplements (Table 1), also has much less variable concentrations than Cd, Pb and As, with only a 12-fold difference between the P5 and P95. Only four other elements (Te, Pt, Rh, Pd) in this study exhibited a lower concentration variability.

When looking at all 71 analysed elements, however, large concentration spans are the rule rather than the exception (Table 1). The top five P95/P5 ratios were found for Zn (with a ∼40,000-fold difference), Ti (∼20,200), Cu (∼19,000), Fe (∼16,000) and Mn (∼18,000). Such wide concentration intervals in different food supplements confirm the findings of previously published studies [3,6,7,9,10,22]. Former assessments, however, have looked at far fewer elements: among the toxic ones most often Pb, As, Cd, Hg, Cr, Ni, and among those that also have essential properties mainly Ca, Co, Cu, Fe, K, Mg, Mn, Na, Zn, Se. Analysing the concentration patterns further we see that the distributions of most elements are positively skewed – i.e., that only a limited number of products are oriented towards the upper end of the min–max concentration span. Note, for example, that mean values for most elements in Table 1 deviate substantially from the medians. This has implications from a risk perspective, since it means on the one hand that supplement intakes in most cases are not associated with a high risk, but on the other hand that the risk in a worst-case scenario can be unacceptable.

### 3.2 Average daily intakes (ADDs) in relation to tolerable daily intakes (TDIs)

#### 3.2.1 Which elements are elevated, and in which products?

Although Cd, Pb and Hg concentrations don’t exceed the MPCs according to EU Commission Regulation (​​EC) no. 1881/2006 (Table 1), the intake of Cd and Pb via food supplements – as well as a number of other elements – can in some cases still be significant in relation to the tolerable daily intake (TDI). Table 2 summarises the ADDs as a % of the corresponding TDIs for women and young children. Results are presented here for the P50 (median), P75, P95 and maximum ADD values. For each element these results are linked to the food supplement that gives the highest daily intake, as well as the average intake (median) and the intake at the 75^th^ and 95^th^ percentiles. The exact ADDs for each individual product are provided in the supplementary material Table S3. ADDs exceeding 10 % of the TDI are highlighted in bold in Table 2. When it comes to both women and children, such examples included Al, As, B, Ba, Br, Cd, Co, Cu, Fe, Mn, Mo, Ni, Pb, Se, Sr and Zn. For 11 of these elements there were also examples among the analysed supplements where ADDs exceed 50 % of the TDI (bold and shaded in Table 2). For 10 of the elements it applied to two or more products, and these were evaluated further (see method section). Half of these are essential (Fe, Mn, Se, Mo, Zn), and as such their use in food supplements can be motivated. The other half (As, Pb, Cd, Al, Ni) consist of non-essential/toxic elements, and as such their presence in food supplements should be considered as unintentional contamination. Table 3 summarises the toxic effects of these ten elements, and for the five essential elements deficiency effects are also shown. Fig. 1 presents boxplots of ADDs for these 10 elements associated with the different supplement groups.

**Table 2:** Average Daily Doses (ADDs) in % of TDI, associated with the investigated food supplements. For each element the results below are linked to the food supplement that gives the average (median) and maximum intakes, and the intakes at the 75^th^ and 95^th^ percentiles. To get corresponding results for older children (9–11 years), i.e. also ADDs in % of TDI, the numbers for the younger children in this table can be multiplied by 0.47 (normal weight) or 0.54 (underweight).

**Bold figures:** ADDs > 10 % of TDI; **shaded and bold figures**: ADDs > 50 % of TDI.

**Table 3:** Tolerable daily intakes (TDIs) of the five essential (Fe, Mn, Mo, Se, Zn) and toxic (As, Pb, Cd, Al, Ni) elements together with critical endpoints used in TDI-determination and examples of other adverse health effects. For the five essential elements, effects of deficiency are also shown.

Element	TDI and critical endpoint	Other adverse health effects	Deficiency symptoms
**Fe**	800 μg/kg/day^j^ Data are considered insufficient and the connection between intake and adverse effects is uncertain^k^.	Damage of the intestinal mucosa, blood losses, loose stools, hypovolemic shock^k, l^. Chronic iron overload might cause liver cirrhosis^k^.	Globally the most common nutritional deficiency disorder^l^. Fatigue, muscle weakness, impaired immune system, negative effects on nail and hair growth^l^.

**Mn**	140 μg/kg/day^m^ The Mn TDI value of 140 μg/kg/day is based on adverse **CNS effects**^m^.	Adverse effects on the central nervous system, effects on liver and heart, endocrine diseases and reproductive disorders in men^n^.	Unusual, few human studies^n, o, p^. Manganese is needed for enzyme functions, normal growth, bone mineralisation, protein and energy metabolism and metabolic regulation^n, q^.

**Mo**	5 μg/kg/day^r^ Critical endpoint for TDI is **increased uric acid levels**^r^.	According to EFSA^s^, no chronic studies are available for a relevant risk assessment. Aching joints and gout-like symptoms are reported as possible effects^s^.	No clinical signs of deficiency in healthy people have been observed^p, t^.

**Se**	5 μg/kg/day^u^ Critical effect systems for selenium TDI-setting are the **nervous-, haematological and dermal systems**^u^.	Nausea, vomiting, nail changes, dryness of hair, hair loss, swelling of fingertips, fatigue, irritability (high doses, about 250 mg)^s, v^, mottled teeth, skin lesions and changes in peripheral nerves^s^.	Possible involvement in e.g. skeletal myopathy, muscle weakness, cardiomyopathy and degeneration of organs and tissues^w^.

**Zn**	500 μg/kg/day^x^ Based on a LOAEL value of 1 mg/kg/day, where the critical endpoint is **decrease in ESOD (erythrocyte superoxide dismutase) activity** in women^x, y^.	Stomach cramps, nausea, vomiting (short time high exposure)^y^, anaemia, haematological effects, damage of pancreas changes in blood lipid profile (chronic exposure) ^x, y^.	Growth inhibition including birth defects, poor immune system, delayed wound healing, skin sores, loss of appetite and loss of cognitive function ^y, z^.

**As**	0.3–8 μg/kg/day^a^, 0.3 μg/kg/day^b^ EFSA^a^ presents a BMDL01 interval of 0.3–8 μg/kg/day for dietary intake of inorganic As. The critical endpoints are **cancers of the lung and bladder, and dermal lesions**. The lower figure of the interval (0.3 μg/kg/day), which we use here, is the same as stated for oral exposure to inorganic arsenic by IRIS.	Cardiovascular diseases, neurotoxicity, abnormal glucose metabolism, diabetes, development disturbances in foetuses and infants^a,c^. Cancer classification 2012 (arsenic and inorganic arsenic compounds): *group 1*, carcinogenic to humans^d^ .	**–**

**Pb**	0.5 μg/kg/day^e^ The lowest BMDL01 level after oral lead exposure is 0.5 μg/kg/day, when **developmental neurotoxicity in children** is the critical endpoint^e^. There are reasons to suspect that the true TDI value should be even lower. However, the provisional limit of 0.5 μg/kg/day is used in this study.	Neurotoxicity, high systolic blood pressure, kidney disease, reduced IQ among children^e^. Cancer classification 2006; lead (inorganic): *group 2A*, probably carcinogenic to humans, lead (organic): *group 3*, not classifiable as to its carcinogenicity to humans^d^.	**–**

**Cd**	0.36 μg/kg/day^f^ The EFSA TWI value (2.5 μg/kg/week = 0.36 μg/kg/day) is set based on the critical endpoint being **kidney toxicity**.	Cancer (of the lung, endometrium, bladder and breast), and bone demineralisation^g^. Cancer classification 2012 (cadmium and cadmium compounds): *group 1*, carcinogenic to humans ^d^.	**–**

**Al**	140 μg/kg/day^h^ The EFSA TWI value (1 mg/kg/week = 140 μg/kg/day) is based on **neurotoxicity, effects on the male reproductive system and embryotoxicity**^h^.	Data on human toxicity are uncertain. The suggested effects include neurotoxicity and involvement in Alzheimer's disease and other neurodegenerative diseases^h^. Cancer classification 2012 (Al production): *group 1,* carcinogenic to humans ^d^.	**–**

**Ni**	2.8 μg/kg/day^i^ The nickel TDI value from represents the BMDL10 dose, with the critical endpoint being **post‐implantation foetal loss**^i^.	Contact dermatitis, gastrointestinal disorders (e.g. vomiting and diarrhoea) and neurological symptoms^i^. Cancer classification 2012 (Ni compounds): *group 1,* carcinogenic to humans ^d^.	**–**

^a^[34], ^b^[90], ^c^[92], ^d^[89], ^e^[39], ^f^[35], ^g^[82], ^h^[33], ^i^[38], ^j^[36], ^k^[81], ^l^[87], ^m^[47], ^n^[93], ^o^[83], ^p^[91], ^q^[9], ^r^[45], ^s^[81], ^t^[84], ^u^[49], ^v^[94], ^w^[85], ^x^[51], ^y^[80], ^z^[86].

**Fig. 1a–e:**
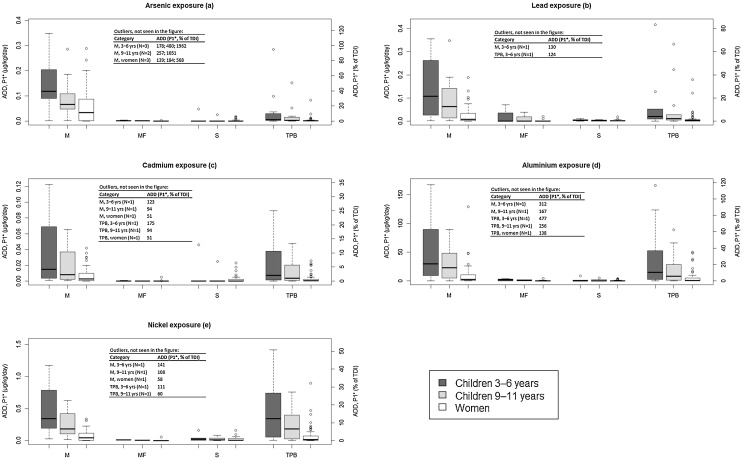
Boxplots showing exposure of the **exclusively toxic elements** after intake of supplements of different origins. M: Marine non-fish oil, MF: Marine fish oil, S: Synthetic and TPB: Terrestrial Plant Based. The upper and lower horizontal lines mark the maximum and minimum value of the dataset, respectively. The line within the box shows the median value, the lower edge of the box the 25^th^ percentile and the upper edge the 75^th^ percentile. *Underweight individuals. To convert the result to normal weight, the ADDs referring to underweight people are divided by 1.35 (3–6 years), 1.53 (9–11 years) and 1.44 (adult women) using the weight ratios specified in the method section.

**Fig. 1f–j:**
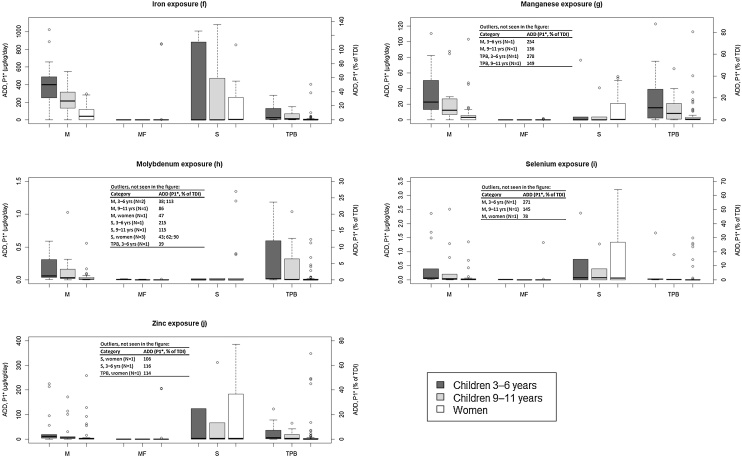
Boxplots showing exposure of **essential elements** after intake of supplements of different origins. M: Marine non-fish oil, MF: Marine fish oil, S: Synthetic and TPB: Terrestrial Plant Based. The upper and lower horizontal lines mark the maximum and minimum value of the dataset, respectively. The line within the box shows the median value, the lower edge of the box the 25^th^ percentile and the upper edge the 75^th^ percentile. *Underweight individuals. To convert the result to normal weight, the ADDs referring to underweight people are divided by 1.35 (3–6 years), 1.53 (9–11 years) and 1.44 (adult women) due to the weight ratios specified in the method section.

##### 3.2.1.1 Non-essential and exclusively toxic elements (As, Pb, Cd, Al, Ni)

Fig. 1a-e show that the highest intakes of the non-essential and toxic elements As, Pb, Cd, Al, Ni are associated with the marine (non-fish oil) products where the most common ingredients are various algae (mainly spirulina or chlorella), mussels etc. (see supplementary Table S1 (A—D) for ingredients in all products) and to a lesser degree with the terrestrial plant-based group. These results affirm that algae are efficient metal accumulators [8,59], and that algae-based food supplements may contain high concentrations of many different metals [22]. In contrast, negligible ADDs for these toxic elements were associated with fish oil supplements as well as synthetic food supplements (Fig. 1a-e). Again, this is in accordance with previously published data. Low concentrations of toxic elements in synthetic supplements have been reported by Avula et al. [57], while low concentrations of Hg, Cu, Zn, Pb, As, Fe and Mn (thus not only non-essential metals) in fish oil have been found in number of studies [60,61,22,62,63]. The proposed explanation for the low concentrations in fish oil is that metals generally bind much more efficiently to proteins, in for example fish flesh, gills or liver tissue, than they accumulate in hydrophobic structures.

The present study, in accordance with previous studies [18,64], found that the intake of As in particular can be significant for consumers of marine (non-fish oil) food supplements (Fig. 1a-e). Within this category, the share of products that gave As ADDs >50 % of TDI was 13 % for women of normal weight (18 % in the case of underweight) and 29 % (or 35 %) for the youngest group of children that was assessed (3–6 years old). For data on individual products, please consult the supplementary information, Table S3.

While all oral TDIs are associated with uncertainties and should not be viewed as marking a distinct border between safe and harmful doses, a critical aspect when assessing oral As exposure is that the current TDI of 0.3 μg/kg/day is based on exposure to inorganic As (iAs). In certain plants and animals (=food), however, iAs can be transformed into a number of organic species. This especially applies to fish and seafood, where organic forms of As often dominate, e.g. as methylarsonic acid (MMA), dimethylarsinic acid (DMA), arsenosugars, arsenolipids, and arsenobetaine (AsB). Generally AsB is by far the most common arsenic species in seafood, especially in finfish and crayfish, and since it is excreted with urine without previous metabolic transformations AsB is considered nontoxic in humans [65,66]. Compared to finfish, however, algae, mussels and other filter feeders usually contain a higher fraction of iAs. In the study by García-Salgado et al. [67], for example, between 8 and 84 % of the total As in a number of different edible algae was inorganic. A broader screening, however, shows a large variability in the iAs concentrations in algae [34]. Additionally, algae show an organic speciation with less AsB and more of the other organoarsenicals than finfish [68,69]. This is important since many of these other organic species are highly toxic, just as iAs [65,69,66]. Therefore, exposure to all kinds of organic As cannot be considered as unconditionally safe. For example, both MMA and DMA are classified as “possibly carcinogenic to humans” (Group 2B) according to the International Agency for Research on Cancer [70]. Although there are large gaps in our knowledge with regards to arsenic transformations in different organisms and along food webs, the review by Thomas and Bradham [71] suggests that algae take up iAs in marine environments. Thereafter parts of the iAs are methylated and transformed into arsenosugars, the latter being essential for the biotransformation into AsB which becomes the dominant form at higher tropic levels. To conclude, an accurate assessment of risks associated with As consumption requires speciation information. Without such information, one can – according to the precaution principle – assume that the majority of the As in the analysed supplements is associated with either iAs or other organic species besides AsB – thus possessing toxic properties of different kinds and magnitudes.

Returning to our own data again, the median As ADDs for normal-weight women and children already correspond to about 8% and 29 % of the As TDI, respectively, when products with marine ingredients are considered. For underweight individuals these figures increase to 11 % and 39 % (Fig. 1a-e). It should be noted that the “tolerable daily intake” for As is not a traditional threshold TDI, i.e. it does not represent a dose that is without risks of negative health effects. Since the critical As toxicity is related to carcinogenicity, for which threshold doses can often not be set, the As TDI rather states the dose that gives a tolerable increase in cancer risk. Among the 38 marine food supplements that were analysed, 5% (for normal-weight women) or 8% (underweight women) even resulted in As ADDs above the TDI. Consumption of the product with the highest As ADD results in an As intake of nearly four times the TDI for normal-weight women and 15 times the TDI for young children (Fig. 1a-e). For people of low bodyweight, the risks are obviously exaggerated, and the maximum ADDs increase to 6 and 20 times the TDI for underweight women and children, respectively.

For the remaining non-essential elements, there are also examples of ADDs that exceed the TDIs, among both the marine (non-fish oil) supplements and the ones of terrestrial plant-based origin (Fig. 1a-e). For Al, the maximum ADD is 477 % of the TDI for underweight children. For Cd, Ni and Pb the corresponding figures are 175 %, 141 % and 130 %. While the risk of high ADDs is admittedly associated with a relatively limited number of the analysed products, the risk of a random product containing high concentrations of these elements is not negligible. Among the marine supplements, for example, we see that 16 % provide an ADD > 50 % of TDI for at least one of the non-essential elements for normal-weight women. It increases to 21 % for underweight women.

Among the marine (non-fish oil) supplements that are labelled suitable for small children as well (aged 3–6 years), as much as 47 % (in the case of normal weight) or 71 % (underweight) of the products showed ADDs > 50 % of the TDIs for at least one of the toxic metals. The share of products with ADDs > TDIs for children were 24 % (normal weight) and 41 % (underweight). For small children (3–6 years old), the ADDs were in many cases also high for supplements belonging to the terrestrial plant-based group. Here 31 % of the analysed supplements showed an intake above 50 % of the TDI for at least one of the toxic metals, for both normal-weight and underweight children. The fraction of TPB supplements that showed an ADD > TDI was 13 % (normal weight) or 25 % (underweight).

##### 3.2.1.2 Essential elements (Fe, Mn, Mo, Se, Zn)

Even for the essential elements, the ADDs can be significant (defined here as >50 % of TDI) after consumption of food supplements with a marine (non-fish oil) or plant-based origin, but now we also see that products from the synthetic group can render high ADDs (Fig. 1f-j). Concentrations and ADDs in the fish oil products were consistently low with the exception of one product to which Fe had been added intentionally. If the fish oil products are left aside, the highest intakes of Mn and Mo are related to the marine and plant-based groups. Among the synthetic mineral supplements, the highest ADDs relative to TDIs are found for Fe, Se and Zn. Since essential elements are vital for a number of physiological functions (Table 3), but cannot be synthesised by the human body, they need to be provided through dietary sources. Consequently, they are often intended constituents, especially in mineral supplements, and high ADDs for these substances are expected. The criterion of an ADD > 50 % of the TDI, which was used for further evaluation in this study, is thus unlikely to be associated with an increase in health risks. Nevertheless, this criterion was fulfilled for 5% of all the analysed supplements (fish oil products excluded) for at least one of the essential elements. In a worst-case scenario, for small and underweight children (3–6 years), the share increases to 39 %. Iron is generally the element that renders the highest ADDs relative TDI, but since lack of Fe is the most common nutritional deficiency globally (Table 3), its addition to food supplements is actually often motivated. Relatively high levels of Fe (in particular), but also of Zn, Se, Mn and Mo, were also found by Korfali et al. [9], who analysed 33 food supplements of mainly synthetic origin.

More relevant for the essential elements is to assess the risk of exceeding TDIs, where negative effects may start to arise (Table 3). For adults such a risk is negligible when considering the intake via food supplements. None of the analysed products gave ADDs > TDI for either Mn, Mo nor Se, even for underweight women. In three of the 129 products (2%) from the marine (non-fish oil), terrestrial plant based and synthetic groups, Fe and Zn ADDs for underweight women were slightly above the TDIs (Fig. 1f-j). Iron intake exceeding recommended upper limits, albeit only in a few supplements, was also found in a study focusing on multi-ingredient food supplements by Poniedziałek et al. [10]. In a worst-case scenario, however, focusing on underweight 3–6 year olds, 7 out of 38 products, or 18 % (marine fish-oil products still excluded), resulted in ADDs > TDIs for Fe, Mn, Mo, Se and/or Zn. In the majority of these, Fe was the element with the highest intake relative to TDI.

### 3.3 Average daily intakes (ADDs) via food supplements in relation to the normal dietary intake

As mentioned already in the section above, high ADDs are associated with a relatively limited number of the analysed products. The majority of the food supplements also give only a marginal addition to the normal dietary intake of the most critical elements identified in this study; As, Pb, Cd, Al, Ni and Fe, Mn, Se, Mo and Zn. This is apparent from the median values for normal weight women in Table 4a and 4b, which demonstrate that the daily intake in most cases increases by only a few percent when food supplements are added to the normal diet. The exceptions are As and Fe, where half of the investigated products from the marine group (the median column again) result in an intake increase of at least 6.6 % and 14 % respectively. For iron, such an increase is uncontroversial. So, focusing on average exposures (ADDs at median level in Table 2) one could argue that the small addition that generally is associated with food supplements is negligible from a health risk perspective. But then, as further seen in Table 4a**+b**, there are several high percentage figures found at the P75, P95 and max level, meaning that there are food supplements that contain concentrations of these 10 elements to an extent that substantially increases the intake relative to what can be expected from a normal diet. Arsenic is the most extreme example, where one product increases the average daily intake by 340 % for a woman of normal weight. But even the other metals can be ingested in high amounts relative to the normal dietary intake, where the “worst-case-products” give ADDs worth a 15–380 % increase. This is of course particularly critical for the non-essential elements. Besides being unwanted dietary constituents with exclusively toxic properties, a normal diet already quite often results in intakes > TDI for these elements (Table 4a). The risk of an excess, and potentially harmful, intake is thus generally high even without food supplements.

**Table 4a:** Normal dietary intake, and the main sources of the toxic elements As, Pb, Cd, Al and Ni in the European population, as well as the percental increase associated with a daily intake of food supplements, based on the median and maximum ADDs derived from the products analysed in this study. For comparison the table also shows tolerable daily intakes (TDIs) for the five elements. Adequate intakes (AIs) are not applicable for this group of exclusively toxic metals. Only the marine (M) and terrestrial plant based (TPB) products are shown in the table since the concentrations of these toxic substances in the other groups are negligible in comparison (Fig. 1**a-e**).

Element	Normal (=average) dietary intake		Intake from food supplements (food supplement ADDs in % of normal dietary intake***)					TDI
			Values ​​in parentheses: underweight individuals					
		Group	Category	Median	P75	P95	Max	
As (inorganic)	0.13–0.56 μg/kg/day	M	Adult women:	6.6 (9.5)	17 (24)	87 (120)	340 (490)	0.3–8 μg/kg/day^a^
			3–6 years:	25 (34)	43 (58)	490 (660)	1200 (1700)	

	Important sources: fish and seafood, algae-based products, cereals (particularly rice), bran and germ, bottled water, vegetables, coffee and beer^a^.	TPB	Adult women:	0.20 (0.28)	0.50 (0.72)	2.2 (3.2)	16 (24)	
3–6 years:			1.2 (1.6)	6.0 (8.1)	31 (41)	60 (81)

Pb	0.36–1.24 μg/kg/day	M	Adult women:	0.67 (1.0)	2.9 (4.2)	9.3 (13)	16 (23)	0.5 μg/kg/day^b^
			3–6 years:	10 (13)	24 (33)	38 (52)	60 (81)	

	Important sources: cereal products, vegetables, tap water^b^.	TPB	Adult women:	0.25 (0.36)	0.55 (0.80)	3.2 (4.6)	16 (22)	
3–6 years:.			1.9 (2.6)	4.8 (6.5)	43 (58)	57 (78)

Cd	0.27–0.43 μg/kg/day	M	Adult women:	0.53 (0.76)	1.9 (2.8)	7.2 (10)	36 (52)	0.36 μg/kg/day^d^
			3–6 years:	3.2 (4.3)	15 (20)	39 (53)	93 (130)	

	Important sources: vegetables grown on land where cadmium-containing fertilisers have been used, seaweed, fish and seafood, chocolate, wild mushrooms^c^.	TPB	Adult women:	0.11 (0.16)	0.43 (0.62)	4.1 (6.0)	36 (52)	
3–6 years:			1.5 (2.0)	7.0 (9.4)	47 (64)	130 (180)

Al	29–214 μg/kg/day	M	Adult women:	1.5 (2.2)	6.1 (8.8)	27 (39)	74 (110)	140 μg/kg/day^e^
			3–6 years:.	18 (24)	55 (74)	140 (180)	270 (370)	

	Important sources: foods rich in sugar (e.g. bread, pastries, beverages), vegetables, dairy products, sausages, seafood, tea leaves, herbs, cocoa, spices^e^.	TPB	Adult women:	0.36 (0.51)	2.7 (3.9)	22 (31)	110 (160)	
3–6 years:			9.1 (12)	26 (35)	180 (240)	410 (560)

Ni	2.0–13.1 μg/kg/day (chronic dietary exposure)	M	Adult women:	0.43 (0.62)	1.0 (1.5)	2.9 (4.2)	15 (21)	2.8 μg/kg/day^f^
			3–6 years:	3.4 (4.5)	7.7 (10)	17 (23)	38 (52)	

	Important sources: cereals, beverages, sugar and confectionery, legumes, nuts, vegetables, mushrooms. Dairy products can be significant sources, especially for young children^f^.	TPB	Adult women:	0.17 (0.24)	0.65 (0.94)	3.7 (5.3)	8.2 (12)	
3–6 years:			3.4 (4.6)	7.1 (10)	18 (24)	30 (41)

^a^[34], ^b^[39], ^c^[58], ^d^[35], ^e^[33], ^f^[38].

**Table 4b:** Normal dietary intake, and the main sources, of the essential elements Fe, Mn, Mo, Se and Zn in the European population, as well as the percental increase associated with a daily intake of food supplements, based on the median, P75, P95 and maximum ADDs derived from the products analysed in this study. For comparison the table also shows tolerable daily intakes (TDIs) and adequate intakes (AIs) for the five essential metals. The marine fish oil (MF) products are not shown in the table since the concentrations in this group are negligible in comparison (Fig. 1**f-j**).

Element	Normal (=average) dietary intake**		Intake from food supplements (food supplement ADDs in % of normal dietary intake***)					TDI	AI*
			Values ​​in parentheses: underweight individuals						
		Group	Category	Median	P75	P95	Max		
Fe	139–264 μg/kg/day	S	Adult women:	1.3 (1.9)	88 (130)	150 (220)	290 (420)	800 μg/kg/day^c^	Women ≥ 18 years: 236/163 μg/kg/day (pre-/postmenopausal)
			3–6 years:	0.52 (0.70)	320 (440)	360 (490)	370 (500)		

	Important sources: liver, game and beef, egg yolks, cereals and pulses^^a,b^^.	M	Adult women:	14 (20)	39 (56)	90 (130)	100 (150)		
			3–6 years:	150 (200)	180 (240)	340 (450)	370 (510)	

TPB		Adult women:	0.36 (0.52)	2.4 (3.5)	15 (21)	140 (200)	Children 1–6 years: 380 μg/kg/day
	3–6 years:	8.9 (12)	48 (65)	66 (89)	100 (140)		

Mn	30–89 μg/kg/day	S	Adult women:	0.39 (0.55)	26 (38)	66 (95)	70 (100)	140 μg/kg/day^e^	Women ≥ 18 years: 44 μg/kg/day
			3–6 years:	0.23 (0.31)	4.8 (6.4)	83 (110)	100 (140)		

	Important sources: cereals, vegetables, fruits, drinks, nuts and chocolate^d^.	M	Adult women:	3.8 (5.5)	7.0 (10)	58 (83)	130 (190)		Children, 4–6 years
			3–6 years:	31 (41)	68 (91)	210 (290)	480 (650)		

TPB		Adult women:	1.0 (1.5)	4.3 (6.2)	33 (47)	140 (200)	54 μg/kg/day
	3–6 years:	21 (28)	51 (69)	250 (340)	520 (710)		

Mo	0.86–2.3 μg/kg/day	S	Adult women:	0.070 (0.10)	0.96 (1.4)	120 (170)	200 (280)	5 μg/kg/day^g^	Women ≥ 18 years: 0.96 μg/kg/day
			3–6 years:	0.035 (0.048)	0.85 (1.1)	400 (540)	500 (680)		

	Important sources: cereals, pulses, offal (liver, kidney) and nuts^f^.	M	Adult women:	0.52 (0.75)	1.7 (2.4)	10 (14)	100 (150)		Children, 4–6 years:
			3–6 years:	2.9 (3.9)	14 (19)	120 (170)	260 (360)		

TPB		Adult women:	0.14 (0.20)	0.44 (0.64)	10 (14)	27 (39)	1.1 μg/kg/day
	3–6 years:	0.72 (1.0)	25 (34)	64 (87)	91 (120)		

Se	0.46–0.97 μg/kg/day	S	Adult women:	5.8 (8.3)	130 (190)	210 (300)	310 (450)	5 μg/kg/day^i^	Women ≥ 18 years: 1.0 μg/kg/day
			3–6 years:	7.3 (9.8)	76 (100)	210 (280)	240 (330)		

	Important sources: milk and dairy products, meat, cereals and fish^h^.	M	Adult women:	0.84 (1.2)	3.1 (4.5)	77 (110)	380 (540)		Children, 4–6 years:
			3–6 years:	7.0 (10)	40 (53)	470 (640)	1400 (1900)		

TPB		Adult women:	0.30 (0.44)	0.75 (1.1)	110 (160)	140 (210)	1.1 μg/kg/day
	3–6 years:	2.4 (3.3)	3.3 (4.5)	46 (62)	170 (230)		

Zn	118–207 μg/kg/day	S	Adult women:	1.1 (1.6)	78 (110)	160 (220)	230 (320)	500 μg/kg/day^l^	Women ≥ 18 years: 111–188 μg/kg/day, depending on level of phytate**** intake.
			3–6 years:	1.4 (1.8)	56 (76)	220 (300)	260 (360)		

	Important sources: meat, eggs, legumes, grains, sea food, water and vegetables^^j,k,l^^.	M	Adult women:	0.59 (0.85)	1.7 (2.4)	42 (60)	110 (160)		Children, 4–6 years:
			3–6 years:	5.4 (7.3)	8.3 (11)	98 (130)	100 (140)		

TPB		Adult women:	0.27 (0.39)	1.3 (1.9)	100 (150)	240 (350)	299 μg/kg/day
	3–6 years:	2.4 (3.3)	14 (19)	40 (55)	56 (75)		

^a^[81], ^b^[87], ^c^[36], ^d^[83], ^e^[47], ^f^[84], ^g^[45], ^h^[85], ^i^[49], ^j^[80], ^k^[86], ^l^[51].

*Based on Summary of Dietary Reference Values [88] originally expressed in mg/day or μg/day. These are all converted to μg/kg/day by division by 67.7 kg (normal-weight women) or 18.4 kg (normal-weight children, an approximate value since this value refers to children 3–6 years).

**The average dietary intake in the original sources were given only in mg/day. For Fe 9.4–17.9 mg/day [87], for Mn 2–6 mg/day [83], for Mo 58–157 μg/day [84], for Se 31.0–65.6 μg/day [85], and for Zn 8.0–14.0 mg/day [86]. The conversion into intake intervals per kilo bodyweight were done by dividing all figures with the mean weight for adult women (67.7 kg).

***The % intake relative to the normal diet is estimated by comparing the median and maximum ADDs for the analysed food supplements with mean value of the normal dietary intake intervals (for Fe, for example: (139 + 264)/2 = 202).

****Phytate inhibits the absorption of zinc [86].

## 4 Conclusions and final remarks

In summary, most of the investigated food supplements are safe for consumers in recommended doses, at least when the inorganic elements investigated here are concerned. There are, nevertheless, still a non-negligible number of products out there where regular consumption is associated with high average daily doses (ADDs) of different elements. This applies both in relation to the tolerable daily intake (TDI), i.e. the maximum intake or dose that is overall assumed acceptable before the onset of negative health effects, and in relation to normal dietary intakes, i.e. the amounts that are ingested via normal food and drinking water.

Among essential elements, the highest ADDs relative to TDI were found for Fe, Mn, Mo, Se and Zn. Increasing the intake of these elements through food supplements should, however, generally not be interpreted as an imminent health risk. Firstly, because these elements are required in balanced amounts to counteract physiological deficiencies, and secondly, because their ADDs seldom exceed the TDIs, at least for adults. For small children, an important finding is that a significant fraction of the products results in ADDs > TDIs if the recommended dosage is administered. A problem may also arise when concentrations deviate from those declared. The existence of considerable deviations between the actual and declared content of elements in food supplements has been reported previously [7,10,[72], [73], [74]], and in the present study the found concentrations were between 50 % and 150 % of the declared, for the majority of supplements. These deviations are probably, at least for “natural products”, to some degree due to differences between batches, which was something not addressed in this study. In addition, the table of contents (TOCs) state only wanted constituents. Out of the 71 elements analysed in this study only 22 were found on *any* product’s TOC, as shown in the supplementary material, Table S1 (B, Br, Ca, Co, Cr, Cu, Fe, I, K, Mg, Mn, Mo, Na, Ni, P, S, Se, Si, Sr, Ti, V and Zn). Hence, it is in many cases difficult for consumers, food inspection authorities etc., to assess the intake even of wanted constituents.

A major finding of the present study was the relatively high concentrations of unwanted toxic elements, or contaminants, in several products of marine and terrestrial plant-based origin. As concentrations of elements in food supplements appear to be extremely variable, results from any given study will be strongly influenced by the product selection. In the present study high intakes relative to TDI were found for Cd, Pb, As, Ni and Al. For all these purely toxic elements a normal diet already often results in intakes > TDIs. It is therefore particularly important to identify dietary sources that can be key contributors of these elements. In our study we found that 16 % of the products with marine ingredients (algae, mussels etc.) gave an ADD > 50 % of TDI for at least one of the toxic elements for normal-weight women. The fraction increased to 21 % for underweight women and to 71 % for young underweight children (3–6 years old). If the product linked to the highest As ADD was given to underweight women or children, the intake would equal 6 times the TDI for women and 20 times the TDI for children.

Based on our (and other researchers’) findings that food supplements can contain certain hazardous metals to a degree that significantly add to the normal dietary intake, and given that concentrations of these metals may vary more in food supplements than in other types of food, there is thus every reason to revise both the regulation and control of food supplements, forcing producers to analyse the content of toxic metals that may otherwise appear in harmful concentrations in their products. One way is to set MPCs ​​for additional elements, and not only for Cd, Pb and Hg, as also pointed out by Rzymski et al. [18] and Hedegaard et al. [75]. According to our results, the elements with MPCs according to the EU commission (Cd, Pb, Hg) do not match the toxic elements that most frequently appear in high concentrations relative to available TDIs, thus probably constituting the most prominent risk (Cd, Pb, Al, As, Ni and possibly Co). Here it should be noted that our risk assessment was restricted to the elements with available TDIs. Almost half, or 32 out of the 71 elements, lack TDIs. One cannot rule out that some, or several, of these elements can also appear in food supplements to a degree that is unhealthy.

One factor that may increase the exposure to toxic elements via food supplements drastically, and which thus further exaggerates the need of stricter control, is the fact that many consumers use more than one food supplement products simultaneously. That the use of multiple food supplements is common has been reported repeatedly in recent years [[76], [77], [78]]. A US cross-sectional survey, for example, showed that about 10 % of food supplement consumers used as many as 5 different products or more [79]. Yet another factor that stresses the need for more rigorous control is the association between demographic factors and food supplement use, where many particularly sensitive individuals are found among the food supplement users. Bailey et al. show, among others, that lower-weight people have an increased tendency to consume food supplements; a fact that also increases ADDs since these are given per kilo bodyweight. In addition, women (who weigh less than men) are more likely to use food supplements [7,12], and an increased use can be seen among children [11].

While the majority of food supplements contain harmless concentrations of elements, the purchase of a random, untested supplement is associated with a non-negligible risk of being exposed to harmful doses of various toxic elements – a risk that increases with the number of products consumed simultaneously and with decreasing body weight. Legislative and control frameworks have to acknowledge this risk and adapt as to ensure that no “high-risk-products” contaminated with toxic elements reach consumers.

## Author statement

All persons who meet authorship criteria are listed as authors, and all authors certify that they have participated sufficiently in the work to take public responsibility for the content, including participation in the concept, design, analysis, writing, or revision of the manuscript. Furthermore, each author certifies that this material or similar material has not been and will not be submitted to or published in any other publication.

## Author contributions

A. Augustsson, I. Rodushkin and E. Engström designed the study. I. Rodushkin and C. Paulukat did the chemical analyses. A. Augustsson and A. Qvarforth analysed the data and took equal part in writing the manuscript. All authors have participated in discussing the results and commented on the manuscript text.

## Declaration of Competing Interest

The authors report no declarations of interest.
