# Supplementary material for: Trace and major elements in food supplements of different origin: Implications for daily intake levels and health risks
Source: Toxicol Rep. 2021 May 2;8:1067–80. doi: 10.1016/j.toxrep.2021.04.012 (PMC8166911; doi:10.1016/j.toxrep.2021.04.012)
Supplement: Supplementary file 1 [file mmc1.docx]

**Supplementary material**

**Trace and major elements in food supplements of different origin: implications for daily intake levels and health risks**

Augustsson A, Qvarforth A, Engström E, Paulukat C, Rodushkin I.

**Table S1.A** Declared content in the 29 analysed synthetic vitamin- and/or mineral food supplements (S01-S29). The weight per tablet/capsule refers to the table/capsule that was digested and analysed for this project, thus the exact number of digits.

| **Product** | **Main ingredients** | **Specified content of minerals and vitamins** | **Weight per tablet/capsule (mg)** | **Daily dose** | |
| --- | --- | --- | --- | --- | --- |
| S01 | Vitamin D | 1 tablet: vit. D = 12.5 µg | 208 | Adults: | 1 tablet |
| S02 | Magnesium | 1 tablet: Mg = 250 mg | 792 | Adults: | 1 tablet |
| S03 | Vitamin B | 1 tablet: vit. B1 = 15 mg; vit. B6 = 15 mg; vit. B2 = 15 mg; vit. B12 = 5 µg; vit. B3 = 60 mg; vit. B5 = 30 mg; vit. B9 = 200 µg | 400 | Adults: | 1 tablet |
| S04 | Calcium, vitamin D | 1 tablet: Ca = 500 mg; vit. D = 5 µg | 1493 | Adults: | 1–2 tablets |
| S05 | Magnesium | 1 tablet: Mg = 250 mg | 2020 | Adults: | 1 tablet |
| S06 | Vitamins and minerals | 1 tablet: Mg = 85 mg; Fe = 14 mg; Cu = 1000 µg; Zn = 10 mg; Mn = 2 mg; Se = 55 µg; Cr = 40 µg; Mo = 50 µg; vit. A = 800 µg; vit. D = 20 µg; vit. E = 12 mg; vit. B1 = 1.1 mg; vit. B2 = 1.4 mg; vit. B3 = 16 mg; vit. B5 = 6 mg; vit. B6 = 1.4 µg. vit. B9 = 200 µg; vit. B12 = 2.5 µg; vit. C = 80 mg | 624 | Adults: | 1 tablet |
| S07 | Iron | 1 tablet: Fe = 14 mg | 778 | Pregnant:  Women:  (menstruating)  Other: | 2–3 tablets  1–2 tablets  1 tablet |
| S08 | Vitamin D | 1 tablet: vit. D = 10 µg | 345 | Adults: | 1 tablet |
| S09 | Vitamins and minerals | 1 tablet: Ca = 200 mg; Mg = 56 mg; Fe = 18 mg; Zn = 9 mg; Cu = 1000 µg; Se = 60 µg; vit. A = 800 μg; vit. D = 10 μg; vit. E = 10 mg; vit. C = 85 mg; vit. B1 = 1.5 mg; vit. B2 = 1.6 mg; vit. B3 = 17 mg; vit. B6 = 1.4 mg; vit. B9 = 500 μg; vit. B12 = 2 μg; vit. B7 = 30 μg; vit. B5 = 6 mg | 1074 | Adults: | 1 tablet |
| S10 | Vitamins and minerals | 1 tablet: Ca = 120 mg; Mg = 60 mg; Fe = 7.5 mg; Zn = 3.5 mg; Cu = 0.45 mg; Mn = 1 mg; Se = 20 µg; Cr = 12.5 µg; Mo = 22.5 µg; vit. A = 350 µg; vit. D3 = 3.8 µg; vit. E = 15 mg; vit. K = 45 µg; vitamin B1 = 0.55 mg; vit. B2 = 0.65 mg; vit. B3 = 7.5 mg; vit. B5 = 2.5 mg; vit. B6 = 0.65 mg; vit. B9 = 200 µg; vit. B12 = 1 µg; vit. B7 = 15 µg; vit. C = 60 mg | 741 | Adults: | 2 tablets |
| S11 | Zinc | 1 tablet: Zn = 6.8 mg | 596 | Adults: | 3 tablets |
| S12 | Vitamins and minerals | 1 tablet: Fe = 10 mg; Zn = 9 mg; Cu = 0.45 mg; Mn = 1 mg; Cr = 75 µg; Se = 50 µg; Mo = 75 µg; Ca = 65 mg; Mg = 125 mg; vit. A = 400 µg; vit. D = 7.5 µg; vit. E = 30 mg; vit. B1 = 1.75 mg; vit. B2 = 2 mg; vit. B3 = 22.5 mg; vit. B6 = 1.9 mg; vit. B9 = 150 µg; vit. B12 = 2 µg; vit. C = 100 mg; vit. B7 = 45 µg; vit. B5 = 3 mg | 1066 | Adults: | 1–2 tablets |
| S13 | Salts, minerals, vitamin D | 1 capsule: Ca = 14 mg; Mg = 7 mg; Na = 190 mg; K = 53 mg; vit. D = 2.5 µg | 1296 | Adults: | Up to 15 capsules |
| S14 | Vitamins and minerals | 1 capsule: Mg = 100 mg; Fe = 14 mg; Zn = 10 mg; Mn = 2 mg; Cu = 1000 µg; Se = 55 µg; Cr = 40 µg; vit. E = 3 mg; vit. A = 250 µg; vit. D = 5 µg; vit. B7 = 50 µg; vit. B9 = 200 µg; vit. B3 = 16 mg; vit. B5 = 6 mg; vit. B1 = 1.1 mg; vit. B2 = 1.4 mg; vit. B6 = 1.4 mg; vit. B12 = 5 µg; vit. C = 40 mg | 747 | Adults: | 1 capsule |
| S15 | Vitamins and minerals | 1 tablet: Ca = 200 mg; Mg = 94 mg; Fe = 3.5 mg; Zn = 10 mg; Cu = 0.25 mg; Mn = 0.5 mg; Se = 55 µg; Cr = 10 µg; Mo = 13 µg; vit. A = 200 µg; vit. D = 5 µg; vit. E = 12 mg; vit. K = 19 µg; vit. C = 80 mg; vit. B1 = 1.1 mg; vit. B2 = 1.4 mg; vit. B3 = 4 mg; vit. B6 = 1.4 mg; vit. B9 = 200 µg; vit. B12 = 2.5 µg | 4561 | Adults: | 1 effervescent tablet |
| S16 | Vitamin C | 1 tablet: vit. C = 1000 mg | 4150 | Adults: | 1 effervescent tablet |
| S17 | Vitamin C | 1 tablet: vit. C = 1000 mg | 4091 | Adults: | 1 effervescent tablet |
| S18 | Vitamins and minerals | 1 tablet: Ca = 100 mg; Mg = 38 mg; Fe = 7.5 mg; Cu = 450 µg; Zn = 3.5 mg; Mn = 1 mg; Se = 25 µg; Cr = 20 µg; Mo = 25 µg; I = 75 µg; vit. A = 700 µg; vit. D3 = 5 µg; vit. K1 = 38 µg; vit. B1 = 0.55 mg; vit. B2 = 0.65 mg; vit. B3 = 7.5 mg; vit. B5 = 3 mg; vit. B6 = 0.65 mg; vit. B7 = 25 µg; vit. B9 = 200 µg; vit. B12 = 1 µg; vit. C = 38 mg; vit. E = 4 mg | 4119 | Adults: | 2 effervescent tablets |
| S19 | Vitamins and minerals | 1 tablet: Zn = 7 mg; Cu = 0.45 mg; Mn = 0.5 mg; Cr = 25 µg; Se = 30 µg; Mo = 50 µg; Ca = 120 mg; Mg = 58 mg; vit. A = 250 µg; vit. D3 = 7.5 µg; vit. E = 10 mg; vit. B1 = 0.7 mg; vit. B2 = 0.8 mg; vit. B3 = 9 mg; vit. B6 = 0.8 mg; vit. B9 = 150 µg; vit. B12 = 1 µg; vit. C = 60 mg; vit. B7 = 15 µg; vit. B5 = 2.5 mg | 803 | Adults: | 1–2 tablets |
| S20 | Zinc | 1 tablet: Zn = 30 mg | 347 | Adults: | 1 tablet |
| S21 | Vitamin D | 1 tablet: vit. D = 100 µg | 198 | Adults: | 1 tablet |
| S22 | Selenium, vitamin E | 1 tablet: Se = 55 µg; vit. E = 12 mg | 330 | Adults: | 1 tablet |
| S23 | Vitamins and minerals | 1 tablet: Fe = 4.5 mg; Zn = 3.5 mg; Mn = 0.5 mg; Cr = 25 µg; Se = 12.5 µg; Mo = 50 µg; Ca = 80 mg; Mg = 20 mg; vit. A = 175 µg; vit. D3 = 3.75 µg; vit. E = 3 mg; vit. B1 = 0.35 mg; vit. B2 = 0.45 mg; vit. B3 = 5.5 mg; vit. B6 = 0.45 mg; vit. B9 = 62.5 µg; vit. B12 = 0.55 µg; vit. C = 20 mg; vit. B7 = 10 µg; vit. B5 = 2.5 mg | 1033 | Adults:  Children^*^: | 1–2 tablets  1–2 tablets |
| S24 | Vitamins | 1 tablet: vit. A = 320 µg; vit. B3 = 8 mg; vit. B5 = 1.2 mg; vit. B6 = 0.7 mg; vit. B12 = 2.5 µg; vit. B7 = 25 µg; vit. B9 = 100 µg; vit. C = 40 mg; vit. D: 10 µg; vit. E = 6 mg | 1937 | Adults:  Children: | 1 chewable tablet  1 chewable tablet |
| S25 | Vitamins | 1 tablet: vit. A = 200 µg; vit. D3 = 5 µg; vit. E = 6 mg; vit. B7 = 25 µg; vit. B9 = 100 µg; vit. B3 = 8 mg; vit. B12 = 1.25 µg; vit. B6 = 0.7 mg; vit. C = 30 mg | 1934 | Adults:  Children: | 1–2 chewable tablets  1–2 chewable tablets |
| S26 | Vitamins and minerals | 1 tablet: Mg = 28 mg; Zn = 0.75 mg; Se = 4.5 µg; Cr = 3 µg; Cu = 75 µg; vit. A = 13 µg; vit. B3 = 4 mg; vit. B5 = 0.6 mg; vit. B6 = 0.35 mg; vit. B12 = 0.63 µg; vit. B7 = 13 µg; vit. B9 = 40 µg; vit. D = 2.5 µg | 2054 | Adults:  Children: | 2 chewable tablets  2 chewable tablets |
| S27 | Selenium | 1 capsule: Se = 200 µg | 476 | Adults: | 1 capsule |
| S28 | Vitamin E | 1 capsule: vit. E = 268 mg | 572 | Adults: | 1 capsule |
| S29 | Vitamins and minerals | 30 ml: Ca = 160 mg; Mg = 225 mg; Fe = 10 mg; Zn = 15 mg; Cu = 1500 mg; Mn = 2.8 mg; Se = 110 µg; Cr = 250 µg; vit. B7 = 200 µg; vit. B9 = 800 µg; vit. B3 = 32 mg; vit. B5 = 30 mg; vit. B2 = 4 mg; vit. B1 = 4 mg; vit. A = 2000 µg; vit. B12 = 10 µg; vit. B6 = 5 mg; vit. C = 400 mg; vit. D = 20 µg; vit. E = 60 mg | - | Adults: | 30 ml oral solution |

* For products where a recommended dose for children is provided, the minimum age is often 3 years. This complies with the youngest age in this study.

**Table S1.B** Declared content in the 38 analysed marine non-fish-oil supplements (M01-M38).

| **Product** | **Main ingredients** | **Specified content of minerals and vitamins** | **Weight per tablet/capsule (mg)** | **Daily dose** | |
| --- | --- | --- | --- | --- | --- |
| M01 | Green lipped mussel, vitamin C, turmeric, etc. | 1 tablet: chondroitin sulphate = 100 mg; turmeric extract = 105 mg; green-lipped mussel = 50 mg; vit. C = 60 mg; MSM (methylsulfonylmethane) = 50 mg; ginger extract = 50 mg | 756 | Adults: | 4 tablets |
| M02 | Algae (spirulina) | 5 g powder: P = 45 mg; Mg = 7.5 mg; Fe = 1.5 mg; K = 60 mg; Zn = 0.8 mg; vit. B1 = 0.1 mg; vit. B2 = 0.2 mg; vit. B3 = 0.4 mg; vit. B6 = 0.04 mg; vit. B12 = 15 µg; vit. B9 = 2.5 µg; vit. K = 45 µg | - | Adults:  Children: | 5–10 g powder  5–10 g powder |
| M03 | Algae (spirulina, chlorella), broccoli, wheatgrass etc. | n/a | - | Adults:  Children: | 3.5–7 g powder  3.5–7 g powder |
| M04 | Algae (spirulina) | 1 tablet: Fe = 11 mg; Cr = 38.5 µg; vit. A = 715 µg; vit. B1 = 0.55 mg | 407 | Adults: | 6–12 tablets |
| M05 | Algae (spirulina), seaweed | 1 tablet: Ca = 120 mg; S = 4.2 mg; P = 1.6 mg; Mg = 1.9 mg; Na = 1.2 mg; K = 2.2 mg; Fe = 0.3 mg; I = 0.075 mg; Si = 0.035 mg; B = 0.02 mg; Zn = 0.02 mg; Mn = 0.01 mg; Cu = 0.0025 mg; Co = 0.001 mg; Ti = 0.001 mg; V = 0.001 mg; Ni = 0.0005 mg; Mo = 0.0001 mg; Br = 0.002 mg; Sr = 0.0002 mg; vit. A = 118 μg; vit. B1 = 1.4 μg; vit. B2 = 2.6 μg; vit. B3 = 31 μg; vit. B5 = 0.5 μg; vit. B6 = 0.12 μg; vit. B7 = 0.13 μg; vit. B9 = 0.15 μg; vit. C = 170 μg; vit. D = 0.1 μg; vit. E = 110 μg; vit. K = 2 μg | 573 | Adults: | 2 tablets |
| M06 | Algae (kelp) | 1 tablet: I = 225 µg | 244 | Adults: | 1 tablet |
| M07 | Algae (kelp) | 1 tablet: I = 50 µg | 249 | Adults: | 4 tablets |
| M08 | Algae (chlorella) | 1 tablet: Fe = 14 mg; Ca = 22 mg; Mg = 40 mg; Na = 17; K = 120 mg; P = 0.29 g; Zn = 0.46 mg; Cr = 0.032 mg; Cu = 100 µg; Mn = 0.92 mg; vit. B1 = 0.2 mg; vit. B2 = 0.8 mg; vit. B3 = 3 mg; vit. B5 = 0.86 mg; vit. B6 = 0.2 mg; vit. B7 = 42 µg; vit. B9 = 0.48 mg; vit. B12 = 0.002 mg; vit. C = 6 mg; carotenoids = 8 mg; lutein = 101 mg; vit. E = 1.4 mg; vit. D = 0.19 µg | 405 | Adults: | 10 tablets |
| M09 | Algae (kelp), horsetail, stinging nettles, propolis | 1 tablet: Ca = 43 mg; P = 35 mg; Si = 10 mg; Zn = 3.7 mg; Fe = 3.3 mg; vit. B7 = 200 µg; vit. B3 = 12 mg; vit. B5 = 1.3 mg; vit. A = 20 µg | 443 | Adults: | 3 tablets |
| M10 | Oyster shell, beetroot powder, vitamins, minerals etc. | 1 tablet: vit. A = 399 µg; vit. D3 = 15 µg; vit. E = 10 mg; vit. C = 75 mg; vit. B1 = 2.5 mg; vit. B2 = 2.5 mg; vit. B3 = 7.5 mg; vit. B6 = 2.5 mg; vit. B9 = 200 µg. vit. B12 = 5 µg; vit. B7 = 51 µg; vit. B5 = 7.5 mg; Ca = 20 mg; Mg = 10 mg; Fe = 6 mg; Zn = 1 mg; Mn = 1 mg; I = 70 µg; Si = 6 mg | 1293 | Adults:  Children 7–12 yrs:  Children 3–6 yrs: | 2 chewable tablets  2 chewable tablets  1 chewable tablet |
| M11 | Green lipped mussel, turmeric, rosehip etc. | 1 tablet: vit. C = 10 mg; Zn = 1.3 mg | 1021 | Adults: | Up to 4 tablets |
| M12 | Algae (kelp) | 1 tablet: I = 85 µg | 399 | Adults: | 1–2 tablets |
| M13 | Algae (spirulina) | n/a | 726 | Adults: | 6 capsules |
| M14 | Algae (chlorella) | 1 capsule: Fe = 0.4 mg; I = 3.75 µg; vit. A = 280 µg; vit. B12 = 0.2 µg | 476 | Adults: | 1–12 capsules |
| M15 | Algae (wakame), chili fruit, green tea,  dandelion, beta glucans, caffeine etc. | n/a | 546 | Adults: | 2 capsules |
| M16 | Algae (spirulina), beetroot, blackberries, spinach, broccoli, vitamins, minerals etc. | 1 capsule: Ca = 25 mg; Mg = 12 mg; Fe = 2.5 mg; Zn = 1.5 mg; Mn = 250 µg; Cu = 150 µg; Cr = 5 µg; Mo = 1.25 µg; I = 37.5 µg; Se = 10 µg; vit. C = 25 mg; vit. B3 = 4 mg; vit. E = 2.5 mg; vit. B5 = 2.5 mg; vit. B2 = 0.5 mg; vit. B6 = 0.5 mg; vit. B1 = 400 µg; vit. A = 200 µg; vit. B9 = 100 µg; vit. K1 = 5 µg; vit. D3 = 2.5 µg; vit. B12 = 1 µg | 477 | Adults:  Children: | 2 capsules  2 capsules |
| M17 | Astaxanthin from algae | 1 capsule: vit. C = 40 mg; vit. E = 3 mg | 575 | Adults: | 1 capsule |
| M18 | Algae oil | 1 capsule: algae oil DHA concentrate 333 mg (omega-3 fatty acids 250 mg, DHA 250 mg) | 537 | Adults: | 1 capsule |
| M19 | Algae oil | 1 capsule: omega-3 fatty acids 375 mg (DHA 170 mg, EPA 560 mg) | 1190 | Adults: | 1–2 capsules |
| M20 | Algae oil | 1 capsule: algae oil (not further specified); Mg = 75 mg; vit. D = 10 µg; vit. C = 70 mg | 883 | Adults: | 1 capsule |
| M21 | Algae (spirulina) | 100 g powder: Ca = 1 g; P = 0.8 g; Mg = 0.4 g; Fe = 104 mg; Na = 0.9 g; K = 1.4 g; Zn = 3 mg; Cu = 1.2 mg; Mn = 5 mg; Cr = 250 µg; Se = 100 µg; vit. A = 40 mg; vit. E = 10 mg; vit. B1 = 3.5 mg; vit. B2 = 2.8 mg; vit. B3 = 14 mg; vit. B6 = 800 µg; vit. B12 = 240 µg; vit. B9 = 10 µg; vit. B7 = 5 µg; vit. B5 = 100 µg | - | Adults:  Children: | 2.5–5 g (1-2 tsp.) powder  2.5–5 g (1-2 tsp.) powder |
| M22 | Algae (spirulina) | n/a | - | Adults:  Children: | 1 tsp. powder  1 tsp. powder |
| M23 | Algae (spirulina) | 100 g powder: Ca = 333 mg; Mg = 300 mg; Fe = 6.6 mg | - | Adults:  Children: | 1–2 tsp. powder  1–2 tsp. powder |
| M24 | Algae (spirulina) | 100 g powder: Na = 0.30 g; K = 1040 mg; Ca = 270 mg; vit. E = 13 mg; vit. B1 = 4.4 mg; vit. B2 = 6.9 mg; vit. B3 = 5.9 mg; vit. B6 = 18.4 mg | - | Adults:  Children: | 5 g powder  5 g powder |
| M25 | Algae (spirulina) | n/a | 510 | Adults: | 3–10 tablets |
| M26 | Algae (spirulina) | n/a | - | Adults:  Children: | 0.5–1 tsp. powder  0.5–1 tsp. powder |
| M27 | Algae (chlorella), wheatgrass, ginger | 100 g powder: Fe = 66 mg; vit. E = 12 mg | - | Adults:  Children: | 1–6 tsp. powder  1–6 tsp. powder |
| M28 | Algae (chlorella) | 100 g powder: Fe = 120 mg; vit. E = 18 mg | - | Adults:  Children: | 4–8 g powder  4–8 g powder |
| M29 | Algae (chlorella) | n/a | - | Adults:  Children: | 0.5–1 tsp. powder  0.5–1 tsp. powder |
| M30 | Algae (spirulina, chlorella), wheatgrass, spinach, acai, alpha-alpha sprouts, green tea extract, coenzyme Q10 etc. | n/a | - | Adults:  Children: | 5 g powder  5 g powder |
| M31 | Algae (spirulina, chlorella), wheatgrass powder, cocoa powder, barley grass powder, kale powder, flaxseed powder, chia seed flour, goji berry powder, broccoli powder, sunflower lecithin, spinach powder, acai, green tea etc. | n/a | - | Adults:  Children: | 5–10 g powder  5–10 g powder |
| M32 | Algae (kelp), vitamins, minerals etc. | 100 g powder: K = 700 mg; Ca = 260 mg; P = 364 mg; Mg = 90 mg; Fe = 8 mg; Zn = 3.6 mg; Cu = 0.26 mg; Se = 20 µg; Cr = 8 µg; Mo = 33 µg; vit. A = 180 µg; vit. D = 2 µg; vit. E = 3 mg; vit. K1 = 2 µg; vit. K2 = 33 µg; vit. C = 60 mg; vit. B1 = 0.24 mg; vit. B2 = 0.28 mg; vit. B3 = 3.2 mg; vit. B6 = 0.34 mg; vit. B9 = 80 µg; vit. B12 = 0.80 µg; vit. B7 = 10 µg; vit. B5 = 1.2 mg | - | Adults:  Children 3–6 yrs: | 100 g powder  70 g powder |
| M33 | Algae (wakame) | n/a | - | Adults:  Children: | Up to 2 g powder  Up to 2 g powder |
| M34 | Green lipped mussel (750 mg) | n/a | 480 | Adults: | 1–2 capsules |
| M35 | Marine collagen (1170 mg) | n/a | 721 | Adults: | 2 capsules |
| M36 | Marine collagen | n/a | - | Adults: | 1–3 tsp. powder |
| M37 | Glucosamine sulphate (from shellfish), 1000 mg | n/a | 1178 | Adults: | 1 tablet |
| M38 | Oil from squid, Indian salaki tree, lutein, algae (*Haematococcus pluvialis*) etc. | 1 capsule: Se = 80 µg; vit. B12 = 2.5 µg; vit. C = 120 mg; vit. D = 15 µg; vit. E = 12 mg | 984 | Adults:  Children: | 1–2 capsules  1–2 capsules |

**Table S1.C** Declared content in the 9 analysed marine fish-oil supplements (MF01-MF09).

| **Product** | **Main ingredients** | **Specified content of minerals and vitamins** | **Weight per tablet/capsule (mg)** | **Daily dose** | |
| --- | --- | --- | --- | --- | --- |
| MF01 | Fish-oil, vitamin E | 1 capsule: fish-oil = 1000 mg (EPA = 330 mg, DHA =220 mg, remaining omega-3 fatty acids = 50 mg); vit. E = 2 mg | 1405 | Adults: | 1–2 capsules |
| MF02 | Fish-oil | 1 capsule: omega-3 fatty acids = 400 mg (EPA = 200 mg, DHA = 150 mg) | 1349 | Adults: | 2–3 capsules |
| MF03 | Fish-oil | 1 capsule: fish-oil = 1000 mg (EPA = 160 mg, DHA =100 mg, remaining omega-3 fatty acids = 300 mg) | 1370 | Adults: | 3 capsules |
| MF04 | Fish-oil, vitamins, minerals | 1 capsule: omega-3 fatty acids = 290 mg (EPA = 29 mg, DHA = 145 mg); Fe = 30 mg; I = 175 μg; Ca = 120 mg; Mg = 57 mg; Zn = 9 mg; Cu = 1000 μg; Se = 60 μg; vit. D3 = 15 μg; vit. B9 = 400 μg; vit. A = 333 μg; vit. B1 = 1.5 mg; vit. B2 = 1.6 mg; vit. B3 = 17 mg; vit. B5 = 6 mg; vit. B6 = 1.5 mg; vit. B7 = 45 μg; vit. B12 = 4 μg; vit. E = 15 mg | 1250 | Adults: | 1 capsule |
| MF05 | Fish-oil | 1 tablet: fish-oil = 90 mg (EPA = 5 mg, DHA = 20 mg, remaining omega-3 fatty acids = 25 mg) | 2618 | Adults:  Children: | 2–4 chewable tablets  2–4 chewable tablets |
| MF06 | Fish-oil, vitamin E | 1 capsule: fish-oil = 1000 mg (EPA = 180 mg; DHA = 120 mg); vit. E = 3 mg | 1383 | Adults: | 4–6 capsules |
| MF07 | Fish-oil, vitamins | 5 ml: omega-3 = 1.2 g (EPA = 0.4 g; DHA = 0.5 g); vit. D = 10 µg; vit. A = 3 mg; vit. E = 10 mg | - | Adults:  Children: | 5 ml oil  5 ml oil |
| MF08 | Fish-oil, vitamins | 5 ml: 1200 mg omega-3 (EPA = 460 mg, DHA = 280 mg); omega-6 = 510 mg; omega-9 = 1500 mg; vit. E = 6.7 mg; vit. D = 5 µg | - | Adults:  Children 3–6: | 5 ml oil  5 ml oil |
| MF09 | Fish-oil | 1 tablet: fish-oil = 114 mg (EPA = 5 mg, DHA = 25 mg) | 2131 | Adults:  Children 3–6: | 2–4 chewable tablets  2–4 chewable tablets |

**Table S1.D** Declared content in the 62 analysed terrestrial plant-based supplements (TPB01-TPB62).

| **Product** | **Main ingredients** | **Specified content of minerals and vitamins** | **Weight per tablet/capsule (mg)** | **Daily dose** | |
| --- | --- | --- | --- | --- | --- |
| TPB01 | Green coffee beans, glucomannan, vitamin B12 | n/a | 1006 | Adults: | 6 tablets |
| TPB02 | Rhubarb, tamarind, figs | n/a | 892 | Adults: | 1 tablet |
| TPB03 | *C. canephora robusta* (coffee beans) | n/a | 1021 | Adults: | 6 tablets |
| TPB04 | Raspberry ketones, green coffee/tea, black pepper extract | 1 tablet: Se = 13 µg; Cr = 13 µg; Zn = 2.5 mg | 342 | Adults: | 4 tablets |
| TPB05 | *Gynostemma pentaphyllum*, citrus flavonoid hesperidin | 1 tablet: Ca = 130 mg | 1592 | Adults: | 1 tablet |
| TPB06 | Arctic root | 1 tablet: Cu = 250 mg; Mn = 0.5 mg; vit. B1 = 0.3 mg; vit. B3 = 4 mg; vit. B7 = 12.5 µg, vit. B5 = 1.5 mg | 721 | Adults: | 1 tablet |
| TPB07 | Green tea | n/a | 1192 | Adults: | 2 tablets |
| TPB08 | Green tea | n/a | 872 | Adults: | 1 tablet |
| TPB09 | Ginseng root, vitamins, minerals | 1 tablet: Mg = 225 mg; Fe = 14 mg; Zn = 10 mg; Cu = 1000 µg; Se = 55 µg; Cr = 40 µg; I = 150 µg; Mo = 50 µg; Mn = 2 mg; vit. A = 800 µg; vit. D = 10 µg; vit. E = 12 mg; vit. B1 = 1.1 mg; vit. B2 = 1.4 mg; vit. B3 = 16 mg; vit. B5 = 6 mg; vit. B6 = 1.4 mg; vit. B9 = 200 µg; vit. B12 = 2.5 µg; vit. B7 = 50 µg; vit. C = 80 mg | 1170 | Adults: | 1 tablet |
| TPB10 | Cranberries | n/a | 464 | Adults: | 1 capsule |
| TPB11 | Cranberries | n/a | 444 | Adults: | 1 capsule |
| TPB12 | Rose hip | n/a | 860 | Adults: | 4–6 capsules |
| TPB13 | Blueberries, marigold | n/a | 417 | Adults: | 2 capsules |
| TPB14 | Green coffee | n/a | 855 | Adults: | 1–2 capsules |
| TPB15 | Rose hip | 1 capsule: vit. C = 27 mg | 890 | Adults: | 3 capsules |
| TPB16 | Turmeric curcumin | n/a | 627 | Adults: | 1–2 capsules |
| TPB17 | Green tea | n/a | 649 | Adults: | 1 capsule |
| TPB18 | Green tea | n/a | 776 | Adults: | 4 capsules |
| TPB19 | Turmeric | n/a | 839 | Adults: | 2 capsules |
| TPB20 | Sesame seeds | n/a | - | Adults:  Children: | 30 g seeds  30 g seeds |
| TPB21 | Turmeric | n/a | - | Adults:  Children: | 1 heaped tbsp. powder  1 heaped tbsp. powder |
| TPB22 | Turmeric | n/a | - | Adults:  Children: | 1 heaped tbsp. powder  1 heaped tbsp. powder |
| TPB23 | Maca superfruit | 100 g: Fe = 9.9 mg; K =1670 mg | - | Adults:  Children: | 16 g powder  16 g powder |
| TPB24 | Stevia leaf | 100 g: Na = 26 mg | - | Adults:  Children: | 1 g powder  1 g powder |
| TPB25 | Baobab | 100 g: Na = 100 mg | - | Adults:  Children: | 5 g powder  5 g powder |
| TPB26 | Chia seeds | n/a | - | Adults:  Children: | 28 g seeds  28 g seeds |
| TPB27 | Matcha green tea | n/a | - | Adults:  Children: | 5 g powder  5 g powder |
| TPB28 | Goji berries | 100 g: NaCl = 686 mg | - | Adults:  Children: | 3 tbsp. (~ 17 g) berries  3 tbsp. (~ 17 g) berries |
| TPB29 | Stinging nettle | n/a | - | Adults:  Children: | 3 tsp. powder  3 tsp. powder |
| TPB30 | Elder berries, zinc, vitamin C | 1 tablet: Zn = 10 mg; vit. C = 200 mg | 3923 | Adults: | 1–2 effervescent tablets |
| TPB31 | Glucomannan | n/a | - | Adults: | 3 sticks (7.5 g) effervescent powder |
| TPB32 | Turmeric, black pepper, zinc | 1 tablet: Zn = 1.5 mg | 438 | Adults: | 2 tablets |
| TPB33 | Turmeric, black pepper | n/a | 774 | Adults: | 1 tablet |
| TPB34 | Bearberries, horsetail, nettle, juniper etc. | n/a | 295 | Adults: | 3 tablets |
| TPB35 | Arctic root, black pepper, vitamin B5 | 1 tablet: vit. B5 = 1 mg | 1067 | Adults: | 1–2 tablets |
| TPB36 | Triphala rose | n/a | 1079 | Adults: | 1–2 tablets |
| TPB37 | Valerian Root | n/a | 529 | Adults: | Up to 10 tablets |
| TPB38 | Passionflower | n/a | 740 | Adults: | 1–2 tablets |
| TPB39 | Ginseng, vitamins, minerals | 1 tablet: Mg 225 mg; Fe: 14 mg; Zn = 10 mg; Cu = 1000 µg; Se = 55 µg; I = 150 µg; vit. A = 400 µg; vit. D 10 µg; vit. E = 12 mg; vit. C 80 mg; vit. B1 = 1.1 mg; vit. B2 = 1.4 mg; vit. B3 = 16 mg; vit. B6 = 1.4 mg; vit. B9 = 200 µg; vit. B12 = 2.5 µg; vit. B7 = 50 µg; vit. B5 = 6 mg | 1118 | Adults: | 1 tablet |
| TPB40 | Green tea | n/a | 685 | Adults: | 2–4 tablets |
| TPB41 | Horsetail, biotin, vitamins, minerals | 1 tablet: Se = 28 µg; Zn = 0.85 mg; I =75 µg; vit. B7 = 25 µg; vit. B3 = 8 mg; vit. B5 = 3 mg; vit. B2 = 0.7 mg; vit. B1 = 0.55 mg; vit. B6 = 0.7 mg; vit. C = 40 mg | 698 | Adults: | 2 tablets |
| TPB42 | Elderberries, vitamin C | 1 tablet: vit. C = 40 mg | 1010 | Adults:  Children: | 1–2 chewable tablets  1–2 chewable tablets |
| TPB43 | Ashwagandha, black pepper, vitamin B5 | 1 capsule: vit. B5 = 0.5 mg | 532 | Adults: | 2–4 capsules |
| TPB44 | Ashwagandha | n/a | 387 | Adults: | 2–4 capsules |
| TPB45 | Green tea | n/a | 711 | Adults: | 1–2 capsules |
| TPB46 | L-Theanine (green tea) | n/a | 314 | Adults: | 1 capsule |
| TPB47 | Green coffee, raspberry ketones | n/a | 540 | Adults: | 2 capsules |
| TPB48 | Glucomannan | n/a | 426 | Adults: | 9 capsules |
| TPB49 | Ashwagandha | n/a | 368 | Adults: | 2 capsules |
| TPB50 | Perilla extract | n/a | 409 | Adults: | 2–4 capsules |
| TPB51 | Flaxseed oil | n/a | 1462 | Adults: | 1–4 capsules |
| TPB52 | Flaxseed oil, vitamin E | 1 capsule: vit. E = 3.6 mg | 395 | Adults: | 3–9 capsules |
| TPB53 | Sea buckthorn, vitamins | 1 capsule: vit. E = 2 mg; vit. A = 200 µg | 662 | Adults: | 1–4 capsules |
| TPB54 | Flaxseed oil | n/a | - | Adults:  Children: | 10 ml oil  10 ml oil |
| TPB55 | Flaxseed oil | n/a | 1323 | Adults: | 4 capsules |
| TPB56 | Wheatgrass | 100 g powder: K = 1700 mg; Ca = 220 mg; Fe = 8.1 mg; Zn = 1.9 mg; Cu = 0.6 mg | - | Adults:  Children: | Up to 15 g powder  Up to 15 g powder |
| TPB57 | Matcha green tea | n/a | - | Adults:  Children: | Up to 5 g powder  Up to 5 g powder |
| TPB58 | Flaxseed | n/a | - | Adults:  Children: | 1–2 tbsp. seeds  1–2 tbsp. seeds |
| TPB59 | Blueberries, marigold, sage | 1 tablet: Se = 62.5 μg; Zn = 15 mg; vit. E = 10 mg; vit. B2 = 1.6 mg; vit. A = 800 μg; vit. C = 80 mg | 761 | Adults: | 1 tablet |
| TPB60 | Green tea | n/a | 308 | Adults: | 1 capsule |
| TPB61 | *Echinacea purpurea* | n/a | - | Adults: | 3–5 ml oral solution |
| TPB62 | Elderberries | n/a | - | Adults:  Children 3—12 yrs: | Up to 40 ml oral solution  Up to 20 ml oral solution |

**Table S2.A** Elemental concentrations in the 29 investigated ***synthetic*** food supplements (S01-S29). Note that units have been adapted to the measured concentrations.

| **Element** | **Unit** | **LOD** | **S01** | **S02** | **S03** | **S04** | **S05** | **S06** | **S07** | **S08** | **S09** | **S10** | **S11** | **S12** | **S13** | **S14** | **S15** | **S16** | **S17** | **S18** | **S19** | **S20** | **S21** | **S22** | **S23** | **S24** | **S25** | **S26** | **S27** | **S28** | **S29** |
| --- | --- | --- | --- | --- | --- | --- | --- | --- | --- | --- | --- | --- | --- | --- | --- | --- | --- | --- | --- | --- | --- | --- | --- | --- | --- | --- | --- | --- | --- | --- | --- |
| **Ag** | µg/kg | 1 | <LOD | <LOD | 3.4 | 6.6 | <LOD | 2.3 | <LOD | 1.5 | 2.0 | 3.2 | 2.9 | 1.6 | <LOD | 0.97 | <LOD | <LOD | <LOD | <LOD | 7.6 | 2.4 | 4.5 | 4.8 | 3.3 | 1.5 | 1.1 | 1.1 | 2.3 | <LOD | <LOD |
| **Al** | mg/kg | 0.2 | 0.62 | 23 | 8.8 | 34 | 7.8 | 46 | 26 | 7.1 | 32 | 23 | 0.85 | 77 | 1.8 | 4.6 | 0.47 | <LOD | <LOD | 4.3 | 99 | 8.5 | 29 | 1.1 | 58 | <LOD | 0.25 | 1.0 | 3.5 | 0.55 | 2.0 |
| **As** | µg/kg | 2 | <LOD | 15 | 6.2 | 68 | <LOD | 28 | 9.1 | 4.5 | 65 | 52 | 3.0 | 390 | 6.9 | 10 | 6.7 | 3.8 | <LOD | 22 | 470 | 46 | 62 | 19 | 320 | <LOD | <LOD | 2.9 | 11 | 9.8 | 16 |
| **Au** | µg/kg | 1 | <LOD | <LOD | <LOD | 130 | <LOD | <LOD | <LOD | <LOD | 6.6 | <LOD | <LOD | <LOD | <LOD | <LOD | <LOD | <LOD | <LOD | <LOD | <LOD | <LOD | 1.3 | <LOD | <LOD | <LOD | <LOD | <LOD | <LOD | <LOD | <LOD |
| **B** | mg/kg | 0.5 | 0.73 | 22 | 1.0 | 2.2 | 1.2 | 16 | 2.2 | 1.3 | 4.3 | 6.2 | 1.1 | 320 | 4.6 | 6.3 | 0.65 | 0.56 | <LOD | 3.8 | 210 | 2.5 | 2.2 | 1.1 | 4.3 | <LOD | <LOD | <LOD | 0.93 | 0.65 | 51 |
| **Ba** | mg/kg | 0.2 | <LOD | 0.23 | 0.48 | 1.3 | <LOD | 0.89 | 0.32 | 1.2 | 0.62 | 0.66 | <LOD | 1.0 | <LOD | <LOD | 35 | 1.3 | 0.23 | 0.92 | 2.7 | 2.1 | 2.0 | 0.61 | 2.4 | <LOD | <LOD | <LOD | 0.47 | 0.31 | <LOD |
| **Be** | µg/kg | 1 | <LOD | <LOD | 2.1 | 17 | <LOD | 1.5 | 3.3 | <LOD | 8.8 | 10 | <LOD | 15 | <LOD | <LOD | 1.5 | 3.2 | <LOD | 1.7 | 29 | 280 | 6.2 | <LOD | 11 | <LOD | <LOD | <LOD | 3.1 | <LOD | <LOD |
| **Bi** | µg/kg | 1 | <LOD | 4.0 | <LOD | <LOD | <LOD | 2.9 | 1.5 | <LOD | <LOD | 1.0 | <LOD | 2.5 | <LOD | <LOD | <LOD | <LOD | <LOD | <LOD | 3.0 | <LOD | <LOD | 2.4 | 1.0 | <LOD | <LOD | <LOD | 2.5 | <LOD | <LOD |
| **Br** | mg/kg | 0.5 | 1.6 | 53 | 1.4 | 0.76 | 1.0 | 3.9 | 6.5 | 0.71 | 1.2 | 1.3 | <LOD | 0.63 | 390 | 180 | 1.0 | 0.84 | 0.50 | <LOD | 1.8 | 1.8 | 3.4 | 0.85 | 0.85 | <LOD | <LOD | <LOD | 2.3 | <LOD | 0.71 |
| **Ca** | g/kg | 0.002 | 0.038 | 0.48 | 10 | 510 | 0.70 | 1.6 | 11 | 0.34 | 230 | 210 | 2.6 | 95 | 14 | 0.68 | 62 | 22 | 0.60 | 32 | 200 | 130 | 240 | 0.13 | 110 | 0.24 | 0.019 | 0.27 | 0.039 | 0.011 | 3.7 |
| **Cd** | µg/kg | 1 | <LOD | 6.0 | 3.9 | 360 | <LOD | 69 | 4.8 | 1.3 | 210 | 190 | 16 | 290 | 2.0 | 10 | 24 | 9.1 | <LOD | 44 | 510 | 15 | 230 | 3.0 | 300 | <LOD | <LOD | <LOD | 3.4 | 1.5 | 1.8 |
| **Ce** | µg/kg | 1 | <LOD | 14 | 49 | 300 | 5.5 | 27 | 27 | 4.2 | 170 | 160 | 4.6 | 410 | 7.0 | 12 | 3,400 | 100 | 9.7 | 37 | 370 | 360 | 180 | 1.9 | 96 | <LOD | <LOD | <LOD | 3.6 | 1.2 | 4.6 |
| **Co** | µg/kg | 1 | 19 | 33 | 1,200 | 34 | 16 | 4,200 | 240 | 17 | 2,000 | 1,800 | 26 | 750 | 3.7 | 1,400 | 45 | 2.4 | <LOD | 90 | 150 | 48 | 29 | 9.0 | 230 | 81 | 53 | 21 | 6.0 | 1.5 | 65 |
| **Cr** | mg/kg | 0.001 | 0.021 | 4.8 | 0.47 | 5.1 | 0.26 | 88 | 0.35 | 0.086 | 5.2 | 25 | 0.39 | 88 | 0.13 | 10 | 3.1 | 0.18 | 0.029 | 6.8 | 39 | 0.25 | 2.9 | 0.034 | 31 | 0.025 | 0.0062 | 1.7 | 0.24 | 0.22 | 12 |
| **Cs** | µg/kg | 1 | <LOD | 1.5 | <LOD | 2.3 | <LOD | 7.7 | 28 | 7.0 | 1.4 | <LOD | <LOD | 2.6 | 16 | <LOD | 1.0 | <LOD | <LOD | <LOD | 3.8 | 1.0 | 1.6 | <LOD | 2.2 | <LOD | <LOD | <LOD | 1.7 | <LOD | <LOD |
| **Cu** | mg/kg | 0.005 | 0.023 | 0.34 | 0.66 | 0.30 | 0.26 | 1,900 | 5.9 | 0.90 | 1,100 | 670 | 10 | 420 | 2.7 | 770 | 74 | 3.1 | 0.18 | 130 | 640 | 3.4 | 0.70 | 0.081 | 0.53 | 0.32 | 0.045 | 46 | 0.29 | 0.076 | 55 |
| **Dy** | µg/kg | 1 | <LOD | 1.2 | 5.1 | 68 | <LOD | 2.7 | 6.8 | <LOD | 38 | 35 | <LOD | 170 | 1.9 | <LOD | 160 | 5.0 | <LOD | 15 | 190 | 110 | 43 | <LOD | 15 | <LOD | <LOD | <LOD | <LOD | <LOD | 1.8 |
| **Er** | µg/kg | 1 | <LOD | <LOD | 3.3 | 46 | <LOD | 1.8 | 4.0 | <LOD | 26 | 25 | 1.1 | 120 | 1.8 | <LOD | 69 | 2.5 | <LOD | 10 | 140 | 74 | 29 | <LOD | 10 | <LOD | <LOD | <LOD | <LOD | <LOD | 1.3 |
| **Eu** | µg/kg | 1 | <LOD | <LOD | 1.1 | 14 | <LOD | <LOD | 1.3 | <LOD | 7.8 | 7.4 | <LOD | 27 | <LOD | <LOD | 73 | 1.6 | <LOD | 2.5 | 27 | 16 | 7.8 | <LOD | 2.5 | <LOD | <LOD | <LOD | <LOD | <LOD | <LOD |
| **Fe** | g/kg | 0.0002 | 0.00046 | 0.034 | 4.7 | 0.036 | 0.0068 | 26 | 17 | 0.29 | 19 | 12 | 0.18 | 9.7 | 0.0093 | 9.9 | 1.0 | 0.043 | 0.0026 | 2.1 | 1.3 | 0.090 | 0.022 | 0.0015 | 5.8 | 0.00027 | <LOD | 0.0047 | 0.0050 | 0.00071 | 0.47 |
| **Ga** | µg/kg | 5 | <LOD | 9.9 | 150 | 7.6 | <LOD | 12 | 5.8 | <LOD | 43 | 11 | 75 | 140 | <LOD | 43 | 19 | <LOD | <LOD | 21 | 63 | 74 | 7.2 | <LOD | 59 | <LOD | <LOD | <LOD | <LOD | <LOD | <LOD |
| **Gd** | µg/kg | 1 | <LOD | 1.0 | 5.2 | 74 | <LOD | 4.2 | 6.5 | <LOD | 41 | 38 | <LOD | 160 | 2.0 | <LOD | 210 | 5.7 | <LOD | 15 | 190 | 120 | 45 | <LOD | 16 | <LOD | <LOD | <LOD | <LOD | <LOD | 1.8 |
| **Ge** | µg/kg | 10 | <LOD | <LOD | <LOD | <LOD | <LOD | <LOD | 26 | <LOD | <LOD | <LOD | <LOD | 18 | <LOD | 430 | 150 | <LOD | <LOD | 86 | 240 | <LOD | 13 | 340 | 180 | <LOD | <LOD | 27 | <LOD | <LOD | 21 |
| **Hf** | µg/kg | 1 | <LOD | 3.6 | 30 | 2.1 | 3.9 | 25 | 4.8 | <LOD | 8.9 | 42 | <LOD | 21 | <LOD | 1.9 | <LOD | <LOD | <LOD | <LOD | 28 | 2.2 | 3.6 | 1.1 | 8.0 | <LOD | <LOD | <LOD | <LOD | <LOD | <LOD |
| **Hg** | µg/kg | 1 | <LOD | <LOD | <LOD | <LOD | <LOD | 3.8 | <LOD | <LOD | 1.3 | 1.3 | <LOD | 1.9 | 2.3 | <LOD | <LOD | <LOD | <LOD | 1.9 | 1.3 | <LOD | <LOD | 1.5 | 4.3 | <LOD | <LOD | <LOD | <LOD | <LOD | <LOD |
| **Ho** | µg/kg | 1 | <LOD | <LOD | 1.1 | 17 | <LOD | <LOD | 1.5 | <LOD | 9.2 | 8.3 | <LOD | 41 | <LOD | <LOD | 28 | <LOD | <LOD | 3.8 | 47 | 26 | 10 | <LOD | 3.0 | <LOD | <LOD | <LOD | <LOD | <LOD | <LOD |
| **I** | mg/kg | 0.25 | 12 | 7.2 | 10 | 0.58 | 7.7 | 290 | 93 | 43 | 190 | 160 | 120 | 97 | 35 | 240 | 15 | 4.9 | 3.4 | 74 | 100 | 130 | 40 | 7.8 | 100 | 0.28 | 0.28 | 9.2 | 0.50 | 1.6 | 7.0 |
| **Ir** | µg/kg | 1 | <LOD | <LOD | <LOD | <LOD | <LOD | <LOD | <LOD | <LOD | <LOD | <LOD | <LOD | <LOD | <LOD | <LOD | <LOD | <LOD | <LOD | <LOD | <LOD | <LOD | <LOD | <LOD | <LOD | <LOD | <LOD | <LOD | <LOD | <LOD | <LOD |
| **K** | g/kg | 0.005 | 0.0050 | 0.045 | 0.10 | <LOD | 0.029 | 0.41 | 2.9 | 0.83 | 0.15 | <LOD | 0.010 | <LOD | 48 | 72 | 0.17 | 0.027 | 0.82 | 0.12 | 0.063 | 0.029 | 0.11 | 0.043 | 0.12 | 0.024 | 0.13 | 0.032 | 1.5 | 0.0079 | 1.9 |
| **La** | µg/kg | 1 | <LOD | 6.4 | 31 | 510 | 3.9 | 13 | 26 | 3.1 | 280 | 260 | 5.5 | 670 | 12 | 6.9 | 1,700 | 51 | 5.0 | 73 | 630 | 180 | 300 | 1.5 | 61 | <LOD | <LOD | <LOD | 2.0 | 1.1 | 7.2 |
| **Li** | µg/kg | 30 | 130 | 570 | 86 | 42 | 53 | 140 | 88 | 83 | 99 | 150 | 35 | 210 | 9,300 | 120 | <LOD | <LOD | <LOD | <LOD | 320 | 150 | 240 | 110 | 240 | <LOD | <LOD | <LOD | 64 | <LOD | 540 |
| **Lu** | µg/kg | 1 | <LOD | <LOD | <LOD | 5.4 | <LOD | <LOD | <LOD | <LOD | 3.1 | 3.0 | <LOD | 13 | <LOD | <LOD | 6.3 | <LOD | <LOD | 1.3 | 16 | 8.0 | 4.1 | <LOD | 1.5 | <LOD | <LOD | <LOD | <LOD | <LOD | <LOD |
| **Mg** | g/kg | 0.0002 | 0.94 | 410 | 2.9 | 0.62 | 120 | 180 | 1.1 | 1.0 | 68 | 100 | 2.0 | 140 | 6.1 | 69 | 22 | 0.92 | 0.056 | 11 | 92 | 2.0 | 0.75 | 0.82 | 26 | 0.027 | 0.0090 | 20 | 0.68 | 0.0021 | 5.8 |
| **Mn** | mg/kg | 0.02 | 0.057 | 7.6 | 5.3 | 3.0 | 2.1 | 3,800 | 60 | 6.5 | 120 | 1,700 | 22 | 1,200 | 1.6 | 1,300 | 140 | 6.0 | 0.36 | 250 | 770 | 41 | 1.9 | 0.13 | 500 | 0.048 | 0.24 | 0.56 | 0.16 | 0.047 | 72 |
| **Mo** | mg/kg | 0.005 | 0.0093 | 0.55 | 0.037 | 0.0090 | 0.026 | 100 | 0.31 | 0.068 | 0.18 | 38 | 0.58 | 99 | 0.036 | 0.12 | 4.1 | 0.20 | 0.014 | 2.2 | 63 | 0.029 | 0.025 | 0.11 | 71 | <LOD | <LOD | <LOD | 0.075 | 0.015 | <LOD |
| **Na** | g/kg | 0.002 | 0.20 | 0.87 | 0.85 | 0.16 | 1.6 | 21 | 9.4 | 0.32 | 0.89 | 0.92 | 3.4 | 0.36 | 150 | 0.41 | 68 | 81 | 72 | 75 | 0.46 | 0.18 | 0.48 | 0.24 | 0.24 | 0.26 | 0.091 | 0.25 | 0.54 | 0.53 | 0.33 |
| **Nb** | µg/kg | 1 | <LOD | 6.4 | 140 | 11 | 7.8 | 680 | 33 | <LOD | 450 | 760 | 3.9 | 370 | 2.2 | 64 | 9.9 | <LOD | <LOD | 3.9 | 280 | 5.2 | 5.1 | 3.7 | 20 | <LOD | <LOD | <LOD | 8.8 | <LOD | <LOD |
| **Nd** | µg/kg | 1 | 1.4 | 6.3 | 28 | 300 | 3.8 | 32 | 26 | 5.7 | 170 | 160 | 10 | 460 | 9.8 | 23 | 1,300 | 37 | 4.1 | 54 | 460 | 260 | 180 | 2.6 | 65 | <LOD | <LOD | 1.2 | 2.3 | 1.5 | 9.2 |
| **Ni** | µg/kg | 50 | 160 | 1,300 | 800 | 310 | 260 | 1,900 | 660 | 110 | 1,600 | 1,100 | 120 | 3,600 | 150 | 1,100 | 67 | <LOD | <LOD | 540 | 860 | 150 | 290 | 59 | 1,000 | <LOD | <LOD | 79 | 94 | <LOD | 89 |
| **Os** | µg/kg | 5 | <LOD | <LOD | <LOD | <LOD | <LOD | <LOD | <LOD | <LOD | <LOD | <LOD | <LOD | <LOD | <LOD | <LOD | <LOD | <LOD | <LOD | <LOD | <LOD | <LOD | <LOD | <LOD | <LOD | <LOD | <LOD | <LOD | <LOD | <LOD | <LOD |
| **P** | g/kg | 0.001 | 0.0093 | 0.11 | 0.43 | 0.21 | 0.38 | 0.11 | 6.0 | 0.19 | 0.11 | 0.11 | 0.012 | 1.0 | 0.010 | 0.015 | 1.0 | 0.047 | 0.0036 | 0.041 | 5.2 | 100 | 0.24 | 0.051 | 3.6 | 0.014 | 0.021 | 0.020 | 0.030 | 0.018 | 0.0036 |
| **Pb** | µg/kg | 1 | 7.0 | 45 | 45 | 63 | 23 | 140 | 71 | 5.7 | 71 | 140 | 32 | 140 | 9.3 | 26 | 26 | 31 | 1.1 | 15 | 220 | 260 | 54 | 2.6 | 70 | 5.0 | 3.3 | 22 | 15 | 5.5 | 28 |
| **Pd** | µg/kg | 5 | <LOD | <LOD | 16 | <LOD | <LOD | 39 | <LOD | <LOD | 12 | 14 | <LOD | 29 | <LOD | <LOD | <LOD | <LOD | <LOD | <LOD | 39 | <LOD | <LOD | <LOD | 55 | <LOD | <LOD | <LOD | <LOD | <LOD | <LOD |
| **Pr** | µg/kg | 1 | <LOD | 1.4 | 7.0 | 70 | <LOD | 3.4 | 5.3 | <LOD | 38 | 36 | <LOD | 99 | 2.2 | 1.6 | 370 | 9.9 | 1.2 | 11 | 100 | 50 | 40 | <LOD | 14 | <LOD | <LOD | <LOD | <LOD | <LOD | 2.1 |
| **Pt** | µg/kg | 1 | <LOD | <LOD | <LOD | <LOD | <LOD | <LOD | <LOD | <LOD | <LOD | <LOD | <LOD | <LOD | <LOD | <LOD | <LOD | <LOD | <LOD | <LOD | <LOD | <LOD | <LOD | <LOD | <LOD | <LOD | <LOD | <LOD | <LOD | <LOD | <LOD |
| **Rb** | mg/kg | 0.01 | <LOD | 0.044 | 0.050 | 0.027 | 0.011 | 0.076 | 6.5 | 1.7 | 0.085 | <LOD | 0.021 | 0.034 | 3.8 | 5.2 | 0.082 | 0.018 | 0.066 | 0.081 | 0.12 | 0.027 | 0.14 | 0.018 | 0.16 | 0.024 | 0.064 | 0.020 | 0.22 | <LOD | 0.15 |
| **Re** | µg/kg | 1 | <LOD | <LOD | <LOD | <LOD | <LOD | 1.1 | <LOD | <LOD | <LOD | <LOD | <LOD | <LOD | <LOD | <LOD | <LOD | <LOD | <LOD | <LOD | <LOD | <LOD | <LOD | <LOD | <LOD | <LOD | <LOD | <LOD | <LOD | <LOD | <LOD |
| **Rh** | µg/kg | 5 | <LOD | <LOD | <LOD | <LOD | <LOD | 16 | <LOD | <LOD | 8.0 | 5.5 | <LOD | <LOD | <LOD | 15 | <LOD | <LOD | <LOD | <LOD | 15 | <LOD | 13 | <LOD | <LOD | <LOD | <LOD | <LOD | <LOD | <LOD | <LOD |
| **Ru** | µg/kg | 5 | <LOD | <LOD | <LOD | <LOD | <LOD | <LOD | <LOD | <LOD | <LOD | <LOD | <LOD | <LOD | <LOD | <LOD | <LOD | <LOD | <LOD | <LOD | <LOD | <LOD | <LOD | <LOD | <LOD | <LOD | <LOD | <LOD | <LOD | <LOD | <LOD |
| **S** | g/kg | 0.005 | 0.011 | 0.26 | 6.9 | 0.11 | 0.013 | 4.2 | 4.6 | 0.11 | 0.48 | 4.9 | 0.057 | 15 | 0.092 | 5.2 | 35 | 1.3 | 0.81 | 2.8 | 5.6 | 14 | 0.14 | 0.059 | 0.44 | 0.46 | 0.55 | 0.63 | 0.61 | 0.62 | 0.049 |
| **Sb** | µg/kg | 1 | <LOD | 2.4 | 12 | 4.7 | <LOD | 4.6 | 7.8 | <LOD | 3.1 | 3.0 | <LOD | 69 | <LOD | 1.2 | <LOD | 3.4 | <LOD | 3.4 | 150 | 990 | 13 | 3.8 | 94 | 1.4 | 1.1 | 2.2 | 10 | 3.7 | 3.6 |
| **Sc** | µg/kg | 1 | <LOD | 3.1 | 6.5 | 24 | 1.0 | 34 | 4.5 | 1.0 | 25 | 23 | 1.1 | 33 | 1.5 | 12 | 6.2 | 1.9 | <LOD | 4.5 | 52 | 110 | 30 | 1.6 | 17 | <LOD | <LOD | <LOD | 1.1 | <LOD | 1.0 |
| **Se** | mg/kg | 0.05 | <LOD | 0.058 | <LOD | <LOD | <LOD | 100 | 1.2 | <LOD | 63 | 45 | 0.48 | 47 | <LOD | 44 | 16 | 0.78 | <LOD | 6.3 | 39 | 0.25 | 0.052 | 220 | 16 | <LOD | <LOD | 2.4 | 320 | <LOD | 3.4 |
| **Si** | mg/kg | 5 | 110 | 30 | 700 | 95 | 160 | 110 | 1,100 | 71 | 710 | 250 | 1,200 | 150 | 11 | 21 | 32 | 7.7 | <LOD | 19 | 110 | 16 | 300 | 170 | 320 | 42 | 48 | 34 | 14 | 53 | 17 |
| **Sm** | µg/kg | 1 | <LOD | 1.1 | 5.7 | 55 | 2.6 | 2.5 | 5.0 | <LOD | 34 | 30 | 1.1 | 98 | 1.4 | 1.1 | 250 | 7.6 | 1.5 | 9.2 | 110 | 74 | 35 | <LOD | 13 | <LOD | <LOD | <LOD | <LOD | <LOD | 1.1 |
| **Sn** | µg/kg | 1 | <LOD | 20 | 18 | 8.4 | <LOD | 85 | 2.4 | 0.5 | 100 | 110 | <LOD | 100 | 17 | 24 | 20 | 6.6 | <LOD | <LOD | 22 | 22 | 12 | <LOD | 28 | 14 | <LOD | 83 | 13 | 4.0 | 8.1 |
| **Sr** | mg/kg | 0.02 | 0.44 | 5.2 | 2.4 | 180 | 0.61 | 3.2 | 4.9 | 1.5 | 90 | 88 | 1.2 | 54 | 180 | 1.5 | 26 | 3.8 | 0.52 | 13 | 91 | 43 | 65 | 0.15 | 57 | 0.48 | 0.078 | 0.47 | 0.43 | 0.063 | 1.8 |
| **Ta** | µg/kg | 50 | <LOD | <LOD | <LOD | 60 | <LOD | 300 | <LOD | <LOD | <LOD | 79 | <LOD | <LOD | <LOD | <LOD | <LOD | <LOD | <LOD | <LOD | <LOD | <LOD | <LOD | <LOD | <LOD | <LOD | <LOD | <LOD | <LOD | <LOD | <LOD |
| **Tb** | µg/kg | 1 | <LOD | <LOD | <LOD | 10 | <LOD | <LOD | 1.1 | <LOD | 5.7 | 5.5 | <LOD | 25 | <LOD | <LOD | 25 | <LOD | <LOD | 2.0 | 26 | 19 | 7.2 | <LOD | 2.3 | <LOD | <LOD | <LOD | <LOD | <LOD | <LOD |
| **Te** | µg/kg | 5 | <LOD | <LOD | <LOD | <LOD | <LOD | <LOD | <LOD | <LOD | <LOD | <LOD | <LOD | <LOD | <LOD | <LOD | <LOD | <LOD | <LOD | <LOD | <LOD | <LOD | <LOD | 11 | <LOD | <LOD | <LOD | <LOD | <LOD | <LOD | <LOD |
| **Th** | µg/kg | 1 | 2.3 | 3.1 | 9.9 | 16 | 2.2 | 11 | 3.3 | 1.4 | 13 | 9.8 | 1.9 | 15 | <LOD | 6.3 | 7.4 | <LOD | <LOD | 2.6 | 35 | 6.9 | 14 | <LOD | 7.7 | <LOD | <LOD | <LOD | 1.8 | <LOD | <LOD |
| **Ti** | mg/kg | 0.02 | 0.066 | 0.46 | 270 | 2.1 | 30 | 1,700 | 55 | 2.0 | 61 | 2,800 | 11 | 3,800 | 3.7 | 58 | 0.089 | <LOD | <LOD | 0.42 | 3,200 | 0.58 | 1.5 | 0.12 | 2.4 | 0.024 | <LOD | 0.023 | 0.16 | 0.12 | 0.11 |
| **Tl** | µg/kg | 1 | <LOD | <LOD | <LOD | 2.7 | <LOD | 15 | 3.2 | <LOD | 7.6 | 4.2 | 1.1 | 3.1 | 2.1 | 5.6 | 37 | 1.9 | <LOD | <LOD | 2.4 | 23 | 1.7 | <LOD | 30 | <LOD | <LOD | 7.2 | <LOD | <LOD | <LOD |
| **Tm** | µg/kg | 1 | <LOD | <LOD | <LOD | 6.4 | <LOD | <LOD | <LOD | <LOD | 3.6 | 3.3 | <LOD | 16 | <LOD | <LOD | 8.6 | <LOD | <LOD | 1.5 | 18 | 9.3 | 3.7 | <LOD | 1.5 | <LOD | <LOD | <LOD | <LOD | <LOD | <LOD |
| **U** | µg/kg | 1 | <LOD | 9.2 | 7.4 | 190 | 3.1 | 28 | 17 | 1.4 | 110 | 100 | 4.7 | 950 | 5.9 | 3.1 | 5.9 | 4.3 | <LOD | 20 | 830 | 150 | 130 | 12 | 290 | 2.8 | <LOD | 3.9 | <LOD | <LOD | 1.8 |
| **W** | µg/kg | 1 | 1.1 | 12 | 3.9 | 3.0 | 5.0 | 10 | 8.8 | 3.7 | 8.2 | 11 | 2.4 | 43 | 2.0 | 3.2 | 1.4 | 8.3 | <LOD | 3.1 | 35 | 46 | 6.4 | 2.1 | 17 | <LOD | <LOD | <LOD | 4.3 | 1.8 | 1.0 |
| **V** | µg/kg | 5 | 11 | 47 | 2,200 | 410 | 17 | 1,700 | 110 | 17 | 980 | 1,100 | 39 | 3,700 | 34 | 650 | 47 | <LOD | <LOD | 430 | 2,500 | 630 | 190 | 50 | 1,800 | 13 | <LOD | 14 | 28 | 12 | 13 |
| **Y** | µg/kg | 1 | <LOD | 13 | 31 | 820 | 4.9 | 18 | 63 | 4.1 | 440 | 410 | 8.5 | 2,600 | 32 | 6.9 | 750 | 25 | 2.3 | 190 | 2,200 | 970 | 340 | 2.2 | 120 | <LOD | <LOD | 1.2 | 2.7 | <LOD | 22 |
| **Yb** | µg/kg | 1 | <LOD | 1.1 | 2.8 | 37 | <LOD | 1.5 | 4.0 | <LOD | 20 | 19 | <LOD | 88 | <LOD | <LOD | 47 | 1.6 | <LOD | 8.6 | 100 | 54 | 25 | <LOD | 8.5 | <LOD | <LOD | <LOD | <LOD | <LOD | 1.6 |
| **Zn** | mg/kg | 0.1 | 0.17 | 0.54 | 1.4 | 3.3 | 0.40 | 18,000 | 51 | 7.3 | 9,700 | 5,500 | 14,000 | 8,500 | 3.6 | 7,500 | 3,000 | 140 | 7.2 | 1,000 | 9,200 | 16,000 | 31 | 2.5 | 3,800 | 0.17 | <LOD | 410 | 2.8 | <LOD | 520 |
| **Zr** | µg/kg | 1 | 2.3 | 200 | 940 | 140 | 120 | 660 | 220 | 28 | 360 | 850 | 22 | 660 | 13 | 45 | 15 | 6.3 | 2.1 | 26 | 820 | 120 | 90 | 29 | 420 | 6.0 | 2.7 | 3.3 | 30 | 6.6 | 6.7 |
| **REE** | mg/kg | - | 0.0089 | 0.052 | 0.18 | 2.4 | 0.027 | 0.14 | 0.18 | 0.024 | 1.3 | 1.2 | 0.037 | 5.1 | 0.074 | 0.068 | 8.5 | 0.25 | 0.029 | 0.44 | 4.7 | 2.4 | 1.3 | 0.015 | 0.45 | 0.0080 | 0.0080 | 0.0094 | 0.017 | 0.010 | 0.056 |

**Table S2.B, part 1 (of 2)** Elemental concentrations in the 38 investigated ***marine non-fish-oil*** food supplements (M01-M38). Note that units have been adapted to the measured concentrations.

| **Element** | **Unit** | **LOD** | **M01** | **M02** | **M03** | **M04** | **M05** | **M06** | **M07** | **M08** | **M09** | **M10** | **M11** | **M12** | **M13** | **M14** | **M15** | **M16** | **M17** | **M18** | **M19** |
| --- | --- | --- | --- | --- | --- | --- | --- | --- | --- | --- | --- | --- | --- | --- | --- | --- | --- | --- | --- | --- | --- |
| **Ag** | µg/kg | 1 | 1.7 | 17 | 6.1 | 54 | 18 | 21 | 7.7 | 6.3 | 21 | 4.0 | 21 | 17 | 12 | 1.5 | 2.4 | 4.4 | 10 | 1.0 | 0.5 |
| **Al** | mg/kg | 0.2 | 37 | 84 | 320 | 99 | 86 | 110 | 75 | 5.8 | 290 | 10 | 88 | 140 | 120 | 4.7 | 250 | 130 | 9.5 | 0.78 | 1.3 |
| **As** | µg/kg | 2 | 1,100 | 2,000 | 400 | 310 | 9,900 | 4,900 | 5,000 | 200 | 7,100 | 11 | 6,400 | 5,100 | 190 | 290 | 280 | 100 | 120 | 2.2 | 17 |
| **Au** | µg/kg | 1 | <LOD | <LOD | <LOD | 1.6 | <LOD | 1.3 | <LOD | 1.0 | 1.4 | <LOD | <LOD | <LOD | <LOD | <LOD | <LOD | <LOD | <LOD | <LOD | <LOD |
| **B** | mg/kg | 0.5 | 2.8 | 9.0 | 20 | 13 | 58 | 30 | 21 | 5.1 | 25 | 1.3 | 8.4 | 14 | 7.7 | 7.6 | 5.9 | 7.4 | 1.0 | 1.5 | 3.1 |
| **Ba** | mg/kg | 0.2 | 1.1 | 2.0 | 13 | 28 | 5.1 | 3.0 | 3.6 | 1.4 | 2.9 | 0.65 | 1.9 | 2.0 | 5.2 | 1.5 | 5.4 | 5.6 | 1.9 | 0.33 | <LOD |
| **Be** | µg/kg | 1 | 7.5 | 5.0 | 16 | 32 | 19 | 9.2 | <LOD | <LOD | 13 | 1.5 | 17 | 4.3 | 4.7 | <LOD | 14 | 9.4 | 6.1 | <LOD | <LOD |
| **Bi** | µg/kg | 1 | 3.1 | 10 | 7.4 | 19 | 3.4 | 1.8 | 1.9 | 4.3 | 1.8 | <LOD | 4.2 | <LOD | 3.6 | 2.9 | 5.5 | 4.8 | <LOD | <LOD | <LOD |
| **Br** | mg/kg | 0.5 | 18 | 8.2 | 19 | 2.5 | 310 | 120 | 220 | 6.8 | 200 | <LOD | 22 | 140 | 7.0 | 3.0 | 4.6 | 3.8 | 1.5 | 1.5 | 0.85 |
| **Ca** | g/kg | 0.002 | 51 | 1.0 | 8.2 | 4.8 | 84 | 140 | 2.7 | 8.6 | 69 | 22 | 1.6 | 120 | 2.2 | 1.6 | 1.9 | 68 | 0.22 | 0.067 | 0.19 |
| **Cd** | µg/kg | 1 | 120 | 26 | 200 | 71 | 240 | 100 | 720 | 30 | 350 | 22 | 480 | 160 | 54 | 5.5 | 33 | 51 | 6.2 | 3.4 | 16 |
| **Ce** | µg/kg | 1 | 88 | 180 | 570 | 360 | 3,100 | 400 | 62 | 39 | 610 | 30 | 98 | 2,400 | 180 | 7.8 | 290 | 2,300 | 36 | <LOD | 1.2 |
| **Co** | µg/kg | 1 | 180 | 380 | 410 | 370 | 280 | 280 | 70 | 74 | 2,100 | 640 | 240 | 450 | 260 | 290 | 160 | 380 | 5,700 | 4.2 | 10 |
| **Cr** | mg/kg | 0.001 | 0.73 | 0.17 | 1.3 | 1.1 | 0.76 | 0.69 | 0.15 | 0.34 | 0.78 | 0.49 | 0.45 | 0.56 | 0.52 | 0.25 | 0.64 | 17 | 0.33 | 0.036 | 0.032 |
| **Cs** | µg/kg | 1 | 7.2 | 21 | 61 | 270 | 20 | 7.0 | 13 | 6.9 | 18 | <LOD | 16 | 9.2 | 27 | 5.4 | 74 | 14 | 3.0 | <LOD | <LOD |
| **Cu** | mg/kg | 0.005 | 1.4 | 1.9 | 14 | 2.7 | 0.65 | 0.32 | 0.54 | 4.9 | 2.0 | 0.68 | 1.8 | 1.8 | 2.4 | 4.9 | 5.1 | 350 | 360 | 0.041 | 0.026 |
| **Dy** | µg/kg | 1 | 9.1 | 9.0 | 31 | 140 | 140 | 58 | 4.2 | 10 | 99 | 6.4 | 6.8 | 200 | 16 | 2.8 | 18 | 130 | 23 | <LOD | <LOD |
| **Er** | µg/kg | 1 | 7.3 | 4.2 | 16 | 160 | 74 | 40 | 3.3 | 5.9 | 65 | 3.8 | 3.4 | 110 | 10 | 1.8 | 10 | 65 | 26 | <LOD | <LOD |
| **Eu** | µg/kg | 1 | 2.1 | 2.3 | 8.2 | 8.5 | 59 | 12 | <LOD | 2.9 | 25 | 1.3 | 1.7 | 60 | 3.6 | <LOD | 5.6 | 50 | 1.5 | <LOD | <LOD |
| **Fe** | g/kg | 0.0002 | 0.058 | 0.58 | 0.49 | 1.0 | 0.27 | 0.17 | 0.066 | 0.28 | 8.3 | 5.1 | 0.12 | 0.28 | 0.40 | 0.51 | 0.25 | 5.7 | 0.10 | 0.00071 | 0.0028 |
| **Ga** | µg/kg | 5 | 13 | 28 | 83 | 41 | 27 | 45 | 11 | 6.5 | 130 | 7.8 | 15 | 55 | 35 | <LOD | 45 | 60 | 5.0 | <LOD | <LOD |
| **Gd** | µg/kg | 1 | 10 | 12 | 40 | 72 | 220 | 72 | 4.6 | 13 | 100 | 6.8 | 8.8 | 230 | 22 | 1.4 | 24 | 170 | 13 | <LOD | <LOD |
| **Ge** | µg/kg | 10 | <LOD | <LOD | 12 | 61 | 23 | 25 | <LOD | 11 | 18 | 310 | 16 | <LOD | <LOD | <LOD | <LOD | 250 | <LOD | <LOD | <LOD |
| **Hf** | µg/kg | 1 | 1.6 | 2.8 | 9.3 | 27 | 7.3 | 15 | 1.2 | 1.3 | 32 | 15 | 1.8 | 14 | 3.8 | <LOD | 1.3 | 10 | 18 | <LOD | <LOD |
| **Hg** | µg/kg | 1 | 10 | 2.8 | 6.6 | <LOD | 6.9 | 6.2 | 1.9 | 3.5 | 9.3 | 1.4 | 21 | 10 | <LOD | <LOD | 2.6 | 4.5 | 8.9 | <LOD | <LOD |
| **Ho** | µg/kg | 1 | 2.2 | 1.5 | 5.8 | 40 | 29 | 13 | 1.0 | 2.8 | 22 | 1.5 | 1.3 | 40 | 3.9 | <LOD | 3.5 | 25 | 6.7 | <LOD | <LOD |
| **I** | mg/kg | 0.25 | 10 | 3.8 | 12 | 5.5 | 360 | 3,800 | 1,200 | 160 | 820 | 70 | 16 | 540 | 5.2 | 2.7 | 15 | 180 | 25 | 1.8 | 0.93 |
| **Ir** | µg/kg | 1 | <LOD | <LOD | <LOD | <LOD | <LOD | <LOD | <LOD | <LOD | <LOD | <LOD | <LOD | <LOD | <LOD | <LOD | <LOD | <LOD | <LOD | <LOD | <LOD |
| **K** | g/kg | 0.005 | 4.4 | 18 | 17 | 15 | 9.2 | 4.3 | 10 | 12 | 6.5 | 0.46 | 4.8 | 3.8 | 7.9 | 10 | 6.8 | 7.0 | 1.4 | 0.23 | 0.63 |
| **La** | µg/kg | 1 | 53 | 95 | 260 | 170 | 1,400 | 290 | 36 | 49 | 500 | 36 | 47 | 1,400 | 84 | 7.8 | 140 | 1,200 | 20 | <LOD | <LOD |
| **Li** | µg/kg | 30 | 130 | 150 | 510 | 300 | 350 | 280 | 240 | 140 | 480 | 37 | 560 | 250 | 390 | 61 | 530 | 230 | 34 | <LOD | <LOD |
| **Lu** | µg/kg | 1 | <LOD | <LOD | 1.8 | 39 | 7.4 | 5.7 | <LOD | 1.3 | 7.2 | <LOD | <LOD | 11 | 1.2 | <LOD | 1.2 | 6.7 | 7.6 | <LOD | <LOD |
| **Mg** | g/kg | 0.0002 | 3.8 | 2.9 | 2.7 | 4.4 | 3.1 | 2.6 | 1.5 | 2.6 | 3.2 | 8.1 | 2.1 | 2.4 | 3.3 | 2.8 | 2.2 | 29 | 0.55 | 0.028 | 0.12 |
| **Mn** | mg/kg | 0.02 | 28 | 34 | 45 | 48 | 59 | 11 | 1.6 | 40 | 48 | 860 | 10 | 21 | 43 | 45 | 65 | 790 | 8.7 | 0.042 | 0.19 |
| **Mo** | mg/kg | 0.005 | 0.30 | 0.19 | 1.1 | 0.15 | 0.23 | 1.5 | 0.085 | 0.41 | 0.23 | 0.18 | 0.48 | 0.14 | 0.069 | 0.32 | 0.25 | 0.33 | 0.44 | 0.0057 | 0.0061 |
| **Na** | g/kg | 0.002 | 15 | 26 | 7.1 | 3.3 | 10 | 9.2 | 5.9 | 1.2 | 11 | 0.45 | 5.9 | 5.4 | 11 | 0.93 | 0.72 | 4.2 | 1.1 | 3.7 | 0.41 |
| **Nb** | µg/kg | 1 | 7.6 | 8.1 | 61 | 41 | 21 | 36 | 3.5 | 3.9 | 65 | 6.2 | 7.1 | 38 | 15 | 2.4 | 6.1 | 28 | 3.9 | 2.8 | <LOD |
| **Nd** | µg/kg | 1 | 47 | 80 | 230 | 190 | 1,300 | 410 | 85 | 49 | 420 | 29 | 47 | 1,200 | 94 | 5.6 | 130 | 1,100 | 25 | 1.1 | 1.1 |
| **Ni** | µg/kg | 50 | 810 | 1,500 | 1,500 | 1,100 | 1,600 | 490 | 340 | 330 | 2,800 | 740 | 1,300 | 470 | 690 | 210 | 1,300 | 2,900 | 260 | <LOD | <LOD |
| **Os** | µg/kg | 5 | <LOD | <LOD | <LOD | <LOD | <LOD | <LOD | <LOD | <LOD | <LOD | <LOD | <LOD | <LOD | <LOD | <LOD | <LOD | <LOD | <LOD | <LOD | <LOD |
| **P** | g/kg | 0.001 | 37 | 11 | 5.3 | 12 | 61 | 110 | 0.89 | 16 | 39 | 5.2 | 3.3 | 87 | 4.1 | 9.2 | 1.7 | 3.3 | 1.0 | 0.012 | 0.19 |
| **Pb** | µg/kg | 1 | 190 | 190 | 690 | 1,300 | 200 | 73 | 52 | 66 | 280 | 20 | 200 | 140 | 240 | 38 | 330 | 310 | 240 | 3.5 | 6.2 |
| **Pd** | µg/kg | 5 | <LOD | <LOD | <LOD | 28 | <LOD | 8.3 | <LOD | <LOD | <LOD | <LOD | <LOD | 6.5 | <LOD | <LOD | <LOD | <LOD | 16 | <LOD | <LOD |
| **Pr** | µg/kg | 1 | 12 | 21 | 61 | 39 | 340 | 60 | 7.3 | 8.6 | 100 | 6.3 | 10 | 290 | 22 | 1.3 | 34 | 270 | 4.6 | <LOD | <LOD |
| **Pt** | µg/kg | 1 | <LOD | <LOD | <LOD | <LOD | <LOD | <LOD | <LOD | 2.2 | <LOD | <LOD | <LOD | <LOD | <LOD | <LOD | <LOD | <LOD | <LOD | <LOD | <LOD |
| **Rb** | mg/kg | 0.01 | 1.5 | 2.0 | 10 | 2.0 | 2.8 | 1.3 | 5.8 | 2.7 | 2.7 | 0.24 | 3.9 | 1.6 | 8.4 | 2.1 | 8.8 | 3.5 | 0.28 | 0.19 | 0.26 |
| **Re** | µg/kg | 1 | <LOD | <LOD | 1.5 | <LOD | 18 | 29 | 1.2 | <LOD | 34 | <LOD | <LOD | 21 | 3.4 | <LOD | <LOD | <LOD | <LOD | <LOD | <LOD |
| **Rh** | µg/kg | 5 | <LOD | <LOD | <LOD | <LOD | <LOD | 12 | 11 | 12 | 8.6 | <LOD | <LOD | 5.8 | <LOD | <LOD | <LOD | 7.2 | 6.5 | <LOD | <LOD |
| **Ru** | µg/kg | 5 | <LOD | <LOD | <LOD | <LOD | <LOD | <LOD | <LOD | <LOD | <LOD | <LOD | <LOD | <LOD | <LOD | <LOD | <LOD | <LOD | <LOD | <LOD | <LOD |
| **S** | g/kg | 0.005 | 38 | 11 | 4.5 | 8.2 | 9.2 | 4.6 | 2.1 | 7.9 | 19 | 0.35 | 6.4 | 3.9 | 3.8 | 6.5 | 2.2 | 1.8 | 1.3 | 5.3 | 1.0 |
| **Sb** | µg/kg | 1 | 90 | 19 | 26 | 32 | 330 | 40 | 11 | 10 | 420 | 6.7 | 6.2 | 1,300 | 7.7 | 5.0 | 14 | 15 | 4.0 | 2.8 | <LOD |
| **Sc** | µg/kg | 1 | 13 | 13 | 80 | 290 | 47 | 52 | 7.9 | 4.2 | 120 | 4.8 | 19 | 100 | 39 | 3.7 | 19 | 42 | 59 | <LOD | <LOD |
| **Se** | mg/kg | 0.05 | 0.25 | 0.53 | 0.14 | 0.24 | <LOD | <LOD | <LOD | <LOD | 0.16 | 25 | 0.72 | 0.072 | 0.062 | <LOD | <LOD | 21 | <LOD | <LOD | <LOD |
| **Si** | mg/kg | 5 | 83 | 97 | 540 | 250 | 600 | 96 | 39 | 37 | 1,400 | 400 | 770 | 1,100 | 100 | 390 | 100 | 100 | 180 | 150 | 16 |
| **Sm** | µg/kg | 1 | 12 | 14 | 44 | 51 | 260 | 60 | 4.5 | 10 | 95 | 5.3 | 10 | 230 | 21 | 1.2 | 28 | 190 | 7.8 | <LOD | <LOD |
| **Sn** | µg/kg | 1 | 14 | 18 | 56 | 65 | 20 | 95 | 13 | 8.2 | 25 | 3.3 | 22 | 88 | 15 | 5.0 | 21 | 26 | 57 | 130 | 13 |
| **Sr** | mg/kg | 0.02 | 12 | 20 | 37 | 32 | 310 | 150 | 140 | 17 | 160 | 5.2 | 12 | 160 | 28 | 5.3 | 14 | 33 | 0.25 | 1.1 | 1.7 |
| **Ta** | µg/kg | 50 | <LOD | <LOD | <LOD | <LOD | <LOD | <LOD | <LOD | <LOD | <LOD | <LOD | <LOD | <LOD | <LOD | <LOD | <LOD | <LOD | <LOD | <LOD | <LOD |
| **Tb** | µg/kg | 1 | 1.6 | 1.8 | 5.3 | 15 | 29 | 11 | 1.5 | 2.5 | 15 | 1.0 | 1.2 | 32 | 3.1 | <LOD | 3.7 | 23 | 2.5 | <LOD | <LOD |
| **Te** | µg/kg | 5 | <LOD | <LOD | <LOD | <LOD | <LOD | 9.0 | <LOD | <LOD | 5.2 | <LOD | <LOD | <LOD | <LOD | <LOD | <LOD | <LOD | <LOD | <LOD | <LOD |
| **Th** | µg/kg | 1 | 19 | 23 | 89 | 980 | 49 | 69 | 20 | 8.6 | 19 | 5.2 | 15 | 17 | 23 | 11 | 25 | 48 | 220 | <LOD | <LOD |
| **Ti** | mg/kg | 0.02 | 2.6 | 2.6 | 13 | 12 | 6.2 | 8.0 | 1.4 | 2.4 | 73 | 2.3 | 3.0 | 17 | 7.1 | 4.1 | 1.8 | 5.3 | 1.6 | 0.056 | 0.11 |
| **Tl** | µg/kg | 1 | 3.4 | 24 | 47 | 15 | 29 | <LOD | <LOD | 4.9 | 3.3 | 1.1 | 6.7 | 1.7 | 17 | 3.0 | 11 | 14 | 1.5 | <LOD | <LOD |
| **Tm** | µg/kg | 1 | <LOD | <LOD | 2.4 | 28 | 10 | 5.6 | <LOD | <LOD | 8.0 | <LOD | <LOD | 13 | 1.2 | <LOD | 1.3 | 8.3 | 5.8 | <LOD | <LOD |
| **U** | µg/kg | 1 | 120 | 250 | 69 | 74 | 330 | 450 | 58 | 11 | 170 | 24 | 27 | 94 | 33 | 3.9 | 15 | 67 | 40 | <LOD | 2.3 |
| **W** | µg/kg | 1 | 19 | 43 | 64 | 160 | 32 | 26 | 8.8 | 7.0 | 13 | 7.2 | 15 | 11 | 13 | 5.7 | 19 | 32 | 11 | 1.3 | 1.5 |
| **V** | µg/kg | 5 | 500 | 260 | 660 | 180 | 860 | 1,400 | 240 | 380 | 2,900 | 68 | 1,900 | 1,200 | 550 | 37 | 340 | 570 | 37 | <LOD | 33 |
| **Y** | µg/kg | 1 | 110 | 91 | 160 | 770 | 920 | 640 | 22 | 120 | 650 | 47 | 33 | 1,300 | 90 | 10 | 100 | 750 | 140 | <LOD | 1.3 |
| **Yb** | µg/kg | 1 | 4.4 | 3.7 | 14 | 240 | 54 | 34 | 1.5 | 4.7 | 42 | 3.2 | 2.8 | 76 | 7.4 | 1.8 | 8.0 | 44 | 45 | <LOD | <LOD |
| **Zn** | mg/kg | 0.1 | 8.4 | 22 | 33 | 18 | 8.4 | 8.8 | 1.6 | 17 | 9,100 | 1000 | 1,500 | 2.6 | 18 | 15 | 60 | 3,000 | 11 | 0.32 | 0.23 |
| **Zr** | µg/kg | 1 | 78 | 110 | 340 | 1,700 | 380 | 610 | 57 | 46 | 960 | 470 | 70 | 520 | 180 | 13 | 52 | 470 | 670 | 2.5 | 2.9 |
| **REE** | mg/kg | - | 0.37 | 0.53 | 1.5 | 2.6 | 8.0 | 2.2 | 0.24 | 0.32 | 2.9 | 0.18 | 0.29 | 7.7 | 0.60 | 0.048 | 0.83 | 6.3 | 0.42 | 0.0086 | 0.010 |

**Table S2.B, part 2 (of 2)**

| **Element** | **Unit** | **LOD** | **M20** | **M21** | **M22** | **M23** | **M24** | **M25** | **M26** | **M27** | **M28** | **M29** | **M30** | **M31** | **M32** | **M33** | **M34** | **M35** | **M36** | **M37** | **M38** |
| --- | --- | --- | --- | --- | --- | --- | --- | --- | --- | --- | --- | --- | --- | --- | --- | --- | --- | --- | --- | --- | --- |
| **Ag** | µg/kg | 1 | <LOD | 4.9 | 3.7 | 110 | 41 | 6.2 | 3.4 | 3.2 | <LOD | 5.6 | 8.2 | 11 | 4.6 | <LOD | 10 | <LOD | <LOD | <LOD | <LOD |
| **Al** | mg/kg | 0.2 | 1.2 | 75 | 270 | 200 | 150 | 50 | 27 | 200 | 3.3 | 32 | 280 | 230 | 5.8 | 1.6 | 130 | 1.1 | 0.55 | 0.39 | 2.4 |
| **As** | µg/kg | 2 | 6.4 | 370 | 790 | 140 | 240 | 410 | 250 | 240 | 590 | 340 | 280 | 160 | 21 | 40,000 | 14,000 | 49 | 25 | 8.5 | 36 |
| **Au** | µg/kg | 1 | <LOD | <LOD | <LOD | <LOD | 1.5 | <LOD | <LOD | <LOD | <LOD | <LOD | <LOD | <LOD | <LOD | 2.1 | 0.63 | <LOD | <LOD | <LOD | <LOD |
| **B** | mg/kg | 0.5 | 4.1 | 4.1 | 6.9 | 10 | 12 | 2.4 | 1.6 | 4.3 | 5.3 | 9.0 | 14 | 14 | 4.5 | 140 | 21 | 1.6 | <LOD | <LOD | 13 |
| **Ba** | mg/kg | 0.2 | 0.72 | 1.8 | 32 | 12 | 16 | 1.5 | 0.91 | 18 | 2.6 | 33 | 7.0 | 9.0 | 2.1 | 7.2 | 0.92 | 0.23 | <LOD | 15 | <LOD |
| **Be** | µg/kg | 1 | <LOD | 3.9 | 14 | 44 | 55 | 2.1 | 1.2 | 4.0 | 3.3 | 8.0 | 14 | 10 | <LOD | <LOD | 6.7 | <LOD | <LOD | <LOD | <LOD |
| **Bi** | µg/kg | 1 | <LOD | 20 | 5.8 | 88 | 63 | 9.1 | 4.3 | 5.1 | 2.0 | 17 | 10 | 4.2 | <LOD | 5.6 | 7.5 | <LOD | <LOD | <LOD | <LOD |
| **Br** | mg/kg | 0.5 | <LOD | 1.0 | 1.3 | 1.1 | 1.1 | 1.8 | 0.82 | 2.6 | 1.1 | 6.7 | 9.0 | 7.3 | 2.0 | 470 | 180 | 3.8 | 2.3 | 5.8 | 28 |
| **Ca** | g/kg | 0.002 | 0.024 | 1.6 | 13 | 2.5 | 2.7 | 0.77 | 1.3 | 3.3 | 3.8 | 5.0 | 4.9 | 3.8 | 2.4 | 8.4 | 3.8 | 0.21 | 0.27 | 0.050 | 5.0 |
| **Cd** | µg/kg | 1 | 13 | 15 | 37 | 70 | 41 | 11 | 5.9 | 41 | 6.3 | 32 | 190 | 170 | 85 | 260 | 1,500 | <LOD | 1.6 | <LOD | 5.4 |
| **Ce** | µg/kg | 1 | 3.2 | 210 | 560 | 280 | 350 | 110 | 67 | 470 | 12 | 68 | 440 | 240 | 8.0 | 8.0 | 160 | 2.0 | <LOD | 6.0 | 59 |
| **Co** | µg/kg | 1 | 1.0 | 490 | 2,800 | 270 | 370 | 300 | 560 | 350 | 460 | 230 | 200 | 290 | 93 | 76 | 590 | 4.6 | 2.6 | 4.0 | 160 |
| **Cr** | mg/kg | 0.001 | 0.010 | 0.38 | 0.73 | 1.0 | 1.7 | 0.19 | 0.15 | 0.50 | 0.26 | 1.2 | 0.87 | 0.57 | 0.089 | 0.053 | 1.0 | 0.10 | 0.055 | 0.22 | 0.55 |
| **Cs** | µg/kg | 1 | 2.3 | 19 | 55 | 78 | 160 | 9.1 | 13 | 35 | 5.3 | 9.1 | 110 | 54 | 4.3 | 31 | 30 | <LOD | <LOD | <LOD | <LOD |
| **Cu** | mg/kg | 0.005 | 0.0082 | 0.72 | 4.2 | 3.7 | 5.5 | 0.70 | 0.27 | 7.5 | 6.7 | 3.7 | 6.4 | 6.7 | 6.7 | 0.41 | 5.0 | 0.13 | 0.20 | 0.019 | 0.094 |
| **Dy** | µg/kg | 1 | <LOD | 9.3 | 35 | 100 | 200 | 5.8 | 3.4 | 14 | 1.6 | 4.3 | 28 | 23 | 1.1 | 2.8 | 10 | <LOD | <LOD | <LOD | 1.8 |
| **Er** | µg/kg | 1 | <LOD | 5.8 | 17 | 120 | 230 | 3.8 | 1.7 | 8.4 | 1.1 | 2.5 | 13 | 13 | <LOD | 2.7 | 8.3 | <LOD | <LOD | <LOD | 1.1 |
| **Eu** | µg/kg | 1 | <LOD | 3.1 | 8.1 | 8.1 | 14 | 1.7 | 1.1 | 2.4 | <LOD | 1.5 | 7.2 | 5.5 | <LOD | <LOD | 3.4 | <LOD | <LOD | 1.5 | <LOD |
| **Fe** | g/kg | 0.0002 | 0.00034 | 0.95 | 4.0 | 1.5 | 1.5 | 0.54 | 0.63 | 0.46 | 0.83 | 0.84 | 0.42 | 0.44 | 0.080 | 0.025 | 0.16 | 0.0016 | 0.0024 | 0.0023 | 0.0053 |
| **Ga** | µg/kg | 5 | <LOD | 25 | 75 | 37 | 37 | 17 | 10 | 47 | <LOD | 33 | 66 | 59 | <LOD | <LOD | 37 | <LOD | <LOD | <LOD | <LOD |
| **Gd** | µg/kg | 1 | <LOD | 14 | 49 | 50 | 90 | 6.7 | 4.3 | 18 | 2.3 | 6.3 | 36 | 29 | 1.4 | 2.4 | 13 | <LOD | <LOD | <LOD | 2.0 |
| **Ge** | µg/kg | 10 | <LOD | <LOD | 16 | 13 | 23 | 13 | <LOD | 22 | <LOD | 18 | 19 | 12 | 18 | 45 | 36 | <LOD | <LOD | <LOD | 11 |
| **Hf** | µg/kg | 1 | <LOD | 3.3 | 4.3 | 18 | 44 | 12 | 2.3 | 5.3 | <LOD | 2.7 | 3.6 | 5.0 | <LOD | <LOD | 17 | <LOD | <LOD | <LOD | 5.9 |
| **Hg** | µg/kg | 1 | <LOD | 1.8 | 2.3 | 5.3 | 2.3 | 2.7 | 1.4 | 3.6 | 1.9 | 5.3 | 12 | 7.4 | 2.7 | 7.0 | 38 | 4.7 | <LOD | 1.4 | 3.9 |
| **Ho** | µg/kg | 1 | <LOD | 1.8 | 6.5 | 30 | 60 | 1.2 | <LOD | 2.8 | <LOD | <LOD | 4.9 | 4.6 | <LOD | <LOD | 2.0 | <LOD | <LOD | <LOD | <LOD |
| **I** | mg/kg | 0.25 | 0.55 | 0.86 | 5.8 | 3.7 | 4.6 | 1.1 | 0.49 | 0.37 | 0.50 | 1.5 | 0.65 | 0.50 | 0.66 | 480 | 11 | 1.4 | 0.72 | 0.13 | 5.0 |
| **Ir** | µg/kg | 1 | <LOD | <LOD | <LOD | <LOD | <LOD | <LOD | <LOD | <LOD | <LOD | <LOD | <LOD | <LOD | <LOD | <LOD | <LOD | <LOD | <LOD | <LOD | <LOD |
| **K** | g/kg | 0.005 | 1.4 | 17 | 15 | 10 | 15 | 15 | 15 | 28 | 11 | 4.3 | 20 | 14 | 7.2 | 58 | 10 | 0.50 | 0.031 | 130 | 0.26 |
| **La** | µg/kg | 1 | 1.4 | 100 | 260 | 120 | 140 | 54 | 31 | 140 | 8.2 | 40 | 220 | 120 | 8.1 | 8.9 | 79 | <LOD | 3.8 | 5.4 | 11 |
| **Li** | µg/kg | 30 | <LOD | 180 | 2,500 | 190 | 180 | 71 | 99 | 91 | 41 | 170 | 750 | 760 | 41 | 1,100 | 2,500 | 90 | <LOD | <LOD | <LOD |
| **Lu** | µg/kg | 1 | <LOD | <LOD | 2.1 | 30 | 59 | <LOD | <LOD | 1.1 | <LOD | <LOD | 1.9 | 1.7 | <LOD | <LOD | <LOD | <LOD | <LOD | <LOD | <LOD |
| **Mg** | g/kg | 0.0002 | 0.035 | 4.0 | 7.3 | 2.9 | 3.8 | 2.9 | 3.8 | 2.9 | 4.4 | 6.1 | 2.8 | 3.3 | 1.0 | 13 | 5.2 | 1.5 | 0.016 | 0.44 | 0.028 |
| **Mn** | mg/kg | 0.02 | 0.14 | 37 | 59 | 29 | 42 | 30 | 26 | 160 | 78 | 52 | 62 | 68 | 21 | 2.9 | 15 | 0.082 | 0.042 | 0.13 | 0.43 |
| **Mo** | mg/kg | 0.005 | <LOD | 0.17 | 0.28 | 0.12 | 0.16 | 0.12 | 0.10 | 0.87 | 0.56 | 0.25 | 0.60 | 0.42 | 1.1 | 0.20 | 2.7 | 0.010 | 0.013 | <LOD | 0.033 |
| **Na** | g/kg | 0.002 | 3.1 | 8.3 | 8.8 | 3.2 | 5.9 | 6.3 | 3.5 | 0.34 | 0.66 | 7.1 | 6.4 | 10 | 2.2 | 81 | 32 | 2.3 | 2.1 | 0.18 | 0.064 |
| **Nb** | µg/kg | 1 | <LOD | 10 | 19 | 39 | 56 | 5.8 | 2.9 | 9.2 | 4.6 | 18 | 21 | 16 | 1.6 | 3.1 | 14 | <LOD | <LOD | <LOD | 2.5 |
| **Nd** | µg/kg | 1 | 1.3 | 88 | 290 | 150 | 220 | 49 | 29 | 97 | 8.2 | 39 | 220 | 120 | 7.0 | 61 | 74 | <LOD | <LOD | 1.5 | 8.1 |
| **Ni** | µg/kg | 50 | <LOD | 1,700 | 5,300 | 560 | 870 | 610 | 930 | 360 | 140 | 530 | 1,100 | 1,400 | 760 | 190 | 1,500 | 140 | <LOD | 130 | 510 |
| **Os** | µg/kg | 5 | <LOD | <LOD | <LOD | <LOD | <LOD | <LOD | <LOD | <LOD | <LOD | <LOD | <LOD | <LOD | <LOD | <LOD | <LOD | <LOD | <LOD | <LOD | <LOD |
| **P** | g/kg | 0.001 | 0.80 | 17 | 17 | 11 | 15 | 11 | 15 | 10 | 15 | 17 | 4.2 | 6.4 | 4.8 | 3.2 | 9.0 | 0.84 | 0.16 | 0.010 | 4.2 |
| **Pb** | µg/kg | 1 | 2.2 | 290 | 290 | 530 | 900 | 120 | 72 | 290 | 58 | 890 | 650 | 360 | 16 | 27 | 310 | 22 | 2.9 | 6.6 | 16 |
| **Pd** | µg/kg | 5 | <LOD | <LOD | <LOD | 17 | 37 | 6.2 | 6.6 | <LOD | <LOD | <LOD | <LOD | <LOD | <LOD | <LOD | 15 | <LOD | <LOD | <LOD | <LOD |
| **Pr** | µg/kg | 1 | <LOD | 23 | 70 | 34 | 42 | 12 | 7.5 | 24 | 1.8 | 10 | 54 | 27 | 1.4 | 1.9 | 17 | <LOD | <LOD | <LOD | 1.9 |
| **Pt** | µg/kg | 1 | <LOD | <LOD | <LOD | <LOD | <LOD | <LOD | <LOD | <LOD | <LOD | <LOD | <LOD | <LOD | <LOD | <LOD | 1.5 | 1.8 | <LOD | <LOD | 1.3 |
| **Rb** | mg/kg | 0.01 | 0.72 | 1.8 | 3.8 | 0.66 | 0.93 | 0.59 | 0.93 | 9.1 | 2.1 | 0.50 | 27 | 16 | 2.7 | 16 | 9.2 | 0.034 | <LOD | 5.3 | 0.14 |
| **Re** | µg/kg | 1 | <LOD | <LOD | <LOD | <LOD | <LOD | <LOD | <LOD | <LOD | <LOD | <LOD | 2.0 | 3.5 | <LOD | 4.1 | <LOD | <LOD | <LOD | <LOD | <LOD |
| **Rh** | µg/kg | 5 | <LOD | <LOD | <LOD | <LOD | <LOD | <LOD | <LOD | <LOD | <LOD | <LOD | <LOD | <LOD | <LOD | 6.7 | <LOD | <LOD | <LOD | <LOD | <LOD |
| **Ru** | µg/kg | 5 | <LOD | <LOD | <LOD | <LOD | <LOD | <LOD | <LOD | <LOD | <LOD | <LOD | <LOD | <LOD | <LOD | <LOD | <LOD | <LOD | <LOD | <LOD | <LOD |
| **S** | g/kg | 0.005 | 4.1 | 13 | 11 | 7.0 | 11 | 9.1 | 10 | 5.9 | 10 | 8.1 | 3.6 | 3.5 | 3.4 | 11 | 18 | 6.8 | 5.8 | 51 | 1.1 |
| **Sb** | µg/kg | 1 | 5.4 | 10 | 31 | 23 | 24 | 5.7 | 8.7 | 7.0 | 4.0 | 46 | 26 | 13 | 2.5 | 18 | 10 | 3.6 | <LOD | <LOD | 25 |
| **Sc** | µg/kg | 1 | <LOD | 51 | 54 | 280 | 350 | 17 | 42 | 33 | 2.8 | 10 | 38 | 56 | 2.3 | 18 | 28 | 1.3 | 1.5 | <LOD | 2.0 |
| **Se** | mg/kg | 0.05 | <LOD | 0.11 | 0.27 | 0.079 | 0.15 | 0.059 | 0.081 | 0.070 | <LOD | 0.19 | <LOD | 0.19 | 0.33 | 0.068 | 2.4 | 0.29 | 0.55 | <LOD | 94 |
| **Si** | mg/kg | 5 | 11 | 71 | 95 | 95 | 170 | 170 | 80 | 430 | 560 | 110 | 170 | 150 | 64 | 10 | 4,200 | 80 | 47 | <LOD | 6,700 |
| **Sm** | µg/kg | 1 | <LOD | 16 | 59 | 40 | 68 | 7.7 | 5.2 | 19 | 1.8 | 5.9 | 43 | 28 | <LOD | 2.7 | 15 | <LOD | <LOD | <LOD | 1.8 |
| **Sn** | µg/kg | 1 | 5.5 | 8.9 | 27 | 82 | 200 | 27 | 8.7 | 20 | 21 | 98 | 29 | 25 | 3.3 | 8.3 | 13 | 7.3 | 5.1 | 2.2 | 45 |
| **Sr** | mg/kg | 0.02 | 0.24 | 32 | 230 | 23 | 22 | 13 | 15 | 8.8 | 17 | 32 | 30 | 33 | 2.8 | 510 | 28 | 1.2 | 0.70 | 0.40 | 2.3 |
| **Ta** | µg/kg | 50 | <LOD | <LOD | <LOD | <LOD | <LOD | <LOD | <LOD | <LOD | <LOD | <LOD | <LOD | <LOD | <LOD | <LOD | <LOD | <LOD | <LOD | <LOD | <LOD |
| **Tb** | µg/kg | 1 | <LOD | 1.9 | 6.0 | 12 | 22 | 1.1 | <LOD | 2.7 | <LOD | <LOD | 5.0 | 4.2 | <LOD | <LOD | 1.8 | <LOD | <LOD | <LOD | <LOD |
| **Te** | µg/kg | 5 | <LOD | <LOD | <LOD | <LOD | <LOD | <LOD | <LOD | <LOD | <LOD | <LOD | <LOD | <LOD | 8.9 | <LOD | <LOD | <LOD | <LOD | <LOD | <LOD |
| **Th** | µg/kg | 1 | <LOD | 58 | 82 | 860 | 1,400 | 55 | 30 | 39 | 4.5 | 11 | 65 | 36 | 1.3 | 3.7 | 23 | 1.0 | <LOD | <LOD | 2.3 |
| **Ti** | mg/kg | 0.02 | 0.029 | 8.7 | 4.9 | 15 | 22 | 2.9 | 3.2 | 6.3 | 5.5 | 8.7 | 7.1 | 11 | 0.24 | 0.44 | 15 | 0.083 | 0.054 | 0.027 | 0.11 |
| **Tl** | µg/kg | 1 | <LOD | 200 | 77 | 19 | 20 | 110 | 110 | 27 | 3.4 | 14 | 30 | 17 | 1.9 | <LOD | 10 | <LOD | <LOD | <LOD | <LOD |
| **Tm** | µg/kg | 1 | <LOD | <LOD | 2.2 | 23 | 46 | <LOD | <LOD | 1.1 | <LOD | <LOD | 2.4 | 1.7 | <LOD | <LOD | <LOD | <LOD | <LOD | <LOD | <LOD |
| **U** | µg/kg | 1 | 1.1 | 160 | 130 | 39 | 65 | 54 | 210 | 13 | 4.3 | 21 | 40 | 32 | 3.0 | 140 | 76 | 4.3 | 2.2 | <LOD | 2.9 |
| **W** | µg/kg | 1 | <LOD | 52 | 54 | 84 | 120 | 31 | 22 | 180 | 3.9 | 130 | 33 | 67 | 73 | 4.4 | 3,100 | 20 | 8.6 | 110 | 17 |
| **V** | µg/kg | 5 | <LOD | 280 | 590 | 220 | 260 | 88 | 160 | 390 | 59 | 200 | 400 | 540 | 21 | 130 | 2,000 | 29 | 25 | <LOD | 26 |
| **Y** | µg/kg | 1 | <LOD | 52 | 180 | 830 | 1,200 | 21 | 14 | 69 | 13 | 21 | 140 | 120 | 11 | 20 | 66 | <LOD | 5.0 | <LOD | 19 |
| **Yb** | µg/kg | 1 | <LOD | 5.0 | 13 | 180 | 350 | 3.5 | 2.0 | 7.1 | <LOD | 2.9 | 12 | 10 | <LOD | 1.9 | 5.2 | <LOD | <LOD | <LOD | 1.1 |
| **Zn** | mg/kg | 0.1 | 0.13 | 15 | 21 | 26 | 25 | 11 | 6.9 | 26 | 24 | 13 | 32 | 25 | 44 | 9.3 | 52 | 0.26 | 1.1 | <LOD | 1.0 |
| **Zr** | µg/kg | 1 | 2.5 | 370 | 180 | 1,500 | 2,500 | 440 | 390 | 130 | 15 | 120 | 170 | 260 | 25 | 14 | 750 | 4.7 | 3.8 | 3.2 | 260 |
| **REE** | mg/kg | - | 0.012 | 0.58 | 1.6 | 2.3 | 3.4 | 0.29 | 0.21 | 0.91 | 0.056 | 0.21 | 1.3 | 0.81 | 0.045 | 0.13 | 0.48 | 0.010 | 0.017 | 0.020 | 0.11 |

**Table S2.C** Elemental concentrations in the 9 investigated ***marine fish-oil*** food supplements (MF01 – MF09). Note that units have been adapted to the measured concentrations.

| **Element** | **Unit** | **LOD** | **MF01** | **MF02** | **MF03** | **MF04** | **MF05** | **MF06** | **MF07** | **MF08** | **MF09** |
| --- | --- | --- | --- | --- | --- | --- | --- | --- | --- | --- | --- |
| **Ag** | µg/kg | 1 | 1.0 | <LOD | <LOD | 4.8 | 1.1 | <LOD | <LOD | <LOD | 2.0 |
| **Al** | mg/kg | 0.2 | 2.0 | 2.0 | 7.4 | 160 | 4.0 | 0.60 | 0.61 | 0.42 | 6.1 |
| **As** | µg/kg | 2 | 13 | 6.4 | 17 | 110 | <LOD | 5.1 | 5.0 | 4.4 | 7.2 |
| **Au** | µg/kg | 1 | <LOD | <LOD | <LOD | <LOD | <LOD | <LOD | <LOD | <LOD | <LOD |
| **B** | mg/kg | 0.5 | 1.4 | 1.4 | 0.80 | 6.1 | <LOD | 0.60 | <LOD | <LOD | <LOD |
| **Ba** | mg/kg | 0.2 | <LOD | <LOD | <LOD | 3.2 | 0.24 | <LOD | <LOD | <LOD | <LOD |
| **Be** | µg/kg | 1 | <LOD | <LOD | <LOD | 16 | <LOD | <LOD | <LOD | <LOD | <LOD |
| **Bi** | µg/kg | 1 | 2.7 | 1.4 | 9.4 | 4.2 | <LOD | <LOD | <LOD | <LOD | <LOD |
| **Br** | mg/kg | 0.5 | 9.6 | 7.4 | 8.1 | 6.5 | <LOD | 0.59 | 2.3 | 2.2 | 0.95 |
| **Ca** | g/kg | 0.002 | 0.076 | 0.025 | 0.037 | 120 | 0.23 | 0.0077 | 0.0036 | 0.0079 | 0.27 |
| **Cd** | µg/kg | 1 | 1.9 | <LOD | <LOD | 190 | 1.2 | <LOD | <LOD | <LOD | <LOD |
| **Ce** | µg/kg | 1 | 1.7 | 4.8 | <LOD | 350 | 1.7 | <LOD | <LOD | <LOD | 6.4 |
| **Co** | µg/kg | 1 | 17 | 2.0 | <LOD | 1,400 | <LOD | 1.9 | 1.4 | 1.9 | 1.1 |
| **Cr** | mg/kg | 0.001 | 0.40 | 0.24 | 0.095 | 4.0 | 0.032 | 0.058 | 0.010 | 0.050 | 0.055 |
| **Cs** | µg/kg | 1 | <LOD | <LOD | <LOD | 43 | <LOD | 5.7 | <LOD | <LOD | <LOD |
| **Cu** | mg/kg | 0.005 | 6.4 | 0.21 | 0.049 | 910 | 0.057 | 0.038 | <LOD | <LOD | 1.4 |
| **Dy** | µg/kg | 1 | <LOD | <LOD | <LOD | 53 | <LOD | <LOD | <LOD | <LOD | <LOD |
| **Er** | µg/kg | 1 | <LOD | <LOD | <LOD | 32 | <LOD | <LOD | <LOD | <LOD | <LOD |
| **Eu** | µg/kg | 1 | <LOD | <LOD | <LOD | 12 | <LOD | <LOD | <LOD | <LOD | <LOD |
| **Fe** | g/kg | 0.0002 | 0.10 | 0.0012 | 0.00059 | 32 | 0.0010 | 0.00043 | <LOD | 0.00049 | 0.0023 |
| **Ga** | µg/kg | 5 | <LOD | <LOD | <LOD | 160 | <LOD | <LOD | <LOD | <LOD | <LOD |
| **Gd** | µg/kg | 1 | <LOD | <LOD | <LOD | 60 | <LOD | <LOD | <LOD | <LOD | <LOD |
| **Ge** | µg/kg | 10 | <LOD | <LOD | <LOD | <LOD | <LOD | <LOD | <LOD | <LOD | <LOD |
| **Hf** | µg/kg | 1 | <LOD | <LOD | <LOD | 4.6 | 2.0 | <LOD | <LOD | <LOD | 1.9 |
| **Hg** | µg/kg | 1 | 2.8 | <LOD | 1.1 | 2.6 | <LOD | <LOD | <LOD | <LOD | <LOD |
| **Ho** | µg/kg | 1 | <LOD | <LOD | <LOD | 11 | <LOD | <LOD | <LOD | <LOD | <LOD |
| **I** | mg/kg | 0.25 | 76 | 8.2 | 7.0 | 19 | 0.78 | 3.0 | 26 | 7.0 | 10 |
| **Ir** | µg/kg | 1 | <LOD | <LOD | <LOD | <LOD | <LOD | <LOD | <LOD | <LOD | <LOD |
| **K** | g/kg | 0.005 | 0.024 | 0.041 | 0.0095 | 0.21 | 0.028 | 0.0052 | <LOD | 0.034 | 1.6 |
| **La** | µg/kg | 1 | 1.5 | 2.9 | <LOD | 280 | <LOD | <LOD | <LOD | <LOD | 3.2 |
| **Li** | µg/kg | 30 | 63 | 93 | 75 | 430 | <LOD | 130 | <LOD | <LOD | <LOD |
| **Lu** | µg/kg | 1 | <LOD | <LOD | <LOD | 3.6 | <LOD | <LOD | <LOD | <LOD | <LOD |
| **Mg** | g/kg | 0.0002 | 1.4 | 0.012 | 0.0038 | 63 | 0.020 | 0.00076 | 0.00060 | 0.0075 | 0.0051 |
| **Mn** | mg/kg | 0.02 | 12 | 0.82 | 0.029 | 73 | 0.044 | <LOD | <LOD | 0.025 | 0.085 |
| **Mo** | mg/kg | 0.005 | 0.23 | 0.010 | <LOD | 0.24 | 0.010 | <LOD | <LOD | <LOD | 0.026 |
| **Na** | g/kg | 0.002 | 0.56 | 0.93 | 0.68 | 0.70 | 0.77 | 0.39 | 0.0033 | 0.0034 | 4.3 |
| **Nb** | µg/kg | 1 | 1.5 | 1.4 | <LOD | 62 | <LOD | <LOD | <LOD | <LOD | <LOD |
| **Nd** | µg/kg | 1 | 12 | 2.2 | 1.1 | 250 | <LOD | <LOD | 1.8 | <LOD | 3.2 |
| **Ni** | µg/kg | 50 | <LOD | <LOD | <LOD | 2,100 | <LOD | <LOD | <LOD | <LOD | <LOD |
| **Os** | µg/kg | 5 | <LOD | <LOD | <LOD | <LOD | <LOD | <LOD | <LOD | <LOD | <LOD |
| **P** | g/kg | 0.001 | 0.067 | 0.019 | 0.013 | 0.52 | 0.68 | 0.077 | 0.0081 | 0.059 | 2.2 |
| **Pb** | µg/kg | 1 | 7.3 | 13 | 3.3 | 430 | 1.3 | 1.6 | <LOD | <LOD | 110 |
| **Pd** | µg/kg | 5 | <LOD | <LOD | <LOD | 9.4 | <LOD | <LOD | <LOD | <LOD | 32 |
| **Pr** | µg/kg | 1 | <LOD | <LOD | <LOD | 60 | <LOD | <LOD | <LOD | <LOD | <LOD |
| **Pt** | µg/kg | 1 | <LOD | 1.0 | <LOD | <LOD | <LOD | <LOD | <LOD | <LOD | <LOD |
| **Rb** | mg/kg | 0.01 | 0.055 | 0.10 | <LOD | 0.47 | 0.021 | <LOD | <LOD | 0.031 | 0.89 |
| **Re** | µg/kg | 1 | <LOD | <LOD | <LOD | <LOD | <LOD | <LOD | <LOD | <LOD | <LOD |
| **Rh** | µg/kg | 5 | <LOD | <LOD | <LOD | 6.6 | <LOD | <LOD | <LOD | <LOD | <LOD |
| **Ru** | µg/kg | 5 | <LOD | <LOD | <LOD | <LOD | <LOD | <LOD | <LOD | <LOD | <LOD |
| **S** | g/kg | 0.005 | 0.85 | 0.87 | 0.65 | 2.0 | 0.26 | 0.49 | <LOD | 0.0068 | 0.16 |
| **Sb** | µg/kg | 1 | 1.5 | 1.4 | 1.1 | 17 | 3.0 | 2.0 | <LOD | 1.3 | 12 |
| **Sc** | µg/kg | 1 | 1.1 | <LOD | <LOD | 36 | <LOD | <LOD | <LOD | <LOD | 1.1 |
| **Se** | mg/kg | 0.05 | 0.47 | 0.050 | 0.073 | 50 | <LOD | 0.070 | <LOD | <LOD | <LOD |
| **Si** | mg/kg | 5 | 34 | 36 | 18 | 81 | 50 | 12 | 7.8 | 5.8 | 110 |
| **Sm** | µg/kg | 1 | <LOD | <LOD | <LOD | 53 | <LOD | <LOD | <LOD | <LOD | <LOD |
| **Sn** | µg/kg | 1 | <LOD | 5.0 | <LOD | 74 | 2.0 | 1.8 | <LOD | <LOD | 24 |
| **Sr** | mg/kg | 0.02 | 0.057 | 0.062 | 0.10 | 39 | 0.57 | <LOD | <LOD | <LOD | 0.74 |
| **Ta** | µg/kg | 50 | <LOD | <LOD | <LOD | 2,400 | <LOD | <LOD | <LOD | <LOD | <LOD |
| **Tb** | µg/kg | 1 | <LOD | <LOD | <LOD | 9.0 | <LOD | <LOD | <LOD | <LOD | <LOD |
| **Te** | µg/kg | 5 | <LOD | <LOD | <LOD | <LOD | <LOD | <LOD | <LOD | <LOD | <LOD |
| **Th** | µg/kg | 1 | 1.9 | <LOD | <LOD | 62 | <LOD | <LOD | <LOD | <LOD | 1.3 |
| **Ti** | mg/kg | 0.02 | 0.71 | 0.63 | 0.051 | 3.2 | 0.075 | 0.056 | 0.083 | 0.067 | 0.10 |
| **Tl** | µg/kg | 1 | <LOD | <LOD | <LOD | 49 | <LOD | <LOD | <LOD | <LOD | <LOD |
| **Tm** | µg/kg | 1 | <LOD | <LOD | <LOD | 3.9 | <LOD | <LOD | <LOD | <LOD | <LOD |
| **U** | µg/kg | 1 | 2.2 | 1.2 | 3.1 | 1,500 | 5.4 | 1.5 | <LOD | <LOD | 13 |
| **W** | µg/kg | 1 | 1.9 | 1.9 | <LOD | 15 | <LOD | 1.2 | <LOD | <LOD | 8.3 |
| **V** | µg/kg | 5 | 23 | <LOD | <LOD | 4,000 | 7.2 | 29 | <LOD | <LOD | 7.9 |
| **Y** | µg/kg | 1 | 1.5 | 1.6 | <LOD | 460 | 1.1 | <LOD | <LOD | <LOD | 3.6 |
| **Yb** | µg/kg | 1 | <LOD | <LOD | <LOD | 23 | <LOD | <LOD | <LOD | <LOD | 1.0 |
| **Zn** | mg/kg | 0.1 | 62 | 0.28 | 0.12 | 7,700 | 0.24 | <LOD | <LOD | <LOD | 2.1 |
| **Zr** | µg/kg | 1 | 18 | 9.3 | 7.1 | 200 | 93 | 6.5 | <LOD | <LOD | 100 |
| **REE** | mg/kg | - | 0.024 | 0.018 | 0.0086 | 1.7 | 0.010 | 0.0080 | 0.0093 | 0.0080 | 0.023 |

**Table S2.D, part 1 (of 3)** Elemental concentrations in the 62 investigated ***terrestrial plant-based*** food supplements (TPB01-TPB62). Note that units have been adapted to the measured concentrations.

| **Element** | **Unit** | **LOD** | **TPB01** | **TPB02** | **TPB03** | **TPB04** | **TPB05** | **TPB06** | **TPB07** | **TPB08** | **TPB09** | **TPB10** | **TPB11** | **TPB12** | **TPB13** | **TPB14** | **TPB15** | **TPB16** | **TPB17** | **TPB18** | **TPB19** | **TPB20** | **TPB21** |
| --- | --- | --- | --- | --- | --- | --- | --- | --- | --- | --- | --- | --- | --- | --- | --- | --- | --- | --- | --- | --- | --- | --- | --- |
| **Ag** | µg/kg | 1 | <LOD | 1.1 | 1.9 | 3.2 | 2.8 | <LOD | 2.0 | 7.2 | <LOD | 1.7 | <LOD | 4.1 | <LOD | <LOD | <LOD | 1.3 | 2.0 | 1.8 | 2.5 | <LOD | 33 |
| **Al** | mg/kg | 0.2 | 2.4 | 26 | 110 | 9.3 | 28 | 52 | 750 | 890 | 110 | 2.6 | 2.3 | 1.4 | 270 | 8.4 | 3.2 | 35 | 250 | 26 | 200 | 1.3 | 210 |
| **As** | µg/kg | 2 | 7.5 | 79 | 54 | 12 | 150 | 230 | 260 | 160 | 40 | 41 | 180 | 6.0 | 120 | 56 | 2.3 | 63 | 33 | 29 | 27 | 18 | 31 |
| **Au** | µg/kg | 1 | <LOD | <LOD | <LOD | <LOD | <LOD | <LOD | <LOD | <LOD | <LOD | <LOD | <LOD | <LOD | <LOD | <LOD | <LOD | <LOD | <LOD | <LOD | <LOD | <LOD | <LOD |
| **B** | mg/kg | 0.5 | 1.4 | 42 | 5.9 | 2.4 | 8.5 | 12 | 13 | 8.7 | 20 | 10 | 20 | 22 | 1.3 | 27 | 23 | 2.3 | 2.9 | 1.7 | 4.1 | 13 | 4.4 |
| **Ba** | mg/kg | 0.2 | 0.68 | 11 | 1.5 | 0.41 | 3.7 | 3.1 | 5.0 | 16 | 0.64 | 1.8 | 1.3 | 5.1 | 0.64 | 3.2 | 36 | 2.0 | 7.0 | 0.81 | 4.9 | 2.5 | 16 |
| **Be** | µg/kg | 1 | <LOD | 3.9 | 12 | 1.3 | 42 | 20 | 24 | 19 | 2.0 | 1.1 | 5.9 | <LOD | 4.8 | 2.9 | <LOD | 2.1 | 7.3 | <LOD | 3.9 | <LOD | 7.1 |
| **Bi** | µg/kg | 1 | <LOD | <LOD | 2.5 | 28 | 8.5 | 2.6 | 11 | 20 | <LOD | <LOD | 1.5 | 0.96 | 1.1 | <LOD | <LOD | <LOD | 7.0 | 1.7 | <LOD | <LOD | <LOD |
| **Br** | mg/kg | 0.5 | <LOD | 4.1 | 2.6 | 2.0 | 6.8 | 0.91 | 1.4 | 1.6 | 3.9 | 45 | 0.56 | 5.5 | <LOD | 7.0 | 8.2 | 7.2 | 7.6 | 6.7 | 34 | 3.4 | 23 |
| **Ca** | g/kg | 0.002 | 3.8 | 2.4 | 110 | 2.7 | 120 | 58 | 2.1 | 3.4 | 2.9 | 0.48 | 0.42 | 5.7 | 0.62 | 0.29 | 8.9 | 3.3 | 0.74 | 0.64 | 2.0 | 0.59 | 1.8 |
| **Cd** | µg/kg | 1 | 16 | 17 | 170 | 82 | 260 | 16 | 19 | 48 | 13 | 13 | 12 | 11 | <LOD | 1.9 | 2.0 | 26 | 14 | 4.4 | 12 | 48 | 29 |
| **Ce** | µg/kg | 1 | 8.3 | 37 | 490 | 18 | 120 | 140 | 88 | 480 | 21 | 4.5 | 2.5 | 13 | 11 | 24 | 58 | 58 | 100 | 12 | 220 | 1.7 | 410 |
| **Co** | µg/kg | 1 | 19 | 190 | 44 | 290 | 550 | 160 | 440 | 380 | 2,500 | 14 | 75 | 25 | 16 | 60 | 21 | 78 | 65 | 18 | 410 | 160 | 290 |
| **Cr** | mg/kg | 0.001 | 0.22 | 0.15 | 0.86 | 39 | 2.4 | 0.50 | 2.8 | 1.7 | 48 | 0.22 | 0.19 | 0.049 | 1.9 | 0.66 | 0.068 | 0.18 | 0.27 | 0.51 | 0.76 | 0.032 | 0.84 |
| **Cs** | µg/kg | 1 | 2.5 | 32 | 13 | 63 | 86 | 3.7 | 530 | 370 | 36 | 8.6 | 13 | 34 | 2.3 | 59 | 770 | 17 | 35 | 13 | 6.9 | 19 | 13 |
| **Cu** | mg/kg | 0.005 | 0.54 | 31 | 1.0 | 0.89 | 9.0 | 440 | 13 | 26 | 1,000 | 2.3 | 4.5 | 4.1 | 0.35 | 4.3 | 2.6 | 2.4 | 1.6 | 0.53 | 31 | 19 | 4.7 |
| **Dy** | µg/kg | 1 | 1.4 | 3.0 | 58 | 1.2 | 25 | 31 | 10 | 40 | 2.3 | <LOD | <LOD | 0.84 | <LOD | 2.9 | 10 | 7.0 | 10 | 1.5 | 36 | <LOD | 22 |
| **Er** | µg/kg | 1 | <LOD | 1.5 | 35 | 1.1 | 17 | 19 | 7.4 | 23 | 1.5 | <LOD | <LOD | <LOD | <LOD | 1.5 | 4.3 | 3.4 | 5.1 | <LOD | 17 | <LOD | 12 |
| **Eu** | µg/kg | 1 | <LOD | <LOD | 14 | <LOD | 4.3 | 5.0 | 1.0 | 8.2 | <LOD | <LOD | <LOD | <LOD | <LOD | <LOD | 2.6 | 1.9 | 1.2 | <LOD | 9.3 | <LOD | 5.1 |
| **Fe** | g/kg | 0.0002 | 0.0079 | 0.029 | 0.11 | 0.013 | 0.045 | 0.069 | 0.061 | 0.55 | 16 | 0.020 | 0.014 | 0.016 | 0.055 | 0.026 | 0.0072 | 0.039 | 0.030 | 0.011 | 0.57 | 0.092 | 0.24 |
| **Ga** | µg/kg | 5 | <LOD | 6.2 | 27 | <LOD | 13 | 13 | 22 | 44 | 31 | <LOD | <LOD | <LOD | 5.6 | <LOD | <LOD | 10 | 5.7 | <LOD | 55 | <LOD | 65 |
| **Gd** | µg/kg | 1 | 1.3 | 3.4 | 64 | 1.5 | 26 | 32 | 9.2 | 45 | 2.2 | <LOD | <LOD | 1.1 | <LOD | 2.5 | 13 | 8.0 | 9.2 | 1.5 | 34 | <LOD | 35 |
| **Ge** | µg/kg | 10 | <LOD | <LOD | <LOD | 18 | <LOD | <LOD | <LOD | 20 | <LOD | <LOD | <LOD | 14 | <LOD | <LOD | <LOD | <LOD | <LOD | <LOD | 20 | <LOD | <LOD |
| **Hf** | µg/kg | 1 | <LOD | 5.7 | 48 | 5.2 | 2.0 | 13 | 10 | 36 | 7.2 | 5.0 | <LOD | <LOD | 6.5 | 7.2 | <LOD | 7.3 | 12 | 6.8 | 9.4 | <LOD | 2.9 |
| **Hg** | µg/kg | 1 | <LOD | <LOD | 1.6 | <LOD | 16 | <LOD | 10 | 5.0 | <LOD | <LOD | <LOD | 4.2 | 1.2 | <LOD | <LOD | 1.1 | 2.6 | <LOD | <LOD | <LOD | 1.3 |
| **Ho** | µg/kg | 1 | <LOD | <LOD | 12 | <LOD | 5.9 | 6.4 | 2.3 | 8.0 | <LOD | <LOD | <LOD | <LOD | <LOD | <LOD | 1.9 | 1.2 | 1.7 | <LOD | 7.0 | <LOD | 4.6 |
| **I** | mg/kg | 0.25 | 6.3 | 17 | 29 | 0.42 | 6.5 | 22 | 0.45 | 140 | 84 | 17 | 0.70 | 23 | 1.2 | 6.4 | 6.4 | 5.6 | 5.9 | 7.2 | 140 | 31 | 2.2 |
| **Ir** | µg/kg | 1 | <LOD | <LOD | <LOD | <LOD | <LOD | <LOD | <LOD | <LOD | <LOD | <LOD | <LOD | <LOD | <LOD | <LOD | <LOD | <LOD | <LOD | <LOD | <LOD | <LOD | <LOD |
| **K** | g/kg | 0.005 | 0.76 | 19 | 2.8 | 6.7 | 26 | 2.2 | 17 | 11 | 2.7 | 5.2 | 8.6 | 10 | 0.67 | 2.0 | 21 | 4.2 | 1.8 | 1.0 | 41 | 5.3 | 28 |
| **La** | µg/kg | 1 | 8.2 | 21 | 360 | 10 | 170 | 140 | 120 | 260 | 16 | 3.0 | 1.8 | 27 | 6.2 | 15 | 200 | 34 | 65 | 9.3 | 120 | 1.2 | 210 |
| **Li** | µg/kg | 30 | 32 | 150 | 130 | <LOD | 99 | 120 | 200 | 200 | 300 | 300 | 460 | 46 | 200 | 120 | 63 | 76 | 130 | 84 | 110 | <LOD | 100 |
| **Lu** | µg/kg | 1 | <LOD | <LOD | 4.6 | <LOD | 1.5 | 2.5 | 1.3 | 3.9 | <LOD | <LOD | <LOD | <LOD | <LOD | <LOD | <LOD | <LOD | <LOD | <LOD | 2.0 | <LOD | 1.2 |
| **Mg** | g/kg | 0.0002 | 0.22 | 2.0 | 1.5 | 1.2 | 4.0 | 1.3 | 2.7 | 4.2 | 260 | 0.87 | 1.3 | 1.6 | 0.31 | 0.45 | 2.7 | 3.8 | 0.60 | 0.44 | 5.5 | 4.1 | 3.3 |
| **Mn** | mg/kg | 0.02 | 1.5 | 8.8 | 17 | 5.0 | 41 | 890 | 510 | 730 | 2,300 | 5.9 | 5.7 | 31 | 1.1 | 1.8 | 53 | 33 | 190 | 31 | 83 | 17 | 38 |
| **Mo** | mg/kg | 0.005 | 0.031 | 0.80 | 0.24 | 0.047 | 0.13 | 0.15 | 0.064 | 1.1 | 25 | 0.052 | 0.078 | 0.17 | 0.054 | 0.067 | 0.043 | 0.46 | 0.088 | 0.080 | 0.082 | 1.8 | 0.17 |
| **Na** | g/kg | 0.002 | 0.67 | 0.68 | 2.5 | 0.28 | 1.5 | 2.8 | 1.0 | 0.30 | 13 | 3.0 | 1.6 | 0.25 | 3.0 | 2.0 | 5.8 | 1.0 | 0.56 | 2.2 | 0.80 | 0.23 | 0.68 |
| **Nb** | µg/kg | 1 | 1.8 | 5.6 | 650 | 6.0 | 4.3 | 20 | 7.1 | 20 | 39 | 2.9 | <LOD | <LOD | 190 | 26 | <LOD | 4.2 | 3.8 | 1.5 | 14 | <LOD | 23 |
| **Nd** | µg/kg | 1 | 6.9 | 16 | 280 | 8.3 | 100 | 110 | 41 | 220 | 16 | 4.5 | 2.2 | 12 | 4.7 | 14 | 75 | 33 | 46 | 7.2 | 140 | 3.1 | 190 |
| **Ni** | µg/kg | 50 | 110 | 3,100 | 450 | 1,700 | 4,100 | 1,100 | 9,300 | 3,900 | 2,600 | 290 | 400 | 760 | 670 | 620 | 350 | 250 | 1,100 | 470 | 680 | 740 | 750 |
| **Os** | µg/kg | 5 | <LOD | <LOD | <LOD | <LOD | <LOD | <LOD | <LOD | <LOD | <LOD | <LOD | <LOD | <LOD | <LOD | <LOD | <LOD | <LOD | <LOD | <LOD | <LOD | <LOD | <LOD |
| **P** | g/kg | 0.001 | 0.11 | 1.6 | 77 | 1.4 | 0.84 | 44 | 2.0 | 1.5 | 0.44 | 0.52 | 1.3 | 2.2 | 1.2 | 0.25 | 1.3 | 1.3 | 0.31 | 0.35 | 3.2 | 9.2 | 3.3 |
| **Pb** | µg/kg | 1 | 32 | 36 | 160 | 260 | 190 | 130 | 330 | 1,600 | 130 | 19 | 35 | 16 | 40 | 82 | 7.8 | 33 | 350 | 48 | 69 | 95 | 140 |
| **Pd** | µg/kg | 5 | <LOD | <LOD | 6.3 | 10 | <LOD | 6.2 | <LOD | 19 | <LOD | <LOD | <LOD | 60 | <LOD | <LOD | <LOD | <LOD | <LOD | <LOD | <LOD | <LOD | <LOD |
| **Pr** | µg/kg | 1 | 1.5 | 4.3 | 70 | 2.1 | 23 | 28 | 11 | 55 | 2.6 | 1.3 | <LOD | 2.5 | 1.4 | 3.3 | 20 | 8.1 | 13 | 1.8 | 28 | <LOD | 47 |
| **Pt** | µg/kg | 1 | <LOD | <LOD | <LOD | <LOD | <LOD | <LOD | <LOD | <LOD | <LOD | <LOD | <LOD | <LOD | <LOD | <LOD | <LOD | <LOD | <LOD | <LOD | <LOD | <LOD | <LOD |
| **Rb** | mg/kg | 0.01 | 0.60 | 10 | 2.9 | 12 | 26 | 1.9 | 110 | 71 | 6.6 | 2.5 | 4.0 | 7.0 | 0.22 | 3.7 | 69 | 5.5 | 5.8 | 3.5 | 4.4 | 13 | 9.1 |
| **Re** | µg/kg | 1 | <LOD | 5.0 | <LOD | <LOD | 2.9 | <LOD | <LOD | <LOD | <LOD | <LOD | <LOD | <LOD | <LOD | <LOD | <LOD | <LOD | <LOD | <LOD | <LOD | <LOD | <LOD |
| **Rh** | µg/kg | 5 | <LOD | <LOD | <LOD | <LOD | <LOD | <LOD | <LOD | <LOD | 7.5 | <LOD | <LOD | <LOD | <LOD | <LOD | <LOD | <LOD | <LOD | <LOD | <LOD | <LOD | <LOD |
| **Ru** | µg/kg | 5 | <LOD | <LOD | <LOD | <LOD | <LOD | <LOD | <LOD | <LOD | <LOD | <LOD | <LOD | <LOD | <LOD | <LOD | <LOD | <LOD | <LOD | <LOD | <LOD | <LOD | <LOD |
| **S** | g/kg | 0.005 | 0.18 | 1.4 | 0.52 | 0.41 | 0.81 | 1.1 | 2.0 | 1.2 | 3.0 | 0.40 | 1.3 | 1.6 | 1.9 | 0.93 | 0.86 | 1.4 | 0.38 | 2.9 | 1.8 | 4.5 | 1.8 |
| **Sb** | µg/kg | 1 | <LOD | 7.4 | 180 | 3.7 | 15 | 73 | 24 | 35 | 7.4 | 4.0 | 4.7 | 2.0 | 13 | 6.7 | <LOD | 1.7 | 9.0 | 4.6 | 2.3 | <LOD | 3.1 |
| **Sc** | µg/kg | 1 | 2.3 | 5.3 | 28 | 1.6 | 12 | 24 | 11 | 48 | 10 | <LOD | <LOD | <LOD | 5.5 | 1.4 | <LOD | 8.3 | 8.5 | <LOD | 67 | 1.2 | 58 |
| **Se** | mg/kg | 0.05 | <LOD | <LOD | 0.055 | 40 | 0.070 | 0.16 | 0.093 | 1.3 | 50 | <LOD | 0.086 | 0.21 | <LOD | <LOD | 0.070 | 0.13 | <LOD | 0.060 | 1.7 | 0.50 | 0.14 |
| **Si** | mg/kg | 5 | 97 | 1,500 | 960 | 1,300 | 550 | 360 | 1,200 | 520 | 120 | 1,200 | 290 | 31 | 130 | 1,000 | 110 | 1,000 | 890 | 810 | 750 | 7.6 | 680 |
| **Sm** | µg/kg | 1 | 1.1 | 3.9 | 59 | 2.1 | 19 | 25 | 8.9 | 44 | 2.2 | <LOD | <LOD | 1.6 | <LOD | 2.7 | 12 | 7.5 | 11 | 1.4 | 31 | <LOD | 38 |
| **Sn** | µg/kg | 1 | <LOD | 25 | 26 | 32 | 45 | 36 | 230 | 120 | 52 | 6.1 | 12 | 1.6 | 45 | 53 | <LOD | <LOD | 37 | 18 | 5.5 | <LOD | 12 |
| **Sr** | mg/kg | 0.02 | 2.5 | 17 | 55 | 1.2 | 49 | 23 | 4.1 | 6.3 | 6.2 | 3.9 | 7.8 | 19 | 5.4 | 1.3 | 79 | 6.1 | 3.0 | 1.5 | 10 | 5.1 | 18 |
| **Ta** | µg/kg | 50 | <LOD | <LOD | 84 | 78 | <LOD | 56 | 86 | <LOD | 660 | <LOD | <LOD | <LOD | 120 | <LOD | <LOD | <LOD | <LOD | <LOD | <LOD | <LOD | 320 |
| **Tb** | µg/kg | 1 | <LOD | <LOD | 9.1 | <LOD | 3.8 | 4.3 | 1.6 | 6.4 | <LOD | <LOD | <LOD | <LOD | <LOD | <LOD | 1.7 | <LOD | 1.5 | <LOD | 5.3 | <LOD | 4.3 |
| **Te** | µg/kg | 5 | <LOD | <LOD | <LOD | <LOD | <LOD | <LOD | <LOD | <LOD | <LOD | <LOD | <LOD | <LOD | <LOD | <LOD | <LOD | <LOD | <LOD | <LOD | <LOD | <LOD | <LOD |
| **Th** | µg/kg | 1 | 1.5 | 4.5 | 22 | 3.2 | 7.6 | 17 | 13 | 41 | 4.6 | <LOD | <LOD | <LOD | 1.9 | 4.1 | <LOD | 4.7 | 7.5 | 2.0 | 17 | 1.9 | 60 |
| **Ti** | mg/kg | 0.02 | 0.29 | 1.4 | 5,200 | 1.1 | 0.65 | 3.6 | 1.5 | 8.3 | 79 | 0.94 | 0.16 | 0.12 | 170 | 20 | 0.18 | 0.75 | 0.81 | 0.94 | 10 | 0.13 | 8.4 |
| **Tl** | µg/kg | 1 | 1.5 | 4.1 | 4.0 | 50 | 72 | 2.5 | 81 | 61 | 22 | 1.1 | 4.3 | <LOD | <LOD | <LOD | 2.3 | 5.7 | 8.4 | 2.8 | 7.4 | 2.1 | 17 |
| **Tm** | µg/kg | 1 | <LOD | <LOD | 4.8 | <LOD | 1.8 | 2.5 | 1.1 | 4.0 | <LOD | <LOD | <LOD | <LOD | <LOD | <LOD | <LOD | <LOD | <LOD | <LOD | 2.2 | <LOD | 1.4 |
| **U** | µg/kg | 1 | 4.9 | 6.2 | 270 | 12 | 350 | 110 | 12 | 23 | 7.8 | 2.0 | 21 | <LOD | 9.2 | 10 | 2.2 | 5.9 | 8.3 | 1.4 | 13 | 6.5 | 18 |
| **W** | µg/kg | 1 | 9.1 | 28 | 12 | 6.6 | 24 | 19 | 120 | 44 | 10 | 16 | 14 | 3.7 | 5.9 | 4.6 | 3.0 | 8.4 | 29 | 3.0 | 2.9 | 13 | 43 |
| **V** | µg/kg | 5 | 13 | 86 | 660 | 54 | 680 | 260 | 90 | 270 | 1,400 | 82 | 37 | 8.4 | 42 | 59 | 24 | 140 | 52 | 22 | 1,200 | 6.2 | 810 |
| **Y** | µg/kg | 1 | 10 | 18 | 550 | 12 | 340 | 310 | 100 | 170 | 20 | 3.1 | 1.7 | 8.5 | 4.2 | 15 | 84 | 36 | 53 | 8.8 | 130 | 1.8 | 120 |
| **Yb** | µg/kg | 1 | <LOD | 1.3 | 29 | 1.0 | 11 | 16 | 8.2 | 24 | <LOD | <LOD | <LOD | <LOD | <LOD | 1.6 | 2.9 | 2.5 | 4.6 | <LOD | 15 | <LOD | 8.4 |
| **Zn** | mg/kg | 0.1 | 2.7 | 25 | 10 | 8,400 | 29 | 20 | 26 | 210 | 9,900 | 2.7 | 4.3 | 58 | 1.4 | 3.2 | 5.2 | 27 | 10 | 3.8 | 250 | 83 | 12 |
| **Zr** | µg/kg | 1 | 20 | 210 | 1,500 | 230 | 100 | 930 | 400 | 1,200 | 170 | 180 | 5.2 | 10 | 210 | 300 | 13 | 270 | 380 | 230 | 280 | 5.2 | 120 |
| **REE** | mg/kg | - | 0.045 | 0.12 | 2.1 | 0.062 | 0.88 | 0.90 | 0.42 | 1.5 | 0.10 | 0.022 | 0.014 | 0.10 | 0.038 | 0.086 | 0.48 | 0.21 | 0.33 | 0.048 | 0.87 | 0.014 | 1.2 |

**Table S2.D, part 2 (of 3)**

| **Element** | **Unit** | **LOD** | **TPB22** | **TPB23** | **TPB24** | **TPB25** | **TPB26** | **TPB27** | **TPB28** | **TPB29** | **TPB30** | **TPB31** | **TPB32** | **TPB33** | **TPB34** | **TPB35** | **TPB36** | **TPB37** | **TPB38** | **TPB39** | **TPB40** | **TPB41** | **TPB42** |
| --- | --- | --- | --- | --- | --- | --- | --- | --- | --- | --- | --- | --- | --- | --- | --- | --- | --- | --- | --- | --- | --- | --- | --- |
| **Ag** | µg/kg | 1 | <LOD | 3.1 | 24 | 2.1 | <LOD | <LOD | 1.6 | 9.0 | <LOD | 1.9 | 3.0 | 5.6 | 3.5 | 2.2 | 22 | 7.4 | 2.9 | 1.9 | 6.2 | 4.4 | 4.9 |
| **Al** | mg/kg | 0.2 | 240 | 110 | 2,200 | 39 | 4.3 | 30 | 25 | 6.6 | 0.35 | 3.0 | 10 | 15 | 11 | 5.1 | 220 | 51 | 5.0 | 30 | 860 | 70 | 3.8 |
| **As** | µg/kg | 2 | 39 | 240 | 430 | 9.2 | 18 | 3.5 | 22 | 6.7 | <LOD | 4.5 | 22 | 22 | 19 | 26 | 250 | 81 | 36 | 22 | 30 | 370 | 4.5 |
| **Au** | µg/kg | 1 | <LOD | 2.8 | <LOD | <LOD | <LOD | <LOD | <LOD | <LOD | <LOD | <LOD | <LOD | <LOD | <LOD | <LOD | <LOD | <LOD | <LOD | <LOD | <LOD | <LOD | <LOD |
| **B** | mg/kg | 0.5 | 4.9 | 17 | 41 | 30 | 12 | 2.0 | 11 | 36 | 1.1 | 1.7 | 88 | 3.3 | 2.3 | 2.4 | 11 | 2.8 | 2.0 | 19 | 5.0 | 4.5 | 0.80 |
| **Ba** | mg/kg | 0.2 | 64 | 11 | 21 | 14 | 26 | 0.53 | 0.84 | 26 | 0.68 | 1.0 | 1.1 | 1.5 | 2.1 | 0.92 | 8.5 | 1.1 | 0.83 | 0.69 | 2.8 | 10 | 0.46 |
| **Be** | µg/kg | 1 | 14 | 7.0 | 77 | 3.6 | <LOD | <LOD | <LOD | 1.3 | <LOD | <LOD | 45 | 10 | 18 | 4.1 | 12 | 2.4 | 4.9 | 1.6 | 25 | 11 | <LOD |
| **Bi** | µg/kg | 1 | 1.9 | 8.8 | 11 | <LOD | <LOD | 6.0 | 1.7 | <LOD | <LOD | <LOD | 3.4 | <LOD | 4.5 | <LOD | 3.7 | <LOD | <LOD | 3.4 | 5.2 | 19 | <LOD |
| **Br** | mg/kg | 0.5 | 8.0 | 19 | 41 | 4.7 | 2.7 | 3.1 | 2.0 | 11 | 1.3 | 1.2 | 7.7 | 1.5 | 2.7 | 2.2 | 5.7 | 2.8 | 20 | 5.5 | 7.3 | 4.0 | <LOD |
| **Ca** | g/kg | 0.002 | 1.7 | 3.7 | 16 | 4.3 | 6.9 | 1.0 | 0.73 | 21 | 0.27 | 0.36 | 110 | 50 | 220 | 64 | 2.9 | 62 | 1.0 | 5.0 | 0.26 | 70 | 0.049 |
| **Cd** | µg/kg | 1 | 120 | 530 | 250 | 5.5 | 8.4 | 1.3 | 55 | 38 | <LOD | 23 | 59 | 12 | 75 | 7.4 | 460 | 25 | 18 | 39 | 11 | 73 | <LOD |
| **Ce** | µg/kg | 1 | 450 | 160 | 3,100 | 190 | 6.4 | 5.1 | 53 | 72 | <LOD | 10 | 600 | 130 | 1,000 | 170 | 610 | 170 | 11 | 25 | 62 | 360 | 21 |
| **Co** | µg/kg | 1 | 230 | 54 | 3,800 | 53 | 140 | 5.4 | 64 | 230 | <LOD | 6.7 | 39 | 36 | 50 | 27 | 290 | 150 | 36 | 2,000 | 310 | 280 | 4.5 |
| **Cr** | mg/kg | 0.001 | 0.32 | 0.32 | 7.6 | 0.13 | 0.017 | 0.069 | 0.055 | 0.12 | 0.027 | 0.14 | 0.59 | 0.31 | 0.60 | 0.32 | 2.5 | 0.31 | 0.10 | 2.4 | 0.18 | 0.85 | 0.057 |
| **Cs** | µg/kg | 1 | 34 | 34 | 190 | 39 | 7.8 | 13 | 7.5 | 390 | 1.0 | 5.2 | <LOD | 1.0 | 2.3 | 10 | 130 | 2.6 | 60 | 5.1 | 170 | 85 | <LOD |
| **Cu** | mg/kg | 0.005 | 4.2 | 3.2 | 20 | 7.2 | 16 | 0.52 | 8.2 | 14 | 0.078 | 0.43 | 0.14 | 0.40 | 0.22 | 0.23 | 6.1 | 2.1 | 4.4 | 980 | 2.2 | 570 | 0.29 |
| **Dy** | µg/kg | 1 | 53 | 10 | 340 | 10 | <LOD | 1.2 | 2.7 | 2.4 | <LOD | <LOD | 62 | 25 | 150 | 27 | 41 | 33 | 3.7 | 3.2 | 10 | 33 | 1.6 |
| **Er** | µg/kg | 1 | 30 | 4.4 | 180 | 5.1 | <LOD | <LOD | 1.5 | 1.6 | <LOD | <LOD | 40 | 14 | 88 | 20 | 20 | 21 | 1.9 | 2.5 | 7.5 | 17 | <LOD |
| **Eu** | µg/kg | 1 | 4.4 | <LOD | 95 | 1.9 | 1.3 | <LOD | <LOD | 1.4 | <LOD | <LOD | 24 | 4.9 | 46 | 7.1 | 10 | 6.2 | <LOD | <LOD | 1.4 | 4.8 | <LOD |
| **Fe** | g/kg | 0.0002 | 0.22 | 0.11 | 3.8 | 0.067 | 0.064 | 0.0060 | 0.062 | 0.18 | 0.00074 | 0.0081 | 0.012 | 0.020 | 0.016 | 0.011 | 0.47 | 0.25 | 0.048 | 13 | 0.011 | 0.11 | 0.0023 |
| **Ga** | µg/kg | 5 | 75 | 28 | 710 | 11 | <LOD | <LOD | 11 | <LOD | 22 | <LOD | 21 | 5.6 | 5.4 | <LOD | 67 | 22 | <LOD | 67 | <LOD | 27 | <LOD |
| **Gd** | µg/kg | 1 | 53 | 11 | 380 | 13 | <LOD | 1.1 | 3.8 | 3.8 | <LOD | <LOD | 76 | 26 | 180 | 30 | 51 | 32 | 3.9 | 3.8 | 8.9 | 42 | 1.5 |
| **Ge** | µg/kg | 10 | <LOD | <LOD | 14 | <LOD | <LOD | <LOD | <LOD | <LOD | <LOD | <LOD | 18 | <LOD | 43 | <LOD | 16 | <LOD | <LOD | 620 | <LOD | 170 | <LOD |
| **Hf** | µg/kg | 1 | 3.5 | 4.0 | 89 | 1.4 | <LOD | 3.2 | 1.8 | <LOD | <LOD | 2.8 | 1.3 | 3.5 | <LOD | 1.1 | 13 | 14 | 2.1 | 2.1 | 11 | 8.3 | 10 |
| **Hg** | µg/kg | 1 | <LOD | <LOD | 16 | 1.5 | <LOD | 1.1 | <LOD | 8.5 | <LOD | <LOD | 1.4 | 3.4 | <LOD | 1.4 | 2.9 | 1.4 | <LOD | <LOD | 2.6 | 3.4 | <LOD |
| **Ho** | µg/kg | 1 | 11 | 1.9 | 67 | 1.7 | <LOD | <LOD | <LOD | <LOD | <LOD | <LOD | 14 | 5.7 | 33 | 6.2 | 7.3 | 6.9 | <LOD | <LOD | 2.4 | 6.5 | <LOD |
| **I** | mg/kg | 0.25 | 0.30 | 20 | 3.2 | 4.3 | 4.0 | 3.5 | 3.1 | 3.7 | 3.5 | 7.9 | 58 | 7.5 | 8.5 | 10 | 97 | 6.4 | 2.9 | 330 | 4.5 | 160 | 12 |
| **Ir** | µg/kg | 1 | <LOD | <LOD | <LOD | <LOD | <LOD | <LOD | <LOD | <LOD | <LOD | <LOD | <LOD | <LOD | <LOD | <LOD | <LOD | <LOD | <LOD | <LOD | <LOD | <LOD | <LOD |
| **K** | g/kg | 0.005 | 34 | 17 | 16 | 31 | 8.7 | 5.1 | 15 | 22 | 3.3 | 3.4 | 0.067 | 0.036 | 0.40 | 0.42 | 12 | 12 | 13 | 1.3 | 12 | 8.5 | 0.084 |
| **La** | µg/kg | 1 | 280 | 120 | 1,400 | 100 | 3.6 | 6.3 | 26 | 57 | <LOD | 5.5 | 680 | 130 | 1,400 | 170 | 400 | 130 | 5.3 | 17 | 31 | 310 | 11 |
| **Li** | µg/kg | 30 | 150 | 140 | 1,000 | 35 | 41 | <LOD | 1,900 | 55 | <LOD | <LOD | 110 | 140 | 170 | 70 | 180 | 80 | 100 | 220 | 560 | 120 | <LOD |
| **Lu** | µg/kg | 1 | 3.1 | <LOD | 20 | <LOD | <LOD | <LOD | <LOD | <LOD | <LOD | <LOD | 3.6 | 2.1 | 8.0 | 2.2 | 2.5 | 2.6 | <LOD | <LOD | 1.3 | 2.2 | <LOD |
| **Mg** | g/kg | 0.0002 | 2.1 | 1.0 | 9.0 | 1.9 | 3.6 | 0.22 | 1.0 | 4.6 | 0.45 | 0.15 | 1.5 | 3.2 | 2.0 | 1.2 | 1.1 | 1.3 | 1.6 | 230 | 1.8 | 2.0 | 0.18 |
| **Mn** | mg/kg | 0.02 | 260 | 32 | 280 | 7.6 | 36 | 32 | 9.5 | 170 | 0.45 | 3.9 | 2.2 | 10 | 4.7 | 4.3 | 25 | 15 | 10 | 53 | 400 | 29 | 0.22 |
| **Mo** | mg/kg | 0.005 | 0.14 | 0.42 | 0.19 | 0.046 | 0.58 | 0.036 | 0.12 | 1.1 | 0.0057 | 0.076 | 0.033 | 0.19 | 0.042 | 0.046 | 0.069 | 0.33 | 0.057 | 0.44 | 0.035 | 0.24 | 0.0074 |
| **Na** | g/kg | 0.002 | 0.034 | 0.063 | 0.18 | 0.10 | 0.0076 | 2.3 | 6.5 | 0.057 | 91 | 0.24 | 0.10 | 0.50 | 0.15 | 1.9 | 0.11 | 0.21 | 3.2 | 0.71 | 0.45 | 6.7 | 0.032 |
| **Nb** | µg/kg | 1 | 30 | 82 | 230 | 8.9 | <LOD | 1.1 | 1.7 | 2.3 | <LOD | 2.3 | 2.5 | 4.2 | 2.9 | 1.8 | 36 | 210 | 22 | 30 | 3.7 | 30 | 5.1 |
| **Nd** | µg/kg | 1 | 210 | 69 | 1,500 | 86 | 4.2 | 4.4 | 23 | 29 | <LOD | 4.2 | 380 | 100 | 830 | 120 | 290 | 130 | 9.3 | 56 | 32 | 200 | 10 |
| **Ni** | µg/kg | 50 | 730 | 1,200 | 6,400 | 600 | 1,500 | 200 | 480 | 830 | <LOD | 70 | 2,000 | 210 | 480 | 370 | 2,100 | 1,100 | 460 | 1,400 | 7,000 | 450 | <LOD |
| **Os** | µg/kg | 5 | <LOD | <LOD | <LOD | <LOD | <LOD | <LOD | <LOD | <LOD | <LOD | <LOD | <LOD | <LOD | <LOD | <LOD | <LOD | <LOD | <LOD | <LOD | <LOD | <LOD | <LOD |
| **P** | g/kg | 0.001 | 3.1 | 4.2 | 4.0 | 0.53 | 9.3 | 3.1 | 2.5 | 7.7 | 0.032 | 0.31 | 88 | 29 | 180 | 49 | 1.0 | 52 | 0.81 | 3.7 | 1.1 | 49 | 0.026 |
| **Pb** | µg/kg | 1 | 270 | 530 | 1,700 | 58 | 5.7 | 11 | 42 | 37 | 2.1 | 30 | 100 | 35 | 230 | 21 | 860 | 50 | 29 | 92 | 100 | 280 | 350 |
| **Pd** | µg/kg | 5 | <LOD | <LOD | 14 | <LOD | <LOD | <LOD | <LOD | <LOD | <LOD | <LOD | <LOD | <LOD | <LOD | <LOD | 10 | 8.0 | <LOD | <LOD | 5.1 | <LOD | <LOD |
| **Pr** | µg/kg | 1 | 53 | 18 | 360 | 22 | <LOD | 1.2 | 6.3 | 7.5 | <LOD | 1.1 | 98 | 23 | 210 | 30 | 68 | 30 | 1.5 | 3.6 | 7.7 | 51 | 2.5 |
| **Pt** | µg/kg | 1 | <LOD | <LOD | <LOD | <LOD | <LOD | <LOD | <LOD | <LOD | <LOD | <LOD | <LOD | <LOD | <LOD | <LOD | <LOD | <LOD | <LOD | <LOD | <LOD | <LOD | <LOD |
| **Rb** | mg/kg | 0.01 | 42 | 19 | 27 | 20 | 2.8 | 2.9 | 2.4 | 91 | 0.30 | 1.3 | 0.042 | 0.024 | 0.56 | 0.24 | 17 | 2.9 | 8.6 | 1.3 | 41 | 13 | 0.076 |
| **Re** | µg/kg | 1 | <LOD | <LOD | <LOD | <LOD | <LOD | <LOD | <LOD | 2.0 | <LOD | <LOD | <LOD | <LOD | <LOD | <LOD | <LOD | <LOD | <LOD | <LOD | <LOD | <LOD | <LOD |
| **Rh** | µg/kg | 5 | <LOD | <LOD | <LOD | <LOD | <LOD | <LOD | <LOD | <LOD | <LOD | <LOD | <LOD | <LOD | 11 | <LOD | <LOD | <LOD | <LOD | 15 | <LOD | 9.3 | <LOD |
| **Ru** | µg/kg | 5 | <LOD | <LOD | <LOD | <LOD | <LOD | <LOD | <LOD | <LOD | <LOD | <LOD | <LOD | <LOD | <LOD | <LOD | <LOD | <LOD | <LOD | <LOD | <LOD | <LOD | <LOD |
| **S** | g/kg | 0.005 | 1.4 | 4.6 | 2.7 | 0.70 | 3.4 | 0.54 | 1.1 | 5.8 | 2.4 | 0.30 | 2.1 | 0.037 | 0.064 | 0.073 | 0.65 | 0.62 | 0.61 | 1.0 | 1.1 | 2.8 | 0.027 |
| **Sb** | µg/kg | 1 | 2.6 | 23 | 17 | 1.4 | <LOD | 18 | 1.8 | 1.5 | <LOD | <LOD | 590 | 110 | 1,300 | 350 | 24 | 14 | 4.1 | 5.3 | 8.4 | 160 | 4.7 |
| **Sc** | µg/kg | 1 | 72 | 24 | 1,000 | 20 | 2.0 | 2.5 | 14 | 2.7 | <LOD | 1.4 | 13 | 16 | 28 | 13 | 82 | 23 | 3.0 | 20 | 14 | 22 | 1.7 |
| **Se** | mg/kg | 0.05 | 0.081 | <LOD | 0.40 | 0.054 | 0.81 | <LOD | <LOD | <LOD | <LOD | <LOD | <LOD | <LOD | <LOD | <LOD | 0.086 | <LOD | <LOD | 55 | <LOD | 24 | <LOD |
| **Si** | mg/kg | 5 | 540 | 180 | 830 | 57 | 14 | 260 | 42 | 140 | <LOD | 880 | 910 | 33 | 59 | 360 | 230 | 1,200 | 160 | 110 | 560 | 510 | 260 |
| **Sm** | µg/kg | 1 | 45 | 13 | 360 | 17 | 1.1 | 1.0 | 4.7 | 3.8 | <LOD | <LOD | 69 | 20 | 140 | 23 | 56 | 27 | 2.4 | 3.8 | 8.6 | 36 | 2.2 |
| **Sn** | µg/kg | 1 | 26 | 45 | 81 | 1.6 | <LOD | <LOD | <LOD | <LOD | <LOD | <LOD | 11 | 5.9 | 3.3 | 6.5 | 200 | 27 | 11 | 14 | 17 | 23 | 8.3 |
| **Sr** | mg/kg | 0.02 | 11 | 8.6 | 56 | 34 | 32 | 1.3 | 6.8 | 78 | 1.0 | 3.6 | 52 | 17 | 100 | 24 | 7.9 | 10 | 1.3 | 4.6 | 1.1 | 31 | 0.22 |
| **Ta** | µg/kg | 50 | <LOD | <LOD | <LOD | <LOD | <LOD | <LOD | <LOD | <LOD | <LOD | <LOD | <LOD | <LOD | <LOD | <LOD | <LOD | <LOD | <LOD | <LOD | <LOD | <LOD | <LOD |
| **Tb** | µg/kg | 1 | 8.7 | 1.9 | 56 | 1.7 | <LOD | <LOD | <LOD | <LOD | <LOD | <LOD | 10 | 3.9 | 22 | 4.5 | 8.0 | 5.3 | <LOD | <LOD | 1.8 | 5.6 | <LOD |
| **Te** | µg/kg | 5 | <LOD | <LOD | <LOD | <LOD | <LOD | <LOD | <LOD | <LOD | <LOD | <LOD | <LOD | <LOD | <LOD | <LOD | <LOD | <LOD | <LOD | <LOD | <LOD | <LOD | <LOD |
| **Th** | µg/kg | 1 | 57 | 17 | 270 | 37 | 1.9 | 1.2 | 9.4 | 2.5 | <LOD | 1.3 | 10 | 7.8 | 12 | 4.7 | 120 | 26 | 5.9 | 5.6 | 16 | 23 | 3.6 |
| **Ti** | mg/kg | 0.02 | 8.9 | 11 | 110 | 2.5 | 0.14 | 0.25 | 1.0 | 0.41 | 0.36 | 0.48 | 0.55 | 0.84 | 0.47 | 0.38 | 12 | 1,700 | 800 | 27 | 0.95 | 2.7 | 0.81 |
| **Tl** | µg/kg | 1 | 24 | 84 | 24 | 2.0 | <LOD | 1.8 | 1.2 | 190 | <LOD | 3.5 | 7.7 | <LOD | <LOD | <LOD | 5.7 | 29 | 5.2 | 7.7 | 42 | 12 | <LOD |
| **Tm** | µg/kg | 1 | 3.9 | <LOD | 23 | <LOD | <LOD | <LOD | <LOD | <LOD | <LOD | <LOD | 4.7 | 2.2 | 9.4 | 2.4 | 2.8 | 2.3 | <LOD | <LOD | 1.2 | 2.3 | <LOD |
| **U** | µg/kg | 1 | 9.3 | 6.6 | 63 | 3.1 | <LOD | <LOD | 6.4 | <LOD | <LOD | 2.8 | 18 | 67 | 36 | 26 | 18 | 170 | 37 | 19 | 25 | 88 | 2.1 |
| **W** | µg/kg | 1 | 6.3 | 2.4 | 31 | 4.1 | 1.6 | 1.8 | 2.7 | 22 | <LOD | 1.4 | 7.1 | 7.4 | 10 | 11 | 12 | 6.6 | 14 | 10 | 10 | 25 | 1.4 |
| **V** | µg/kg | 5 | 390 | 180 | 15,000 | 100 | 6.7 | 5.1 | 40 | 27 | <LOD | 15 | 140 | 230 | 410 | 260 | 880 | 540 | 80 | 820 | 35 | 680 | 32 |
| **Y** | µg/kg | 1 | 350 | 69 | 1,700 | 43 | 2.6 | 9.4 | 13 | 22 | <LOD | 3.0 | 930 | 190 | 2,000 | 250 | 140 | 210 | 16 | 31 | 58 | 200 | 9.0 |
| **Yb** | µg/kg | 1 | 24 | 3.7 | 140 | 5.0 | <LOD | <LOD | 1.2 | <LOD | <LOD | <LOD | 24 | 13 | 54 | 14 | 16 | 16 | 1.2 | 2.1 | 7.1 | 15 | <LOD |
| **Zn** | mg/kg | 0.1 | 19 | 46 | 59 | 6.9 | 60 | 5.2 | 9.2 | 110 | 3,400 | 9.5 | 3,600 | 1.9 | 2.2 | 1.2 | 12 | 16 | 28 | 9,500 | 12 | 1,100 | 1.7 |
| **Zr** | µg/kg | 1 | 120 | 170 | 3,100 | 37 | 6.2 | 110 | 26 | 5.6 | 2.2 | 120 | 84 | 320 | 63 | 87 | 320 | 390 | 85 | 150 | 430 | 320 | 460 |
| **REE** | mg/kg | - | 1.7 | 0.51 | 11 | 0.52 | 0.026 | 0.036 | 0.15 | 0.21 | 0.0080 | 0.030 | 3.0 | 0.71 | 6.2 | 0.89 | 1.8 | 0.86 | 0.061 | 0.17 | 0.25 | 1.3 | 0.064 |

**Table S2.D, part 3 (of 3)**

| **Element** | **Unit** | **LOD** | **TPB43** | **TPB44** | **TPB45** | **TPB46** | **TPB47** | **TPB48** | **TPB49** | **TPB50** | **TPB51** | **TPB52** | **TPB53** | **TPB54** | **TPB55** | **TPB56** | **TPB57** | **TPB58** | **TPB59** | **TPB60** | **TPB61** | **TPB62** |
| --- | --- | --- | --- | --- | --- | --- | --- | --- | --- | --- | --- | --- | --- | --- | --- | --- | --- | --- | --- | --- | --- | --- |
| **Ag** | µg/kg | 1 | 3.7 | 4.1 | 3.5 | 2.8 | 3.7 | 5.4 | 8.8 | 4.8 | <LOD | <LOD | <LOD | <LOD | <LOD | 3.9 | 1.7 | <LOD | 18 | 19 | <LOD | <LOD |
| **Al** | mg/kg | 0.2 | 5.2 | 15 | 500 | 60 | 1.7 | 13 | 58 | 1.8 | 2.3 | 1.2 | 1.6 | 0.30 | 0.60 | 67 | 1,900 | 23 | 14 | 82 | <LOD | 0.52 |
| **As** | µg/kg | 2 | 12 | 170 | 55 | 68 | 26 | 29 | 32 | 5.7 | 10 | 10 | 13 | 3.5 | 8.1 | 89 | 16 | 12 | 12 | 29 | <LOD | 8.7 |
| **Au** | µg/kg | 1 | <LOD | 1.2 | <LOD | <LOD | <LOD | <LOD | <LOD | <LOD | <LOD | <LOD | <LOD | <LOD | <LOD | <LOD | <LOD | <LOD | <LOD | 1.1 | <LOD | <LOD |
| **B** | mg/kg | 0.5 | 5.1 | 27 | 8.1 | 4.4 | 12 | 4.5 | 7.6 | 1.5 | <LOD | 1.2 | 4.8 | <LOD | 1.0 | 4.4 | 22 | 20 | 15 | 6.2 | 1.3 | 1.3 |
| **Ba** | mg/kg | 0.2 | 0.54 | 2.0 | 1.9 | 2.6 | 2.0 | 6.0 | 5.3 | 0.65 | 0.23 | 0.28 | 0.94 | <LOD | <LOD | 14 | 19 | 0.90 | 1.2 | 1.3 | <LOD | 0.39 |
| **Be** | µg/kg | 1 | <LOD | 2.0 | 11 | 4.4 | 1.7 | 1.1 | 3.0 | <LOD | <LOD | <LOD | <LOD | <LOD | <LOD | 4.0 | 14 | <LOD | 5.1 | 5.0 | <LOD | <LOD |
| **Bi** | µg/kg | 1 | <LOD | 2.3 | 1.2 | 4.0 | <LOD | <LOD | <LOD | <LOD | <LOD | <LOD | <LOD | <LOD | <LOD | 1.9 | 4.6 | <LOD | <LOD | <LOD | 1.9 | <LOD |
| **Br** | mg/kg | 0.5 | 72 | 270 | 4.7 | 2.1 | 1.0 | 1.9 | 13 | 2.1 | <LOD | 0.67 | <LOD | <LOD | <LOD | 5.1 | 1.9 | 1.0 | 3.3 | 8.6 | <LOD | <LOD |
| **Ca** | g/kg | 0.002 | 0.34 | 0.88 | 0.37 | 2.0 | 0.46 | 0.47 | 8.5 | 0.077 | 0.043 | 0.066 | 0.027 | 0.0075 | 0.086 | 3.1 | 5.4 | 2.0 | 18 | 0.39 | 0.060 | 0.066 |
| **Cd** | µg/kg | 1 | 2.8 | 14 | 7.4 | 7.2 | 1.1 | 70 | 14 | 1.1 | 1.7 | 5.5 | 15 | <LOD | 2.3 | 43 | 14 | 120 | 59 | 3.9 | <LOD | <LOD |
| **Ce** | µg/kg | 1 | 8.4 | 81 | 45 | 100 | 3.8 | 74 | 79 | 2.5 | 2.9 | 1.1 | 3.6 | <LOD | 5.0 | 140 | 190 | 27 | 52 | 56 | <LOD | 4.4 |
| **Co** | µg/kg | 1 | 48 | 1,000 | 360 | 48 | 29 | 27 | 88 | 9.1 | 2.4 | 2.3 | 2.7 | <LOD | 1.5 | 52 | 85 | 250 | 280 | 67 | 1.5 | 2.5 |
| **Cr** | mg/kg | 0.001 | 0.21 | 1.0 | 0.29 | 0.34 | 0.13 | 0.63 | 0.30 | 0.048 | 0.015 | 0.057 | 0.013 | 0.0032 | 0.031 | 0.43 | 0.088 | 0.11 | 0.43 | 0.41 | 0.0030 | 0.0064 |
| **Cs** | µg/kg | 1 | 13 | 36 | 340 | 26 | 44 | 4.4 | 17 | 5.6 | 1.2 | 1.6 | 2.7 | <LOD | 1.4 | 14 | 400 | 4.0 | 16 | 21 | 1.0 | 7.0 |
| **Cu** | mg/kg | 0.005 | 2.9 | 25 | 2.1 | 1.5 | 0.41 | 0.81 | 4.7 | 0.13 | 0.018 | 0.031 | 0.015 | <LOD | 0.036 | 6.0 | 32 | 15 | 1.4 | 0.77 | 0.19 | 0.25 |
| **Dy** | µg/kg | 1 | <LOD | 17 | 10 | 5.5 | <LOD | 1.6 | 7.4 | <LOD | <LOD | <LOD | <LOD | <LOD | <LOD | 10 | 72 | 2.1 | 10 | 4.7 | <LOD | <LOD |
| **Er** | µg/kg | 1 | <LOD | 12 | 9.0 | 3.1 | <LOD | 1.2 | 3.5 | <LOD | <LOD | <LOD | <LOD | <LOD | <LOD | 5.0 | 48 | 1.3 | 7.1 | 2.6 | <LOD | <LOD |
| **Eu** | µg/kg | 1 | <LOD | 4.5 | 1.2 | 1.5 | <LOD | <LOD | 2.5 | <LOD | <LOD | <LOD | <LOD | <LOD | <LOD | 1.6 | 13 | <LOD | 1.9 | 1.2 | <LOD | <LOD |
| **Fe** | g/kg | 0.0002 | 0.026 | 0.037 | 0.014 | 0.074 | 0.0054 | 0.019 | 0.072 | 0.0014 | 0.0015 | 0.0015 | 0.00050 | <LOD | 0.00087 | 0.13 | 0.066 | 0.075 | 0.91 | 0.012 | <LOD | 0.0011 |
| **Ga** | µg/kg | 5 | <LOD | 6.5 | <LOD | 19 | <LOD | <LOD | 23 | <LOD | <LOD | <LOD | <LOD | <LOD | <LOD | 18 | <LOD | 6.5 | 64 | 5.5 | <LOD | <LOD |
| **Gd** | µg/kg | 1 | 1.4 | 21 | 8.5 | 10 | <LOD | 2.1 | 10 | <LOD | <LOD | <LOD | <LOD | <LOD | <LOD | 12 | 83 | 2.5 | 9.1 | 2.8 | <LOD | <LOD |
| **Ge** | µg/kg | 10 | <LOD | 19 | <LOD | <LOD | <LOD | <LOD | <LOD | <LOD | <LOD | <LOD | <LOD | <LOD | <LOD | 23 | <LOD | <LOD | 1,100 | 28 | <LOD | <LOD |
| **Hf** | µg/kg | 1 | <LOD | <LOD | <LOD | 1.2 | <LOD | 5.3 | 2.5 | 6.1 | <LOD | <LOD | <LOD | <LOD | <LOD | 1.9 | <LOD | <LOD | 16 | 22 | <LOD | <LOD |
| **Hg** | µg/kg | 1 | 1.4 | 3.0 | <LOD | <LOD | <LOD | 1.8 | <LOD | <LOD | <LOD | <LOD | <LOD | <LOD | <LOD | 2.3 | 7.3 | <LOD | 11 | 3.2 | <LOD | <LOD |
| **Ho** | µg/kg | 1 | <LOD | 3.6 | 2.7 | 1.3 | <LOD | <LOD | 1.8 | <LOD | <LOD | <LOD | <LOD | <LOD | <LOD | 1.8 | 16 | <LOD | 2.2 | 1.1 | <LOD | <LOD |
| **I** | mg/kg | 0.25 | 4.0 | 6.0 | 1.5 | 5.2 | 1.8 | 2.3 | 6.8 | 2.8 | 0.70 | 0.92 | 0.71 | 0.13 | 0.82 | 0.40 | 0.44 | 0.28 | 43 | 96 | 0.13 | 0.13 |
| **Ir** | µg/kg | 1 | <LOD | <LOD | <LOD | <LOD | <LOD | <LOD | <LOD | <LOD | <LOD | <LOD | <LOD | <LOD | <LOD | <LOD | <LOD | <LOD | <LOD | <LOD | <LOD | <LOD |
| **K** | g/kg | 0.005 | 11 | 61 | 12 | 3.2 | 2.2 | 5.8 | 10 | 0.24 | 1.0 | 0.30 | 1.7 | 0.0057 | 0.27 | 35 | 14 | 9.2 | 1.3 | 2.1 | 0.90 | 1.6 |
| **La** | µg/kg | 1 | 4.2 | 22 | 43 | 53 | 2.3 | 12 | 38 | 1.6 | 1.3 | <LOD | 2.0 | <LOD | 2.2 | 67 | 490 | 11 | 58 | 25 | <LOD | 2.0 |
| **Li** | µg/kg | 30 | 220 | 600 | 180 | 170 | 41 | 44 | 160 | 51 | <LOD | <LOD | 34 | <LOD | <LOD | 1,000 | 280 | 60 | 130 | 100 | <LOD | <LOD |
| **Lu** | µg/kg | 1 | <LOD | 1.4 | 2.0 | <LOD | <LOD | <LOD | <LOD | <LOD | <LOD | <LOD | <LOD | <LOD | <LOD | <LOD | 7.2 | <LOD | 1.0 | <LOD | <LOD | <LOD |
| **Mg** | g/kg | 0.0002 | 0.46 | 6.2 | 2.3 | 0.36 | 0.51 | 0.33 | 1.1 | 0.035 | 0.017 | 0.030 | 0.042 | 0.0024 | 0.031 | 1.3 | 2.5 | 4.0 | 1.5 | 1.2 | 0.15 | 0.14 |
| **Mn** | mg/kg | 0.02 | 1.1 | 29 | 430 | 5.4 | 5.3 | 8.4 | 6.4 | 0.21 | 0.11 | 0.069 | 0.16 | 0.029 | 0.32 | 37 | 1,100 | 29 | 17 | 55 | 0.075 | 1.1 |
| **Mo** | mg/kg | 0.005 | 0.029 | 0.22 | 0.020 | 0.19 | 0.032 | 0.11 | 0.13 | 0.012 | <LOD | 0.0070 | 0.0054 | <LOD | 0.0052 | 1.8 | 0.025 | 0.95 | 0.26 | 0.024 | <LOD | 0.0084 |
| **Na** | g/kg | 0.002 | 0.67 | 0.95 | 0.75 | 0.23 | 0.57 | 0.47 | 2.6 | 0.53 | 0.27 | 3.8 | 3.4 | 0.0061 | 4.0 | 0.11 | 0.039 | 0.79 | 0.58 | 0.33 | 0.0033 | 0.019 |
| **Nb** | µg/kg | 1 | 1.2 | 3.6 | <LOD | 2.3 | <LOD | 74 | 5.5 | 150 | <LOD | 5.1 | <LOD | <LOD | 5.1 | 6.4 | <LOD | 1.4 | 19 | 10 | <LOD | <LOD |
| **Nd** | µg/kg | 1 | 4.4 | 52 | 27 | 44 | 2.8 | 12 | 44 | 1.7 | 1.5 | <LOD | 2.0 | <LOD | 2.4 | 72 | 350 | 12 | 50 | 31 | <LOD | 2.2 |
| **Ni** | µg/kg | 50 | 380 | 2,900 | 2,300 | 270 | 200 | 270 | 430 | <LOD | <LOD | <LOD | <LOD | <LOD | <LOD | 630 | 2,700 | 1,100 | 600 | 1,100 | <LOD | <LOD |
| **Os** | µg/kg | 5 | <LOD | <LOD | <LOD | <LOD | <LOD | <LOD | <LOD | <LOD | <LOD | <LOD | <LOD | <LOD | <LOD | <LOD | <LOD | <LOD | <LOD | <LOD | <LOD | <LOD |
| **P** | g/kg | 0.001 | 0.19 | 2.7 | 2.1 | 0.33 | 0.35 | 0.69 | 2.8 | 0.048 | 0.011 | 0.032 | 0.80 | 0.011 | 0.091 | 4.8 | 3.2 | 6.1 | 12 | 0.26 | 0.017 | 0.16 |
| **Pb** | µg/kg | 1 | 31 | 210 | 66 | 140 | 9.1 | 100 | 72 | 10 | 6.2 | 4.9 | 3.1 | <LOD | 4.9 | 380 | 88 | 18 | 230 | 66 | <LOD | 3.3 |
| **Pd** | µg/kg | 5 | <LOD | <LOD | <LOD | <LOD | <LOD | <LOD | <LOD | <LOD | <LOD | <LOD | <LOD | <LOD | <LOD | <LOD | <LOD | <LOD | <LOD | 390 | <LOD | <LOD |
| **Pr** | µg/kg | 1 | 1.5 | 10 | 6.8 | 12 | <LOD | 3.2 | 11 | <LOD | <LOD | <LOD | <LOD | <LOD | <LOD | 17 | 88 | 2.9 | 11 | 7.5 | <LOD | <LOD |
| **Pt** | µg/kg | 1 | <LOD | <LOD | <LOD | <LOD | <LOD | <LOD | <LOD | <LOD | <LOD | <LOD | <LOD | <LOD | <LOD | <LOD | <LOD | <LOD | 1.4 | 3.2 | <LOD | <LOD |
| **Rb** | mg/kg | 0.01 | 10 | 51 | 97 | 1.2 | 5.5 | 2.8 | 13 | 0.13 | 0.12 | 0.32 | 0.86 | <LOD | 0.32 | 7.1 | 31 | 5.3 | 2.9 | 5.7 | 0.47 | 1.1 |
| **Re** | µg/kg | 1 | 1.8 | 4.1 | <LOD | <LOD | <LOD | <LOD | <LOD | <LOD | <LOD | <LOD | <LOD | <LOD | <LOD | <LOD | <LOD | <LOD | <LOD | <LOD | <LOD | <LOD |
| **Rh** | µg/kg | 5 | <LOD | <LOD | <LOD | <LOD | <LOD | <LOD | <LOD | <LOD | <LOD | <LOD | <LOD | <LOD | <LOD | <LOD | <LOD | <LOD | <LOD | 5.8 | <LOD | <LOD |
| **Ru** | µg/kg | 5 | <LOD | <LOD | <LOD | <LOD | <LOD | <LOD | <LOD | <LOD | <LOD | <LOD | <LOD | <LOD | <LOD | <LOD | <LOD | <LOD | <LOD | <LOD | <LOD | <LOD |
| **S** | g/kg | 0.005 | 1.4 | 9.0 | 1.0 | 0.84 | 0.11 | 0.69 | 1.6 | 0.52 | 0.81 | 5.5 | 4.7 | 0.028 | 5.0 | 2.1 | 3.1 | 3.6 | 0.40 | 0.28 | 0.018 | 0.054 |
| **Sb** | µg/kg | 1 | 4.3 | 19 | 18 | 12 | 4.6 | 5.4 | 3.4 | 2.7 | 1.5 | 1.5 | 5.6 | <LOD | 1.8 | 9.0 | 3.6 | 1.5 | 21 | 5.8 | <LOD | 1.8 |
| **Sc** | µg/kg | 1 | 1.7 | 2.2 | 6.3 | 10 | 1.8 | 2.7 | 19 | 1.3 | <LOD | <LOD | <LOD | <LOD | <LOD | 20 | 33 | 4.6 | 10 | 5.8 | <LOD | <LOD |
| **Se** | mg/kg | 0.05 | 0.071 | 0.13 | <LOD | <LOD | <LOD | 0.052 | <LOD | <LOD | <LOD | <LOD | <LOD | <LOD | <LOD | <LOD | <LOD | 0.055 | 92 | 0.10 | <LOD | <LOD |
| **Si** | mg/kg | 5 | 670 | 210 | 78 | 62 | 74 | 52 | 97 | 43 | 11 | 13 | 15 | <LOD | 7.8 | 490 | 180 | 45 | 250 | 930 | 8.1 | 6.8 |
| **Sm** | µg/kg | 1 | <LOD | 15 | 7.4 | 8.3 | <LOD | 1.9 | 9.0 | <LOD | <LOD | <LOD | <LOD | <LOD | <LOD | 15 | 72 | 2.6 | 11 | 3.6 | <LOD | <LOD |
| **Sn** | µg/kg | 1 | 11 | 180 | 24 | 9.0 | 2.4 | 2.6 | 5.3 | 6.5 | 75 | 8.2 | 52 | <LOD | 5.3 | 100 | 13 | <LOD | 30 | 20 | 2.0 | 1.2 |
| **Sr** | mg/kg | 0.02 | 1.0 | 2.5 | 2.7 | 9.3 | 1.6 | 4.1 | 43 | 0.39 | 0.27 | 1.2 | 0.28 | 0.028 | 1.0 | 10 | 13 | 10 | 11 | 1.7 | 0.026 | 0.36 |
| **Ta** | µg/kg | 50 | <LOD | <LOD | <LOD | <LOD | <LOD | <LOD | <LOD | <LOD | <LOD | <LOD | <LOD | <LOD | <LOD | <LOD | <LOD | <LOD | <LOD | <LOD | <LOD | <LOD |
| **Tb** | µg/kg | 1 | <LOD | 3.7 | 1.3 | 1.2 | <LOD | <LOD | 1.6 | <LOD | <LOD | <LOD | <LOD | <LOD | <LOD | 1.9 | 11 | <LOD | 1.9 | <LOD | <LOD | <LOD |
| **Te** | µg/kg | 5 | <LOD | <LOD | <LOD | <LOD | <LOD | <LOD | <LOD | <LOD | <LOD | <LOD | <LOD | <LOD | <LOD | <LOD | <LOD | <LOD | <LOD | <LOD | <LOD | <LOD |
| **Th** | µg/kg | 1 | 1.8 | 8.4 | 5.7 | 19 | 1.1 | 1.8 | 21 | 2.5 | <LOD | <LOD | <LOD | <LOD | <LOD | 25 | 2.6 | 4.3 | 12 | 7.3 | <LOD | <LOD |
| **Ti** | mg/kg | 0.02 | 0.33 | 0.38 | 0.22 | 1.0 | 0.28 | 620 | 2.5 | 1,400 | 0.043 | 0.053 | 0.088 | 0.062 | 0.022 | 1.3 | 0.37 | 0.35 | 6.0 | 2.0 | <LOD | <LOD |
| **Tl** | µg/kg | 1 | 8.8 | 61 | 160 | 2.6 | <LOD | 7.8 | 7.3 | <LOD | <LOD | 1.4 | <LOD | <LOD | <LOD | 2.4 | 30 | 13 | 17 | 6.4 | <LOD | <LOD |
| **Tm** | µg/kg | 1 | <LOD | 1.3 | 1.7 | <LOD | <LOD | <LOD | <LOD | <LOD | <LOD | <LOD | <LOD | <LOD | <LOD | <LOD | 7.1 | <LOD | <LOD | <LOD | <LOD | <LOD |
| **U** | µg/kg | 1 | 3.0 | 13 | 6.9 | 3.8 | 1.9 | 2.3 | 52 | 1.8 | 3.6 | 1.0 | 1.5 | <LOD | <LOD | 5.0 | 3.2 | <LOD | 61 | 5.2 | <LOD | <LOD |
| **W** | µg/kg | 1 | 3.1 | 49 | 17 | 5.3 | 8.5 | 2.8 | 5.0 | 4.9 | 1.8 | <LOD | 2.3 | <LOD | <LOD | 92 | 5.9 | 3.1 | 28 | 7.3 | <LOD | <LOD |
| **V** | µg/kg | 5 | 61 | 480 | 28 | 140 | 41 | 41 | 280 | 53 | <LOD | <LOD | <LOD | <LOD | <LOD | 170 | 73 | 53 | 180 | 49 | <LOD | 6.9 |
| **Y** | µg/kg | 1 | 4.4 | 77 | 96 | 22 | 2.8 | 8.7 | 43 | 1.5 | <LOD | <LOD | <LOD | <LOD | 1.9 | 55 | 650 | 11 | 120 | 23 | <LOD | 2.4 |
| **Yb** | µg/kg | 1 | <LOD | 8.2 | 13 | 2.3 | <LOD | <LOD | 3.5 | <LOD | <LOD | <LOD | <LOD | <LOD | <LOD | 3.5 | 40 | <LOD | 5.4 | 2.5 | <LOD | <LOD |
| **Zn** | mg/kg | 0.1 | 4.3 | 45 | 24 | 3.6 | 1.0 | 27 | 28 | 2.4 | 0.21 | 0.53 | 0.83 | 0.27 | 1.3 | 25 | 25 | 60 | 22,000 | 18 | 0.21 | 0.31 |
| **Zr** | µg/kg | 1 | 17 | 32 | 17 | 29 | 8.3 | 130 | 94 | 170 | 6.2 | 2.1 | 3.1 | <LOD | 1.6 | 70 | 13 | 14 | 790 | 900 | <LOD | <LOD |
| **REE** | mg/kg | - | 0.030 | 0.33 | 0.28 | 0.28 | 0.019 | 0.12 | 0.27 | 0.014 | 0.012 | 0.0086 | 0.014 | 0.0080 | 0.017 | 0.43 | 2.2 | 0.081 | 0.35 | 0.17 | 0.0080 | 0.017 |

**Table S3.A, part 1 (of 2)** Average Daily Doses (ADDs; µg/kg/day) of all elements with available TDIs, associated with each investigated ***synthetic*** food supplement (S01-S29). Values in parentheses = ADDs in % of TDIs.

| **Product** | **Group** | **Ag** | | **Al** | | **As** | | **B** | | **Ba** | | **Be** | | **Br** | | **Cd** | | **Co** | | **Cr** | | **Cu** | | **Fe** | |
| --- | --- | --- | --- | --- | --- | --- | --- | --- | --- | --- | --- | --- | --- | --- | --- | --- | --- | --- | --- | --- | --- | --- | --- | --- | --- |
| S01 | Women, normal weight | 1.5E-06 | (0) | 1.9E-03 | (0) | 3.1E-06 | (0) | 2.2E-03 | (0) | 3.1E-04 | (0) | 1.5E-06 | (0) | 4.8E-03 | (0) | 1.5E-06 | (0) | 6.0E-05 | (0) | 6.5E-05 | (0) | 7.2E-05 | (0) | 1.4E-03 | (0) |
| S02 | Women, normal weight | 5.8E-06 | (0) | 2.7E-01 | (0) | 1.8E-04 | (0) | 2.6E-01 | (0) | 2.7E-03 | (0) | 5.8E-06 | (0) | 6.3E-01 | (0) | 7.1E-05 | (0) | 3.9E-04 | (0) | 5.6E-02 | (0) | 4.0E-03 | (0) | 3.9E-01 | (0) |
| S03 | Women, normal weight | 2.0E-05 | (0) | 5.2E-02 | (0) | 3.6E-05 | (0) | 5.9E-03 | (0) | 2.8E-03 | (0) | 1.2E-05 | (0) | 8.1E-03 | (0) | 2.3E-05 | (0) | 7.3E-03 | (1) | 2.7E-03 | (0) | 3.9E-03 | (0) | 2.7E+01 | (3) |
| S04 | Women, normal weight | 2.9E-04 | (0) | 1.5E+00 | (1) | 3.0E-03 | (1) | 9.6E-02 | (0) | 5.6E-02 | (0) | 7.4E-04 | (0) | 3.4E-02 | (0) | 1.6E-02 | (4) | 1.5E-03 | (0) | 2.2E-01 | (0) | 1.3E-02 | (0) | 1.6E+00 | (0) |
| S05 | Women, normal weight | 1.5E-05 | (0) | 2.3E-01 | (0) | 3.0E-05 | (0) | 3.7E-02 | (0) | 3.0E-03 | (0) | 1.5E-05 | (0) | 3.0E-02 | (0) | 1.5E-05 | (0) | 4.8E-04 | (0) | 7.7E-03 | (0) | 7.8E-03 | (0) | 2.0E-01 | (0) |
| S06 | Women, normal weight | 2.1E-05 | (0) | 4.2E-01 | (0) | 2.6E-04 | (0) | 1.4E-01 | (0) | 8.2E-03 | (0) | 1.4E-05 | (0) | 3.6E-02 | (0) | 6.4E-04 | (0) | 3.9E-02 | (3) | 8.1E-01 | (0) | 1.8E+01 | (13) | 2.4E+02 | (30) |
| S07 | Women, normal weight | 1.7E-05 | (0) | 8.8E-01 | (1) | 3.1E-04 | (0) | 7.7E-02 | (0) | 1.1E-02 | (0) | 1.1E-04 | (0) | 2.2E-01 | (0) | 1.7E-04 | (0) | 8.4E-03 | (1) | 1.2E-02 | (0) | 2.0E-01 | (0) | 5.9E+02 | (74) |
|  | Children 3–6 yrs, underweight | 2.9E-05 | (0) | 1.5E+00 | (1) | 5.4E-04 | (0) | 1.3E-01 | (0) | 1.9E-02 | (0) | 1.9E-04 | (0) | 3.8E-01 | (0) | 2.8E-04 | (0) | 1.4E-02 | (1) | 2.0E-02 | (0) | 3.5E-01 | (0) | 1.0E+03 | (126) |
| S08 | Women, normal weight | 7.8E-06 | (0) | 3.6E-02 | (0) | 2.3E-05 | (0) | 6.8E-03 | (0) | 5.9E-03 | (0) | 2.5E-06 | (0) | 3.6E-03 | (0) | 6.6E-06 | (0) | 8.8E-05 | (0) | 4.4E-04 | (0) | 4.6E-03 | (0) | 1.5E+00 | (0) |
| S09 | Women, normal weight | 3.1E-05 | (0) | 5.1E-01 | (0) | 1.0E-03 | (0) | 6.8E-02 | (0) | 9.8E-03 | (0) | 1.4E-04 | (0) | 1.9E-02 | (0) | 3.4E-03 | (1) | 3.2E-02 | (2) | 8.2E-02 | (0) | 1.7E+01 | (12) | 3.0E+02 | (38) |
| S10 | Women, normal weight | 7.1E-05 | (0) | 5.0E-01 | (0) | 1.1E-03 | (0) | 1.3E-01 | (0) | 1.4E-02 | (0) | 2.3E-04 | (0) | 2.8E-02 | (0) | 4.1E-03 | (1) | 3.9E-02 | (3) | 5.6E-01 | (0) | 1.5E+01 | (11) | 2.6E+02 | (32) |
| S11 | Women, normal weight | 7.6E-05 | (0) | 2.3E-02 | (0) | 7.9E-05 | (0) | 2.8E-02 | (0) | 2.6E-03 | (0) | 1.3E-05 | (0) | 6.6E-03 | (0) | 4.3E-04 | (0) | 6.9E-04 | (0) | 1.0E-02 | (0) | 2.7E-01 | (0) | 4.7E+00 | (1) |
| S12 | Women, normal weight | 5.1E-05 | (0) | 2.4E+00 | (2) | 1.2E-02 | (4) | 1.0E+01 | (5) | 3.2E-02 | (0) | 4.7E-04 | (0) | 2.0E-02 | (0) | 9.1E-03 | (3) | 2.4E-02 | (2) | 2.8E+00 | (0) | 1.3E+01 | (9) | 3.0E+02 | (38) |
| S13 | Women, normal weight | 1.4E-04 | (0) | 5.1E-01 | (0) | 2.0E-03 | (1) | 1.3E+00 | (1) | 2.9E-02 | (0) | 1.4E-04 | (0) | 1.1E+02 | (11) | 5.7E-04 | (0) | 1.0E-03 | (0) | 3.8E-02 | (0) | 7.8E-01 | (1) | 2.7E+00 | (0) |
| S14 | Women, normal weight | 1.1E-05 | (0) | 5.0E-02 | (0) | 1.1E-04 | (0) | 6.9E-02 | (0) | 1.1E-03 | (0) | 5.5E-06 | (0) | 1.9E+00 | (0) | 1.1E-04 | (0) | 1.5E-02 | (1) | 1.1E-01 | (0) | 8.5E+00 | (6) | 1.1E+02 | (14) |
| S15 | Women, normal weight | 3.4E-05 | (0) | 3.2E-02 | (0) | 4.5E-04 | (0) | 4.4E-02 | (0) | 2.3E+00 | (1) | 9.8E-05 | (0) | 6.9E-02 | (0) | 1.6E-03 | (0) | 3.0E-03 | (0) | 2.1E-01 | (0) | 5.0E+00 | (4) | 7.0E+01 | (9) |
| S16 | Women, normal weight | 3.1E-05 | (0) | 6.1E-03 | (0) | 2.3E-04 | (0) | 3.4E-02 | (0) | 7.9E-02 | (0) | 2.0E-04 | (0) | 5.2E-02 | (0) | 5.6E-04 | (0) | 1.5E-04 | (0) | 1.1E-02 | (0) | 1.9E-01 | (0) | 2.6E+00 | (0) |
| S17 | Women, normal weight | 3.0E-05 | (0) | 6.0E-03 | (0) | 6.0E-05 | (0) | 1.5E-02 | (0) | 1.4E-02 | (0) | 3.0E-05 | (0) | 3.1E-02 | (0) | 3.0E-05 | (0) | 3.0E-05 | (0) | 1.7E-03 | (0) | 1.1E-02 | (0) | 1.6E-01 | (0) |
| S18 | Women, normal weight | 6.1E-05 | (0) | 5.2E-01 | (0) | 2.7E-03 | (1) | 4.6E-01 | (0) | 1.1E-01 | (0) | 2.0E-04 | (0) | 3.0E-02 | (0) | 5.4E-03 | (2) | 1.1E-02 | (1) | 8.3E-01 | (0) | 1.5E+01 | (11) | 2.5E+02 | (31) |
| S19 | Women, normal weight | 1.8E-04 | (0) | 2.4E+00 | (2) | 1.1E-02 | (4) | 4.9E+00 | (2) | 6.3E-02 | (0) | 6.9E-04 | (0) | 4.2E-02 | (0) | 1.2E-02 | (3) | 3.6E-03 | (0) | 9.2E-01 | (0) | 1.5E+01 | (11) | 3.2E+01 | (4) |
| S20 | Women, normal weight | 1.2E-05 | (0) | 4.3E-02 | (0) | 2.4E-04 | (0) | 1.3E-02 | (0) | 1.1E-02 | (0) | 1.4E-03 | (0) | 9.0E-03 | (0) | 7.9E-05 | (0) | 2.5E-04 | (0) | 1.3E-03 | (0) | 1.7E-02 | (0) | 4.6E-01 | (0) |
| S21 | Women, normal weight | 1.3E-05 | (0) | 8.6E-02 | (0) | 1.8E-04 | (0) | 6.5E-03 | (0) | 5.9E-03 | (0) | 1.8E-05 | (0) | 1.0E-02 | (0) | 6.8E-04 | (0) | 8.6E-05 | (0) | 8.6E-03 | (0) | 2.0E-03 | (0) | 6.6E-02 | (0) |
| S22 | Women, normal weight | 2.3E-05 | (0) | 5.4E-03 | (0) | 9.1E-05 | (0) | 5.5E-03 | (0) | 3.0E-03 | (0) | 2.4E-06 | (0) | 4.1E-03 | (0) | 1.4E-05 | (0) | 4.4E-05 | (0) | 1.6E-04 | (0) | 4.0E-04 | (0) | 7.3E-03 | (0) |
| S23 | Women, normal weight | 1.0E-04 | (0) | 1.8E+00 | (1) | 9.8E-03 | (3) | 1.3E-01 | (0) | 7.2E-02 | (0) | 3.4E-04 | (0) | 2.6E-02 | (0) | 9.2E-03 | (3) | 7.0E-03 | (1) | 9.6E-01 | (0) | 1.6E-02 | (0) | 1.8E+02 | (22) |
|  | Children 3–6 yrs, underweight | 5.1E-04 | (0) | 8.8E+00 | (6) | 4.9E-02 | (16) | 6.6E-01 | (0) | 3.6E-01 | (0) | 1.7E-03 | (0) | 1.3E-01 | (0) | 4.6E-02 | (13) | 3.5E-02 | (3) | 4.8E+00 | (0) | 8.1E-02 | (0) | 8.8E+02 | (111) |
| S24 | Women, normal weight | 4.2E-05 | (0) | 2.9E-03 | (0) | 2.9E-05 | (0) | 7.2E-03 | (0) | 2.9E-03 | (0) | 1.4E-05 | (0) | 7.2E-03 | (0) | 1.4E-05 | (0) | 2.3E-03 | (0) | 7.3E-04 | (0) | 9.3E-03 | (0) | 7.8E-03 | (0) |
|  | Children 3–6 yrs, underweight | 2.1E-04 | (0) | 1.4E-02 | (0) | 1.4E-04 | (0) | 3.6E-02 | (0) | 1.4E-02 | (0) | 7.1E-05 | (0) | 3.6E-02 | (0) | 7.1E-05 | (0) | 1.2E-02 | (1) | 3.6E-03 | (0) | 4.6E-02 | (0) | 3.9E-02 | (0) |
| S25 | Women, normal weight | 6.5E-05 | (0) | 1.4E-02 | (0) | 5.7E-05 | (0) | 1.4E-02 | (0) | 5.7E-03 | (0) | 2.9E-05 | (0) | 1.4E-02 | (0) | 2.9E-05 | (0) | 3.0E-03 | (0) | 3.6E-04 | (0) | 2.6E-03 | (0) | 5.7E-03 | (0) |
|  | Children 3–6 yrs, underweight | 3.2E-04 | (0) | 7.1E-02 | (0) | 2.8E-04 | (0) | 7.1E-02 | (0) | 2.8E-02 | (0) | 1.4E-04 | (0) | 7.1E-02 | (0) | 1.4E-04 | (0) | 1.5E-02 | (1) | 1.8E-03 | (0) | 1.3E-02 | (0) | 2.8E-02 | (0) |
| S26 | Women, normal weight | 6.7E-05 | (0) | 6.2E-02 | (0) | 1.8E-04 | (0) | 1.5E-02 | (0) | 6.1E-03 | (0) | 3.0E-05 | (0) | 1.5E-02 | (0) | 3.0E-05 | (0) | 1.3E-03 | (0) | 1.0E-01 | (0) | 2.8E+00 | (2) | 2.8E-01 | (0) |
|  | Children 3–6 yrs, underweight | 3.3E-04 | (0) | 3.1E-01 | (0) | 8.9E-04 | (0) | 7.6E-02 | (0) | 3.0E-02 | (0) | 1.5E-04 | (0) | 7.6E-02 | (0) | 1.5E-04 | (0) | 6.3E-03 | (0) | 5.1E-01 | (0) | 1.4E+01 | (10) | 1.4E+00 | (0) |
| S27 | Women, normal weight | 1.6E-05 | (0) | 2.5E-02 | (0) | 7.6E-05 | (0) | 6.6E-03 | (0) | 3.3E-03 | (0) | 2.2E-05 | (0) | 1.6E-02 | (0) | 2.4E-05 | (0) | 4.2E-05 | (0) | 1.7E-03 | (0) | 2.0E-03 | (0) | 3.5E-02 | (0) |
| S28 | Women, normal weight | 4.2E-06 | (0) | 4.6E-03 | (0) | 8.3E-05 | (0) | 5.5E-03 | (0) | 2.7E-03 | (0) | 4.2E-06 | (0) | 2.1E-03 | (0) | 1.2E-05 | (0) | 1.3E-05 | (0) | 1.8E-03 | (0) | 6.5E-04 | (0) | 6.0E-03 | (0) |
| S29 | Women, normal weight | 2.2E-04 | (0) | 9.0E-01 | (1) | 6.9E-03 | (2) | 2.3E+01 | (11) | 4.4E-02 | (0) | 2.2E-04 | (0) | 3.2E-01 | (0) | 7.9E-04 | (0) | 2.9E-02 | (2) | 5.3E+00 | (0) | 2.5E+01 | (18) | 2.1E+02 | (26) |

**Table S3.A, part 2 (of 2)**

| **Product** | **Group** | **Hg** | | **Mn** | | **Mo** | | **Ni** | | **Pb** | | **Sb** | | **Se** | | **Sn** | | **Sr** | | **U** | | **Zn** | | **REE** | |
| --- | --- | --- | --- | --- | --- | --- | --- | --- | --- | --- | --- | --- | --- | --- | --- | --- | --- | --- | --- | --- | --- | --- | --- | --- | --- |
| S01 | Women, normal weight | 1.5E-06 | (0) | 1.7E-04 | (0) | 2.8E-05 | (0) | 5.0E-04 | (0) | 2.2E-05 | (0) | 1.5E-06 | (0) | 7.7E-05 | (0) | 1.5E-06 | (0) | 1.4E-03 | (0) | 1.5E-06 | (0) | 5.2E-04 | (0) | 2.7E-05 | (0) |
| S02 | Women, normal weight | 5.8E-06 | (0) | 8.9E-02 | (0) | 6.5E-03 | (0) | 1.5E-02 | (1) | 5.3E-04 | (0) | 2.8E-05 | (0) | 6.8E-04 | (0) | 2.4E-04 | (0) | 6.1E-02 | (0) | 1.1E-04 | (0) | 6.3E-03 | (0) | 6.0E-04 | (0) |
| S03 | Women, normal weight | 3.0E-06 | (0) | 3.1E-02 | (0) | 2.2E-04 | (0) | 4.7E-03 | (0) | 2.7E-04 | (0) | 7.0E-05 | (0) | 1.5E-04 | (0) | 1.1E-04 | (0) | 1.4E-02 | (0) | 4.4E-05 | (0) | 8.4E-03 | (0) | 1.1E-03 | (0) |
| S04 | Women, normal weight | 2.2E-05 | (0) | 1.3E-01 | (0) | 4.0E-04 | (0) | 1.4E-02 | (0) | 2.8E-03 | (1) | 2.1E-04 | (0) | 1.1E-03 | (0) | 3.7E-04 | (0) | 7.8E+00 | (1) | 8.2E-03 | (0) | 1.4E-01 | (0) | 1.0E-01 | (0) |
| S05 | Women, normal weight | 1.5E-05 | (0) | 6.3E-02 | (0) | 7.6E-04 | (0) | 7.8E-03 | (0) | 6.7E-04 | (0) | 1.5E-05 | (0) | 7.5E-04 | (0) | 1.5E-05 | (0) | 1.8E-02 | (0) | 9.3E-05 | (0) | 1.2E-02 | (0) | 8.0E-04 | (0) |
| S06 | Women, normal weight | 3.5E-05 | (0) | 3.5E+01 | (25) | 9.4E-01 | (19) | 1.8E-02 | (1) | 1.3E-03 | (0) | 4.2E-05 | (0) | 9.3E-01 | (19) | 7.8E-04 | (0) | 2.9E-02 | (0) | 2.6E-04 | (0) | 1.6E+02 | (32) | 1.3E-03 | (0) |
| S07 | Women, normal weight | 1.7E-05 | (0) | 2.1E+00 | (1) | 1.1E-02 | (0) | 2.3E-02 | (1) | 2.4E-03 | (0) | 2.7E-04 | (0) | 4.2E-02 | (1) | 8.3E-05 | (0) | 1.7E-01 | (0) | 5.9E-04 | (0) | 1.8E+00 | (0) | 6.3E-03 | (0) |
|  | Children 3–6 yrs, underweight | 2.9E-05 | (0) | 3.5E+00 | (3) | 1.8E-02 | (0) | 3.9E-02 | (1) | 4.2E-03 | (1) | 4.6E-04 | (0) | 7.1E-02 | (1) | 1.4E-04 | (0) | 2.9E-01 | (0) | 1.0E-03 | (0) | 3.0E+00 | (1) | 1.1E-02 | (0) |
| S08 | Women, normal weight | 2.5E-06 | (0) | 3.3E-02 | (0) | 3.5E-04 | (0) | 5.7E-04 | (0) | 2.9E-05 | (0) | 2.5E-06 | (0) | 1.3E-04 | (0) | 2.5E-06 | (0) | 7.4E-03 | (0) | 7.0E-06 | (0) | 3.7E-02 | (0) | 1.2E-04 | (0) |
| S09 | Women, normal weight | 2.0E-05 | (0) | 1.9E+00 | (1) | 2.9E-03 | (0) | 2.5E-02 | (1) | 1.1E-03 | (0) | 4.9E-05 | (0) | 1.0E+00 | (20) | 1.7E-03 | (0) | 1.4E+00 | (0) | 1.8E-03 | (0) | 1.5E+02 | (31) | 2.1E-02 | (0) |
| S10 | Women, normal weight | 2.9E-05 | (0) | 3.7E+01 | (26) | 8.3E-01 | (17) | 2.4E-02 | (1) | 3.1E-03 | (1) | 6.5E-05 | (0) | 9.8E-01 | (20) | 2.3E-03 | (0) | 1.9E+00 | (0) | 2.2E-03 | (0) | 1.2E+02 | (24) | 2.7E-02 | (0) |
| S11 | Women, normal weight | 1.3E-05 | (0) | 5.9E-01 | (0) | 1.5E-02 | (0) | 3.1E-03 | (0) | 8.3E-04 | (0) | 1.3E-05 | (0) | 1.3E-02 | (0) | 1.3E-05 | (0) | 3.2E-02 | (0) | 1.2E-04 | (0) | 3.7E+02 | (74) | 9.8E-04 | (0) |
| S12 | Women, normal weight | 5.9E-05 | (0) | 3.9E+01 | (28) | 3.1E+00 | (62) | 1.1E-01 | (4) | 4.3E-03 | (1) | 2.2E-03 | (1) | 1.5E+00 | (30) | 3.3E-03 | (0) | 1.7E+00 | (0) | 3.0E-02 | (0) | 2.7E+02 | (54) | 1.6E-01 | (0) |
| S13 | Women, normal weight | 6.6E-04 | (0) | 4.5E-01 | (0) | 1.0E-02 | (0) | 4.3E-02 | (2) | 2.7E-03 | (1) | 1.4E-04 | (0) | 7.2E-03 | (0) | 5.0E-03 | (0) | 5.3E+01 | (9) | 1.7E-03 | (0) | 1.0E+00 | (0) | 2.1E-02 | (0) |
| S14 | Women, normal weight | 5.5E-06 | (0) | 1.4E+01 | (10) | 1.4E-03 | (0) | 1.2E-02 | (0) | 2.9E-04 | (0) | 1.3E-05 | (0) | 4.9E-01 | (10) | 2.7E-04 | (0) | 1.6E-02 | (0) | 3.4E-05 | (0) | 8.3E+01 | (17) | 7.5E-04 | (0) |
| S15 | Women, normal weight | 3.4E-05 | (0) | 9.4E+00 | (7) | 2.8E-01 | (6) | 4.5E-03 | (0) | 1.8E-03 | (0) | 3.4E-05 | (0) | 1.1E+00 | (21) | 1.3E-03 | (0) | 1.7E+00 | (0) | 3.9E-04 | (0) | 2.0E+02 | (40) | 5.7E-01 | (1) |
| S16 | Women, normal weight | 3.1E-05 | (0) | 3.7E-01 | (0) | 1.2E-02 | (0) | 1.5E-03 | (0) | 1.9E-03 | (0) | 2.1E-04 | (0) | 4.8E-02 | (1) | 4.0E-04 | (0) | 2.3E-01 | (0) | 2.6E-04 | (0) | 8.7E+00 | (2) | 1.6E-02 | (0) |
| S17 | Women, normal weight | 3.0E-05 | (0) | 2.2E-02 | (0) | 8.3E-04 | (0) | 1.5E-03 | (0) | 6.7E-05 | (0) | 3.0E-05 | (0) | 1.5E-03 | (0) | 3.0E-05 | (0) | 3.2E-02 | (0) | 3.0E-05 | (0) | 4.3E-01 | (0) | 1.7E-03 | (0) |
| S18 | Women, normal weight | 2.3E-04 | (0) | 3.1E+01 | (22) | 2.7E-01 | (5) | 6.5E-02 | (2) | 1.9E-03 | (0) | 4.1E-04 | (0) | 7.6E-01 | (15) | 6.1E-05 | (0) | 1.6E+00 | (0) | 2.5E-03 | (0) | 1.3E+02 | (25) | 5.3E-02 | (0) |
| S19 | Women, normal weight | 3.2E-05 | (0) | 1.8E+01 | (13) | 1.5E+00 | (30) | 2.0E-02 | (1) | 5.1E-03 | (1) | 3.5E-03 | (1) | 9.2E-01 | (18) | 5.1E-04 | (0) | 2.2E+00 | (0) | 2.0E-02 | (0) | 2.2E+02 | (44) | 1.1E-01 | (0) |
| S20 | Women, normal weight | 2.6E-06 | (0) | 2.1E-01 | (0) | 1.5E-04 | (0) | 7.7E-04 | (0) | 1.3E-03 | (0) | 5.1E-03 | (1) | 1.3E-03 | (0) | 1.1E-04 | (0) | 2.2E-01 | (0) | 7.7E-04 | (0) | 8.3E+01 | (17) | 1.2E-02 | (0) |
| S21 | Women, normal weight | 1.5E-06 | (0) | 5.6E-03 | (0) | 7.5E-05 | (0) | 8.5E-04 | (0) | 1.6E-04 | (0) | 3.7E-05 | (0) | 1.5E-04 | (0) | 3.6E-05 | (0) | 1.9E-01 | (0) | 3.9E-04 | (0) | 9.1E-02 | (0) | 3.7E-03 | (0) |
| S22 | Women, normal weight | 7.2E-06 | (0) | 6.5E-04 | (0) | 5.5E-04 | (0) | 2.9E-04 | (0) | 1.3E-05 | (0) | 1.8E-05 | (0) | 1.1E+00 | (21) | 2.4E-06 | (0) | 7.2E-04 | (0) | 6.0E-05 | (0) | 1.2E-02 | (0) | 7.4E-05 | (0) |
| S23 | Women, normal weight | 1.3E-04 | (0) | 1.5E+01 | (11) | 2.2E+00 | (43) | 3.2E-02 | (1) | 2.1E-03 | (0) | 2.9E-03 | (1) | 4.8E-01 | (10) | 8.5E-04 | (0) | 1.7E+00 | (0) | 9.0E-03 | (0) | 1.2E+02 | (23) | 1.4E-02 | (0) |
|  | Children 3–6 yrs, underweight | 6.5E-04 | (0) | 7.6E+01 | (55) | 1.1E+01 | (215) | 1.6E-01 | (6) | 1.1E-02 | (2) | 1.4E-02 | (4) | 2.4E+00 | (48) | 4.2E-03 | (0) | 8.7E+00 | (1) | 4.5E-02 | (0) | 5.8E+02 | (116) | 6.9E-02 | (0) |
| S24 | Women, normal weight | 1.4E-05 | (0) | 1.4E-03 | (0) | 7.2E-05 | (0) | 7.2E-04 | (0) | 1.4E-04 | (0) | 4.1E-05 | (0) | 7.2E-04 | (0) | 4.0E-04 | (0) | 1.4E-02 | (0) | 7.9E-05 | (0) | 4.9E-03 | (0) | 2.3E-04 | (0) |
|  | Children 3–6 yrs, underweight | 7.1E-05 | (0) | 6.8E-03 | (0) | 3.6E-04 | (0) | 3.6E-03 | (0) | 7.1E-04 | (0) | 2.1E-04 | (0) | 3.6E-03 | (0) | 2.0E-03 | (0) | 6.8E-02 | (0) | 3.9E-04 | (0) | 2.4E-02 | (0) | 1.1E-03 | (0) |
| S25 | Women, normal weight | 2.9E-05 | (0) | 1.3E-02 | (0) | 1.4E-04 | (0) | 1.4E-03 | (0) | 1.9E-04 | (0) | 6.4E-05 | (0) | 1.4E-03 | (0) | 2.9E-05 | (0) | 4.5E-03 | (0) | 2.9E-05 | (0) | 2.9E-03 | (0) | 4.6E-04 | (0) |
|  | Children 3–6 yrs, underweight | 1.4E-04 | (0) | 6.7E-02 | (0) | 7.1E-04 | (0) | 7.1E-03 | (0) | 9.4E-04 | (0) | 3.2E-04 | (0) | 7.1E-03 | (0) | 1.4E-04 | (0) | 2.2E-02 | (0) | 1.4E-04 | (0) | 1.4E-02 | (0) | 2.3E-03 | (0) |
| S26 | Women, normal weight | 3.0E-05 | (0) | 3.4E-02 | (0) | 1.5E-04 | (0) | 4.8E-03 | (0) | 1.3E-03 | (0) | 1.3E-04 | (0) | 1.5E-01 | (3) | 5.1E-03 | (0) | 2.9E-02 | (0) | 2.3E-04 | (0) | 2.5E+01 | (5) | 5.7E-04 | (0) |
|  | Children 3–6 yrs, underweight | 1.5E-04 | (0) | 1.7E-01 | (0) | 7.6E-04 | (0) | 2.4E-02 | (1) | 6.6E-03 | (1) | 6.5E-04 | (0) | 7.4E-01 | (15) | 2.5E-02 | (0) | 1.4E-01 | (0) | 1.2E-03 | (0) | 1.2E+02 | (25) | 2.8E-03 | (0) |
| S27 | Women, normal weight | 3.5E-06 | (0) | 1.1E-03 | (0) | 5.3E-04 | (0) | 6.6E-04 | (0) | 1.1E-04 | (0) | 7.3E-05 | (0) | 2.2E+00 | (45) | 9.3E-05 | (0) | 3.0E-03 | (0) | 3.5E-06 | (0) | 1.9E-02 | (0) | 1.2E-04 | (0) |
| S28 | Women, normal weight | 4.2E-06 | (0) | 4.0E-04 | (0) | 1.3E-04 | (0) | 2.1E-04 | (0) | 4.6E-05 | (0) | 3.1E-05 | (0) | 2.1E-04 | (0) | 3.4E-05 | (0) | 5.3E-04 | (0) | 4.2E-06 | (0) | 4.2E-04 | (0) | 8.7E-05 | (0) |
| S29 | Women, normal weight | 2.2E-04 | (0) | 3.2E+01 | (23) | 1.1E-03 | (0) | 3.9E-02 | (1) | 1.3E-02 | (3) | 1.6E-03 | (0) | 1.5E+00 | (30) | 3.6E-03 | (0) | 7.8E-01 | (0) | 8.1E-04 | (0) | 2.3E+02 | (46) | 2.5E-02 | (0) |

**Table S3.B, part 1 (of 2)** Average Daily Doses (ADDs; µg/kg/day) of all elements with available TDIs, associated with each investigated ***marine non-fish-oil*** food supplement (M01-M38). Values in parentheses = ADDs in % of TDIs.

| **Product** | **Group** | **Ag** | | **Al** | | **As** | | **B** | | **Ba** | | **Be** | | **Br** | | **Cd** | | **Co** | | **Cr** | | | **Cu** | | | **Fe** | |
| --- | --- | --- | --- | --- | --- | --- | --- | --- | --- | --- | --- | --- | --- | --- | --- | --- | --- | --- | --- | --- | --- | --- | --- | --- | --- | --- | --- |
| M01 | Women, normal weight | 7.8E-05 | (0) | 1.7E+00 | (1) | 5.1E-02 | (17) | 1.2E-01 | (0) | 4.8E-02 | (0) | 3.4E-04 | (0) | 7.8E-01 | (0) | 5.3E-03 | (1) | 8.0E-03 | (1) | 3.2E-02 | (0) | 6.3E-02 | | (0) | 2.6E+00 | | (0) |
| M02 | Women, normal weight | 2.6E-03 | (0) | 1.2E+01 | (9) | 2.9E-01 | (96) | 1.3E+00 | (1) | 2.9E-01 | (0) | 7.3E-04 | (0) | 1.2E+00 | (0) | 3.9E-03 | (1) | 5.6E-02 | (4) | 2.6E-02 | (0) | 2.8E-01 | | (0) | 8.6E+01 | | (11) |
|  | Children 3–6 yrs, underweight | 1.3E-02 | (0) | 6.2E+01 | (43) | 1.4E+00 | (480) | 6.6E+00 | (3) | 1.5E+00 | (1) | 3.6E-03 | (0) | 6.0E+00 | (1) | 1.9E-02 | (5) | 2.8E-01 | (20) | 1.3E-01 | (0) | 1.4E+00 | | (1) | 4.3E+02 | | (54) |
| M03 | Women, normal weight | 6.4E-04 | (0) | 3.3E+01 | (23) | 4.1E-02 | (14) | 2.1E+00 | (1) | 1.3E+00 | (1) | 1.7E-03 | (0) | 1.9E+00 | (0) | 2.1E-02 | (6) | 4.2E-02 | (3) | 1.3E-01 | (0) | 1.5E+00 | | (1) | 5.1E+01 | | (6) |
|  | Children 3–6 yrs, underweight | 3.2E-03 | (0) | 1.6E+02 | (115) | 2.0E-01 | (68) | 1.0E+01 | (5) | 6.7E+00 | (3) | 8.3E-03 | (0) | 9.6E+00 | (1) | 1.0E-01 | (29) | 2.1E-01 | (15) | 6.6E-01 | (0) | 7.4E+00 | | (5) | 2.5E+02 | | (31) |
| M04 | Women, normal weight | 3.9E-03 | (0) | 7.1E+00 | (5) | 2.2E-02 | (7) | 9.6E-01 | (0) | 2.0E+00 | (1) | 2.3E-03 | (0) | 1.8E-01 | (0) | 5.1E-03 | (1) | 2.7E-02 | (2) | 7.7E-02 | (0) | 2.0E-01 | | (0) | 6.9E+01 | | (9) |
| M05 | Women, normal weight | 3.1E-04 | (0) | 1.5E+00 | (1) | 1.7E-01 | (56) | 9.8E-01 | (0) | 8.7E-02 | (0) | 3.2E-04 | (0) | 5.3E+00 | (1) | 4.1E-03 | (1) | 4.8E-03 | (0) | 1.3E-02 | (0) | 1.1E-02 | | (0) | 4.5E+00 | | (1) |
| M06 | Women, normal weight | 7.5E-05 | (0) | 4.0E-01 | (0) | 1.8E-02 | (6) | 1.1E-01 | (0) | 1.1E-02 | (0) | 3.3E-05 | (0) | 4.3E-01 | (0) | 3.6E-04 | (0) | 1.0E-03 | (0) | 2.5E-03 | (0) | 1.2E-03 | | (0) | 6.2E-01 | | (0) |
| M07 | Women, normal weight | 1.1E-04 | (0) | 1.1E+00 | (1) | 7.4E-02 | (25) | 3.1E-01 | (0) | 5.3E-02 | (0) | 7.4E-06 | (0) | 3.2E+00 | (0) | 1.1E-02 | (3) | 1.0E-03 | (0) | 2.2E-03 | (0) | 7.9E-03 | | (0) | 9.8E-01 | | (0) |
| M08 | Women, normal weight | 3.7E-04 | (0) | 3.5E-01 | (0) | 1.2E-02 | (4) | 3.1E-01 | (0) | 8.4E-02 | (0) | 3.0E-05 | (0) | 4.1E-01 | (0) | 1.8E-03 | (1) | 4.4E-03 | (0) | 2.0E-02 | (0) | 2.9E-01 | | (0) | 1.7E+01 | | (2) |
| M09 | Women, normal weight | 4.1E-04 | (0) | 5.8E+00 | (4) | 1.4E-01 | (46) | 5.0E-01 | (0) | 5.8E-02 | (0) | 2.6E-04 | (0) | 3.9E+00 | (0) | 6.9E-03 | (2) | 4.2E-02 | (3) | 1.5E-02 | (0) | 3.8E-02 | | (0) | 1.6E+02 | | (20) |
| M10 | Women, normal weight | 1.5E-04 | (0) | 4.0E-01 | (0) | 4.1E-04 | (0) | 4.8E-02 | (0) | 2.5E-02 | (0) | 5.6E-05 | (0) | 9.5E-03 | (0) | 8.3E-04 | (0) | 2.4E-02 | (2) | 1.9E-02 | (0) | 2.6E-02 | | (0) | 2.0E+02 | | (25) |
|  | Children 3–6 yrs, underweight | 3.9E-04 | (0) | 1.0E+00 | (1) | 1.0E-03 | (0) | 1.2E-01 | (0) | 6.2E-02 | (0) | 1.4E-04 | (0) | 2.4E-02 | (0) | 2.1E-03 | (1) | 6.1E-02 | (4) | 4.7E-02 | (0) | 6.5E-02 | | (0) | 4.9E+02 | | (61) |
| M11 | Women, normal weight | 1.3E-03 | (0) | 5.3E+00 | (4) | 3.8E-01 | (128) | 5.0E-01 | (0) | 1.1E-01 | (0) | 1.0E-03 | (0) | 1.3E+00 | (0) | 2.9E-02 | (8) | 1.5E-02 | (1) | 2.7E-02 | (0) | 1.1E-01 | | (0) | 7.2E+00 | | (1) |
| M12 | Women, normal weight | 1.9E-04 | (0) | 1.6E+00 | (1) | 6.1E-02 | (20) | 1.6E-01 | (0) | 2.4E-02 | (0) | 5.1E-05 | (0) | 1.6E+00 | (0) | 1.9E-03 | (1) | 5.4E-03 | (0) | 6.6E-03 | (0) | 2.1E-02 | | (0) | 3.3E+00 | | (0) |
| M13 | Women, normal weight | 7.6E-04 | (0) | 7.5E+00 | (5) | 1.2E-02 | (4) | 4.9E-01 | (0) | 3.4E-01 | (0) | 3.0E-04 | (0) | 4.5E-01 | (0) | 3.5E-03 | (1) | 1.7E-02 | (1) | 3.3E-02 | (0) | 1.6E-01 | | (0) | 2.6E+01 | | (3) |
| M14 | Women, normal weight | 1.3E-04 | (0) | 4.0E-01 | (0) | 2.4E-02 | (8) | 6.4E-01 | (0) | 1.3E-01 | (0) | 4.2E-05 | (0) | 2.5E-01 | (0) | 4.6E-04 | (0) | 2.4E-02 | (2) | 2.1E-02 | (0) | 4.1E-01 | | (0) | 4.3E+01 | | (5) |
| M15 | Women, normal weight | 3.9E-05 | (0) | 4.1E+00 | (3) | 4.6E-03 | (2) | 9.4E-02 | (0) | 8.6E-02 | (0) | 2.2E-04 | (0) | 7.4E-02 | (0) | 5.3E-04 | (0) | 2.5E-03 | (0) | 1.0E-02 | (0) | 8.3E-02 | | (0) | 4.0E+00 | | (0) |
| M16 | Women, normal weight | 6.2E-05 | (0) | 1.8E+00 | (1) | 1.4E-03 | (0) | 1.0E-01 | (0) | 7.8E-02 | (0) | 1.3E-04 | (0) | 5.4E-02 | (0) | 7.1E-04 | (0) | 5.4E-03 | (0) | 2.4E-01 | (0) | 4.9E+00 | | (4) | 8.1E+01 | | (10) |
|  | Children 3–6 yrs, underweight | 3.1E-04 | (0) | 9.2E+00 | (6) | 7.2E-03 | (2) | 5.2E-01 | (0) | 3.9E-01 | (0) | 6.6E-04 | (0) | 2.7E-01 | (0) | 3.6E-03 | (1) | 2.7E-02 | (2) | 1.2E+00 | (0) | 2.4E+01 | | (17) | 4.0E+02 | | (50) |
| M17 | Women, normal weight | 8.3E-05 | (0) | 8.0E-02 | (0) | 1.0E-03 | (0) | 8.3E-03 | (0) | 1.6E-02 | (0) | 5.2E-05 | (0) | 1.2E-02 | (0) | 5.3E-05 | (0) | 4.8E-02 | (3) | 2.8E-03 | (0) | 3.1E+00 | | (2) | 8.7E-01 | | (0) |
| M18 | Women, normal weight | 8.0E-06 | (0) | 6.2E-03 | (0) | 1.8E-05 | (0) | 1.2E-02 | (0) | 2.6E-03 | (0) | 4.0E-06 | (0) | 1.2E-02 | (0) | 2.7E-05 | (0) | 3.3E-05 | (0) | 2.9E-04 | (0) | 3.2E-04 | | (0) | 5.6E-03 | | (0) |
| M19 | Women, normal weight | 1.8E-05 | (0) | 4.5E-02 | (0) | 6.1E-04 | (0) | 1.1E-01 | (0) | 3.5E-03 | (0) | 1.8E-05 | (0) | 3.0E-02 | (0) | 5.7E-04 | (0) | 3.4E-04 | (0) | 1.1E-03 | (0) | 9.1E-04 | | (0) | 9.7E-02 | | (0) |
| M20 | Women, normal weight | 6.5E-06 | (0) | 1.6E-02 | (0) | 8.3E-05 | (0) | 5.3E-02 | (0) | 9.4E-03 | (0) | 6.5E-06 | (0) | 3.3E-03 | (0) | 1.7E-04 | (0) | 1.4E-05 | (0) | 1.3E-04 | (0) | 1.1E-04 | | (0) | 4.4E-03 | | (0) |
| M21 | Women, normal weight | 3.6E-04 | (0) | 5.5E+00 | (4) | 2.8E-02 | (9) | 3.0E-01 | (0) | 1.3E-01 | (0) | 2.9E-04 | (0) | 7.5E-02 | (0) | 1.1E-03 | (0) | 3.6E-02 | (3) | 2.8E-02 | (0) | 5.3E-02 | | (0) | 7.0E+01 | | (9) |
|  | Children 3–6 yrs, underweight | 1.8E-03 | (0) | 2.7E+01 | (19) | 1.4E-01 | (46) | 1.5E+00 | (1) | 6.5E-01 | (0) | 1.4E-03 | (0) | 3.7E-01 | (0) | 5.6E-03 | (2) | 1.8E-01 | (13) | 1.4E-01 | (0) | 2.6E-01 | | (0) | 3.5E+02 | | (44) |
| M22 | Women, normal weight | 1.7E-04 | (0) | 1.2E+01 | (9) | 3.5E-02 | (12) | 3.1E-01 | (0) | 1.4E+00 | (1) | 6.4E-04 | (0) | 5.7E-02 | (0) | 1.6E-03 | (0) | 1.3E-01 | (9) | 3.2E-02 | (0) | 1.9E-01 | | (0) | 1.8E+02 | | (22) |
|  | Children 3–6 yrs, underweight | 8.3E-04 | (0) | 6.1E+01 | (42) | 1.7E-01 | (58) | 1.5E+00 | (1) | 7.0E+00 | (3) | 3.2E-03 | (0) | 2.8E-01 | (0) | 8.1E-03 | (2) | 6.2E-01 | (45) | 1.6E-01 | (0) | 9.2E-01 | | (1) | 8.9E+02 | | (111) |
| M23 | Women, normal weight | 1.0E-02 | (0) | 1.8E+01 | (13) | 1.3E-02 | (4) | 8.8E-01 | (0) | 1.0E+00 | (1) | 3.9E-03 | (0) | 1.0E-01 | (0) | 6.2E-03 | (2) | 2.4E-02 | (2) | 9.2E-02 | (0) | 3.3E-01 | | (0) | 1.3E+02 | | (17) |
|  | Children 3–6 yrs, underweight | 5.1E-02 | (1) | 9.0E+01 | (63) | 6.4E-02 | (21) | 4.4E+00 | (2) | 5.1E+00 | (3) | 1.9E-02 | (1) | 5.0E-01 | (0) | 3.1E-02 | (9) | 1.2E-01 | (8) | 4.6E-01 | (0) | 1.6E+00 | | (1) | 6.6E+02 | | (82) |
| M24 | Women, normal weight | 3.0E-03 | (0) | 1.1E+01 | (8) | 1.8E-02 | (6) | 8.5E-01 | (0) | 1.2E+00 | (1) | 4.1E-03 | (0) | 7.8E-02 | (0) | 3.0E-03 | (1) | 2.7E-02 | (2) | 1.3E-01 | (0) | 4.0E-01 | | (0) | 1.1E+02 | | (14) |
|  | Children 3–6 yrs, underweight | 1.5E-02 | (0) | 5.7E+01 | (40) | 9.0E-02 | (30) | 4.2E+00 | (2) | 5.7E+00 | (3) | 2.0E-02 | (1) | 3.9E-01 | (0) | 1.5E-02 | (4) | 1.3E-01 | (10) | 6.4E-01 | (0) | 2.0E+00 | | (1) | 5.6E+02 | | (70) |
| M25 | Women, normal weight | 4.7E-04 | (0) | 3.8E+00 | (3) | 3.1E-02 | (10) | 1.8E-01 | (0) | 1.1E-01 | (0) | 1.6E-04 | (0) | 1.4E-01 | (0) | 8.3E-04 | (0) | 2.3E-02 | (2) | 1.4E-02 | (0) | 5.3E-02 | | (0) | 4.0E+01 | | (5) |
| M26 | Women, normal weight | 2.5E-04 | (0) | 2.0E+00 | (1) | 1.9E-02 | (6) | 1.2E-01 | (0) | 6.7E-02 | (0) | 8.6E-05 | (0) | 6.1E-02 | (0) | 4.3E-04 | (0) | 4.1E-02 | (3) | 1.1E-02 | (0) | 2.0E-02 | | (0) | 4.6E+01 | | (6) |
|  | Children 3–6 yrs, underweight | 1.2E-03 | (0) | 9.8E+00 | (7) | 9.2E-02 | (31) | 6.0E-01 | (0) | 3.3E-01 | (0) | 4.3E-04 | (0) | 3.0E-01 | (0) | 2.2E-03 | (1) | 2.1E-01 | (15) | 5.4E-02 | (0) | 9.8E-02 | | (0) | 2.3E+02 | | (29) |
| M27 | Women, normal weight | 1.4E-03 | (0) | 9.0E+01 | (63) | 1.1E-01 | (36) | 1.9E+00 | (1) | 7.9E+00 | (4) | 1.8E-03 | (0) | 1.2E+00 | (0) | 1.8E-02 | (5) | 1.6E-01 | (11) | 2.2E-01 | (0) | 3.3E+00 | | (2) | 2.1E+02 | | (26) |
|  | Children 3–6 yrs, underweight | 7.1E-03 | (0) | 4.5E+02 | (312) | 5.3E-01 | (178) | 9.6E+00 | (5) | 3.9E+01 | (20) | 8.7E-03 | (0) | 5.8E+00 | (1) | 9.0E-02 | (25) | 7.8E-01 | (56) | 1.1E+00 | (0) | 1.7E+01 | | (12) | 1.0E+03 | | (128) |
| M28 | Women, normal weight | 5.9E-05 | (0) | 3.9E-01 | (0) | 7.0E-02 | (23) | 6.3E-01 | (0) | 3.1E-01 | (0) | 3.9E-04 | (0) | 1.2E-01 | (0) | 7.5E-04 | (0) | 5.4E-02 | (4) | 3.0E-02 | (0) | 7.9E-01 | | (1) | 9.8E+01 | | (12) |
|  | Children 3–6 yrs, underweight | 2.9E-04 | (0) | 1.9E+00 | (1) | 3.5E-01 | (116) | 3.1E+00 | (2) | 1.5E+00 | (1) | 2.0E-03 | (0) | 6.2E-01 | (0) | 3.7E-03 | (1) | 2.7E-01 | (19) | 1.5E-01 | (0) | 3.9E+00 | | (3) | 4.9E+02 | | (61) |
| M29 | Women, normal weight | 4.2E-04 | (0) | 2.3E+00 | (2) | 2.5E-02 | (8) | 6.6E-01 | (0) | 2.4E+00 | (1) | 5.9E-04 | (0) | 4.9E-01 | (0) | 2.4E-03 | (1) | 1.7E-02 | (1) | 9.0E-02 | (0) | 2.7E-01 | | (0) | 6.2E+01 | | (8) |
|  | Children 3–6 yrs, underweight | 2.1E-03 | (0) | 1.2E+01 | (8) | 1.2E-01 | (41) | 3.3E+00 | (2) | 1.2E+01 | (6) | 3.0E-03 | (0) | 2.4E+00 | (0) | 1.2E-02 | (3) | 8.4E-02 | (6) | 4.5E-01 | (0) | 1.4E+00 | | (1) | 3.1E+02 | | (39) |
| M30 | Women, normal weight | 6.1E-04 | (0) | 2.0E+01 | (14) | 2.1E-02 | (7) | 1.0E+00 | (1) | 5.2E-01 | (0) | 1.0E-03 | (0) | 6.7E-01 | (0) | 1.4E-02 | (4) | 1.5E-02 | (1) | 6.4E-02 | (0) | 4.8E-01 | | (0) | 3.1E+01 | | (4) |
|  | Children 3–6 yrs, underweight | 3.0E-03 | (0) | 1.0E+02 | (71) | 1.0E-01 | (34) | 5.0E+00 | (3) | 2.6E+00 | (1) | 5.1E-03 | (0) | 3.3E+00 | (0) | 6.9E-02 | (19) | 7.5E-02 | (5) | 3.2E-01 | (0) | 2.4E+00 | | (2) | 1.5E+02 | | (19) |
| M31 | Women, normal weight | 1.7E-03 | (0) | 3.4E+01 | (24) | 2.4E-02 | (8) | 2.0E+00 | (1) | 1.3E+00 | (1) | 1.5E-03 | (0) | 1.1E+00 | (0) | 2.5E-02 | (7) | 4.2E-02 | (3) | 8.4E-02 | (0) | 9.9E-01 | | (1) | 6.4E+01 | | (8) |
|  | Children 3–6 yrs, underweight | 8.4E-03 | (0) | 1.7E+02 | (117) | 1.2E-01 | (39) | 1.0E+01 | (5) | 6.6E+00 | (3) | 7.4E-03 | (0) | 5.3E+00 | (1) | 1.2E-01 | (34) | 2.1E-01 | (15) | 4.2E-01 | (0) | 4.9E+00 | | (4) | 3.2E+02 | | (40) |
| M32 | Women, normal weight | 6.7E-03 | (0) | 8.6E+00 | (6) | 3.1E-02 | (10) | 6.7E+00 | (3) | 3.0E+00 | (2) | 7.4E-04 | (0) | 3.0E+00 | (0) | 1.3E-01 | (35) | 1.4E-01 | (10) | 1.3E-01 | (0) | 9.9E+00 | | (7) | 1.2E+02 | | (15) |
|  | Children 3–6 yrs, underweight | 2.3E-02 | (0) | 3.0E+01 | (21) | 1.1E-01 | (37) | 2.3E+01 | (12) | 1.1E+01 | (5) | 2.6E-03 | (0) | 1.1E+01 | (1) | 4.4E-01 | (123) | 4.8E-01 | (34) | 4.6E-01 | (0) | 3.4E+01 | | (25) | 4.1E+02 | | (52) |
| M33 | Women, normal weight | 1.5E-05 | (0) | 4.7E-02 | (0) | 1.2E+00 | (394) | 4.0E+00 | (2) | 2.1E-01 | (0) | 1.5E-05 | (0) | 1.4E+01 | (1) | 7.7E-03 | (2) | 2.3E-03 | (0) | 1.6E-03 | (0) | 1.2E-02 | | (0) | 7.3E-01 | | (0) |
|  | Children 3–6 yrs, underweight | 7.4E-05 | (0) | 2.3E-01 | (0) | 5.9E+00 | (1962) | 2.0E+01 | (10) | 1.1E+00 | (1) | 7.4E-05 | (0) | 6.9E+01 | (7) | 3.8E-02 | (11) | 1.1E-02 | (1) | 7.7E-03 | (0) | 6.1E-02 | | (0) | 3.6E+00 | | (0) |
| M34 | Women, normal weight | 1.4E-04 | (0) | 1.9E+00 | (1) | 2.0E-01 | (67) | 3.0E-01 | (0) | 1.3E-02 | (0) | 9.4E-05 | (0) | 2.5E+00 | (0) | 2.1E-02 | (6) | 8.3E-03 | (1) | 1.4E-02 | (0) | 7.1E-02 | | (0) | 2.3E+00 | | (0) |
| M35 | Women, normal weight | 1.1E-05 | (0) | 2.2E-02 | (0) | 1.0E-03 | (0) | 3.5E-02 | (0) | 4.9E-03 | (0) | 1.1E-05 | (0) | 8.1E-02 | (0) | 1.1E-05 | (0) | 9.7E-05 | (0) | 2.2E-03 | (0) | 2.8E-03 | | (0) | 3.4E-02 | | (0) |
| M36 | Women, normal weight | 2.2E-05 | (0) | 2.4E-02 | (0) | 1.1E-03 | (0) | 1.1E-02 | (0) | 4.4E-03 | (0) | 2.2E-05 | (0) | 1.0E-01 | (0) | 7.0E-05 | (0) | 1.2E-04 | (0) | 2.4E-03 | (0) | 8.7E-03 | | (0) | 1.1E-01 | | (0) |
| M37 | Women, normal weight | 8.7E-06 | (0) | 6.8E-03 | (0) | 1.5E-04 | (0) | 4.3E-03 | (0) | 2.6E-01 | (0) | 8.7E-06 | (0) | 1.0E-01 | (0) | 8.7E-06 | (0) | 6.9E-05 | (0) | 3.9E-03 | (0) | 3.3E-04 | | (0) | 4.0E-02 | | (0) |
| M38 | Women, normal weight | 1.5E-05 | (0) | 6.9E-02 | (0) | 1.0E-03 | (0) | 3.9E-01 | (0) | 2.9E-03 | (0) | 1.5E-05 | (0) | 8.0E-01 | (0) | 1.6E-04 | (0) | 4.8E-03 | (0) | 1.6E-02 | (0) | 2.7E-03 | | (0) | 1.5E-01 | | (0) |
|  | Children 3–6 yrs, underweight | 7.2E-05 | (0) | 3.4E-01 | (0) | 5.2E-03 | (2) | 1.9E+00 | (1) | 1.4E-02 | (0) | 7.2E-05 | (0) | 4.0E+00 | (0) | 7.8E-04 | (0) | 2.4E-02 | (2) | 7.9E-02 | (0) | 1.4E-02 | | (0) | 7.7E-01 | | (0) |

**Table S3.B, part 2 (of 2)**

| **Product** | **Group** | **Hg** | | **Mn** | | **Mo** | | **Ni** | | **Pb** | | **Sb** | | **Se** | | **Sn** | | **Sr** | | **U** | | **Zn** | | **REE** | |
| --- | --- | --- | --- | --- | --- | --- | --- | --- | --- | --- | --- | --- | --- | --- | --- | --- | --- | --- | --- | --- | --- | --- | --- | --- | --- |
| M01 | Women, normal weight | 4.4E-04 | (0) | 1.2E+00 | (1) | 1.3E-02 | (0) | 3.6E-02 | (1) | 8.5E-03 | (2) | 4.0E-03 | (1) | 1.1E-02 | (0) | 6.2E-04 | (0) | 5.4E-01 | (0) | 5.4E-03 | (0) | 3.8E-01 | (0) | 1.7E-02 | (0) |
| M02 | Women, normal weight | 4.1E-04 | (0) | 5.0E+00 | (4) | 2.8E-02 | (1) | 2.1E-01 | (8) | 2.8E-02 | (6) | 2.8E-03 | (1) | 7.8E-02 | (2) | 2.7E-03 | (0) | 3.0E+00 | (0) | 3.8E-02 | (0) | 3.3E+00 | (1) | 7.9E-02 | (0) |
|  | Children 3–6 yrs, underweight | 2.1E-03 | (1) | 2.5E+01 | (18) | 1.4E-01 | (3) | 1.1E+00 | (38) | 1.4E-01 | (28) | 1.4E-02 | (4) | 3.9E-01 | (8) | 1.3E-02 | (0) | 1.5E+01 | (2) | 1.9E-01 | (0) | 1.6E+01 | (3) | 3.9E-01 | (1) |
| M03 | Women, normal weight | 6.9E-04 | (0) | 4.6E+00 | (3) | 1.2E-01 | (2) | 1.5E-01 | (5) | 7.2E-02 | (14) | 2.7E-03 | (1) | 1.4E-02 | (0) | 5.8E-03 | (0) | 3.8E+00 | (1) | 7.2E-03 | (0) | 3.4E+00 | (1) | 1.6E-01 | (0) |
|  | Children 3–6 yrs, underweight | 3.4E-03 | (2) | 2.3E+01 | (16) | 5.9E-01 | (12) | 7.6E-01 | (27) | 3.6E-01 | (71) | 1.3E-02 | (3) | 7.0E-02 | (1) | 2.9E-02 | (0) | 1.9E+01 | (3) | 3.6E-02 | (0) | 1.7E+01 | (3) | 7.9E-01 | (1) |
| M04 | Women, normal weight | 3.6E-05 | (0) | 3.5E+00 | (2) | 1.0E-02 | (0) | 8.2E-02 | (3) | 9.0E-02 | (18) | 2.3E-03 | (1) | 1.7E-02 | (0) | 4.7E-03 | (0) | 2.3E+00 | (0) | 5.3E-03 | (0) | 1.3E+00 | (0) | 1.9E-01 | (0) |
| M05 | Women, normal weight | 1.2E-04 | (0) | 9.9E-01 | (1) | 3.9E-03 | (0) | 2.7E-02 | (1) | 3.3E-03 | (1) | 5.5E-03 | (1) | 4.2E-04 | (0) | 3.4E-04 | (0) | 5.3E+00 | (1) | 5.6E-03 | (0) | 1.4E-01 | (0) | 1.3E-01 | (0) |
| M06 | Women, normal weight | 2.2E-05 | (0) | 4.1E-02 | (0) | 5.4E-03 | (0) | 1.8E-03 | (0) | 2.6E-04 | (0) | 1.4E-04 | (0) | 9.0E-05 | (0) | 3.4E-04 | (0) | 5.4E-01 | (0) | 1.6E-03 | (0) | 3.2E-02 | (0) | 7.8E-03 | (0) |
| M07 | Women, normal weight | 2.9E-05 | (0) | 2.4E-02 | (0) | 1.2E-03 | (0) | 5.0E-03 | (0) | 7.6E-04 | (0) | 1.6E-04 | (0) | 3.7E-04 | (0) | 1.9E-04 | (0) | 2.1E+00 | (0) | 8.6E-04 | (0) | 2.4E-02 | (0) | 3.6E-03 | (0) |
| M08 | Women, normal weight | 2.1E-04 | (0) | 2.4E+00 | (2) | 2.5E-02 | (0) | 2.0E-02 | (1) | 3.9E-03 | (1) | 5.8E-04 | (0) | 1.5E-03 | (0) | 4.9E-04 | (0) | 1.0E+00 | (0) | 6.4E-04 | (0) | 1.0E+00 | (0) | 1.9E-02 | (0) |
| M09 | Women, normal weight | 1.8E-04 | (0) | 9.3E-01 | (1) | 4.6E-03 | (0) | 5.6E-02 | (2) | 5.4E-03 | (1) | 8.3E-03 | (2) | 3.1E-03 | (0) | 4.8E-04 | (0) | 3.2E+00 | (1) | 3.3E-03 | (0) | 1.8E+02 | (36) | 5.7E-02 | (0) |
| M10 | Women, normal weight | 5.4E-05 | (0) | 3.3E+01 | (23) | 6.7E-03 | (0) | 2.8E-02 | (1) | 7.6E-04 | (0) | 2.6E-04 | (0) | 9.4E-01 | (19) | 1.3E-04 | (0) | 2.0E-01 | (0) | 9.0E-04 | (0) | 3.8E+01 | (8) | 7.0E-03 | (0) |
|  | Children 3–6 yrs, underweight | 1.3E-04 | (0) | 8.2E+01 | (59) | 1.7E-02 | (0) | 7.1E-02 | (3) | 1.9E-03 | (0) | 6.4E-04 | (0) | 2.4E+00 | (47) | 3.2E-04 | (0) | 5.0E-01 | (0) | 2.3E-03 | (0) | 9.6E+01 | (19) | 1.8E-02 | (0) |
| M11 | Women, normal weight | 1.3E-03 | (1) | 6.3E-01 | (0) | 2.9E-02 | (1) | 8.1E-02 | (3) | 1.2E-02 | (2) | 3.7E-04 | (0) | 4.4E-02 | (1) | 1.3E-03 | (0) | 7.4E-01 | (0) | 1.6E-03 | (0) | 8.9E+01 | (18) | 1.8E-02 | (0) |
| M12 | Women, normal weight | 1.2E-04 | (0) | 2.5E-01 | (0) | 1.7E-03 | (0) | 5.6E-03 | (0) | 1.6E-03 | (0) | 1.5E-02 | (4) | 8.4E-04 | (0) | 1.0E-03 | (0) | 1.9E+00 | (0) | 1.1E-03 | (0) | 3.1E-02 | (0) | 9.1E-02 | (0) |
| M13 | Women, normal weight | 3.2E-05 | (0) | 2.8E+00 | (2) | 4.4E-03 | (0) | 4.5E-02 | (2) | 1.6E-02 | (3) | 4.9E-04 | (0) | 4.0E-03 | (0) | 9.4E-04 | (0) | 1.8E+00 | (0) | 2.1E-03 | (0) | 1.2E+00 | (0) | 3.8E-02 | (0) |
| M14 | Women, normal weight | 4.2E-05 | (0) | 3.8E+00 | (3) | 2.7E-02 | (1) | 1.8E-02 | (1) | 3.2E-03 | (1) | 4.2E-04 | (0) | 2.1E-03 | (0) | 4.2E-04 | (0) | 4.5E-01 | (0) | 3.3E-04 | (0) | 1.3E+00 | (0) | 4.0E-03 | (0) |
| M15 | Women, normal weight | 4.1E-05 | (0) | 1.0E+00 | (1) | 4.0E-03 | (0) | 2.1E-02 | (1) | 5.3E-03 | (1) | 2.3E-04 | (0) | 4.0E-04 | (0) | 3.4E-04 | (0) | 2.2E-01 | (0) | 2.5E-04 | (0) | 9.7E-01 | (0) | 1.3E-02 | (0) |
| M16 | Women, normal weight | 6.3E-05 | (0) | 1.1E+01 | (8) | 4.7E-03 | (0) | 4.1E-02 | (1) | 4.4E-03 | (1) | 2.1E-04 | (0) | 3.0E-01 | (6) | 3.7E-04 | (0) | 4.7E-01 | (0) | 9.5E-04 | (0) | 4.3E+01 | (9) | 8.8E-02 | (0) |
|  | Children 3–6 yrs, underweight | 3.1E-04 | (0) | 5.5E+01 | (40) | 2.3E-02 | (0) | 2.0E-01 | (7) | 2.2E-02 | (4) | 1.0E-03 | (0) | 1.5E+00 | (30) | 1.8E-03 | (0) | 2.3E+00 | (0) | 4.7E-03 | (0) | 2.1E+02 | (43) | 4.4E-01 | (1) |
| M17 | Women, normal weight | 7.6E-05 | (0) | 7.4E-02 | (0) | 3.7E-03 | (0) | 2.2E-03 | (0) | 2.0E-03 | (0) | 3.4E-05 | (0) | 2.1E-04 | (0) | 4.9E-04 | (0) | 2.2E-03 | (0) | 3.4E-04 | (0) | 9.2E-02 | (0) | 3.6E-03 | (0) |
| M18 | Women, normal weight | 4.0E-06 | (0) | 3.3E-04 | (0) | 4.5E-05 | (0) | 2.0E-04 | (0) | 2.8E-05 | (0) | 2.2E-05 | (0) | 2.0E-04 | (0) | 1.0E-03 | (0) | 8.4E-03 | (0) | 4.0E-06 | (0) | 2.6E-03 | (0) | 6.8E-05 | (0) |
| M19 | Women, normal weight | 1.8E-05 | (0) | 6.8E-03 | (0) | 2.2E-04 | (0) | 8.8E-04 | (0) | 2.2E-04 | (0) | 1.8E-05 | (0) | 8.8E-04 | (0) | 4.6E-04 | (0) | 6.0E-02 | (0) | 8.2E-05 | (0) | 8.0E-03 | (0) | 3.5E-04 | (0) |
| M20 | Women, normal weight | 6.5E-06 | (0) | 1.8E-03 | (0) | 3.3E-05 | (0) | 3.3E-04 | (0) | 2.9E-05 | (0) | 7.0E-05 | (0) | 3.3E-04 | (0) | 7.2E-05 | (0) | 3.2E-03 | (0) | 1.5E-05 | (0) | 1.7E-03 | (0) | 1.6E-04 | (0) |
| M21 | Women, normal weight | 1.4E-04 | (0) | 2.8E+00 | (2) | 1.2E-02 | (0) | 1.2E-01 | (4) | 2.1E-02 | (4) | 7.2E-04 | (0) | 8.4E-03 | (0) | 6.6E-04 | (0) | 2.4E+00 | (0) | 1.2E-02 | (0) | 1.1E+00 | (0) | 4.3E-02 | (0) |
|  | Children 3–6 yrs, underweight | 6.8E-04 | (0) | 1.4E+01 | (10) | 6.1E-02 | (1) | 6.2E-01 | (22) | 1.1E-01 | (21) | 3.6E-03 | (1) | 4.2E-02 | (1) | 3.3E-03 | (0) | 1.2E+01 | (2) | 6.0E-02 | (0) | 5.6E+00 | (1) | 2.1E-01 | (0) |
| M22 | Women, normal weight | 1.0E-04 | (0) | 2.6E+00 | (2) | 1.3E-02 | (0) | 2.4E-01 | (8) | 1.3E-02 | (3) | 1.4E-03 | (0) | 1.2E-02 | (0) | 1.2E-03 | (0) | 1.0E+01 | (2) | 5.7E-03 | (0) | 9.2E-01 | (0) | 7.1E-02 | (0) |
|  | Children 3–6 yrs, underweight | 5.2E-04 | (0) | 1.3E+01 | (9) | 6.2E-02 | (1) | 1.2E+00 | (42) | 6.3E-02 | (13) | 6.8E-03 | (2) | 5.9E-02 | (1) | 6.0E-03 | (0) | 5.2E+01 | (9) | 2.8E-02 | (0) | 4.6E+00 | (1) | 3.5E-01 | (1) |
| M23 | Women, normal weight | 4.7E-04 | (0) | 2.6E+00 | (2) | 1.0E-02 | (0) | 4.9E-02 | (2) | 4.7E-02 | (9) | 2.0E-03 | (1) | 7.0E-03 | (0) | 7.3E-03 | (0) | 2.0E+00 | (0) | 3.4E-03 | (0) | 2.3E+00 | (0) | 2.0E-01 | (0) |
|  | Children 3–6 yrs, underweight | 2.4E-03 | (1) | 1.3E+01 | (9) | 5.2E-02 | (1) | 2.5E-01 | (9) | 2.3E-01 | (47) | 1.0E-02 | (3) | 3.5E-02 | (1) | 3.6E-02 | (0) | 1.0E+01 | (2) | 1.7E-02 | (0) | 1.1E+01 | (2) | 1.0E+00 | (2) |
| M24 | Women, normal weight | 1.7E-04 | (0) | 3.1E+00 | (2) | 1.2E-02 | (0) | 6.4E-02 | (2) | 6.6E-02 | (13) | 1.7E-03 | (0) | 1.1E-02 | (0) | 1.5E-02 | (0) | 1.6E+00 | (0) | 4.8E-03 | (0) | 1.9E+00 | (0) | 2.5E-01 | (0) |
|  | Children 3–6 yrs, underweight | 8.6E-04 | (0) | 1.5E+01 | (11) | 5.8E-02 | (1) | 3.2E-01 | (11) | 3.3E-01 | (66) | 8.6E-03 | (2) | 5.4E-02 | (1) | 7.5E-02 | (0) | 8.1E+00 | (1) | 2.4E-02 | (0) | 9.2E+00 | (2) | 1.3E+00 | (2) |
| M25 | Women, normal weight | 2.0E-04 | (0) | 2.3E+00 | (2) | 8.7E-03 | (0) | 4.6E-02 | (2) | 9.0E-03 | (2) | 4.3E-04 | (0) | 4.5E-03 | (0) | 2.1E-03 | (0) | 9.5E-01 | (0) | 4.0E-03 | (0) | 8.0E-01 | (0) | 2.2E-02 | (0) |
| M26 | Women, normal weight | 1.0E-04 | (0) | 1.9E+00 | (1) | 7.7E-03 | (0) | 6.9E-02 | (2) | 5.3E-03 | (1) | 6.4E-04 | (0) | 6.0E-03 | (0) | 6.4E-04 | (0) | 1.1E+00 | (0) | 1.6E-02 | (0) | 5.1E-01 | (0) | 1.6E-02 | (0) |
|  | Children 3–6 yrs, underweight | 5.1E-04 | (0) | 9.6E+00 | (7) | 3.9E-02 | (1) | 3.4E-01 | (12) | 2.7E-02 | (5) | 3.2E-03 | (1) | 3.0E-02 | (1) | 3.2E-03 | (0) | 5.6E+00 | (1) | 7.8E-02 | (0) | 2.5E+00 | (1) | 7.7E-02 | (0) |
| M27 | Women, normal weight | 1.6E-03 | (1) | 7.2E+01 | (51) | 3.9E-01 | (8) | 1.6E-01 | (6) | 1.3E-01 | (26) | 3.1E-03 | (1) | 3.1E-02 | (1) | 9.0E-03 | (0) | 3.9E+00 | (1) | 5.7E-03 | (0) | 1.1E+01 | (2) | 4.0E-01 | (1) |
|  | Children 3–6 yrs, underweight | 7.9E-03 | (4) | 3.6E+02 | (254) | 1.9E+00 | (38) | 7.9E-01 | (28) | 6.5E-01 | (130) | 1.5E-02 | (4) | 1.6E-01 | (3) | 4.5E-02 | (0) | 1.9E+01 | (3) | 2.8E-02 | (0) | 5.7E+01 | (11) | 2.0E+00 | (3) |
| M28 | Women, normal weight | 2.3E-04 | (0) | 9.2E+00 | (7) | 6.6E-02 | (1) | 1.7E-02 | (1) | 6.9E-03 | (1) | 4.7E-04 | (0) | 3.0E-03 | (0) | 2.5E-03 | (0) | 2.0E+00 | (0) | 5.1E-04 | (0) | 2.9E+00 | (1) | 6.6E-03 | (0) |
|  | Children 3–6 yrs, underweight | 1.1E-03 | (1) | 4.6E+01 | (33) | 3.3E-01 | (7) | 8.4E-02 | (3) | 3.4E-02 | (7) | 2.3E-03 | (1) | 1.5E-02 | (0) | 1.2E-02 | (0) | 9.8E+00 | (2) | 2.6E-03 | (0) | 1.4E+01 | (3) | 3.3E-02 | (0) |
| M29 | Women, normal weight | 3.9E-04 | (0) | 3.9E+00 | (3) | 1.9E-02 | (0) | 3.9E-02 | (1) | 6.6E-02 | (13) | 3.4E-03 | (1) | 1.4E-02 | (0) | 7.3E-03 | (0) | 2.4E+00 | (0) | 1.6E-03 | (0) | 9.5E-01 | (0) | 1.6E-02 | (0) |
|  | Children 3–6 yrs, underweight | 1.9E-03 | (1) | 1.9E+01 | (14) | 9.3E-02 | (2) | 2.0E-01 | (7) | 3.3E-01 | (66) | 1.7E-02 | (4) | 6.9E-02 | (1) | 3.6E-02 | (0) | 1.2E+01 | (2) | 7.7E-03 | (0) | 4.7E+00 | (1) | 7.8E-02 | (0) |
| M30 | Women, normal weight | 8.9E-04 | (0) | 4.6E+00 | (3) | 4.4E-02 | (1) | 8.1E-02 | (3) | 4.8E-02 | (10) | 1.9E-03 | (0) | 1.8E-03 | (0) | 2.2E-03 | (0) | 2.2E+00 | (0) | 3.0E-03 | (0) | 2.4E+00 | (0) | 9.4E-02 | (0) |
|  | Children 3–6 yrs, underweight | 4.4E-03 | (2) | 2.3E+01 | (16) | 2.2E-01 | (4) | 4.0E-01 | (15) | 2.4E-01 | (48) | 9.5E-03 | (2) | 9.2E-03 | (0) | 1.1E-02 | (0) | 1.1E+01 | (2) | 1.5E-02 | (0) | 1.2E+01 | (2) | 4.7E-01 | (1) |
| M31 | Women, normal weight | 1.1E-03 | (1) | 1.0E+01 | (7) | 6.2E-02 | (1) | 2.1E-01 | (8) | 5.3E-02 | (11) | 1.9E-03 | (0) | 2.8E-02 | (1) | 3.7E-03 | (0) | 4.8E+00 | (1) | 4.8E-03 | (0) | 3.7E+00 | (1) | 1.2E-01 | (0) |
|  | Children 3–6 yrs, underweight | 5.5E-03 | (3) | 5.0E+01 | (36) | 3.1E-01 | (6) | 1.1E+00 | (38) | 2.6E-01 | (52) | 9.6E-03 | (2) | 1.4E-01 | (3) | 1.8E-02 | (0) | 2.4E+01 | (4) | 2.4E-02 | (0) | 1.8E+01 | (4) | 5.9E-01 | (1) |
| M32 | Women, normal weight | 3.9E-03 | (2) | 3.2E+01 | (23) | 1.6E+00 | (32) | 1.1E+00 | (40) | 2.4E-02 | (5) | 3.6E-03 | (1) | 4.8E-01 | (10) | 4.8E-03 | (0) | 4.1E+00 | (1) | 4.4E-03 | (0) | 6.5E+01 | (13) | 6.6E-02 | (0) |
|  | Children 3–6 yrs, underweight | 1.4E-02 | (7) | 1.1E+02 | (79) | 5.6E+00 | (113) | 3.9E+00 | (141) | 8.2E-02 | (16) | 1.3E-02 | (3) | 1.7E+00 | (34) | 1.7E-02 | (0) | 1.4E+01 | (2) | 1.5E-02 | (0) | 2.3E+02 | (45) | 2.3E-01 | (0) |
| M33 | Women, normal weight | 2.1E-04 | (0) | 8.5E-02 | (0) | 5.8E-03 | (0) | 5.5E-03 | (0) | 8.1E-04 | (0) | 5.3E-04 | (0) | 2.0E-03 | (0) | 2.5E-04 | (0) | 1.5E+01 | (3) | 4.2E-03 | (0) | 2.8E-01 | (0) | 3.9E-03 | (0) |
|  | Children 3–6 yrs, underweight | 1.0E-03 | (1) | 4.2E-01 | (0) | 2.9E-02 | (1) | 2.7E-02 | (1) | 4.0E-03 | (1) | 2.6E-03 | (1) | 1.0E-02 | (0) | 1.2E-03 | (0) | 7.5E+01 | (13) | 2.1E-02 | (0) | 1.4E+00 | (0) | 1.9E-02 | (0) |
| M34 | Women, normal weight | 5.3E-04 | (0) | 2.1E-01 | (0) | 3.8E-02 | (1) | 2.2E-02 | (1) | 4.4E-03 | (1) | 1.4E-04 | (0) | 3.5E-02 | (1) | 1.9E-04 | (0) | 4.0E-01 | (0) | 1.1E-03 | (0) | 7.4E-01 | (0) | 6.8E-03 | (0) |
| M35 | Women, normal weight | 1.0E-04 | (0) | 1.7E-03 | (0) | 2.1E-04 | (0) | 3.0E-03 | (0) | 4.8E-04 | (0) | 7.6E-05 | (0) | 6.1E-03 | (0) | 1.6E-04 | (0) | 2.5E-02 | (0) | 9.1E-05 | (0) | 5.6E-03 | (0) | 2.2E-04 | (0) |
| M36 | Women, normal weight | 2.2E-05 | (0) | 1.9E-03 | (0) | 5.5E-04 | (0) | 1.1E-03 | (0) | 1.3E-04 | (0) | 2.2E-05 | (0) | 2.4E-02 | (0) | 2.3E-04 | (0) | 3.1E-02 | (0) | 9.6E-05 | (0) | 5.0E-02 | (0) | 7.5E-04 | (0) |
| M37 | Women, normal weight | 2.4E-05 | (0) | 2.3E-03 | (0) | 4.3E-05 | (0) | 2.3E-03 | (0) | 1.2E-04 | (0) | 8.7E-06 | (0) | 4.3E-04 | (0) | 3.9E-05 | (0) | 7.0E-03 | (0) | 8.7E-06 | (0) | 8.7E-04 | (0) | 3.5E-04 | (0) |
| M38 | Women, normal weight | 1.1E-04 | (0) | 1.2E-02 | (0) | 9.5E-04 | (0) | 1.5E-02 | (1) | 4.7E-04 | (0) | 7.2E-04 | (0) | 2.7E+00 | (54) | 1.3E-03 | (0) | 6.6E-02 | (0) | 8.4E-05 | (0) | 2.8E-02 | (0) | 3.3E-03 | (0) |
|  | Children 3–6 yrs, underweight | 5.7E-04 | (0) | 6.2E-02 | (0) | 4.7E-03 | (0) | 7.4E-02 | (3) | 2.3E-03 | (0) | 3.6E-03 | (1) | 1.4E+01 | (271) | 6.5E-03 | (0) | 3.3E-01 | (0) | 4.2E-04 | (0) | 1.4E-01 | (0) | 1.6E-02 | (0) |

**Table S3.C, part 1 (of 2)** Average Daily Doses (ADDs; µg/kg/day) of all elements with available TDIs, associated with each investigated ***marine fish-oil*** food supplement (MF01-MF09). Values in parentheses = ADDs in % of TDIs.

| **Product** | **Group** | **Ag** | | **Al** | | **As** | | **B** | | **Ba** | | **Be** | | **Br** | | **Cd** | | **Co** | | **Cr** | | **Cu** | | **Fe** | |
| --- | --- | --- | --- | --- | --- | --- | --- | --- | --- | --- | --- | --- | --- | --- | --- | --- | --- | --- | --- | --- | --- | --- | --- | --- | --- |
| MF01 | Women, normal weight | 4.1E-05 | (0) | 8.1E-02 | (0) | 5.4E-04 | (0) | 5.8E-02 | (0) | 4.2E-03 | (0) | 2.1E-05 | (0) | 4.0E-01 | (0) | 8.0E-05 | (0) | 6.9E-04 | (0) | 1.7E-02 | (0) | 2.6E-01 | (0) | 4.0E+00 | (0) |
| MF02 | Women, normal weight | 3.0E-05 | (0) | 1.2E-01 | (0) | 3.8E-04 | (0) | 8.5E-02 | (0) | 6.0E-03 | (0) | 3.0E-05 | (0) | 4.4E-01 | (0) | 3.0E-05 | (0) | 1.2E-04 | (0) | 1.4E-02 | (0) | 1.2E-02 | (0) | 7.4E-02 | (0) |
| MF03 | Women, normal weight | 3.0E-05 | (0) | 4.5E-01 | (0) | 1.0E-03 | (0) | 4.8E-02 | (0) | 6.1E-03 | (0) | 3.0E-05 | (0) | 4.9E-01 | (0) | 3.0E-05 | (0) | 3.0E-05 | (0) | 5.7E-03 | (0) | 3.0E-03 | (0) | 3.6E-02 | (0) |
| MF04 | Women, normal weight | 8.8E-05 | (0) | 2.9E+00 | (2) | 2.1E-03 | (1) | 1.1E-01 | (0) | 5.9E-02 | (0) | 2.9E-04 | (0) | 1.2E-01 | (0) | 3.6E-03 | (1) | 2.5E-02 | (2) | 7.3E-02 | (0) | 1.7E+01 | (12) | 6.0E+02 | (75) |
| MF05 | Women, normal weight | 1.7E-04 | (0) | 6.2E-01 | (0) | 1.5E-04 | (0) | 3.9E-02 | (0) | 3.8E-02 | (0) | 7.7E-05 | (0) | 3.9E-02 | (0) | 1.8E-04 | (0) | 7.7E-05 | (0) | 5.0E-03 | (0) | 8.9E-03 | (0) | 1.5E-01 | (0) |
|  | Children 3–6 yrs, underweight | 8.6E-04 | (0) | 3.1E+00 | (2) | 7.7E-04 | (0) | 1.9E-01 | (0) | 1.9E-01 | (0) | 3.9E-04 | (0) | 1.9E-01 | (0) | 9.2E-04 | (0) | 3.9E-04 | (0) | 2.5E-02 | (0) | 4.4E-02 | (0) | 7.6E-01 | (0) |
| MF06 | Women, normal weight | 6.1E-05 | (0) | 7.4E-02 | (0) | 6.2E-04 | (0) | 7.3E-02 | (0) | 1.2E-02 | (0) | 6.1E-05 | (0) | 7.2E-02 | (0) | 6.1E-05 | (0) | 2.3E-04 | (0) | 7.1E-03 | (0) | 4.7E-03 | (0) | 5.3E-02 | (0) |
| MF07 | Women, normal weight | 3.7E-05 | (0) | 4.5E-02 | (0) | 3.7E-04 | (0) | 1.8E-02 | (0) | 7.4E-03 | (0) | 3.7E-05 | (0) | 1.7E-01 | (0) | 3.7E-05 | (0) | 1.1E-04 | (0) | 7.5E-04 | (0) | 1.8E-04 | (0) | 7.4E-03 | (0) |
|  | Children 3–6 yrs, underweight | 1.8E-04 | (0) | 2.2E-01 | (0) | 1.8E-03 | (1) | 9.2E-02 | (0) | 3.7E-02 | (0) | 1.8E-04 | (0) | 8.6E-01 | (0) | 1.8E-04 | (0) | 5.2E-04 | (0) | 3.7E-03 | (0) | 9.2E-04 | (0) | 3.7E-02 | (0) |
| MF08 | Women, normal weight | 3.7E-05 | (0) | 3.1E-02 | (0) | 3.3E-04 | (0) | 1.8E-02 | (0) | 7.4E-03 | (0) | 3.7E-05 | (0) | 1.6E-01 | (0) | 3.7E-05 | (0) | 1.4E-04 | (0) | 3.7E-03 | (0) | 1.8E-04 | (0) | 3.6E-02 | (0) |
|  | Children 3–6 yrs, underweight | 1.8E-04 | (0) | 1.5E-01 | (0) | 1.6E-03 | (1) | 9.2E-02 | (0) | 3.7E-02 | (0) | 1.8E-04 | (0) | 8.1E-01 | (0) | 1.8E-04 | (0) | 7.1E-04 | (0) | 1.8E-02 | (0) | 9.2E-04 | (0) | 1.8E-01 | (0) |
| MF09 | Women, normal weight | 2.5E-04 | (0) | 7.6E-01 | (1) | 9.0E-04 | (0) | 3.1E-02 | (0) | 1.3E-02 | (0) | 6.3E-05 | (0) | 1.2E-01 | (0) | 6.3E-05 | (0) | 1.4E-04 | (0) | 6.9E-03 | (0) | 1.8E-01 | (0) | 2.8E-01 | (0) |
|  | Children 3–6 yrs, underweight | 1.3E-03 | (0) | 3.8E+00 | (3) | 4.5E-03 | (1) | 1.6E-01 | (0) | 6.3E-02 | (0) | 3.1E-04 | (0) | 5.9E-01 | (0) | 3.1E-04 | (0) | 6.8E-04 | (0) | 3.4E-02 | (0) | 8.9E-01 | (1) | 1.4E+00 | (0) |

**Table S3.C, part 2 (of 2)**

| **Product** | **Group** | **Hg** | | **Mn** | | **Mo** | | **Ni** | | **Pb** | | **Sb** | | **Se** | | **Sn** | | **Sr** | | **U** | | **Zn** | | **REE** | |
| --- | --- | --- | --- | --- | --- | --- | --- | --- | --- | --- | --- | --- | --- | --- | --- | --- | --- | --- | --- | --- | --- | --- | --- | --- | --- |
| MF01 | Women, normal weight | 1.2E-04 | (0) | 5.0E-01 | (0) | 9.4E-03 | (0) | 1.0E-03 | (0) | 3.0E-04 | (0) | 6.4E-05 | (0) | 1.9E-02 | (0) | 2.1E-05 | (0) | 2.4E-03 | (0) | 9.2E-05 | (0) | 2.6E+00 | (1) | 9.8E-04 | (0) |
| MF02 | Women, normal weight | 3.0E-05 | (0) | 4.9E-02 | (0) | 5.8E-04 | (0) | 1.5E-03 | (0) | 7.5E-04 | (0) | 8.4E-05 | (0) | 3.0E-03 | (0) | 3.0E-04 | (0) | 3.7E-03 | (0) | 7.1E-05 | (0) | 1.7E-02 | (0) | 1.0E-03 | (0) |
| MF03 | Women, normal weight | 6.6E-05 | (0) | 1.8E-03 | (0) | 1.5E-04 | (0) | 1.5E-03 | (0) | 2.0E-04 | (0) | 6.9E-05 | (0) | 4.4E-03 | (0) | 3.0E-05 | (0) | 6.0E-03 | (0) | 1.9E-04 | (0) | 7.5E-03 | (0) | 5.2E-04 | (0) |
| MF04 | Women, normal weight | 4.8E-05 | (0) | 1.3E+00 | (1) | 4.3E-03 | (0) | 3.8E-02 | (1) | 7.9E-03 | (2) | 3.2E-04 | (0) | 9.2E-01 | (18) | 1.4E-03 | (0) | 7.3E-01 | (0) | 2.8E-02 | (0) | 1.4E+02 | (29) | 3.2E-02 | (0) |
| MF05 | Women, normal weight | 7.7E-05 | (0) | 6.8E-03 | (0) | 1.6E-03 | (0) | 3.9E-03 | (0) | 2.1E-04 | (0) | 4.7E-04 | (0) | 3.9E-03 | (0) | 3.1E-04 | (0) | 8.9E-02 | (0) | 8.4E-04 | (0) | 3.7E-02 | (0) | 1.5E-03 | (0) |
|  | Children 3–6 yrs, underweight | 3.9E-04 | (0) | 3.4E-02 | (0) | 8.0E-03 | (0) | 1.9E-02 | (1) | 1.0E-03 | (0) | 2.3E-03 | (1) | 1.9E-02 | (0) | 1.5E-03 | (0) | 4.4E-01 | (0) | 4.2E-03 | (0) | 1.8E-01 | (0) | 7.5E-03 | (0) |
| MF06 | Women, normal weight | 6.1E-05 | (0) | 1.2E-03 | (0) | 3.1E-04 | (0) | 3.1E-03 | (0) | 2.0E-04 | (0) | 2.4E-04 | (0) | 8.6E-03 | (0) | 2.2E-04 | (0) | 1.2E-03 | (0) | 1.8E-04 | (0) | 6.1E-03 | (0) | 9.8E-04 | (0) |
| MF07 | Women, normal weight | 3.7E-05 | (0) | 7.4E-04 | (0) | 1.8E-04 | (0) | 1.8E-03 | (0) | 3.7E-05 | (0) | 3.7E-05 | (0) | 1.8E-03 | (0) | 3.7E-05 | (0) | 7.4E-04 | (0) | 3.7E-05 | (0) | 3.7E-03 | (0) | 6.9E-04 | (0) |
|  | Children 3–6 yrs, underweight | 1.8E-04 | (0) | 3.7E-03 | (0) | 9.2E-04 | (0) | 9.2E-03 | (0) | 1.8E-04 | (0) | 1.8E-04 | (0) | 9.2E-03 | (0) | 1.8E-04 | (0) | 3.7E-03 | (0) | 1.8E-04 | (0) | 1.8E-02 | (0) | 3.4E-03 | (0) |
| MF08 | Women, normal weight | 3.7E-05 | (0) | 1.9E-03 | (0) | 1.8E-04 | (0) | 1.8E-03 | (0) | 3.7E-05 | (0) | 9.8E-05 | (0) | 1.8E-03 | (0) | 3.7E-05 | (0) | 7.4E-04 | (0) | 3.7E-05 | (0) | 3.7E-03 | (0) | 5.9E-04 | (0) |
|  | Children 3–6 yrs, underweight | 1.8E-04 | (0) | 9.3E-03 | (0) | 9.2E-04 | (0) | 9.2E-03 | (0) | 1.8E-04 | (0) | 4.9E-04 | (0) | 9.2E-03 | (0) | 1.8E-04 | (0) | 3.7E-03 | (0) | 1.8E-04 | (0) | 1.8E-02 | (0) | 2.9E-03 | (0) |
| MF09 | Women, normal weight | 6.3E-05 | (0) | 1.1E-02 | (0) | 3.3E-03 | (0) | 3.1E-03 | (0) | 1.4E-02 | (3) | 1.5E-03 | (0) | 3.1E-03 | (0) | 3.0E-03 | (0) | 9.3E-02 | (0) | 1.7E-03 | (0) | 2.7E-01 | (0) | 2.9E-03 | (0) |
|  | Children 3–6 yrs, underweight | 3.1E-04 | (0) | 5.3E-02 | (0) | 1.6E-02 | (0) | 1.6E-02 | (1) | 7.1E-02 | (14) | 7.7E-03 | (2) | 1.6E-02 | (0) | 1.5E-02 | (0) | 4.6E-01 | (0) | 8.4E-03 | (0) | 1.3E+00 | (0) | 1.5E-02 | (0) |

**Table S3.D, part 1 (of 2)** Average Daily Doses (ADDs; µg/kg/day) of all elements with available TDIs, associated with each investigated ***terrestrial plant-based* food** supplement (TPB01-TPB62). Values in parentheses = ADDs in % of TDIs.

| **Product** | **Group** | **Ag** | | **Al** | | **As** | | **B** | | **Ba** | | **Be** | | **Br** | | **Cd** | | **Co** | | **Cr** | | **Cu** | | **Fe** | |
| --- | --- | --- | --- | --- | --- | --- | --- | --- | --- | --- | --- | --- | --- | --- | --- | --- | --- | --- | --- | --- | --- | --- | --- | --- | --- |
| TPB01 | Women, normal weight | 4.5E-05 | (0) | 2.1E-01 | (0) | 6.7E-04 | (0) | 1.2E-01 | (0) | 6.0E-02 | (0) | 4.5E-05 | (0) | 2.2E-02 | (0) | 1.5E-03 | (0) | 1.7E-03 | (0) | 1.9E-02 | (0) | 4.8E-02 | (0) | 7.0E-01 | (0) |
| TPB02 | Women, normal weight | 1.4E-05 | (0) | 3.4E-01 | (0) | 1.0E-03 | (0) | 5.5E-01 | (0) | 1.5E-01 | (0) | 5.2E-05 | (0) | 5.4E-02 | (0) | 2.3E-04 | (0) | 2.5E-03 | (0) | 2.0E-03 | (0) | 4.0E-01 | (0) | 3.8E-01 | (0) |
| TPB03 | Women, normal weight | 1.7E-04 | (0) | 1.0E+01 | (7) | 4.9E-03 | (2) | 5.4E-01 | (0) | 1.4E-01 | (0) | 1.1E-03 | (0) | 2.3E-01 | (0) | 1.5E-02 | (4) | 4.0E-03 | (0) | 7.8E-02 | (0) | 9.3E-02 | (0) | 1.0E+01 | (1) |
| TPB04 | Women, normal weight | 6.4E-05 | (0) | 1.9E-01 | (0) | 2.5E-04 | (0) | 4.9E-02 | (0) | 8.2E-03 | (0) | 2.6E-05 | (0) | 4.0E-02 | (0) | 1.6E-03 | (0) | 5.9E-03 | (0) | 7.9E-01 | (0) | 1.8E-02 | (0) | 2.6E-01 | (0) |
| TPB05 | Women, normal weight | 6.5E-05 | (0) | 6.5E-01 | (0) | 3.5E-03 | (1) | 2.0E-01 | (0) | 8.7E-02 | (0) | 9.9E-04 | (0) | 1.6E-01 | (0) | 6.2E-03 | (2) | 1.3E-02 | (1) | 5.7E-02 | (0) | 2.1E-01 | (0) | 1.1E+00 | (0) |
| TPB06 | Women, normal weight | 5.3E-06 | (0) | 5.5E-01 | (0) | 2.5E-03 | (1) | 1.3E-01 | (0) | 3.3E-02 | (0) | 2.1E-04 | (0) | 9.7E-03 | (0) | 1.7E-04 | (0) | 1.7E-03 | (0) | 5.3E-03 | (0) | 4.7E+00 | (3) | 7.4E-01 | (0) |
| TPB07 | Women, normal weight | 7.1E-05 | (0) | 2.6E+01 | (18) | 9.1E-03 | (3) | 4.5E-01 | (0) | 1.8E-01 | (0) | 8.5E-04 | (0) | 4.8E-02 | (0) | 6.6E-04 | (0) | 1.6E-02 | (1) | 9.8E-02 | (0) | 4.4E-01 | (0) | 2.2E+00 | (0) |
| TPB08 | Women, normal weight | 9.2E-05 | (0) | 1.1E+01 | (8) | 2.0E-03 | (1) | 1.1E-01 | (0) | 2.0E-01 | (0) | 2.5E-04 | (0) | 2.1E-02 | (0) | 6.1E-04 | (0) | 4.9E-03 | (0) | 2.1E-02 | (0) | 3.3E-01 | (0) | 7.1E+00 | (1) |
| TPB09 | Women, normal weight | 8.6E-06 | (0) | 1.8E+00 | (1) | 6.9E-04 | (0) | 3.5E-01 | (0) | 1.1E-02 | (0) | 3.4E-05 | (0) | 6.8E-02 | (0) | 2.2E-04 | (0) | 4.2E-02 | (3) | 8.3E-01 | (0) | 1.8E+01 | (13) | 2.8E+02 | (35) |
| TPB10 | Women, normal weight | 1.2E-05 | (0) | 1.8E-02 | (0) | 2.8E-04 | (0) | 6.7E-02 | (0) | 1.2E-02 | (0) | 7.2E-06 | (0) | 3.1E-01 | (0) | 9.2E-05 | (0) | 9.8E-05 | (0) | 1.5E-03 | (0) | 1.6E-02 | (0) | 1.4E-01 | (0) |
| TPB11 | Women, normal weight | 3.3E-06 | (0) | 1.5E-02 | (0) | 1.2E-03 | (0) | 1.3E-01 | (0) | 8.5E-03 | (0) | 3.9E-05 | (0) | 3.7E-03 | (0) | 7.9E-05 | (0) | 4.9E-04 | (0) | 1.2E-03 | (0) | 3.0E-02 | (0) | 8.9E-02 | (0) |
| TPB12 | Women, normal weight | 3.1E-04 | (0) | 1.0E-01 | (0) | 4.6E-04 | (0) | 1.7E+00 | (1) | 3.9E-01 | (0) | 3.8E-05 | (0) | 4.2E-01 | (0) | 8.6E-04 | (0) | 1.9E-03 | (0) | 3.8E-03 | (0) | 3.1E-01 | (0) | 1.2E+00 | (0) |
| TPB13 | Women, normal weight | 6.2E-06 | (0) | 3.3E+00 | (2) | 1.5E-03 | (1) | 1.6E-02 | (0) | 7.9E-03 | (0) | 6.0E-05 | (0) | 3.1E-03 | (0) | 6.2E-06 | (0) | 2.0E-04 | (0) | 2.3E-02 | (0) | 4.3E-03 | (0) | 6.8E-01 | (0) |
| TPB14 | Women, normal weight | 1.3E-05 | (0) | 2.1E-01 | (0) | 1.4E-03 | (0) | 6.7E-01 | (0) | 8.0E-02 | (0) | 7.3E-05 | (0) | 1.8E-01 | (0) | 4.7E-05 | (0) | 1.5E-03 | (0) | 1.7E-02 | (0) | 1.1E-01 | (0) | 6.6E-01 | (0) |
| TPB15 | Women, normal weight | 2.0E-05 | (0) | 1.3E-01 | (0) | 9.2E-05 | (0) | 9.2E-01 | (0) | 1.4E+00 | (1) | 2.0E-05 | (0) | 3.2E-01 | (0) | 8.1E-05 | (0) | 8.2E-04 | (0) | 2.7E-03 | (0) | 1.0E-01 | (0) | 2.8E-01 | (0) |
| TPB16 | Women, normal weight | 2.3E-05 | (0) | 6.5E-01 | (0) | 1.2E-03 | (0) | 4.3E-02 | (0) | 3.6E-02 | (0) | 3.9E-05 | (0) | 1.3E-01 | (0) | 4.8E-04 | (0) | 1.4E-03 | (0) | 3.3E-03 | (0) | 4.4E-02 | (0) | 7.1E-01 | (0) |
| TPB17 | Women, normal weight | 1.9E-05 | (0) | 2.4E+00 | (2) | 3.2E-04 | (0) | 2.8E-02 | (0) | 6.7E-02 | (0) | 7.0E-05 | (0) | 7.2E-02 | (0) | 1.3E-04 | (0) | 6.3E-04 | (0) | 2.6E-03 | (0) | 1.5E-02 | (0) | 2.8E-01 | (0) |
| TPB18 | Women, normal weight | 8.4E-05 | (0) | 1.2E+00 | (1) | 1.3E-03 | (0) | 8.0E-02 | (0) | 3.7E-02 | (0) | 2.3E-05 | (0) | 3.1E-01 | (0) | 2.0E-04 | (0) | 8.1E-04 | (0) | 2.4E-02 | (0) | 2.4E-02 | (0) | 5.2E-01 | (0) |
| TPB19 | Women, normal weight | 6.2E-05 | (0) | 4.9E+00 | (3) | 6.8E-04 | (0) | 1.0E-01 | (0) | 1.2E-01 | (0) | 9.6E-05 | (0) | 8.5E-01 | (0) | 2.9E-04 | (0) | 1.0E-02 | (1) | 1.9E-02 | (0) | 7.6E-01 | (1) | 1.4E+01 | (2) |
| TPB20 | Women, normal weight | 7.7E-06 | (0) | 2.1E-02 | (0) | 2.8E-04 | (0) | 2.0E-01 | (0) | 3.8E-02 | (0) | 7.7E-06 | (0) | 5.3E-02 | (0) | 7.4E-04 | (0) | 2.4E-03 | (0) | 4.9E-04 | (0) | 2.9E-01 | (0) | 1.4E+00 | (0) |
|  | Children 3–6 yrs, underweight | 3.8E-05 | (0) | 1.0E-01 | (0) | 1.4E-03 | (0) | 9.8E-01 | (0) | 1.9E-01 | (0) | 3.8E-05 | (0) | 2.6E-01 | (0) | 3.7E-03 | (1) | 1.2E-02 | (1) | 2.5E-03 | (0) | 1.4E+00 | (1) | 7.0E+00 | (1) |
| TPB21 | Women, normal weight | 4.9E-04 | (0) | 3.2E+00 | (2) | 4.6E-04 | (0) | 6.4E-02 | (0) | 2.3E-01 | (0) | 1.0E-04 | (0) | 3.4E-01 | (0) | 4.2E-04 | (0) | 4.3E-03 | (0) | 1.2E-02 | (0) | 7.0E-02 | (0) | 3.6E+00 | (0) |
|  | Children 3–6 yrs, underweight | 2.4E-03 | (0) | 1.6E+01 | (11) | 2.3E-03 | (1) | 3.2E-01 | (0) | 1.2E+00 | (1) | 5.2E-04 | (0) | 1.7E+00 | (0) | 2.1E-03 | (1) | 2.1E-02 | (2) | 6.2E-02 | (0) | 3.5E-01 | (0) | 1.8E+01 | (2) |
| TPB22 | Women, normal weight | 7.4E-06 | (0) | 3.6E+00 | (3) | 5.7E-04 | (0) | 7.2E-02 | (0) | 9.5E-01 | (0) | 2.1E-04 | (0) | 1.2E-01 | (0) | 1.8E-03 | (0) | 3.3E-03 | (0) | 4.8E-03 | (0) | 6.2E-02 | (0) | 3.2E+00 | (0) |
|  | Children 3–6 yrs, underweight | 3.7E-05 | (0) | 1.8E+01 | (13) | 2.8E-03 | (1) | 3.6E-01 | (0) | 4.7E+00 | (2) | 1.0E-03 | (0) | 5.9E-01 | (0) | 8.9E-03 | (2) | 1.7E-02 | (1) | 2.4E-02 | (0) | 3.1E-01 | (0) | 1.6E+01 | (2) |
| TPB23 | Women, normal weight | 7.3E-04 | (0) | 2.5E+01 | (17) | 5.7E-02 | (19) | 3.9E+00 | (2) | 2.7E+00 | (1) | 1.7E-03 | (0) | 4.5E+00 | (0) | 1.3E-01 | (35) | 1.3E-02 | (1) | 7.7E-02 | (0) | 7.7E-01 | (1) | 2.7E+01 | (3) |
|  | Children 3–6 yrs, underweight | 3.6E-03 | (0) | 1.2E+02 | (87) | 2.8E-01 | (95) | 1.9E+01 | (10) | 1.3E+01 | (7) | 8.3E-03 | (0) | 2.2E+01 | (2) | 6.2E-01 | (175) | 6.3E-02 | (5) | 3.8E-01 | (0) | 3.8E+00 | (3) | 1.3E+02 | (17) |
| TPB24 | Women, normal weight | 3.6E-04 | (0) | 3.3E+01 | (23) | 6.4E-03 | (2) | 6.1E-01 | (0) | 3.1E-01 | (0) | 1.1E-03 | (0) | 6.2E-01 | (0) | 3.7E-03 | (1) | 5.6E-02 | (4) | 1.1E-01 | (0) | 3.0E-01 | (0) | 5.6E+01 | (7) |
|  | Children 3–6 yrs, underweight | 1.8E-03 | (0) | 1.7E+02 | (116) | 3.2E-02 | (11) | 3.1E+00 | (2) | 1.5E+00 | (1) | 5.7E-03 | (0) | 3.1E+00 | (0) | 1.8E-02 | (5) | 2.8E-01 | (20) | 5.6E-01 | (0) | 1.5E+00 | (1) | 2.8E+02 | (35) |
| TPB25 | Women, normal weight | 1.5E-04 | (0) | 2.8E+00 | (2) | 6.8E-04 | (0) | 2.2E+00 | (1) | 1.0E+00 | (1) | 2.6E-04 | (0) | 3.4E-01 | (0) | 4.0E-04 | (0) | 3.9E-03 | (0) | 9.3E-03 | (0) | 5.3E-01 | (0) | 5.0E+00 | (1) |
|  | Children 3–6 yrs, underweight | 7.6E-04 | (0) | 1.4E+01 | (10) | 3.4E-03 | (1) | 1.1E+01 | (5) | 5.1E+00 | (3) | 1.3E-03 | (0) | 1.7E+00 | (0) | 2.0E-03 | (1) | 2.0E-02 | (1) | 4.6E-02 | (0) | 2.7E+00 | (2) | 2.5E+01 | (3) |
| TPB26 | Women, normal weight | 2.1E-04 | (0) | 1.8E+00 | (1) | 7.5E-03 | (2) | 4.8E+00 | (2) | 1.1E+01 | (5) | 2.1E-04 | (0) | 1.1E+00 | (0) | 3.5E-03 | (1) | 6.0E-02 | (4) | 7.2E-03 | (0) | 6.4E+00 | (5) | 2.6E+01 | (3) |
|  | Children 3–6 yrs, underweight | 1.0E-03 | (0) | 8.8E+00 | (6) | 3.7E-02 | (12) | 2.4E+01 | (12) | 5.4E+01 | (27) | 1.0E-03 | (0) | 5.6E+00 | (1) | 1.7E-02 | (5) | 3.0E-01 | (21) | 3.6E-02 | (0) | 3.2E+01 | (23) | 1.3E+02 | (16) |
| TPB27 | Women, normal weight | 3.7E-05 | (0) | 2.2E+00 | (2) | 2.6E-04 | (0) | 1.5E-01 | (0) | 3.9E-02 | (0) | 3.7E-05 | (0) | 2.3E-01 | (0) | 9.6E-05 | (0) | 4.0E-04 | (0) | 5.1E-03 | (0) | 3.8E-02 | (0) | 4.4E-01 | (0) |
|  | Children 3–6 yrs, underweight | **1.8E-04** | **(0)** | **1.1E+01** | **(8)** | **1.3E-03** | **(0)** | **7.5E-01** | **(0)** | **1.9E-01** | **(0)** | **1.8E-04** | **(0)** | **1.1E+00** | **(0)** | **4.8E-04** | **(0)** | **2.0E-03** | **(0)** | **2.5E-02** | **(0)** | **1.9E-01** | **(0)** | **2.2E+00** | **(0)** |
| TPB28 | Women, normal weight | 4.1E-04 | (0) | 6.3E+00 | (4) | 5.5E-03 | (2) | 2.9E+00 | (1) | 2.1E-01 | (0) | 1.3E-04 | (0) | 5.0E-01 | (0) | 1.4E-02 | (4) | 1.6E-02 | (1) | 1.4E-02 | (0) | 2.0E+00 | (1) | 1.6E+01 | (2) |
|  | Children 3–6 yrs, underweight | 2.0E-03 | (0) | 3.1E+01 | (22) | 2.7E-02 | (9) | 1.4E+01 | (7) | 1.1E+00 | (1) | 6.3E-04 | (0) | 2.5E+00 | (0) | 6.9E-02 | (19) | 8.0E-02 | (6) | 6.9E-02 | (0) | 1.0E+01 | (7) | 7.8E+01 | (10) |
| TPB29 | Women, normal weight | 1.3E-03 | (0) | 9.7E-01 | (1) | 9.9E-04 | (0) | 5.3E+00 | (3) | 3.8E+00 | (2) | 1.9E-04 | (0) | 1.6E+00 | (0) | 5.7E-03 | (2) | 3.4E-02 | (2) | 1.8E-02 | (0) | 2.1E+00 | (1) | 2.6E+01 | (3) |
|  | Children 3–6 yrs, underweight | 6.6E-03 | (0) | 4.9E+00 | (3) | 4.9E-03 | (2) | 2.6E+01 | (13) | 1.9E+01 | (9) | 9.5E-04 | (0) | 8.2E+00 | (1) | 2.8E-02 | (8) | 1.7E-01 | (12) | 8.7E-02 | (0) | 1.0E+01 | (7) | 1.3E+02 | (16) |
| TPB30 | Women, normal weight | 5.8E-05 | (0) | 4.1E-02 | (0) | 1.2E-04 | (0) | 1.2E-01 | (0) | 7.9E-02 | (0) | 5.8E-05 | (0) | 1.5E-01 | (0) | 5.8E-05 | (0) | 5.8E-05 | (0) | 3.1E-03 | (0) | 9.0E-03 | (0) | 8.5E-02 | (0) |
| TPB31 | Women, normal weight | 2.1E-04 | (0) | 3.4E-01 | (0) | 5.1E-04 | (0) | 1.9E-01 | (0) | 1.1E-01 | (0) | 5.6E-05 | (0) | 1.3E-01 | (0) | 2.6E-03 | (1) | 7.4E-04 | (0) | 1.5E-02 | (0) | 4.8E-02 | (0) | 9.0E-01 | (0) |

**Table S3.D, part 1 (of 2)**, *continued*

| **Product** | **Group** | **Ag** | | **Al** | | **As** | | **B** | | **Ba** | | **Be** | | **Br** | | **Cd** | | **Co** | | **Cr** | | **Cu** | | **Fe** | |
| --- | --- | --- | --- | --- | --- | --- | --- | --- | --- | --- | --- | --- | --- | --- | --- | --- | --- | --- | --- | --- | --- | --- | --- | --- | --- |
| TPB32 | Women, normal weight | 3.8E-05 | (0) | 1.2E-01 | (0) | 2.9E-04 | (0) | 1.1E+00 | (1) | 1.4E-02 | (0) | 5.9E-04 | (0) | 1.0E-01 | (0) | 7.7E-04 | (0) | 5.1E-04 | (0) | 7.7E-03 | (0) | 1.8E-03 | (0) | 1.6E-01 | (0) |
| TPB33 | Women, normal weight | 6.4E-05 | (0) | 1.7E-01 | (0) | 2.5E-04 | (0) | 3.7E-02 | (0) | 1.7E-02 | (0) | 1.2E-04 | (0) | 1.7E-02 | (0) | 1.3E-04 | (0) | 4.1E-04 | (0) | 3.5E-03 | (0) | 4.6E-03 | (0) | 2.3E-01 | (0) |
| TPB34 | Women, normal weight | 4.5E-05 | (0) | 1.4E-01 | (0) | 2.5E-04 | (0) | 3.0E-02 | (0) | 2.8E-02 | (0) | 2.4E-04 | (0) | 3.5E-02 | (0) | 9.8E-04 | (0) | 6.6E-04 | (0) | 7.8E-03 | (0) | 2.9E-03 | (0) | 2.0E-01 | (0) |
| TPB35 | Women, normal weight | 6.9E-05 | (0) | 1.6E-01 | (0) | 8.2E-04 | (0) | 7.5E-02 | (0) | 2.9E-02 | (0) | 1.3E-04 | (0) | 6.9E-02 | (0) | 2.3E-04 | (0) | 8.4E-04 | (0) | 1.0E-02 | (0) | 7.2E-03 | (0) | 3.6E-01 | (0) |
| TPB36 | Women, normal weight | 7.0E-04 | (0) | 7.0E+00 | (5) | 7.8E-03 | (3) | 3.5E-01 | (0) | 2.7E-01 | (0) | 3.7E-04 | (0) | 1.8E-01 | (0) | 1.5E-02 | (4) | 9.2E-03 | (1) | 8.1E-02 | (0) | 1.9E-01 | (0) | 1.5E+01 | (2) |
| TPB37 | Women, normal weight | 5.8E-04 | (0) | 3.9E+00 | (3) | 6.3E-03 | (2) | 2.2E-01 | (0) | 8.4E-02 | (0) | 1.9E-04 | (0) | 2.2E-01 | (0) | 1.9E-03 | (1) | 1.1E-02 | (1) | 2.4E-02 | (0) | 1.7E-01 | (0) | 2.0E+01 | (2) |
| TPB38 | Women, normal weight | 6.3E-05 | (0) | 1.1E-01 | (0) | 7.9E-04 | (0) | 4.3E-02 | (0) | 1.8E-02 | (0) | 1.1E-04 | (0) | 4.4E-01 | (0) | 3.8E-04 | (0) | 7.9E-04 | (0) | 2.2E-03 | (0) | 9.7E-02 | (0) | 1.0E+00 | (0) |
| TPB39 | Women, normal weight | 3.1E-05 | (0) | 5.0E-01 | (0) | 3.7E-04 | (0) | 3.2E-01 | (0) | 1.1E-02 | (0) | 2.7E-05 | (0) | 9.1E-02 | (0) | 6.5E-04 | (0) | 3.2E-02 | (2) | 4.0E-02 | (0) | 1.6E+01 | (12) | 2.1E+02 | (27) |
| TPB40 | Women, normal weight | 2.5E-04 | (0) | 3.5E+01 | (24) | 1.2E-03 | (0) | 2.0E-01 | (0) | 1.1E-01 | (0) | 1.0E-03 | (0) | 3.0E-01 | (0) | 4.4E-04 | (0) | 1.3E-02 | (1) | 7.2E-03 | (0) | 9.0E-02 | (0) | 4.3E-01 | (0) |
| TPB41 | Women, normal weight | 9.0E-05 | (0) | 1.4E+00 | (1) | 7.6E-03 | (3) | 9.4E-02 | (0) | 2.1E-01 | (0) | 2.3E-04 | (0) | 8.3E-02 | (0) | 1.5E-03 | (0) | 5.8E-03 | (0) | 1.7E-02 | (0) | 1.2E+01 | (8) | 2.3E+00 | (0) |
| TPB42 | Women, normal weight | 1.5E-04 | (0) | 1.1E-01 | (0) | 1.3E-04 | (0) | 2.4E-02 | (0) | 1.4E-02 | (0) | 1.5E-05 | (0) | 7.5E-03 | (0) | 1.5E-05 | (0) | 1.4E-04 | (0) | 1.7E-03 | (0) | 8.6E-03 | (0) | 6.9E-02 | (0) |
|  | Children 3–6 yrs, underweight | 7.3E-04 | (0) | 5.7E-01 | (0) | 6.6E-04 | (0) | 1.2E-01 | (0) | 6.8E-02 | (0) | 7.4E-05 | (0) | 3.7E-02 | (0) | 7.4E-05 | (0) | 6.7E-04 | (0) | 8.5E-03 | (0) | 4.3E-02 | (0) | 3.4E-01 | (0) |
| TPB43 | Women, normal weight | 1.2E-04 | (0) | 1.6E-01 | (0) | 3.8E-04 | (0) | 1.6E-01 | (0) | 1.7E-02 | (0) | 1.6E-05 | (0) | 2.3E+00 | (0) | 8.8E-05 | (0) | 1.5E-03 | (0) | 6.5E-03 | (0) | 9.2E-02 | (0) | 8.1E-01 | (0) |
| TPB44 | Women, normal weight | 9.4E-05 | (0) | 3.3E-01 | (0) | 4.0E-03 | (1) | 6.2E-01 | (0) | 4.5E-02 | (0) | 4.5E-05 | (0) | 6.1E+00 | (1) | 3.3E-04 | (0) | 2.2E-02 | (2) | 2.4E-02 | (0) | 5.8E-01 | (0) | 8.5E-01 | (0) |
| TPB45 | Women, normal weight | 7.4E-05 | (0) | 1.0E+01 | (7) | 1.2E-03 | (0) | 1.7E-01 | (0) | 4.0E-02 | (0) | 2.2E-04 | (0) | 9.9E-02 | (0) | 1.6E-04 | (0) | 7.6E-03 | (1) | 6.1E-03 | (0) | 4.3E-02 | (0) | 3.0E-01 | (0) |
| TPB46 | Women, normal weight | 1.3E-05 | (0) | 2.8E-01 | (0) | 3.2E-04 | (0) | 2.0E-02 | (0) | 1.2E-02 | (0) | 2.0E-05 | (0) | 9.6E-03 | (0) | 3.4E-05 | (0) | 2.2E-04 | (0) | 1.6E-03 | (0) | 7.1E-03 | (0) | 3.5E-01 | (0) |
| TPB47 | Women, normal weight | 5.8E-05 | (0) | 2.7E-02 | (0) | 4.1E-04 | (0) | 1.9E-01 | (0) | 3.2E-02 | (0) | 2.6E-05 | (0) | 1.5E-02 | (0) | 1.8E-05 | (0) | 4.6E-04 | (0) | 2.1E-03 | (0) | 6.5E-03 | (0) | 8.5E-02 | (0) |
| TPB48 | Women, normal weight | 3.0E-04 | (0) | 7.1E-01 | (0) | 1.7E-03 | (1) | 2.6E-01 | (0) | 3.4E-01 | (0) | 6.1E-05 | (0) | 1.1E-01 | (0) | 3.9E-03 | (1) | 1.5E-03 | (0) | 3.6E-02 | (0) | 4.6E-02 | (0) | 1.1E+00 | (0) |
| TPB49 | Women, normal weight | 9.5E-05 | (0) | 6.3E-01 | (0) | 3.5E-04 | (0) | 8.2E-02 | (0) | 5.8E-02 | (0) | 3.3E-05 | (0) | 1.4E-01 | (0) | 1.5E-04 | (0) | 9.6E-04 | (0) | 3.3E-03 | (0) | 5.1E-02 | (0) | 7.8E-01 | (0) |
| TPB50 | Women, normal weight | 1.2E-04 | (0) | 4.4E-02 | (0) | 1.4E-04 | (0) | 3.7E-02 | (0) | 1.6E-02 | (0) | 1.2E-05 | (0) | 5.2E-02 | (0) | 2.8E-05 | (0) | 2.2E-04 | (0) | 1.2E-03 | (0) | 3.1E-03 | (0) | 3.4E-02 | (0) |
| TPB51 | Women, normal weight | 4.3E-05 | (0) | 2.0E-01 | (0) | 8.7E-04 | (0) | 2.2E-02 | (0) | 2.0E-02 | (0) | 4.3E-05 | (0) | 2.2E-02 | (0) | 1.5E-04 | (0) | 2.1E-04 | (0) | 1.3E-03 | (0) | 1.5E-03 | (0) | 1.3E-01 | (0) |
| TPB52 | Women, normal weight | 2.6E-05 | (0) | 6.0E-02 | (0) | 5.4E-04 | (0) | 6.3E-02 | (0) | 1.5E-02 | (0) | 2.6E-05 | (0) | 3.5E-02 | (0) | 2.9E-04 | (0) | 1.2E-04 | (0) | 3.0E-03 | (0) | 1.6E-03 | (0) | 7.9E-02 | (0) |
| TPB53 | Women, normal weight | 2.0E-05 | (0) | 6.2E-02 | (0) | 5.0E-04 | (0) | 1.9E-01 | (0) | 3.7E-02 | (0) | 2.0E-05 | (0) | 9.8E-03 | (0) | 6.0E-04 | (0) | 1.1E-04 | (0) | 5.2E-04 | (0) | 5.7E-04 | (0) | 1.9E-02 | (0) |
| TPB54 | Women, normal weight | 7.4E-05 | (0) | 4.5E-02 | (0) | 5.2E-04 | (0) | 3.7E-02 | (0) | 1.5E-02 | (0) | 7.4E-05 | (0) | 3.7E-02 | (0) | 7.4E-05 | (0) | 7.4E-05 | (0) | 4.7E-04 | (0) | 3.7E-04 | (0) | 1.5E-02 | (0) |
|  | Children 3–6 yrs, underweight | 3.7E-04 | (0) | 2.2E-01 | (0) | 2.6E-03 | (1) | 1.8E-01 | (0) | 7.4E-02 | (0) | 3.7E-04 | (0) | 1.8E-01 | (0) | 3.7E-04 | (0) | 3.7E-04 | (0) | 2.3E-03 | (0) | 1.8E-03 | (0) | 7.4E-02 | (0) |
| TPB55 | Women, normal weight | 3.9E-05 | (0) | 4.7E-02 | (0) | 6.3E-04 | (0) | 7.5E-02 | (0) | 7.8E-03 | (0) | 3.9E-05 | (0) | 2.0E-02 | (0) | 1.8E-04 | (0) | 1.2E-04 | (0) | 2.5E-03 | (0) | 2.8E-03 | (0) | 6.8E-02 | (0) |
| TPB56 | Women, normal weight | 8.6E-04 | (0) | 1.5E+01 | (10) | 2.0E-02 | (7) | 9.7E-01 | (0) | 3.1E+00 | (2) | 8.9E-04 | (0) | 1.1E+00 | (0) | 9.5E-03 | (3) | 1.1E-02 | (1) | 9.5E-02 | (0) | 1.3E+00 | (1) | 2.9E+01 | (4) |
|  | Children 3–6 yrs, underweight | 4.3E-03 | (0) | 7.4E+01 | (52) | 9.8E-02 | (33) | 4.8E+00 | (2) | 1.5E+01 | (8) | 4.4E-03 | (0) | 5.6E+00 | (1) | 4.7E-02 | (13) | 5.7E-02 | (4) | 4.7E-01 | (0) | 6.6E+00 | (5) | 1.5E+02 | (18) |
| TPB57 | Women, normal weight | 1.3E-04 | (0) | 1.4E+02 | (96) | 1.2E-03 | (0) | 1.6E+00 | (1) | 1.4E+00 | (1) | 1.0E-03 | (0) | 1.4E-01 | (0) | 1.0E-03 | (0) | 6.3E-03 | (0) | 6.5E-03 | (0) | 2.3E+00 | (2) | 4.8E+00 | (1) |
|  | Children 3–6 yrs, underweight | 6.3E-04 | (0) | 6.8E+02 | (477) | 6.0E-03 | (2) | 7.9E+00 | (4) | 6.9E+00 | (3) | 5.1E-03 | (0) | 6.9E-01 | (0) | 5.2E-03 | (1) | 3.1E-02 | (2) | 3.2E-02 | (0) | 1.2E+01 | (8) | 2.4E+01 | (3) |
| TPB58 | Women, normal weight | 7.4E-05 | (0) | 3.4E+00 | (2) | 1.8E-03 | (1) | 2.9E+00 | (1) | 1.3E-01 | (0) | 7.4E-05 | (0) | 1.4E-01 | (0) | 1.8E-02 | (5) | 3.8E-02 | (3) | 1.7E-02 | (0) | 2.2E+00 | (2) | 1.1E+01 | (1) |
|  | Children 3–6 yrs, underweight | 3.7E-04 | (0) | 1.7E+01 | (12) | 8.8E-03 | (3) | 1.5E+01 | (7) | 6.6E-01 | (0) | 3.7E-04 | (0) | 7.0E-01 | (0) | 8.9E-02 | (25) | 1.9E-01 | (13) | 8.4E-02 | (0) | 1.1E+01 | (8) | 5.5E+01 | (7) |
| TPB59 | Women, normal weight | 2.0E-04 | (0) | 1.6E-01 | (0) | 1.4E-04 | (0) | 1.7E-01 | (0) | 1.3E-02 | (0) | 5.7E-05 | (0) | 3.7E-02 | (0) | 6.7E-04 | (0) | 3.2E-03 | (0) | 4.9E-03 | (0) | 1.6E-02 | (0) | 1.0E+01 | (1) |
| TPB60 | Women, normal weight | 8.5E-05 | (0) | 3.7E-01 | (0) | 1.3E-04 | (0) | 2.8E-02 | (0) | 6.0E-03 | (0) | 2.3E-05 | (0) | 3.9E-02 | (0) | 1.8E-05 | (0) | 3.0E-04 | (0) | 1.9E-03 | (0) | 3.5E-03 | (0) | 5.6E-02 | (0) |
| TPB61 | Women, normal weight | 3.7E-05 | (0) | 7.4E-03 | (0) | 7.4E-05 | (0) | 9.5E-02 | (0) | 7.4E-03 | (0) | 3.7E-05 | (0) | 1.8E-02 | (0) | 3.7E-05 | (0) | 1.1E-04 | (0) | 2.2E-04 | (0) | 1.4E-02 | (0) | 7.4E-03 | (0) |
| TPB62 | Women, normal weight | 3.0E-04 | (0) | 3.0E-01 | (0) | 5.1E-03 | (2) | 7.8E-01 | (0) | 2.3E-01 | (0) | 3.0E-04 | (0) | 1.5E-01 | (0) | 3.0E-04 | (0) | 1.5E-03 | (0) | 3.8E-03 | (0) | 1.5E-01 | (0) | 6.7E-01 | (0) |
|  | Children 3–6 yrs, underweight | 7.4E-04 | (0) | 7.6E-01 | (1) | 1.3E-02 | (4) | 1.9E+00 | (1) | 5.7E-01 | (0) | 7.4E-04 | (0) | 3.7E-01 | (0) | 7.4E-04 | (0) | 3.7E-03 | (0) | 9.5E-03 | (0) | 3.6E-01 | (0) | 1.7E+00 | (0) |

**Table S3.D, part 2 (of 2)**

| **Product** | **Group** | **Hg** | | **Mn** | | **Mo** | | **Ni** | | **Pb** | | **Sb** | | **Se** | | **Sn** | | **Sr** | | **U** | | **Zn** | | **REE** | |
| --- | --- | --- | --- | --- | --- | --- | --- | --- | --- | --- | --- | --- | --- | --- | --- | --- | --- | --- | --- | --- | --- | --- | --- | --- | --- |
| TPB01 | Women, normal weight | 4.5E-05 | (0) | 1.4E-01 | (0) | 2.7E-03 | (0) | 9.7E-03 | (0) | 2.8E-03 | (1) | 4.5E-05 | (0) | 2.2E-03 | (0) | 4.5E-05 | (0) | 2.3E-01 | (0) | 4.3E-04 | (0) | 2.4E-01 | (0) | 4.0E-03 | (0) |
| TPB02 | Women, normal weight | 6.6E-06 | (0) | 1.2E-01 | (0) | 1.1E-02 | (0) | 4.1E-02 | (1) | 4.7E-04 | (0) | 9.8E-05 | (0) | 3.3E-04 | (0) | 3.2E-04 | (0) | 2.2E-01 | (0) | 8.2E-05 | (0) | 3.3E-01 | (0) | 1.5E-03 | (0) |
| TPB03 | Women, normal weight | 1.5E-04 | (0) | 1.5E+00 | (1) | 2.2E-02 | (0) | 4.0E-02 | (1) | 1.5E-02 | (3) | 1.7E-02 | (4) | 4.9E-03 | (0) | 2.4E-03 | (0) | 5.0E+00 | (1) | 2.4E-02 | (0) | 9.0E-01 | (0) | 1.9E-01 | (0) |
| TPB04 | Women, normal weight | 1.0E-05 | (0) | 1.0E-01 | (0) | 9.5E-04 | (0) | 3.3E-02 | (1) | 5.2E-03 | (1) | 7.6E-05 | (0) | 8.1E-01 | (16) | 6.4E-04 | (0) | 2.4E-02 | (0) | 2.4E-04 | (0) | 1.7E+02 | (34) | 1.2E-03 | (0) |
| TPB05 | Women, normal weight | 3.7E-04 | (0) | 9.6E-01 | (1) | 3.1E-03 | (0) | 9.6E-02 | (3) | 4.5E-03 | (1) | 3.5E-04 | (0) | 1.7E-03 | (0) | 1.0E-03 | (0) | 1.2E+00 | (0) | 8.1E-03 | (0) | 6.9E-01 | (0) | 2.1E-02 | (0) |
| TPB06 | Women, normal weight | 5.3E-06 | (0) | 9.4E+00 | (7) | 1.6E-03 | (0) | 1.1E-02 | (0) | 1.3E-03 | (0) | 7.7E-04 | (0) | 1.7E-03 | (0) | 3.8E-04 | (0) | 2.4E-01 | (0) | 1.2E-03 | (0) | 2.1E-01 | (0) | 9.6E-03 | (0) |
| TPB07 | Women, normal weight | 3.5E-04 | (0) | 1.8E+01 | (13) | 2.3E-03 | (0) | 3.3E-01 | (12) | 1.2E-02 | (2) | 8.3E-04 | (0) | 3.3E-03 | (0) | 8.3E-03 | (0) | 1.5E-01 | (0) | 4.2E-04 | (0) | 9.2E-01 | (0) | 1.5E-02 | (0) |
| TPB08 | Women, normal weight | 6.4E-05 | (0) | 9.4E+00 | (7) | 1.4E-02 | (0) | 5.0E-02 | (2) | 2.0E-02 | (4) | 4.5E-04 | (0) | 1.6E-02 | (0) | 1.6E-03 | (0) | 8.1E-02 | (0) | 2.9E-04 | (0) | 2.7E+00 | (1) | 1.9E-02 | (0) |
| TPB09 | Women, normal weight | 8.6E-06 | (0) | 3.9E+01 | (28) | 4.3E-01 | (9) | 4.5E-02 | (2) | 2.2E-03 | (0) | 1.3E-04 | (0) | 8.7E-01 | (17) | 9.0E-04 | (0) | 1.1E-01 | (0) | 1.3E-04 | (0) | 1.7E+02 | (34) | 1.7E-03 | (0) |
| TPB10 | Women, normal weight | 3.4E-06 | (0) | 4.0E-02 | (0) | 3.6E-04 | (0) | 2.0E-03 | (0) | 1.3E-04 | (0) | 2.8E-05 | (0) | 1.7E-04 | (0) | 4.2E-05 | (0) | 2.7E-02 | (0) | 1.4E-05 | (0) | 1.9E-02 | (0) | 1.5E-04 | (0) |
| TPB11 | Women, normal weight | 3.3E-06 | (0) | 3.8E-02 | (0) | 5.1E-04 | (0) | 2.6E-03 | (0) | 2.3E-04 | (0) | 3.1E-05 | (0) | 5.7E-04 | (0) | 8.2E-05 | (0) | 5.1E-02 | (0) | 1.4E-04 | (0) | 2.8E-02 | (0) | 9.3E-05 | (0) |
| TPB12 | Women, normal weight | 3.2E-04 | (0) | 2.4E+00 | (2) | 1.3E-02 | (0) | 5.8E-02 | (2) | 1.2E-03 | (0) | 1.5E-04 | (0) | 1.6E-02 | (0) | 1.2E-04 | (0) | 1.4E+00 | (0) | 3.8E-05 | (0) | 4.4E+00 | (1) | 5.4E-03 | (0) |
| TPB13 | Women, normal weight | 1.4E-05 | (0) | 1.4E-02 | (0) | 6.6E-04 | (0) | 8.3E-03 | (0) | 4.9E-04 | (0) | 1.6E-04 | (0) | 3.1E-04 | (0) | 5.6E-04 | (0) | 6.7E-02 | (0) | 1.1E-04 | (0) | 1.7E-02 | (0) | 4.7E-04 | (0) |
| TPB14 | Women, normal weight | 1.3E-05 | (0) | 4.6E-02 | (0) | 1.7E-03 | (0) | 1.6E-02 | (1) | 2.1E-03 | (0) | 1.7E-04 | (0) | 6.3E-04 | (0) | 1.3E-03 | (0) | 3.2E-02 | (0) | 2.4E-04 | (0) | 8.2E-02 | (0) | 2.2E-03 | (0) |
| TPB15 | Women, normal weight | 2.0E-05 | (0) | 2.1E+00 | (1) | 1.7E-03 | (0) | 1.4E-02 | (0) | 3.1E-04 | (0) | 2.0E-05 | (0) | 2.7E-03 | (0) | 2.0E-05 | (0) | 3.1E+00 | (1) | 8.8E-05 | (0) | 2.1E-01 | (0) | 1.9E-02 | (0) |
| TPB16 | Women, normal weight | 2.1E-05 | (0) | 6.2E-01 | (0) | 8.6E-03 | (0) | 4.7E-03 | (0) | 6.1E-04 | (0) | 3.1E-05 | (0) | 2.4E-03 | (0) | 9.3E-06 | (0) | 1.1E-01 | (0) | 1.1E-04 | (0) | 5.1E-01 | (0) | 3.9E-03 | (0) |
| TPB17 | Women, normal weight | 2.5E-05 | (0) | 1.8E+00 | (1) | 8.4E-04 | (0) | 1.0E-02 | (0) | 3.3E-03 | (1) | 8.6E-05 | (0) | 2.4E-04 | (0) | 3.5E-04 | (0) | 2.8E-02 | (0) | 7.9E-05 | (0) | 9.8E-02 | (0) | 3.2E-03 | (0) |
| TPB18 | Women, normal weight | 2.3E-05 | (0) | 1.4E+00 | (1) | 3.7E-03 | (0) | 2.2E-02 | (1) | 2.2E-03 | (0) | 2.1E-04 | (0) | 2.8E-03 | (0) | 8.5E-04 | (0) | 7.0E-02 | (0) | 6.3E-05 | (0) | 1.7E-01 | (0) | 2.2E-03 | (0) |
| TPB19 | Women, normal weight | 1.2E-05 | (0) | 2.1E+00 | (1) | 2.0E-03 | (0) | 1.7E-02 | (1) | 1.7E-03 | (0) | 5.8E-05 | (0) | 4.2E-02 | (1) | 1.4E-04 | (0) | 2.6E-01 | (0) | 3.3E-04 | (0) | 6.1E+00 | (1) | 2.2E-02 | (0) |
| TPB20 | Women, normal weight | 7.7E-06 | (0) | 2.7E-01 | (0) | 2.8E-02 | (1) | 1.1E-02 | (0) | 1.5E-03 | (0) | 7.7E-06 | (0) | 7.7E-03 | (0) | 7.7E-06 | (0) | 7.9E-02 | (0) | 1.0E-04 | (0) | 1.3E+00 | (0) | 2.2E-04 | (0) |
|  | Children 3–6 yrs, underweight | 3.8E-05 | (0) | 1.3E+00 | (1) | 1.4E-01 | (3) | 5.6E-02 | (2) | 7.2E-03 | (1) | 3.8E-05 | (0) | 3.8E-02 | (1) | 3.8E-05 | (0) | 3.9E-01 | (0) | 5.0E-04 | (0) | 6.3E+00 | (1) | 1.1E-03 | (0) |
| TPB21 | Women, normal weight | 1.9E-05 | (0) | 5.7E-01 | (0) | 2.5E-03 | (0) | 1.1E-02 | (0) | 2.1E-03 | (0) | 4.6E-05 | (0) | 2.1E-03 | (0) | 1.8E-04 | (0) | 2.6E-01 | (0) | 2.6E-04 | (0) | 1.8E-01 | (0) | 1.7E-02 | (0) |
|  | Children 3–6 yrs, underweight | 9.4E-05 | (0) | 2.8E+00 | (2) | 1.3E-02 | (0) | 5.5E-02 | (2) | 1.0E-02 | (2) | 2.3E-04 | (0) | 1.0E-02 | (0) | 8.8E-04 | (0) | 1.3E+00 | (0) | 1.3E-03 | (0) | 8.8E-01 | (0) | 8.6E-02 | (0) |
| TPB22 | Women, normal weight | 7.4E-06 | (0) | 3.8E+00 | (3) | 2.1E-03 | (0) | 1.1E-02 | (0) | 4.0E-03 | (1) | 3.8E-05 | (0) | 1.2E-03 | (0) | 3.9E-04 | (0) | 1.6E-01 | (0) | 1.4E-04 | (0) | 2.8E-01 | (0) | 2.4E-02 | (0) |
|  | Children 3–6 yrs, underweight | 3.7E-05 | (0) | 1.9E+01 | (13) | 1.1E-02 | (0) | 5.4E-02 | (2) | 2.0E-02 | (4) | 1.9E-04 | (0) | 6.0E-03 | (0) | 1.9E-03 | (0) | 7.9E-01 | (0) | 6.8E-04 | (0) | 1.4E+00 | (0) | 1.2E-01 | (0) |
| TPB23 | Women, normal weight | 1.2E-04 | (0) | 7.5E+00 | (5) | 9.9E-02 | (2) | 2.8E-01 | (10) | 1.2E-01 | (25) | 5.4E-03 | (1) | 5.9E-03 | (0) | 1.1E-02 | (0) | 2.0E+00 | (0) | 1.6E-03 | (0) | 1.1E+01 | (2) | 1.2E-01 | (0) |
|  | Children 3–6 yrs, underweight | 5.9E-04 | (0) | 3.7E+01 | (27) | 4.9E-01 | (10) | 1.4E+00 | (51) | 6.2E-01 | (124) | 2.7E-02 | (7) | 2.9E-02 | (1) | 5.3E-02 | (0) | 1.0E+01 | (2) | 7.8E-03 | (0) | 5.4E+01 | (11) | 6.0E-01 | (1) |
| TPB24 | Women, normal weight | 2.4E-04 | (0) | 4.1E+00 | (3) | 2.8E-03 | (0) | 9.5E-02 | (3) | 2.6E-02 | (5) | 2.6E-04 | (0) | 5.9E-03 | (0) | 1.2E-03 | (0) | 8.3E-01 | (0) | 9.3E-04 | (0) | 8.7E-01 | (0) | 1.6E-01 | (0) |
|  | Children 3–6 yrs, underweight | 1.2E-03 | (1) | 2.1E+01 | (15) | 1.4E-02 | (0) | 4.7E-01 | (17) | 1.3E-01 | (25) | 1.3E-03 | (0) | 3.0E-02 | (1) | 6.0E-03 | (0) | 4.2E+00 | (1) | 4.6E-03 | (0) | 4.3E+00 | (1) | 7.9E-01 | (1) |
| TPB25 | Women, normal weight | 1.1E-04 | (0) | 5.6E-01 | (0) | 3.4E-03 | (0) | 4.4E-02 | (2) | 4.3E-03 | (1) | 1.0E-04 | (0) | 4.0E-03 | (0) | 1.2E-04 | (0) | 2.5E+00 | (0) | 2.3E-04 | (0) | 5.1E-01 | (0) | 3.8E-02 | (0) |
|  | Children 3–6 yrs, underweight | 5.4E-04 | (0) | 2.8E+00 | (2) | 1.7E-02 | (0) | 2.2E-01 | (8) | 2.1E-02 | (4) | 5.0E-04 | (0) | 2.0E-02 | (0) | 6.0E-04 | (0) | 1.2E+01 | (2) | 1.1E-03 | (0) | 2.5E+00 | (1) | 1.9E-01 | (0) |
| TPB26 | Women, normal weight | 2.1E-04 | (0) | 1.5E+01 | (11) | 2.4E-01 | (5) | 6.2E-01 | (22) | 2.4E-03 | (0) | 2.1E-04 | (0) | 3.4E-01 | (7) | 2.1E-04 | (0) | 1.3E+01 | (2) | 2.1E-04 | (0) | 2.5E+01 | (5) | 1.1E-02 | (0) |
|  | Children 3–6 yrs, underweight | 1.0E-03 | (1) | 7.5E+01 | (54) | 1.2E+00 | (24) | 3.1E+00 | (111) | 1.2E-02 | (2) | 1.0E-03 | (0) | 1.7E+00 | (33) | 1.0E-03 | (0) | 6.5E+01 | (11) | 1.0E-03 | (0) | 1.2E+02 | (25) | 5.3E-02 | (0) |
| TPB27 | Women, normal weight | 7.8E-05 | (0) | 2.3E+00 | (2) | 2.7E-03 | (0) | 1.5E-02 | (1) | 8.3E-04 | (0) | 1.3E-03 | (0) | 1.8E-03 | (0) | 3.7E-05 | (0) | 9.3E-02 | (0) | 3.7E-05 | (0) | 3.8E-01 | (0) | 2.6E-03 | (0) |
|  | Children 3–6 yrs, underweight | 3.9E-04 | (0) | 1.2E+01 | (8) | 1.3E-02 | (0) | 7.5E-02 | (3) | 4.1E-03 | (1) | 6.6E-03 | (2) | 9.2E-03 | (0) | 1.8E-04 | (0) | 4.6E-01 | (0) | 1.8E-04 | (0) | 1.9E+00 | (0) | 1.3E-02 | (0) |
| TPB28 | Women, normal weight | 1.3E-04 | (0) | 2.4E+00 | (2) | 3.1E-02 | (1) | 1.2E-01 | (4) | 1.0E-02 | (2) | 4.5E-04 | (0) | 6.3E-03 | (0) | 1.3E-04 | (0) | 1.7E+00 | (0) | 1.6E-03 | (0) | 2.3E+00 | (0) | 3.8E-02 | (0) |
|  | Children 3–6 yrs, underweight | 6.3E-04 | (0) | 1.2E+01 | (8) | 1.5E-01 | (3) | 6.0E-01 | (22) | 5.2E-02 | (10) | 2.2E-03 | (1) | 3.1E-02 | (1) | 6.3E-04 | (0) | 8.5E+00 | (1) | 8.0E-03 | (0) | 1.1E+01 | (2) | 1.9E-01 | (0) |
| TPB29 | Women, normal weight | 1.3E-03 | (1) | 2.5E+01 | (18) | 1.6E-01 | (3) | 1.2E-01 | (4) | 5.5E-03 | (1) | 2.1E-04 | (0) | 3.7E-03 | (0) | 7.4E-05 | (0) | 1.2E+01 | (2) | 7.4E-05 | (0) | 1.6E+01 | (3) | 3.0E-02 | (0) |
|  | Children 3–6 yrs, underweight | 6.2E-03 | (3) | 1.2E+02 | (88) | 7.8E-01 | (16) | 6.1E-01 | (22) | 2.7E-02 | (5) | 1.1E-03 | (0) | 1.8E-02 | (0) | 3.7E-04 | (0) | 5.7E+01 | (10) | 3.7E-04 | (0) | 7.8E+01 | (16) | 1.5E-01 | (0) |
| TPB30 | Women, normal weight | 5.8E-05 | (0) | 5.3E-02 | (0) | 6.6E-04 | (0) | 2.9E-03 | (0) | 2.5E-04 | (0) | 5.8E-05 | (0) | 2.9E-03 | (0) | 5.8E-05 | (0) | 1.1E-01 | (0) | 5.8E-05 | (0) | 4.0E+02 | (79) | 9.3E-04 | (0) |
| TPB31 | Women, normal weight | 5.6E-05 | (0) | 4.4E-01 | (0) | 8.5E-03 | (0) | 7.7E-03 | (0) | 3.3E-03 | (1) | 5.6E-05 | (0) | 2.8E-03 | (0) | 5.6E-05 | (0) | 4.0E-01 | (0) | 3.1E-04 | (0) | 1.1E+00 | (0) | 3.4E-03 | (0) |

**Table S3.D, part 2 (of 2)**, *continued*

| **Product** | **Group** | **Hg** | | **Mn** | | **Mo** | | **Ni** | | **Pb** | | **Sb** | | **Se** | | **Sn** | | **Sr** | | **U** | | **Zn** | | **REE** | |
| --- | --- | --- | --- | --- | --- | --- | --- | --- | --- | --- | --- | --- | --- | --- | --- | --- | --- | --- | --- | --- | --- | --- | --- | --- | --- |
| TPB32 | Women, normal weight | 1.8E-05 | (0) | 2.9E-02 | (0) | 4.3E-04 | (0) | 2.6E-02 | (1) | 1.3E-03 | (0) | 7.7E-03 | (2) | 3.2E-04 | (0) | 1.5E-04 | (0) | 6.8E-01 | (0) | 2.3E-04 | (0) | 4.7E+01 | (9) | 3.9E-02 | (0) |
| TPB33 | Women, normal weight | 3.8E-05 | (0) | 1.2E-01 | (0) | 2.2E-03 | (0) | 2.4E-03 | (0) | 4.0E-04 | (0) | 1.3E-03 | (0) | 2.9E-04 | (0) | 6.7E-05 | (0) | 2.0E-01 | (0) | 7.7E-04 | (0) | 2.2E-02 | (0) | 8.1E-03 | (0) |
| TPB34 | Women, normal weight | 6.5E-06 | (0) | 6.1E-02 | (0) | 5.6E-04 | (0) | 6.3E-03 | (0) | 3.0E-03 | (1) | 1.7E-02 | (4) | 3.3E-04 | (0) | 4.3E-05 | (0) | 1.3E+00 | (0) | 4.8E-04 | (0) | 2.9E-02 | (0) | 8.1E-02 | (0) |
| TPB35 | Women, normal weight | 4.4E-05 | (0) | 1.4E-01 | (0) | 1.5E-03 | (0) | 1.2E-02 | (0) | 6.6E-04 | (0) | 1.1E-02 | (3) | 7.9E-04 | (0) | 2.1E-04 | (0) | 7.7E-01 | (0) | 8.3E-04 | (0) | 3.7E-02 | (0) | 2.8E-02 | (0) |
| TPB36 | Women, normal weight | 9.2E-05 | (0) | 8.1E-01 | (1) | 2.2E-03 | (0) | 6.7E-02 | (2) | 2.8E-02 | (6) | 7.7E-04 | (0) | 2.8E-03 | (0) | 6.3E-03 | (0) | 2.5E-01 | (0) | 5.8E-04 | (0) | 3.9E-01 | (0) | 5.7E-02 | (0) |
| TPB37 | Women, normal weight | 1.1E-04 | (0) | 1.2E+00 | (1) | 2.5E-02 | (1) | 8.7E-02 | (3) | 3.9E-03 | (1) | 1.1E-03 | (0) | 2.0E-03 | (0) | 2.1E-03 | (0) | 7.8E-01 | (0) | 1.3E-02 | (0) | 1.3E+00 | (0) | 6.7E-02 | (0) |
| TPB38 | Women, normal weight | 1.1E-05 | (0) | 2.1E-01 | (0) | 1.3E-03 | (0) | 1.0E-02 | (0) | 6.3E-04 | (0) | 9.0E-05 | (0) | 5.5E-04 | (0) | 2.3E-04 | (0) | 2.8E-02 | (0) | 8.1E-04 | (0) | 6.2E-01 | (0) | 1.3E-03 | (0) |
| TPB39 | Women, normal weight | 8.3E-06 | (0) | 8.8E-01 | (1) | 7.2E-03 | (0) | 2.3E-02 | (1) | 1.5E-03 | (0) | 8.7E-05 | (0) | 9.1E-01 | (18) | 2.3E-04 | (0) | 7.5E-02 | (0) | 3.1E-04 | (0) | 1.6E+02 | (31) | 2.8E-03 | (0) |
| TPB40 | Women, normal weight | 1.0E-04 | (0) | 1.6E+01 | (12) | 1.4E-03 | (0) | 2.8E-01 | (10) | 4.0E-03 | (1) | 3.4E-04 | (0) | 1.0E-03 | (0) | 7.1E-04 | (0) | 4.4E-02 | (0) | 1.0E-03 | (0) | 4.8E-01 | (0) | 1.0E-02 | (0) |
| TPB41 | Women, normal weight | 7.0E-05 | (0) | 6.0E-01 | (0) | 4.9E-03 | (0) | 9.4E-03 | (0) | 5.8E-03 | (1) | 3.4E-03 | (1) | 5.0E-01 | (10) | 4.8E-04 | (0) | 6.4E-01 | (0) | 1.8E-03 | (0) | 2.3E+01 | (5) | 2.7E-02 | (0) |
| TPB42 | Women, normal weight | 1.5E-05 | (0) | 6.7E-03 | (0) | 2.2E-04 | (0) | 7.5E-04 | (0) | 1.1E-02 | (2) | 1.4E-04 | (0) | 7.5E-04 | (0) | 2.5E-04 | (0) | 6.5E-03 | (0) | 6.2E-05 | (0) | 5.1E-02 | (0) | 1.9E-03 | (0) |
|  | Children 3–6 yrs, underweight | 7.4E-05 | (0) | 3.3E-02 | (0) | 1.1E-03 | (0) | 3.7E-03 | (0) | 5.2E-02 | (10) | 7.0E-04 | (0) | 3.7E-03 | (0) | 1.2E-03 | (0) | 3.2E-02 | (0) | 3.1E-04 | (0) | 2.5E-01 | (0) | 9.5E-03 | (0) |
| TPB43 | Women, normal weight | 4.3E-05 | (0) | 3.5E-02 | (0) | 9.0E-04 | (0) | 1.2E-02 | (0) | 9.6E-04 | (0) | 1.4E-04 | (0) | 2.2E-03 | (0) | 3.3E-04 | (0) | 3.1E-02 | (0) | 9.3E-05 | (0) | 1.3E-01 | (0) | 9.6E-04 | (0) |
| TPB44 | Women, normal weight | 6.8E-05 | (0) | 6.7E-01 | (0) | 5.0E-03 | (0) | 6.6E-02 | (2) | 4.9E-03 | (1) | 4.5E-04 | (0) | 3.1E-03 | (0) | 4.0E-03 | (0) | 5.8E-02 | (0) | 3.0E-04 | (0) | 1.0E+00 | (0) | 7.6E-03 | (0) |
| TPB45 | Women, normal weight | 1.1E-05 | (0) | 9.0E+00 | (6) | 4.2E-04 | (0) | 4.8E-02 | (2) | 1.4E-03 | (0) | 3.8E-04 | (0) | 5.3E-04 | (0) | 5.0E-04 | (0) | 5.6E-02 | (0) | 1.4E-04 | (0) | 5.1E-01 | (0) | 5.9E-03 | (0) |
| TPB46 | Women, normal weight | 2.3E-06 | (0) | 2.5E-02 | (0) | 9.0E-04 | (0) | 1.3E-03 | (0) | 6.7E-04 | (0) | 5.6E-05 | (0) | 1.2E-04 | (0) | 4.2E-05 | (0) | 4.3E-02 | (0) | 1.8E-05 | (0) | 1.7E-02 | (0) | 1.3E-03 | (0) |
| TPB47 | Women, normal weight | 8.0E-06 | (0) | 8.4E-02 | (0) | 5.0E-04 | (0) | 3.2E-03 | (0) | 1.5E-04 | (0) | 7.3E-05 | (0) | 4.0E-04 | (0) | 3.8E-05 | (0) | 2.6E-02 | (0) | 3.1E-05 | (0) | 1.6E-02 | (0) | 3.0E-04 | (0) |
| TPB48 | Women, normal weight | 1.0E-04 | (0) | 4.8E-01 | (0) | 6.2E-03 | (0) | 1.5E-02 | (1) | 5.7E-03 | (1) | 3.1E-04 | (0) | 2.9E-03 | (0) | 1.5E-04 | (0) | 2.3E-01 | (0) | 1.3E-04 | (0) | 1.5E+00 | (0) | 6.9E-03 | (0) |
| TPB49 | Women, normal weight | 5.4E-06 | (0) | 7.0E-02 | (0) | 1.4E-03 | (0) | 4.7E-03 | (0) | 7.8E-04 | (0) | 3.7E-05 | (0) | 2.7E-04 | (0) | 5.7E-05 | (0) | 4.7E-01 | (0) | 5.6E-04 | (0) | 3.0E-01 | (0) | 3.0E-03 | (0) |
| TPB50 | Women, normal weight | 1.2E-05 | (0) | 5.2E-03 | (0) | 2.8E-04 | (0) | 6.0E-04 | (0) | 2.4E-04 | (0) | 6.5E-05 | (0) | 6.0E-04 | (0) | 1.6E-04 | (0) | 9.5E-03 | (0) | 4.3E-05 | (0) | 5.8E-02 | (0) | 3.4E-04 | (0) |
| TPB51 | Women, normal weight | 4.3E-05 | (0) | 9.8E-03 | (0) | 2.2E-04 | (0) | 2.2E-03 | (0) | 5.3E-04 | (0) | 1.3E-04 | (0) | 2.2E-03 | (0) | 6.4E-03 | (0) | 2.3E-02 | (0) | 3.1E-04 | (0) | 1.8E-02 | (0) | 1.1E-03 | (0) |
| TPB52 | Women, normal weight | 2.6E-05 | (0) | 3.6E-03 | (0) | 3.7E-04 | (0) | 1.3E-03 | (0) | 2.6E-04 | (0) | 8.1E-05 | (0) | 1.3E-03 | (0) | 4.3E-04 | (0) | 6.1E-02 | (0) | 5.4E-05 | (0) | 2.8E-02 | (0) | 4.5E-04 | (0) |
| TPB53 | Women, normal weight | 2.0E-05 | (0) | 6.3E-03 | (0) | 2.1E-04 | (0) | 9.8E-04 | (0) | 1.2E-04 | (0) | 2.2E-04 | (0) | 9.8E-04 | (0) | 2.0E-03 | (0) | 1.1E-02 | (0) | 6.0E-05 | (0) | 3.3E-02 | (0) | 5.5E-04 | (0) |
| TPB54 | Women, normal weight | 7.4E-05 | (0) | 4.3E-03 | (0) | 3.7E-04 | (0) | 3.7E-03 | (0) | 7.4E-05 | (0) | 7.4E-05 | (0) | 3.7E-03 | (0) | 7.4E-05 | (0) | 4.1E-03 | (0) | 7.4E-05 | (0) | 3.9E-02 | (0) | 1.2E-03 | (0) |
|  | Children 3–6 yrs, underweight | 3.7E-04 | (0) | 2.1E-02 | (0) | 1.8E-03 | (0) | 1.8E-02 | (1) | 3.7E-04 | (0) | 3.7E-04 | (0) | 1.8E-02 | (0) | 3.7E-04 | (0) | 2.1E-02 | (0) | 3.7E-04 | (0) | 2.0E-01 | (0) | 5.9E-03 | (0) |
| TPB55 | Women, normal weight | 3.9E-05 | (0) | 2.5E-02 | (0) | 4.0E-04 | (0) | 2.0E-03 | (0) | 3.8E-04 | (0) | 1.4E-04 | (0) | 2.0E-03 | (0) | 4.1E-04 | (0) | 7.5E-02 | (0) | 3.9E-05 | (0) | 1.0E-01 | (0) | 1.4E-03 | (0) |
| TPB56 | Women, normal weight | 5.0E-04 | (0) | 8.2E+00 | (6) | 3.9E-01 | (8) | 1.4E-01 | (5) | 8.4E-02 | (17) | 2.0E-03 | (0) | 5.5E-03 | (0) | 2.3E-02 | (0) | 2.2E+00 | (0) | 1.1E-03 | (0) | 5.5E+00 | (1) | 9.4E-02 | (0) |
|  | Children 3–6 yrs, underweight | 2.5E-03 | (1) | 4.1E+01 | (29) | 1.9E+00 | (39) | 7.0E-01 | (25) | 4.2E-01 | (83) | 9.9E-03 | (2) | 2.8E-02 | (1) | 1.1E-01 | (0) | 1.1E+01 | (2) | 5.5E-03 | (0) | 2.7E+01 | (5) | 4.7E-01 | (1) |
| TPB57 | Women, normal weight | 5.4E-04 | (0) | 7.8E+01 | (56) | 1.9E-03 | (0) | 2.0E-01 | (7) | 6.5E-03 | (1) | 2.6E-04 | (0) | 1.8E-03 | (0) | 9.7E-04 | (0) | 9.2E-01 | (0) | 2.3E-04 | (0) | 1.8E+00 | (0) | 1.6E-01 | (0) |
|  | Children 3–6 yrs, underweight | 2.7E-03 | (1) | 3.9E+02 | (278) | 9.3E-03 | (0) | 9.8E-01 | (35) | 3.2E-02 | (6) | 1.3E-03 | (0) | 9.2E-03 | (0) | 4.8E-03 | (0) | 4.6E+00 | (1) | 1.2E-03 | (0) | 9.0E+00 | (2) | 8.0E-01 | (1) |
| TPB58 | Women, normal weight | 7.4E-05 | (0) | 4.2E+00 | (3) | 1.4E-01 | (3) | 1.6E-01 | (6) | 2.6E-03 | (1) | 2.2E-04 | (0) | 8.1E-03 | (0) | 7.4E-05 | (0) | 1.4E+00 | (0) | 7.4E-05 | (0) | 8.9E+00 | (2) | 1.2E-02 | (0) |
|  | Children 3–6 yrs, underweight | 3.7E-04 | (0) | 2.1E+01 | (15) | 7.0E-01 | (14) | 7.8E-01 | (28) | 1.3E-02 | (3) | 1.1E-03 | (0) | 4.1E-02 | (1) | 3.7E-04 | (0) | 7.1E+00 | (1) | 3.7E-04 | (0) | 4.4E+01 | (9) | 5.9E-02 | (0) |
| TPB59 | Women, normal weight | 1.2E-04 | (0) | 1.9E-01 | (0) | 2.9E-03 | (0) | 6.7E-03 | (0) | 2.6E-03 | (1) | 2.4E-04 | (0) | 1.0E+00 | (21) | 3.4E-04 | (0) | 1.2E-01 | (0) | 6.8E-04 | (0) | 2.4E+02 | (48) | 3.9E-03 | (0) |
| TPB60 | Women, normal weight | 1.5E-05 | (0) | 2.5E-01 | (0) | 1.1E-04 | (0) | 5.0E-03 | (0) | 3.0E-04 | (0) | 2.6E-05 | (0) | 4.7E-04 | (0) | 9.0E-05 | (0) | 7.7E-03 | (0) | 2.4E-05 | (0) | 8.4E-02 | (0) | 7.7E-04 | (0) |
| TPB61 | Women, normal weight | 3.7E-05 | (0) | 5.5E-03 | (0) | 1.8E-04 | (0) | 1.8E-03 | (0) | 3.7E-05 | (0) | 3.7E-05 | (0) | 1.8E-03 | (0) | 1.4E-04 | (0) | 2.0E-03 | (0) | 3.7E-05 | (0) | 1.5E-02 | (0) | 5.9E-04 | (0) |
| TPB62 | Women, normal weight | 3.0E-04 | (0) | 6.4E-01 | (0) | 4.9E-03 | (0) | 1.5E-02 | (1) | 2.0E-03 | (0) | 1.1E-03 | (0) | 1.5E-02 | (0) | 7.0E-04 | (0) | 2.1E-01 | (0) | 3.0E-04 | (0) | 1.8E-01 | (0) | 1.0E-02 | (0) |
|  | Children 3–6 yrs, underweight | 7.4E-04 | (0) | 1.6E+00 | (1) | 1.2E-02 | (0) | 3.7E-02 | (1) | 4.9E-03 | (1) | 2.7E-03 | (1) | 3.7E-02 | (1) | 1.7E-03 | (0) | 5.3E-01 | (0) | 7.4E-04 | (0) | 4.6E-01 | (0) | 2.5E-02 | (0) |
